# Prevalence of caregiver hesitancy for vaccinations in children and its associated factors: A systematic review and meta-analysis

**DOI:** 10.1371/journal.pone.0302379

**Published:** 2024-10-24

**Authors:** Nur Hasnah Maamor, Nor Asiah Muhamad, Nor Soleha Mohd Dali, Fatin Norhasny Leman, Izzah Athirah Rosli, Tengku Puteri Nadiah Tengku Bahrudin Shah, Nurul Hidayah Jamalluddin, Nurul Syazwani Misnan, Zuraifah Asrah Mohamad, Sophia Karen Bakon, Mohd Hatta Abdul Mutalip, Muhammad Radzi Abu Hassan, Nai Ming Lai

**Affiliations:** 1 National Institutes of Health, Sector for Evidence-based Healthcare, Ministry of Health, Setia Alam, Malaysia; 2 Institute for Medical Research, National Institutes of Health, Ministry of Health, Setia Alam, Malaysia; 3 Institute for Public Health, National Institutes of Health, Ministry of Health, Setia Alam, Malaysia; 4 Office of Director General Ministry of Health, Putrajaya, Malaysia; 5 School of Medicine, Taylor’s University, Subang Jaya, Malaysia; Regional Health Care and Social Agency of Lodi, ITALY

## Abstract

This review aimed to systematically compare and pool the prevalence of all the known evidence on caregiver hesitancy and to describe the factors influencing vaccine hesitancy among caregiver worldwide such as COVID-19, MMR, Influenza, HPV and others. We searched article from few electronic databases (PubMed, CENTRAL, ProQuest, and Web of Science) from inception to August 2023 using specific keywords for example caregiver, parents, prevalence, factor, hesitancy, and others. We included population-based studies that reported the prevalence of caregiver hesitancy. We used random-effects meta-analyses for pool prevalence estimates of caregiver hesitancy. A total of 765 studies met our inclusion criteria, containing data on 38,210,589 caregivers from seven regions across the globe. Overall or pool prevalence of vaccine hesitancy among caregiver is 25.0% (95% CI: 0.22–0.27, I^2^ = 99.91%, p = 0.001). Based on the evidence gathered, vaccine hesitancy was found to be religious sentiments, personal beliefs, perceived safety concerns, and a desire for more information from healthcare providers, along with factors related to availability, accessibility, affordability, and acceptability of vaccinations. Vaccine safety and efficiency have been identified as the main factor for caregiver vaccine hesitancy globally with a prevalence of 91.4%.

Trial registration

PROSPERO registration number: CRD42022331629. https://www.crd.york.ac.uk/prospero/display_record.php?ID=CRD42022331629.

## Introduction

Vaccination is considered one of the most accomplished methods for communicable disease prevention, which has resulted in the reduction of mortality and improvement the quality of life in the population [1]. This method is one of the most effective ways of controlling communicable diseases [2]. Over the past few years, global vaccination coverage has been reported as remaining steady [3,4]. Vaccination coverage among children refers to the proportion of children who have received the recommended vaccination [5,6]. With well-defined target groups and outreach activities, adequate vaccination coverage can be administered methodically and effectively. Hence, it is easily adaptable and feasible to implement [4,5]. Vaccine hesitancy, on the other hand, is defined by the World Health Organization (WHO) as delaying accepting or refusing or declining vaccine despite the availability of vaccination services [4,7]. It is complex and context-specific, varying according to time, geography, and vaccination type. Factors like complacency, convenience, and confidence have an impact on this occurrence [7]. The prevalence of vaccine hesitancy among caregiver for the children has been reported differently for different type of vaccine worldwide for example (i) COVID-19 has been reported to have prevalence between 1.0–89.0% [8,9]; (ii) HPV between 2.0–95.0% [10,11]); (iii) Influenza between 3.3–95.0% [12,13]; (iv) Mix between 0.5–88% [14,15] and (v) MMR between 1.9–76.0% [16,17]. Few examples of countries and regions that reported with caregiver vaccine hesitancy are shown at **Table 1.**

**Table 1:** The approximate prevalence of vaccine hesitancy by regions.

	Country	Region	Approximately prevalence (%)	Reference
Covid-19				
	Ghana	Africa	26.7	Kyei-Arthur et al 2022 [18]
	Taiwan	Asia	1	Tsai et al 2022 [19]
	China		85.3	Zhou et al 2021 [20]
	Italy	Europe	89.4	Savarese et al 2022 [21]
	Latin America, Caribbean	Mix	7.8	Urrunaga et al 2021 [22]
	USA, Canada, Israel, Japan, Spain, Switzerland		62.7	Goldman et al 2022 [23]
	USA	North America	1.6–68.0	Teasdale et al 2021 [24]
	USA			Temple et al 2022 [25]
	Australia	Oceania	9–30.4	Bolsewicz et al 2023 [26]
	New Zealand			Jeffs et al 2021 [27]
HPV				
	Nigeria	Africa	61.0	Balogun et al 2022 [28]
	South Africa		3.2	Milondzo et al 2022 [29]
	Japan	Asia	2.0	Ugumori et al 2021 [30]
	China		95.0	Huang et al 2021 [31]
	England	Europe	3.6	Taylor et al 2022 [32]
	Italy		33.0	Della et al 2020 [33]
	USA	North America	63.0	Dorell et al 2014 [34]
	USA		2.1	Hirth et al 2019 [35]
Influenza				
	Saudi Arabia	Asia	3.3	Alenazi et al 2022 [36]
	Saudi Arabia		75.0	AlOmran et al 2022 [37]
	Scotland	Europe	7	Bielecki et al 2020 [38]
	Italy		95.0	Prospero et al 2019 [39]
	USA	North America	6.3	Kempe et al 2020 [40]
	USA		68.0	Nguyen et al 2022 [41]
	Australia	Oceania	17.0	Jones et al 1992 [42]
Mix				
	Uganda	Africa	40.0	Kamya et al 1992 [43]
	Mozambique		1.0	Powelson et al 2022 [44]
	Malaysia	Asia	0.5	Chan et al 2018 [45]
	Malaysia		88.0	Zin ZM et al 2022 [46]
	Romania	Europe	1.0	Miron et al 2022 [47]
	Italy		50.0	Caso et al 2021 [48]
	Turkey	Mix	3.5	Durmaz et al 2022 [49]
	Turkey		14.9	Yilmaz et al 2023 [50]
	USA	North America	2	Navin et al 2019 [51]
	Australia	Oceania	11.0	Forbes et al 2015 [52]
	New Zealand		16.3	Debela et al 2022 [53]
	Brazil	South America	5.0	Olbrich Neto et al 2023 [54]
	Venezuela		37.0	Burghouts et al 2017 [55]
MMR				
	Uganda	Africa	15.1	Griffith et al 2022 [56]
	Nigeria		16.1	Cockcroft et al 2014 [57]
	Israel		12.0	Ashkenazi et al 2020 [58]
	United Kingdom	Europe	2.0	Campbell et al 2017 [59]
	Wales		76.0	Roberts et al 1995 [60]
	USA	North America	2.0	Cataldi et al 2016 [61]
	USA		83.1	Wharton et al 2019 [62]

The success of the children’s vaccination program does not only depend on adequate immunization coverage but also on parental willingness to vaccinate their children. Many caregivers have been concerned about actual or perceived vaccine adverse events from vaccination which make them hesitant to vaccinate their children [63–65].

Lack of knowledge among caregivers and their beliefs regarding the effect and contraindications of immunization may lead to hesitancy to allow their children to be vaccinated [66–69]. Many caregivers are more concerned about the adverse events related to vaccines that outweigh the perceived benefits that vaccines might bring to their children, which are thought to be more frequent and serious than the actual effect or the complications that could arise from vaccine preventable diseases [70]. Additionally, caregiver hesitancy can be due to other factors such as missing the children’s vaccine appointment [71], vaccine shortage, and difficulty to access the health facilities. Therefore, awareness programs and knowledge regarding vaccination are crucial to convince the caregivers of its importance and give a better understanding to the caregivers who are hesitant to accept vaccinations.

Numerous studies have been conducted to identify various determinants and factors that influence vaccination decisions that contribute to vaccine hesitancy [72,73]. However, there is no single review on the overall prevalence and hesitancy reasons for different types of vaccine among caregiver discussed in one paper. Additionally, vaccine hesitancy finding across the global is limited, potentially limiting the evidence of these studies have on evidence informed policymaking. Therefore, this review aims to systematically synthesized all the published evidence on the prevalence and determine the reasons associated with caregiver hesitancy globally.

## Materials and methods

We followed the Preferred Reporting Items for Systematic Reviews and Meta-Analyses (PRISMA) guidelines [74].

### Search methods for identification of studies

We searched four electronic databases namely PubMed, CENTRAL, ProQuest, and Web of Science for observational studies reporting the prevalence and reasons for vaccine hesitancy in children among caregivers (NHM, IAR, NSMD, and FNL). Caregiver is defined as a person who is taking care of their children and able to give consent about the children’s health to healthcare providers. This term can alternatively with parent, parental or guardians. We also used free text terms as “vaccine, immuniz(s)e, shot, jab, hesitant, reluctant, confidence, acceptance, reject, delay, comply, and uptake” in this review. The full search syntax is shown in the **S1 Table**. We hand-searched reference lists of all the included studies to identify other relevant studies.

### Eligibility criteria

We included studies according to the criteria or guideline for meta-analysis of prevalence approach [75]; condition or problem, context and population (CoCoPop). We included population studies that provide the prevalence estimates of caregiver hesitancy under condition (Co). Factors that contribute to the outcome such as caregiver knowledge, information, attitudes, safety and efficiency as well as beliefs regarding vaccines is identify under context (Co). In this review, we identified the population (Pop) as caregivers/guardians/parents of children requiring vaccination. We included all studies with information either on prevalence or factors or reasons that contribute to the parental vaccine hesitancy, those studies will be included. The detailed on the CoCoPop is stated in **S2 Table**.

We included studies with the information on either caregivers, parents or guardians with children between the age of 0–17 years old with or without the prevalence and/or factors that contributed to the caregiver vaccine hesitancy. We listed all the factors that contributed to the caregiver vaccine hesitancy and categorized the factors into five main components: (i) regional belief, (ii) knowledge, (iii) information, (iv) safety and efficiency and (v) others after discussing with the other authors.

We excluded abstracts, letters to the editor, reviews, commentaries, editorials, and studies without either primary data or descriptions of study methodologies. Systematic reviews, non-empirical studies, conferences, abstracts, editorials, commentaries, book reviews, and abstracts that were not accompanied by a full text were also excluded. We excluded studies that focused on the seasonal character of the vaccine program when the study population was not caregivers, parents or guardians, or if vaccine requirements deviated from the general population routine recommendations. We also excluded all non-English studies or when the population were not children or adolescents.

### Study selection process

Two authors (NHM & NSM) independently screened all the titles and abstracts to examine all the potential studies we identified and coded them either “retrieve” (eligible or potentially eligible/unclear) or “do not retrieve”. After we retrieved the full-text reports/publications, and two additional review authors (TPNTB & NHJ) independently screened the full text to identify the eligible studies and recorded reasons for exclusion of the ineligible studies. We resolved any disagreements through discussion or by consulting third review author (NAM) for the final decision. We identified, removed duplicates, and compiled multiple reports of the same study. We recorded the selection process in sufficient detail to complete a PRISMA flow diagram as shown in **S1 Fig.**

### Data extraction and management

Two review authors (NM and NSMD) independently extracted the outcome data. We resolved disagreements through a discussion among the authors. We consulted third review author (NML) if disagreement persisted. We summarized and reported the results based on the reasons and prevalence number of vaccine hesitancy among caregiver. We followed the strategies in the Cochrane Handbook for Systematic Reviews of Interventions for data management [76].

### Data item

Information from each paper was extracted that include the main author’s name, year of publication, country, region, study design, total number of respondents, and reason(s) for refusal as shown in **Table 2**.

**Table 2:** Data collection form for study characteristics and outcome data.

No	Author	Year of publication	Country	Region	Study design	Total of respondent	Religious belief	Knowledge	Information	Safety and efficacy	Others	Type of vaccine

### Quality assessment and risk of bias

The quality of the included studies was appraised independently by two review authors (FNL and IAR). Any disagreement was resolved through discussion or referred to third author (MHAM) when issues persisted. Risk of bias was assessed independently by two authors (NSMD, FNL) using the appropriate Newcastle-Ottawa scale (NOS) for cohort and case control studies [77] with disagreements resolved by consensus with the other author (NAM). The NOS scale evaluates research in three components: assessing studies on participant selection, comparability, and outcome/exposure assessment. A study is awarded stars for items in each category with the maximum of nine stars. We determined that studies with nine stars would have a low risk of bias, those with seven or eight stars would have a moderate risk of bias, and those with fewer stars would have a high risk of bias. For cross-sectional studies, the quality of each study was assess using a modified NOS [77,78] while the Critical Appraisal Skills Programme or CASP Randomized Clinical Trial was used for randomized clinical trial (RCT) study design [79]. Risk of bias was identified using the Egger test by observing the *p*-value. If p is <0.10, it indicates the present of publication bias. While p>0.10, it suggests that there is no publication bias [80].

### Data synthesis and analysis

We used Stata software version 17 for statistical analysis [81]. We considered studies to be sufficiently similar, namely, with the same category of condition, context and population as defined above. Our primary outcome was a prevalence of vaccine hesitancy among caregivers for children. We conducted a meta-analysis by pooling the appropriate data using random effect model to calculate the pooled prevalence with their 95% confidence intervals [81,82]. The heterogeneity was assessed using I^2^ statistics and Cochran’s Q test [83]. The I^2^ statistics were used to evaluate the explained variance attributable to study heterogeneity, with I^2^ scores of 25.0, 50.0, and 75.0% respectively, indicate low, moderate, and high [81–84]. We did not perform any subgroup analyses due to insufficient data. The average frequency was used to identify the reason for vaccine hesitancy across the globe.

## Results

A total of 115,931 titles and abstracts were screened for inclusion. Of these, 115,031 reports were excluded. We retrieved a total of 900 full texts for inclusion. We further excluded 135 full texts based on our criteria, of which 765 were included in the final analysis (**S1 Fig**). **S1 Appendix** shows the list of the included study.

### Summary of studies included in the review

A total of 765 studies (N = 38,210,589) were included in this review and shows in the **S3 Table**. The number of participants ranged from 6 to 35,0256 caregivers. The number of studies were divided into six regions based on the study sites (**S1 Appendix**): 65 from Africa^1-64^, 258 from North America^65–322^, 241 from Asia^323–563^, 15 from Multiple or Mixed region ^564–578^, 24 from Oceania (Australia and New Zealand)^579-602^, 148 from Europe^603–750^ and 14 from South America^751-765^. Various types of vaccines were also assessed, such as (i) COVID-19; (ii) human papillomavirus (HPV); (iii) Influenza; (iv) Mumps, Measles & Rubella, or MMR; (v) Mixed vaccines- children who take more than one vaccine such as the Influenza and COVID-19, HPV and MMR and others; (vi) other vaccines such as Hepatitis A & B, Malaria, Polio, Diphtheria, pneumococcal and others. Studies that failed to specify or did not provide the vaccine names were group in not stated vaccines. All included studies that reported the reason for the caregiver vaccine hesitancy across the region are shown in **S2 Fig.** From 765 studies, 434 studies reported on the prevalence of caregiver hesitancy across the region (**S3 Fig**).

### Quality assessment of included studies

Two review authors (FNL and MHAM) independently assessed the quality of the included studies using NOS for cohort (40 studies), case-control (9 studies), an adapted version of the NOS for cross-sectional (711 studies), and Critical Appraisal Skills Programme or CASP Randomized Clinical Trial (5 studies). The quality of evidence for NOS was rated as poor, fair, and good quality while for CASP it was rated either poor, moderate, or good quality. Summary of the quality assessment score shows at Table 3 while the detailed list of studies included in this quality assessment is listed in the **S4 Table**.

**Table 3:** Summary of the quality assessment score.

Study design	Score		
	Poor	Fair	Good
Cohort (N = 40)	4	20	16
Case-control (N = 9)	4	4	1
Cross-sectional (N = 711)	146	203	362
	Poor	Moderate	Good
Randomized Clinical Trial (N = 5)	0	1	4

### The estimates of pooled or overall prevalence of overall vaccine hesitancy

A total of 434 studies reported the prevalence of vaccine hesitancy; Oceania (N = 10), Europe (N = 82), Asia (N = 162), South America, (N = 7), Africa (N = 19), North America (N = 145) and Mix region (contain more than 1 region in the publication, N = 9; **S5 Table**). The overall estimate of pooled vaccine hesitant was 25.0% (95% CI: 0.22–0.27); I^2^ = 99.91%, p = 0.001). In Oceania, the overall pooled prevalence of vaccine hesitancy was 22.0% (95% CI: 0.11–0.33; I^2^ = 99.00%, p = 0.001), while it was 27.0% (95% CI: 0.22–0.32; I^2^ = 99.94%, p = 0.001) in Europe. In Asia, the overall pooled prevalence of vaccine hesitancy was 31.0% (95% CI: 0.27–0.34; I^2^ = 99.95%, p = 0.001), while in South America was 18.0% (95% CI: 0.08–0.27; I^2^ = 99.85%, p = 0.001). Meanwhile, in Africa, the overall pooled prevalence of vaccine hesitancy was 26.0% (95% CI: 0.16–0.36; I^2^ = 99.78%, p = 0.001), whereas in North America, the overall pooled prevalence of vaccine hesitancy was 25.0% (95% CI: 0.23–0.28; I^2^ = 99.87%, p = 0.001). Interestingly, the Mix region also shows the similar overall pooled prevalence of vaccine hesitancy with North America region, 25.0% (95% CI: 0.13–0.37; I^2^ = 99.85%, p = 0.001).

### The estimates of the pooled or overall prevalence of COVID-19 vaccine hesitancy among caregivers across the region

About 189 studies reported on the overall prevalence of COVID-19 vaccine hesitancy among caregivers: Asia (N = 81), Europe (N = 31), Mix (N = 6), North America (N = 63), Oceania (N = 3), Africa (N = 2) and South America (N = 3). The overall prevalence of COVID-19 vaccine hesitancy among caregivers was 29% (95% CI: 0.26–0.32; I^2^ = 99.91%, p = 0.001). The highest overall prevalence of COVID-19 vaccine hesitancy among caregivers was reported in the Africa region, 41% (95% CI: 0.13, 0.69; I^2^ = 99.03%, p = 0.001). South America has the lowest overall prevalence 16% (95% CI: 0.01–0.32; I^2^ = 99.95%, p = 0.001).

### The estimates of the pooled or overall prevalence of Influenza vaccine hesitancy among caregivers across the region

**Fig 1:**
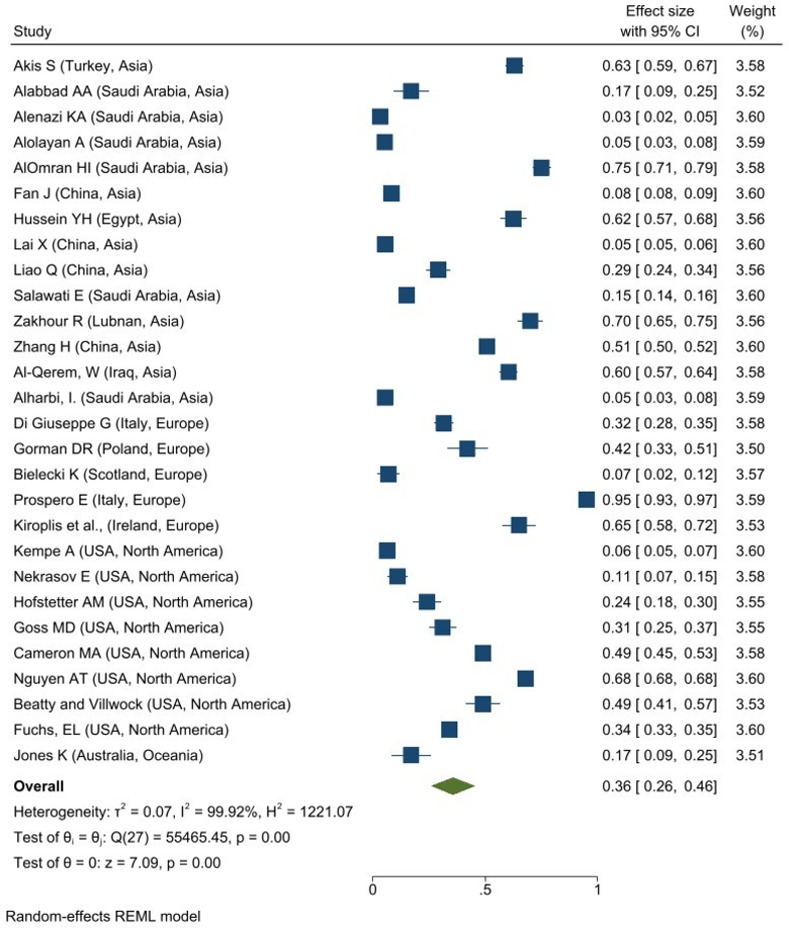
Forest plot of overall prevalence of influenza vaccine hesitancy among caregivers.

A total of 28 studies reported the overall prevalence of Influenza vaccine hesitancy among caregivers in Asia (N = 14), Europe (N = 5), North America (N = 8) and Oceania (N = 1). The overall prevalence of Influenza vaccine hesitancy among caregivers was 36% (95% CI: 0.26–0.46; I^2^ = 99.92%, P = 0.001; **Fig 1)**. The Europe region had the highest overall prevalence of Influenza vaccine hesitant among caregivers, 48% (95% CI: 0.19–0.78; I^2^ = 99.54%, p = 0.001) while the lowest overall prevalence is observed in North America, 34% (95% CI: 0.20–0.48; I^2^ = 99.85%, p = 0.001) and Asia 34% (95% CI: 0.19–0.48; I^2^ = 99.92%, p = 0.001).

### The estimates of the pooled or overall prevalence of HPV vaccine hesitancy among caregivers across the region

**Fig 2:**
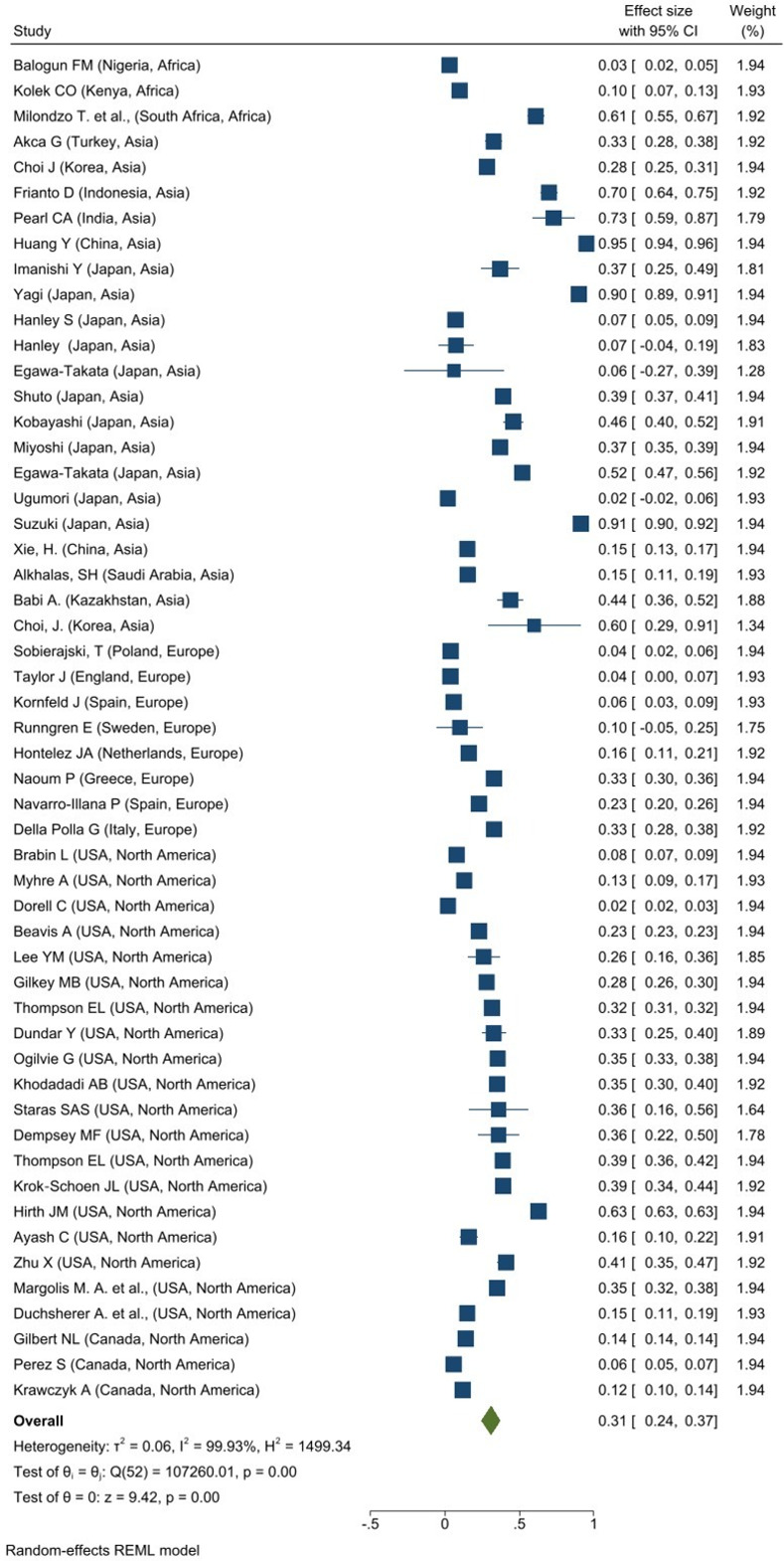
Forest plot of overall prevalence of HPV vaccine hesitancy among caregivers.

Meanwhile, 53 studies reported the prevalence of HPV vaccine hesitancy among caregivers: in Africa (N = 3), Asia (N = 20), Europe (N = 8), and North America (N = 22). As illustrated in **Fig 2**, the overall prevalence of HPV vaccine hesitancy among caregivers across regions was 31% (95% CI: 0.24–0.37; I^2^ = 99.93%, p = 0.001). The highest overall prevalence of HPV vaccine refusal among caregivers was reported in the Asia region with 43% (95% CI: 0.30–0.56; I^2^ = 99.82%, p = 0.001) while Europe had the lowest overall prevalence, 16% (95% CI: 0.07–0.25; I^2^ = 97.94%, p = 0.001).

### The estimates of the pooled or overall prevalence of MMR vaccine hesitancy among caregivers across the region

**Fig 3:**
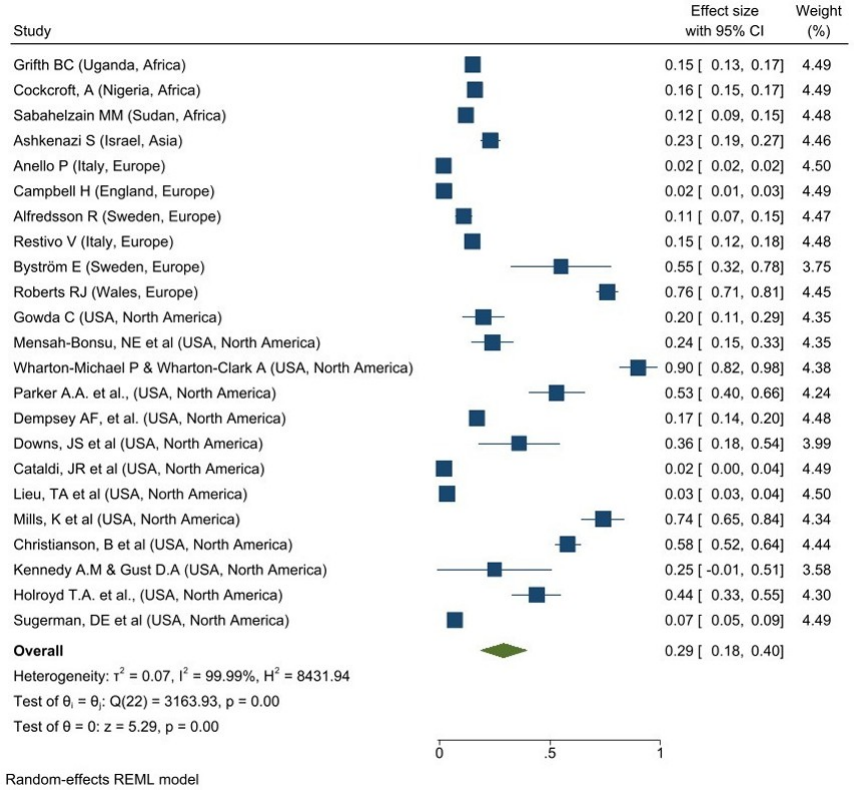
Forest plot of overall prevalence of MMR vaccine hesitancy among caregivers.

The prevalence of MMR vaccine hesitant among caregivers was reported in 23 studies: Africa (N = 3), Asia (N = 1), Europe (N = 6) and North America (N = 13). An overall prevalence of MMR vaccine hesitancy among caregivers was 29% (95% CI: 0.18–0.40; I^2^ = 99.99%, p = 0.001) as depicted in **Fig 3**. North America had the highest overall prevalence of MMR vaccine hesitancy among caregivers 35% (95% CI: 0.19–0.50; I^2^ = 99.76%, p = 0.001) while the lowest overall prevalence is reported in Africa, 15% (95% CI: 0.12–0.17; I^2^ = 69.65%, p = 0.001).

### The estimates of the pooled or overall prevalence of Mix vaccine hesitancy among caregivers across the region

A total of 76 studies reported the prevalence of Mix vaccine hesitancy among caregivers: Africa (N = 1), Asia (N = 28), Europe (N = 18), mixed region (N = 3), North America (N = 21), Oceania (N = 2) and South America (N = 3). An overall prevalence of Mix vaccine hesitancy among caregivers was 20% (95% CI: 0.16–0.24; I^2^ = 99.93%, p = 0.001). The highest overall prevalence of Mix vaccine hesitancy among caregivers was reported in Asia with 31% (95% CI: 0.20–0.41; I^2^ = 99.97%, p = 0.001) while Mix region reported the lowest overall prevalence 9% (95% CI: 0.03–0.16; I^2^ = 95.95%, p = 0.001).

### The estimates of the pooled or overall prevalence of other vaccine hesitancy among caregivers across the region

**Fig 4:**
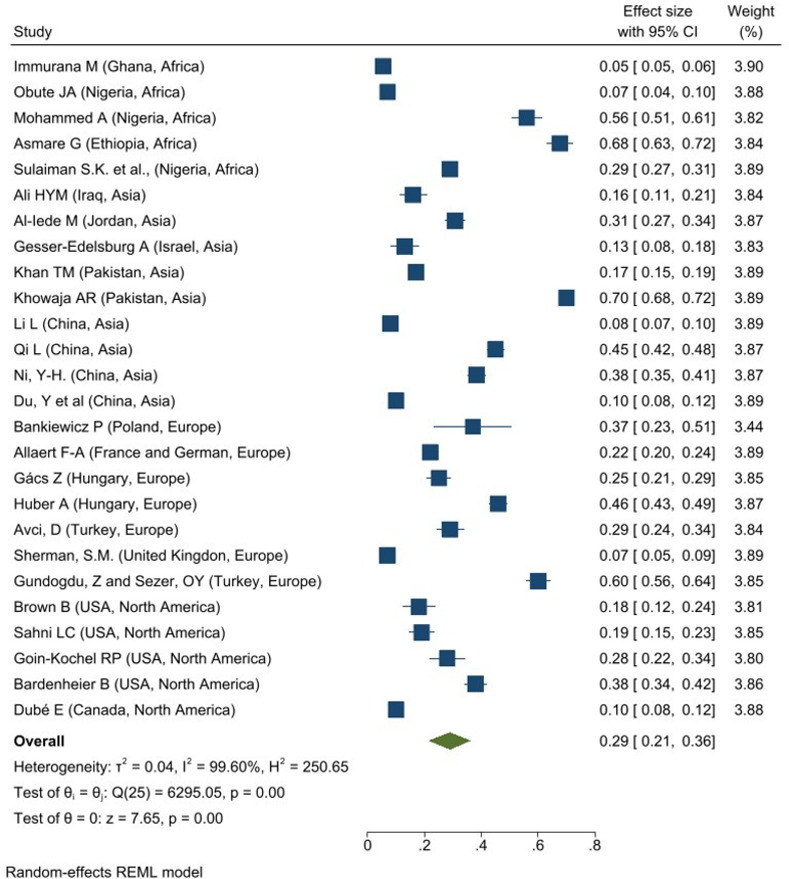
Forest plot of overall prevalence of other vaccine hesitancy among caregivers.

About 26 studies reported the prevalence of the Others vaccine hesitant among caregivers: Africa (N = 5), Asia (N = 9), Europe (N = 7) and North America (N = 5). An overall prevalence of the Others vaccine hesitancy among caregivers was 29% (95% CI: 0.21–0.36; I^2^ = 99.60%, p = 0.001). The highest overall prevalence of other vaccine hesitancy was reported in Africa with 33% (95% CI: 0.08–0.58; I^2^ = 99.87%, p = 0.001) as shows in **Fig 4**. North America reported the lowest overall prevalence, 23% (95% CI: 0.13–0.32; I^2^ = 96.47%, p = 0.001).

### The estimates of pooled or overall prevalence of Not stated vaccine hesitancy among caregivers across the region

**Fig 5:**
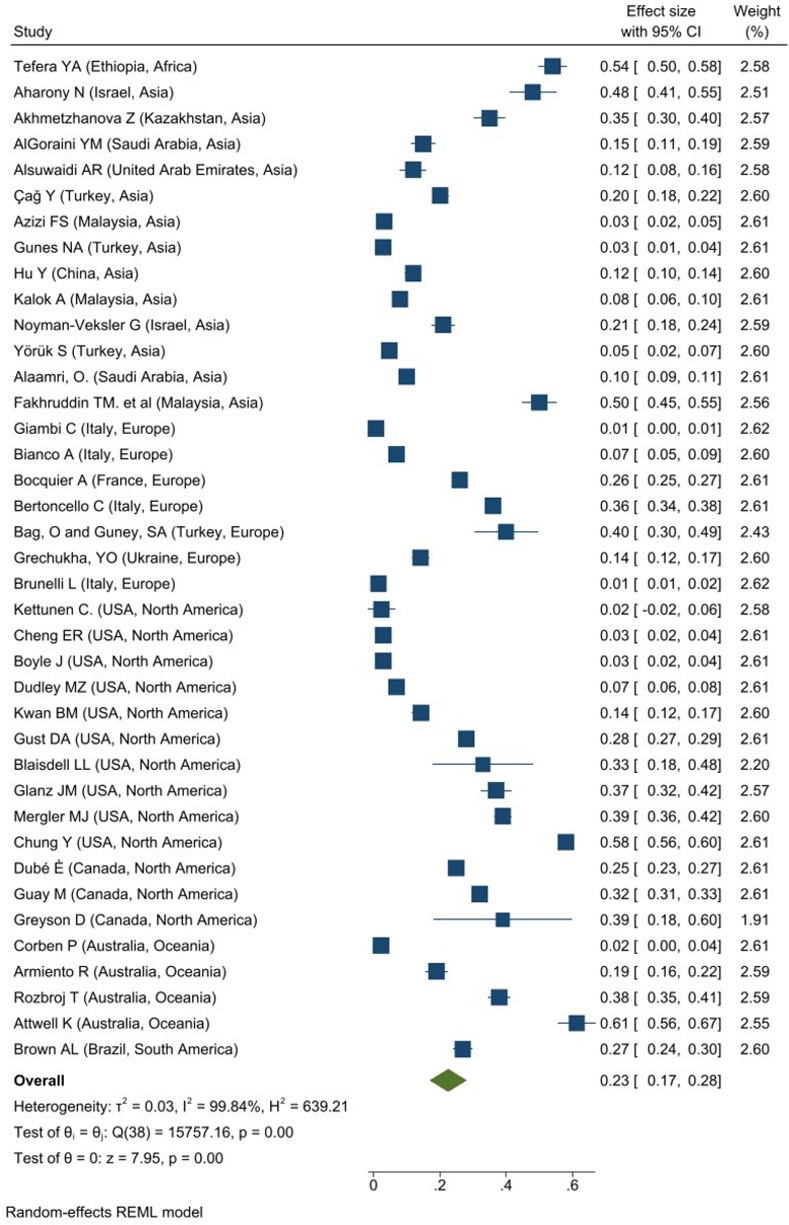
Forest plot of overall prevalence of Not Stated vaccine hesitancy among caregivers.

Meanwhile, 39 studies reported the overall prevalence of the Not stated vaccine hesitant among caregivers in Africa (N = 1), Asia (N = 13), Europe (N = 7), North America (N = 13), Oceania (N = 4) and South America (N = 1). The overall prevalence of the Not stated vaccine hesitancy among caregivers was 23% (95% CI: 0.21–0.36; I^2^ = 99.60%, p = 0.001) as shown in **Fig 5**. Africa region reported the highest overall prevalence of Not stated vaccine hesitant among caregivers with 33% (95% CI: 0.08–0.58; I^2^ = 99.87%, p = 0.001) and the lowest overall prevalence is reported in North America, 23% (95% CI: 0.13–0.32; I^2^ = 96.47%, p = 0.001).

The detail on the overall prevalence of the above vaccine hesitancy among caregivers listed in **S6 Table** while **S4 Fig** shows the forest plot results of the findings.

### The reasons for vaccine hesitant across the globe

In this review, all the reasons for caregiver vaccine hesitant were identified and categorized into five: (i) religious beliefs which includes the vaccination’s production proses, which involves using questionable sources such as pigs, cows and other animals that are prohibited in some religious; (ii) knowledge that drive the force for public panic and fear such as lack of awareness; (iii) a lack of information about vaccines that leads to misinformation such as believing false information posted on websites like blogs, Facebooks. Twitters, and others by individuals without any medical or health-related background and may be intended to mislead and deceive other; (iv) concern regarding safety and efficacy of vaccines, particularly after taking the vaccine regarding the presence of other diseases either moderate to severe such as cardiovascular disease, renal failure, skin rashes and others; and (v) other, more general reasons such as lack of concern for health, forgetfulness, difficulty obtaining transportation, distrust towards with the government, vaccine cost, young age and indecisive, timing issue as parents need to take leave for the vaccination, vaccine is not compulsory, unavailable vaccine near the medical facilities, location of medical facilities, bad experience with healthcare worker, misplaced or loss of the vaccination card, children having chronic medical condition and waiting for locally produced vaccine. In general, the most common reason of vaccine hesitancy around the worldwide is a lack of vaccine safety and efficiency (91.4%). About 88.1% is due to a lack of information, knowledge (77.9%), others (38.5%), and religious belief (36.2%). Regarding the COVID-19 vaccine, the most common reason for caregiver vaccine hesitancy around the world is also the lack of evidence on vaccine safety and efficiency (92.6%). A similar finding was observed for the Influenza (95.7%), MMR (90.0%), Mix (88.8%), and Others (93.0%) vaccine.

The detailed findings on the reason for vaccine hesitancy among caregivers according to regions: Australia, Europe, Asia, North America, South America, Africa, and Mix was depicted in **S7 Table.** Further analysis was also performed based on the type of vaccine applied in general and across the region.

## Discussion

Vaccine hesitancy among caregivers has been a worldwide issue for decades. However, there were not many reports on the pool or overall prevalence of vaccine hesitancy among caregivers across the regions. In this review, we pooled all prevalence of vaccine hesitancy across the regions, type of vaccine applied, and the reasons for vaccine hesitancy. Few vaccines including MMR, HPV, Influenza, COVID-19, mix, not stated, and others are listed in this review which reveals the findings for overall prevalence of caregiver vaccine hesitancy in the general vaccine is 25%. Whereas across the regions were between 14% - 30%. The South America region showed the lowest prevalence, 14% compared to other regions; 22% - 30%. This finding also showed that the prevalence of caregiver hesitancy is in the moderate prevalence and some steps are required to reduce the prevalence.

Asia had the highest prevalence of vaccine hesitancy; 41.0%, with knowledge and information being identified as contributing reasons. This finding is almost similar with the study in 2018. The poor or low awareness of HPV in this region can be attributed to the high prevalence of knowledge and information. Besides that, the classification of countries as high- or low-income countries [85] may lead to the findings since Europe region is observed with the lowest caregiver vaccine hesitancy against HPV. However, when it comes to influenza, Europe had the highest rate of caregiver vaccine hesitancy (48.0%) with the knowledge and information are listed as the contributing factors. Similar finding was reported in other study and suggested that although the region had supported the influenza vaccine as a part of a national vaccination program, it could be challenging to harmonies vaccine policies in this region due to the difference in background and culture [86].

The MMR vaccination rate was observed higher in North America (35.0%) and vaccine safety and efficacy factors contribute to the caregiver vaccine hesitancy. A similar finding was reported in 2023 and included the possibility that a caregiver’s high income and level of education might influence their decision to vaccinate their children [87]. The category of mixed vaccination was reported higher in Asia (31.0%), and vaccine hesitancy is influence by both vaccine safety and efficacy. A study in China reported that caregivers’ vaccine hesitancy was significantly associated with the number of children in the family, children health status, caregiver education level, and family annual income [88]. Whereas for the other vaccine category, there is a small difference in prevalence of caregiver vaccine hesitancy across the region, between 23.0% - 33.0% with information and vaccine safety and efficiency contribute to the vaccine hesitancy. A review study on Hepatitis vaccination suggested that the age of caregiver plays a role for the vaccine acceptance, with caregiver between age of 27–38 years having a higher chance of accepting the Malaria vaccine than caregiver over 50 years old [89].

The COVID-19 vaccine is the most recent vaccine type covered in this analysis with range of 29% globally and 16% to 32% across the regions. A study in the USA reported that children represent 14.4% of total COVID-19 cases since it hit pandemically in March 2020 and the prevalence rose to 18.0% in August 2021 [90]. Children with COVID-19 make up 2.4% of total hospitalizations in The United States America (USA) and about 1% of all paediatric COVID-19 cases resulted in hospitalization since the beginning of the pandemic [91]. This caused the idea of urgent COVID-19 vaccine development in 2020 and raised concerns about vaccine hesitancy among caregivers due to the limited timeframe required to produce the vaccine.

Lack of knowledge, safety, religious beliefs, vaccine information, and others such as forgetfulness, financial burden, the expense cost of the vaccine production, and children’s decision to receive the vaccine are listed as factors contributing to vaccine hesitation among caregivers across the regions. Besides that, the vaccine that required a few cycles of injections [92] and the expensive price [91] were also named as a factor that contributed to the caregiver vaccine hesitancy. Quinn et al 2016 also reported the similar finding [92]. On a separate note, religious belief is generally linked to the core beliefs of caregivers or guardians for example vaccines made from the mixture of substance from unclean derivates, derivatives from pigs. This is because majority of Islamic jurist believe that any component of pig product is unclean and forbidden to be consumed by Muslim community [93] or in short, they are concerned about the vaccine’s ‘*halal*’ status. Furthermore, 80% of the crucial substance or ingredients used in the vaccine production; gelatin and trypsin, are made from pig hide extract and the remaining 5% come from the pig’s bone [94]. Some of trypsin is produced from pig pancreas [95]. Overall, among the five listed reasons mentioned, the main reasons for caregiver vaccine hesitancy are vaccine safety and efficacy for almost all types of vaccines listed in this review, either globally or across the regions (>90%). However, certain vaccines and regions showed different findings. For example, the HPV vaccine showed the highest percentage of information gaps globally and in Europe. Similar findings were observed in mixed vaccines category for Africa, South America, and Europe. As stated previously, the different prevalence of vaccine refusal across the populations might be due to the vast differences in the health system, caregivers’ educational background, and family income [2]. The highest prevalence of the main reason of vaccination hesitancy is similar with other finding reported about Malaria vaccine. The Africa area had the highest prevalence of Malaria infections and deaths (94%) [96] which may have an impact on caregiver willingness to accept Malaria vaccine. However, depending on the number of Malaria cases in the area, the prevalence of Malaria vaccine hesitancy might differ across the region. According to WHO statistics, the European region has been Malaria-free since 2015, but Southeast Asia region has 33% prevalence of Malaria [96]. In contrast, less than 8% of caregivers were hesitant to accept the malaria vaccine for their child when it is available [97]. On the other hand, study conducted by Biezen et al. reported that caregiver attitudes toward vaccine safety and efficacy are the most important factors that influence decisions in the uptake of vaccination [2].

To overcome caregiver vaccine hesitancy and decrease the prevalence of vaccine hesitant, healthcare providers must be able to understand caregivers’ primary concerns about their children’s vaccinations for example providing more data, information, and details about the vaccine including the vaccine production, potential side effect and the importance of vaccination for their children [98]. They must also be able to give the caregivers with the information and knowledge that are needed to make the best decisions for their children. This will assist caregivers who are hesitant to vaccinate their children, as their main concern is their children’s safety. Furthermore, healthcare providers must have open and honest conversations with caregivers. It can help them to understand the benefits of vaccination without being judged, criticized, or attacked for the concerns about their child’s health [98–100]. Although it has been established that caregivers who are more educated and informative have a better attitude and acceptance of their children’s vaccinations, the exact or precise messages and techniques that healthcare providers should use to educate caregivers have not been fully defined [101].

Additionally, it has been proven that children free vaccination programs, which are delivered as a nationwide or routine program in the certain countries, minimize vaccine hesitant among caregivers [102]. Messages emphasizing the personal health risks and collective health consequences of not vaccinating. According to Motta et al, it will dramatically enhance a person’s intentions to vaccinate [103]. Interestingly, although most of the countries in North America regions showed mandatory childhood vaccination, there is still a high prevalence of caregiver vaccine hesitancy (>20%) [104]. An interactive, tailored, and targeted educational strategies might help to increase the vaccination rates. Having a caregivers education website, for example, may aid in the creation of appropriate and effective immunization information for individuals [103]. Caregivers’ attitudes, perceptions of their peers, sense of self-efficacy, desire to vaccinate, and vaccination behavior can be influenced by a well-designed educational intervention [105]. Simple and effective pamphlets can also be used to consult caregivers to ensure that content is comprehensible and understandable to them, which can be an essential step in combatting anti-vaccination information on the internet [106].

The importance of social media and public trust in the government may play a role in encouraging immunization and preventing diseases. Furthermore, to prevent erroneous information from spreading on social media, health authorities must provide scientific data and adequate information about vaccine benefits and risks, such as the COVID-19 vaccine [107]. Moreover, it will lead to the development of public trust via transparent, clear, and timely information based on scientific knowledge. As a result, it will increase caregivers’ or guardians’ willingness to vaccinate their child or children [107]. Another strategy to boost vaccine uptake is to verify caregivers’ correct and valid addresses or phone numbers at each visit to minimize missed vaccination appointments [108]. Thus, using a variety of reminder mechanisms, such as physical mail and mobile text reminders, could help enhance vaccine uptake [109]. Therefore, a few steps are suggested to overcome the caregiver hesitant towards vaccines: (i) intervention for caregivers such as children outreach or home visits, (ii) interventions for providers for example having nurses or other medical staff members take a more active role in the immunization process and (iii) interventions for clinics and communities such as providing vaccinations free of charge [110]. Other intervention such as nudging approaches can be applied to overcome this issue as it has been shown to increase vaccine confidence and improve low- and middle-income countries as it changes the vaccination attitudes and behaviour [111]. Other incentive including providing the financial aids in the form of gift cards, have been observed to elevate the vaccination rates by 2.64 to 4.23% among children between the ages of 12- to 17-year-olds [112].

Despite a comprehensive literature search, there are several limitations in our review. First, we only included studies that were published in English, meaning that only work publications written in English were considered. Due to the scarcity of studies from non-English-speaking nations, we may have missed studies in other languages. The statistical heterogeneity likely to be high due probably to heterogeneity in research study designs and populations [113]. To account for this heterogeneity, the random effects model was applied in this review [113]. We also categorise the studies according to the regions and the vaccine taken to overcome this issue. Besides that, we are unable to draw the conclusion about the causal linkages between caregivers’ acceptance to vaccinate their children against any vaccines and predictors of this altitude since most of the included studies were cross-sectional. We noted that most of the included studies were self-administered by the respondent using online or physical forms. This might increase the possibility that the respondents did not answer or misunderstood the questions properly and provided inaccurate answers.

## Conclusions

This review showed that the overall pooled prevalence of vaccine hesitancy among caregivers across the regions is 25% with vaccine safety and efficiency are identified as a major factor contribute to vaccine hesitancy among caregivers. Finding of this review also provides essential information for future vaccination programs that can be implemented across the region to reduce the prevalence of vaccine hesitancy among caregivers. Therefore, future research is needed to determine the best strategies to improve understanding and the importance of vaccine among caregiver mainly in the vaccine safety and efficiency.

## Supporting information

S1 Fig PRISMA flow diagram on selection process. (PDF)

S2 Fig Included papers reported on the reason of the parental vaccine hesitancy across the region in the review. (PDF)

S3 Fig Included paper stated the prevalence of parental vaccine hesitancy across the region. (PDF)

S4 Fig Forest plot overall prevalence of vaccine hesitancy. (PDF)

S1 Table Key search strategy vaccine hesitancy. (PDF)

S2 Table Search strategy vaccine hesitancy using CoCoPop. (PDF)

S3 Table Data collection form. (PDF)

S4 Table Quality assessment for included study. (PDF)

S5 Table Prevalence of vaccine hesitancy among parents across the region. (PDF)

S6 Table Overall prevalence of the vaccine hesitancy across the regions. (PDF)

S7 Table Reasons for the parental hesitancy towards vaccine. (PDF)

S1 Appendix List of the included study. (PDF)

S2 Appendix PRISMA 2020 checklist. (PDF)
