# Supplementary material for: Prevalence of caregiver hesitancy for vaccinations in children and its associated factors: A systematic review and meta-analysis
Source: PLoS One. 2024 Oct 24;19(10):e0302379. doi: 10.1371/journal.pone.0302379 (PMC11500859; doi:10.1371/journal.pone.0302379)

S3 Figure: Included paper stated the prevalence of parental vaccine hesitancy across the region

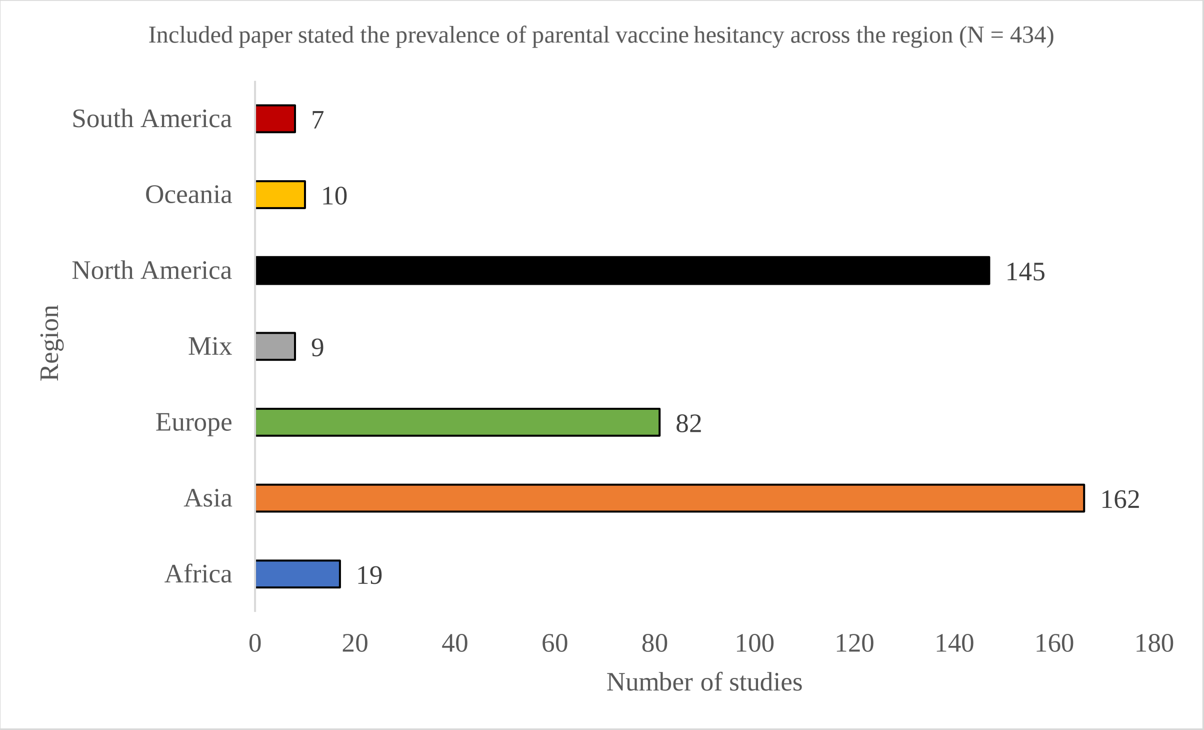

Supplement: S3 Fig — (PDF) [file pone.0302379.s003.pdf]
