# Supplementary material for: Prevalence of caregiver hesitancy for vaccinations in children and its associated factors: A systematic review and meta-analysis
Source: PLoS One. 2024 Oct 24;19(10):e0302379. doi: 10.1371/journal.pone.0302379 (PMC11500859; doi:10.1371/journal.pone.0302379)
Supplement: S4 Fig — (PDF) [file pone.0302379.s004.pdf]

S4 Figure: Forest plot overall prevalence of vaccine hesitancy

(1) <u>All vaccine</u>

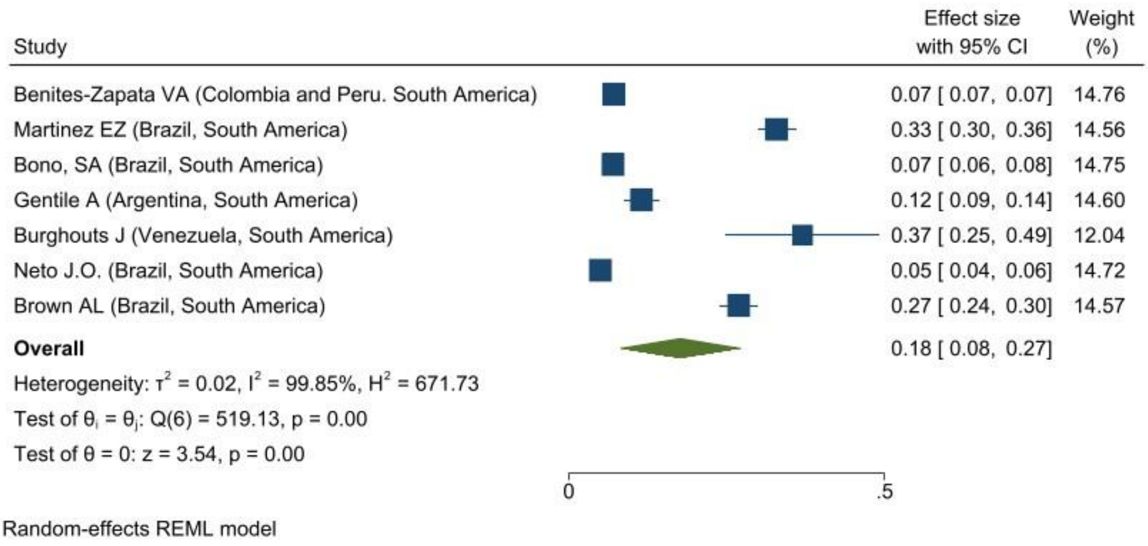

i. *South America region*

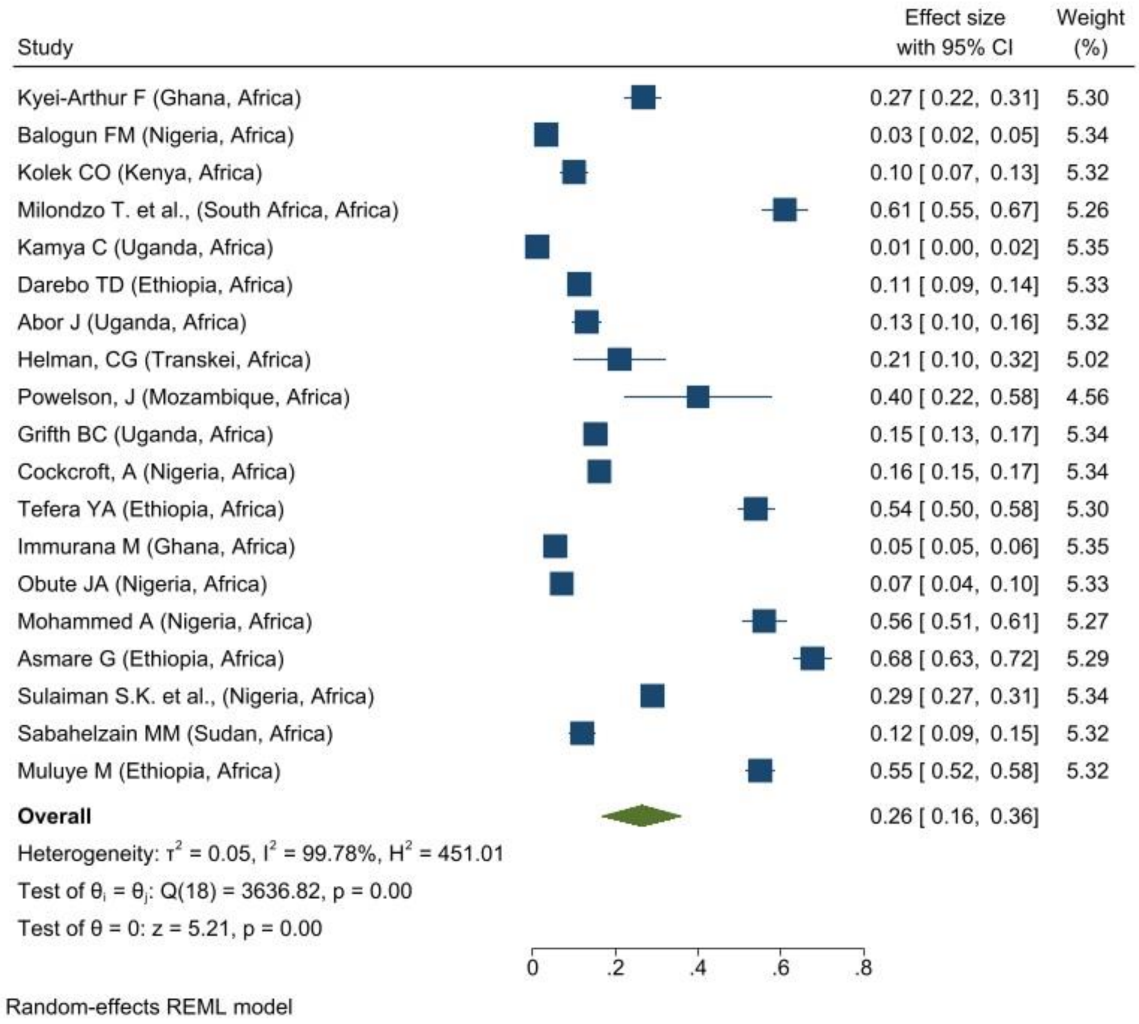

ii. *Africa region*

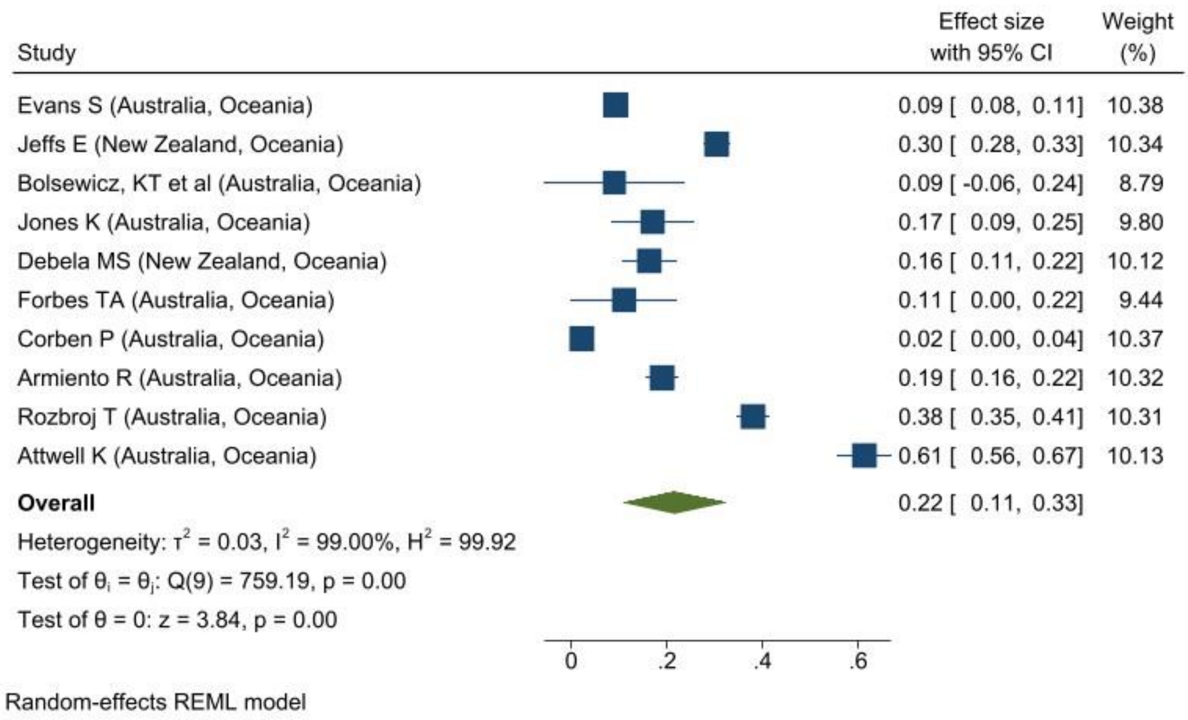

iii. Oceania region

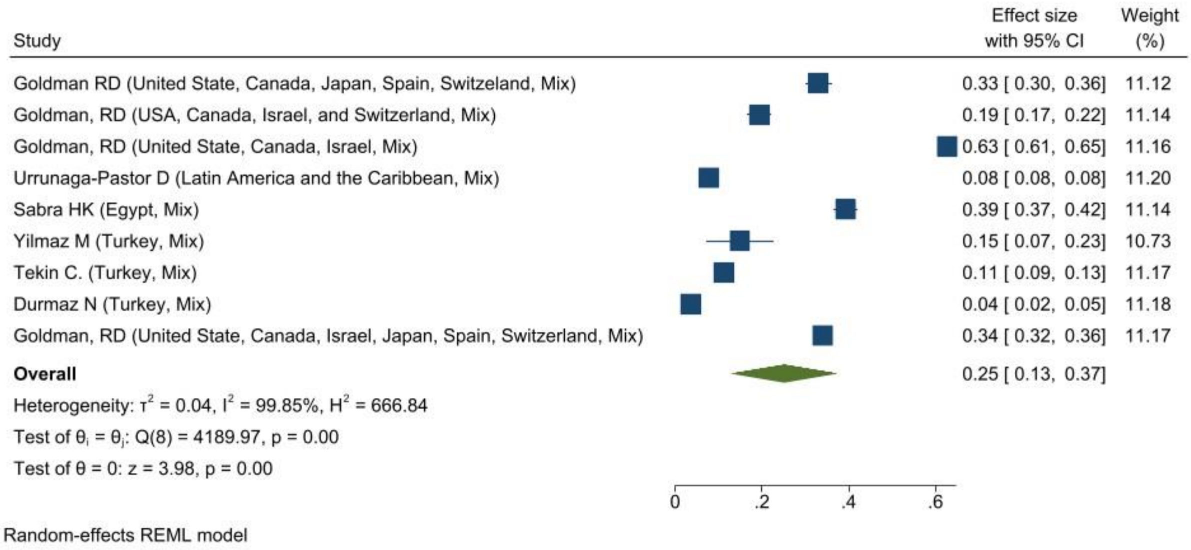

v. Mix region

- Forest plot for all regions, Asia, North America and Europe are unable to be generated due to the total of articles (high number of articles) included for all type of vaccines.

(2) Covid-19 vaccine

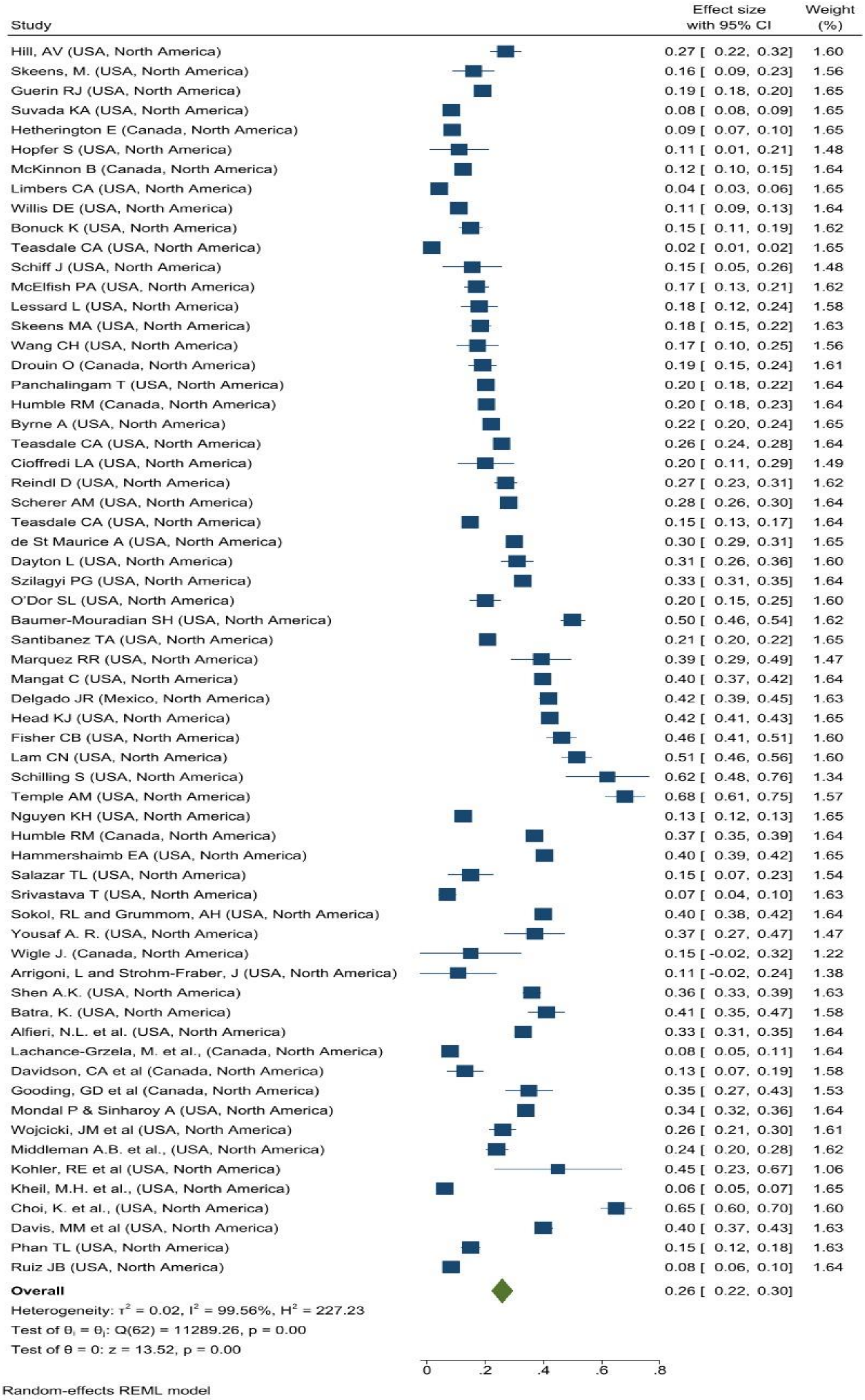

i. North America region

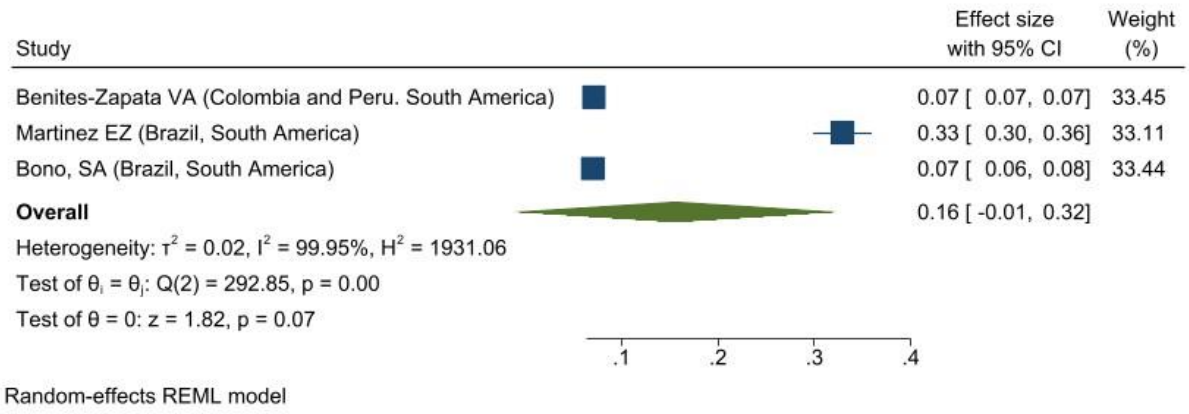

ii. South America region

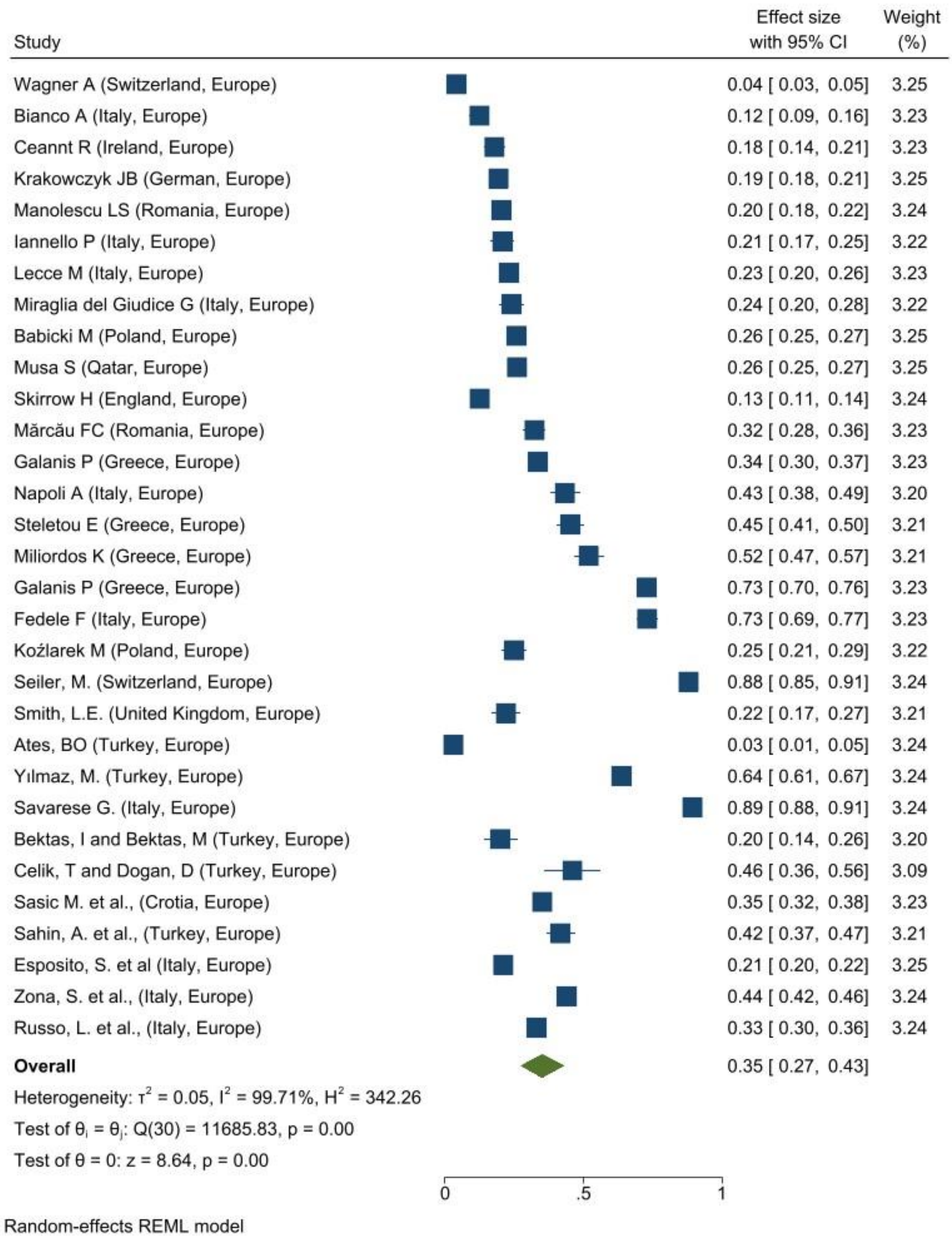

iii. Europe region

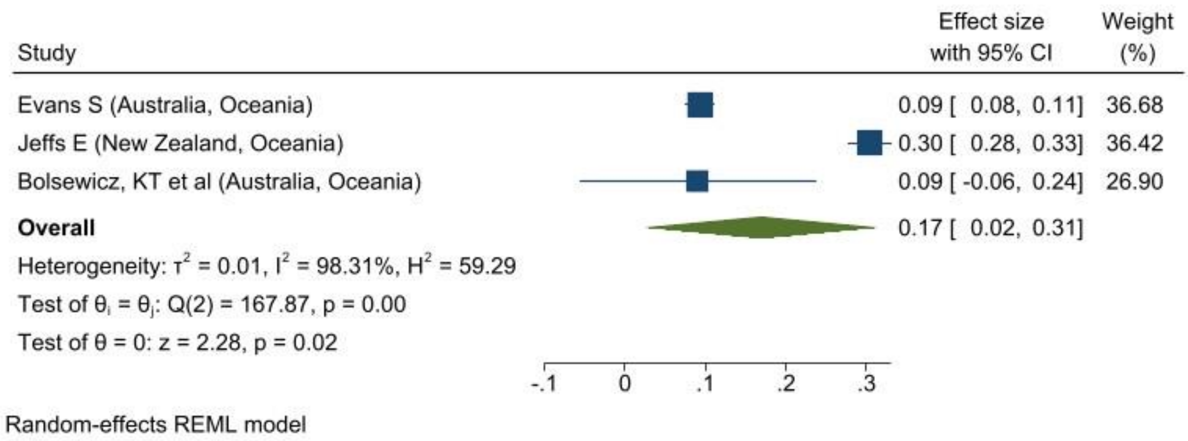

iv. Oceania region

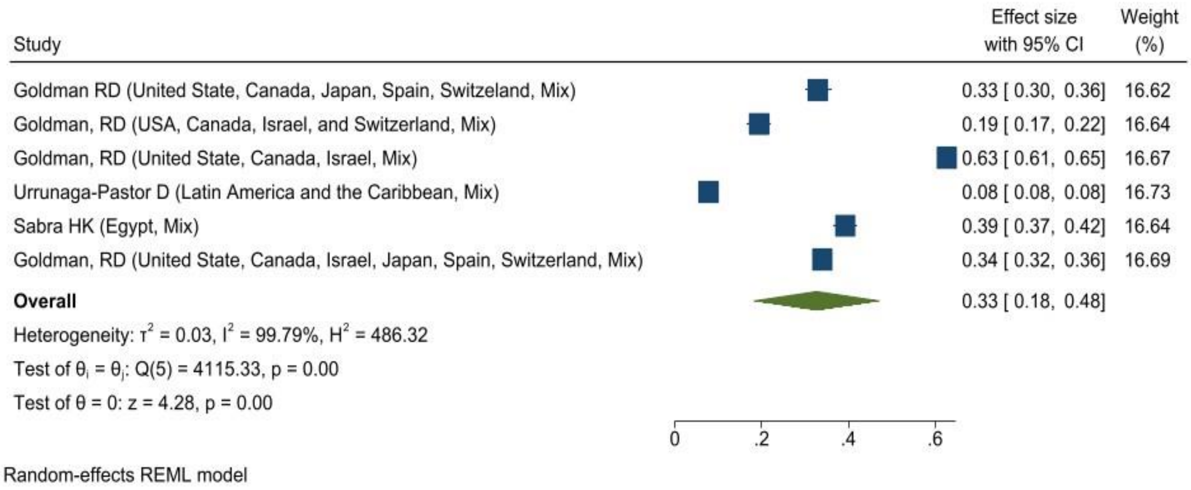

v. Mix region

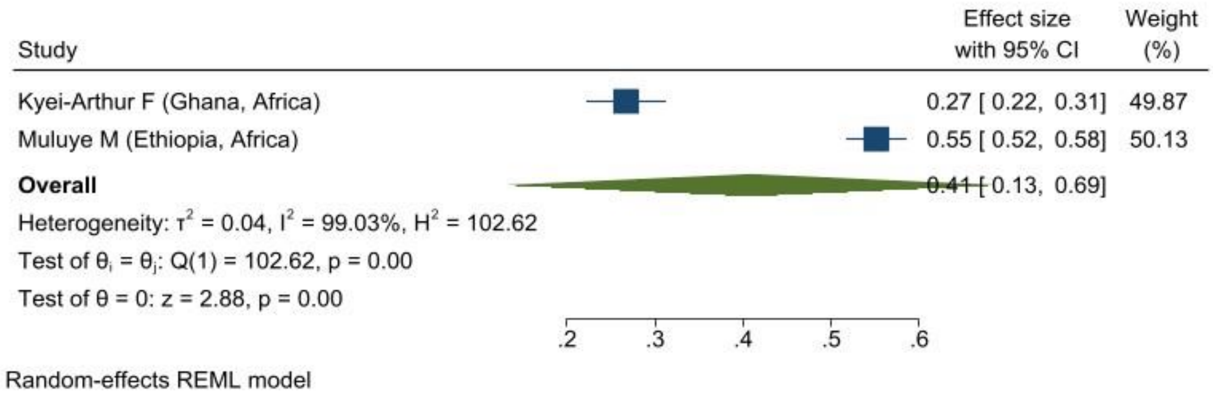

vi. Africa region

- Forest plot for all regions and Asia are unable to be generated due to the total of articles (high number of articles) included for COVID-19 vaccine.

(3) HPV vaccine

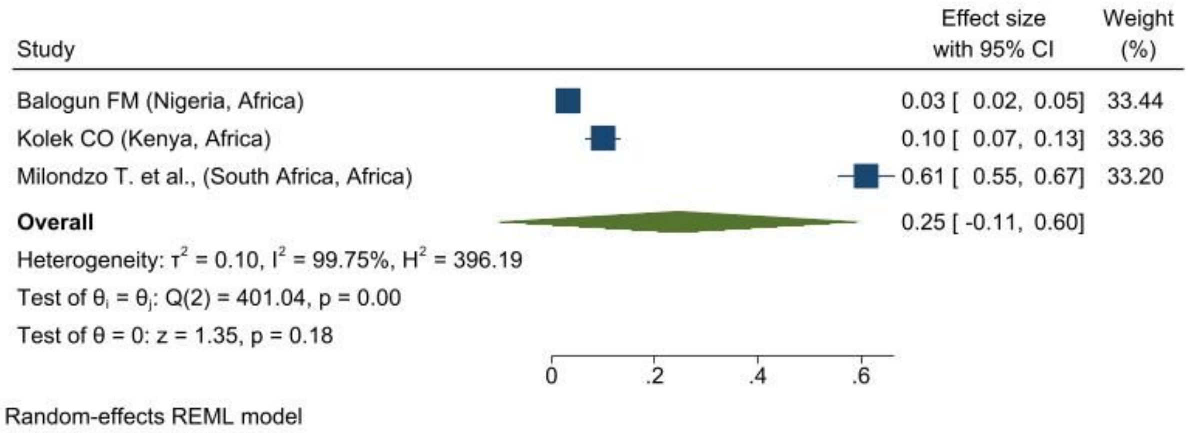

i. Africa region

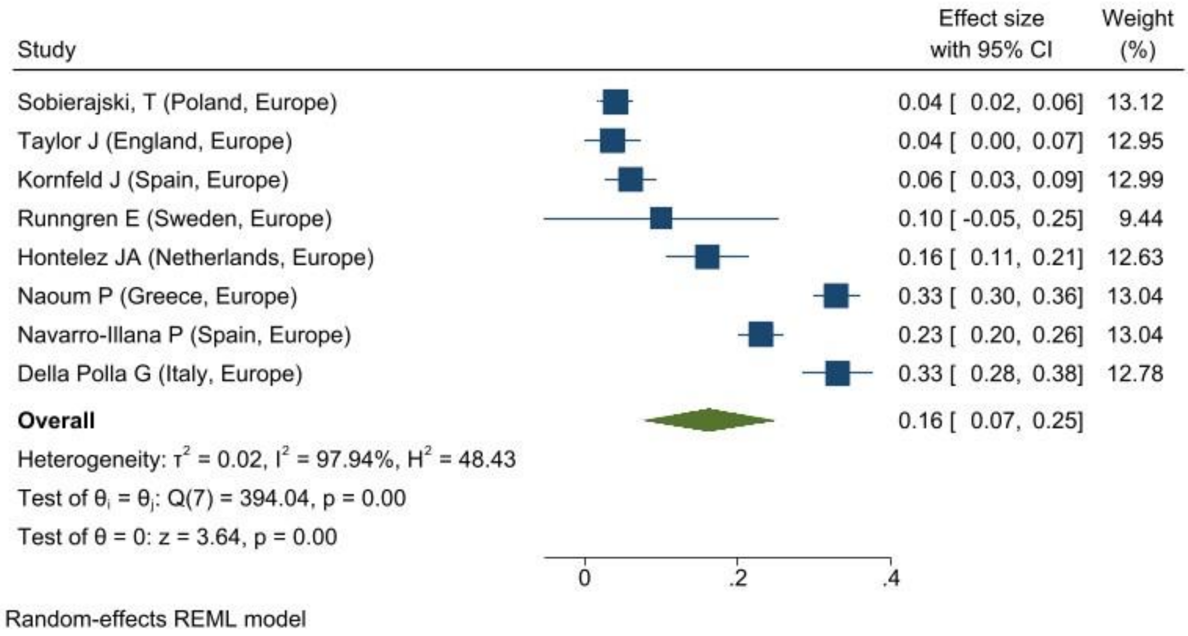

ii. Europe region

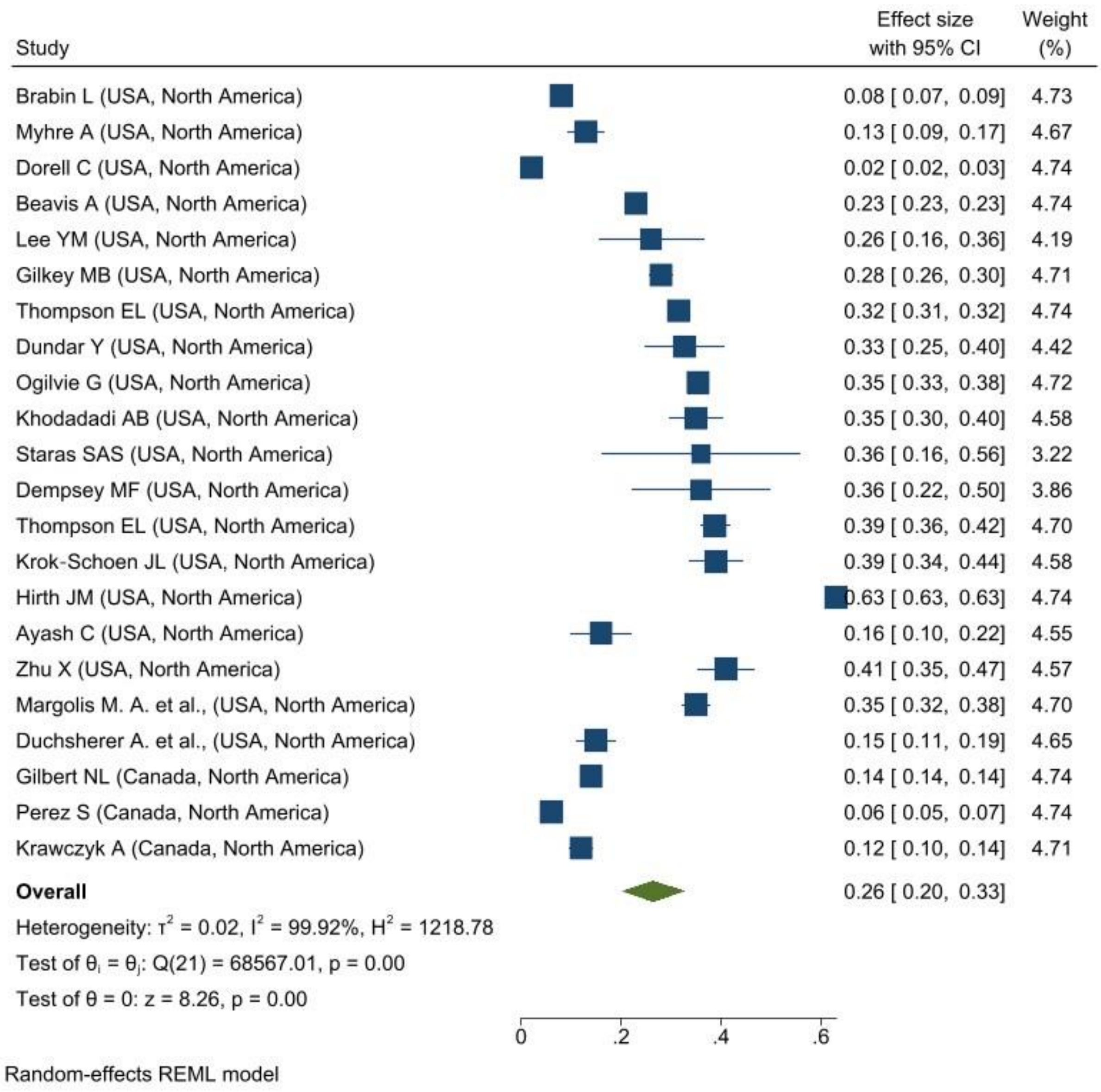

iii. North America region

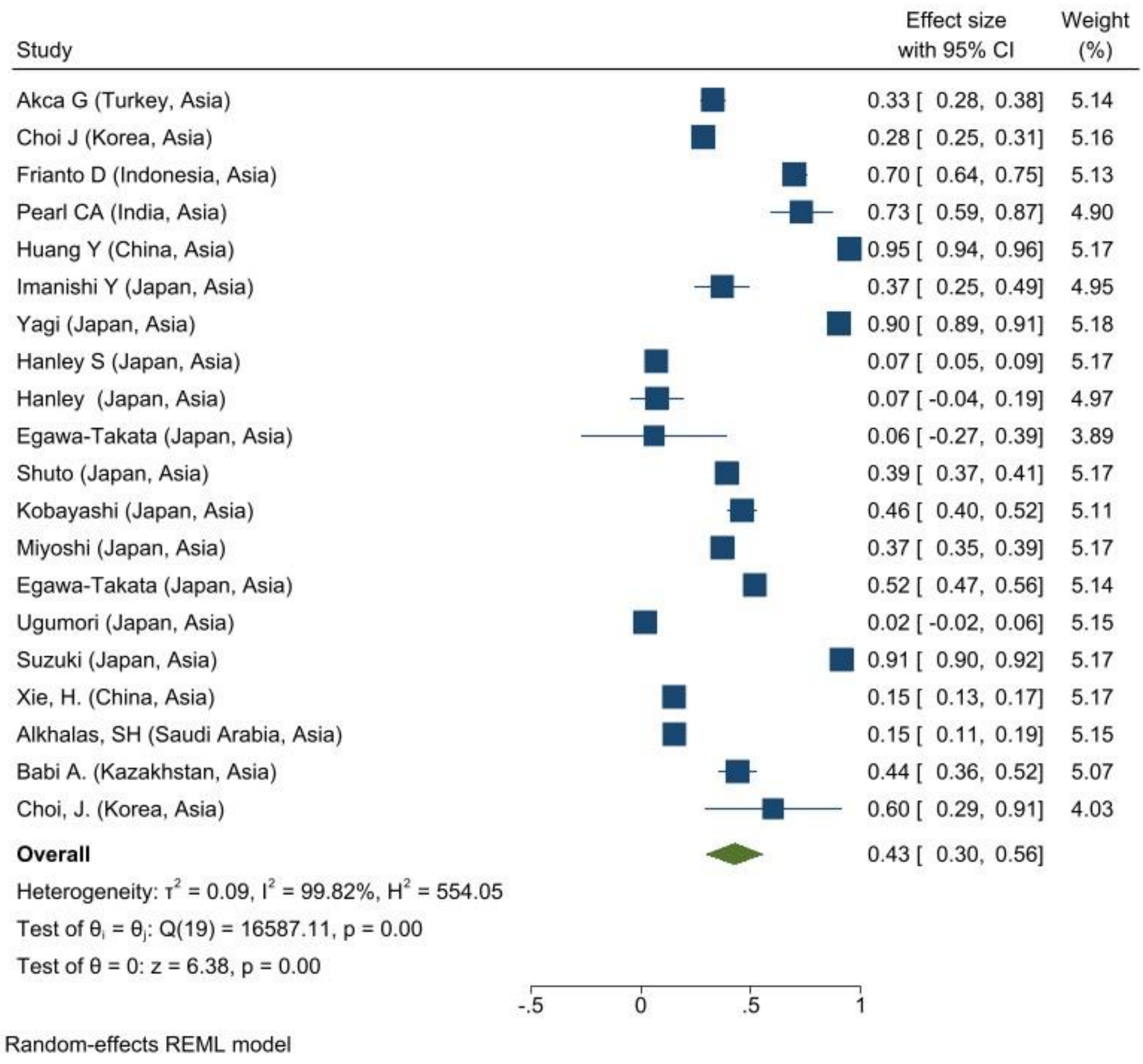

iv. Asia region

4) Influenza vaccine

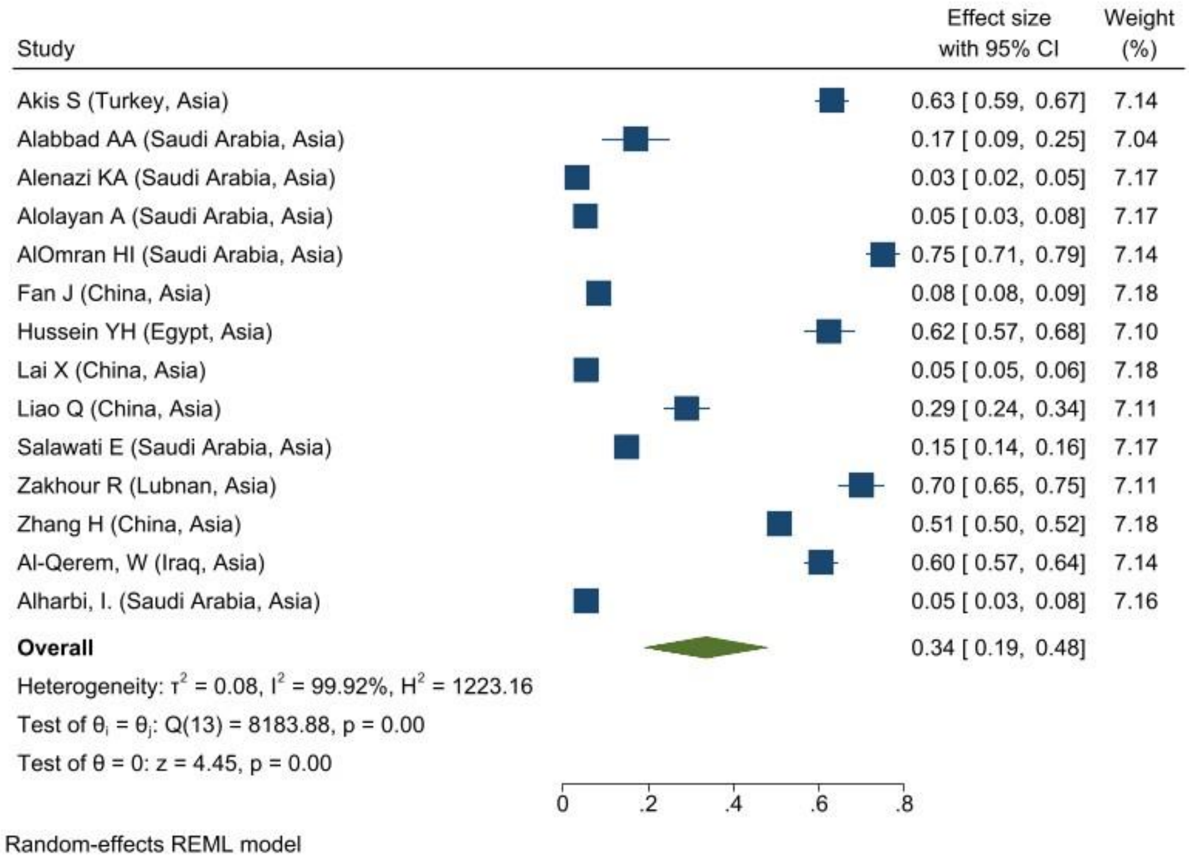

i. Asia region

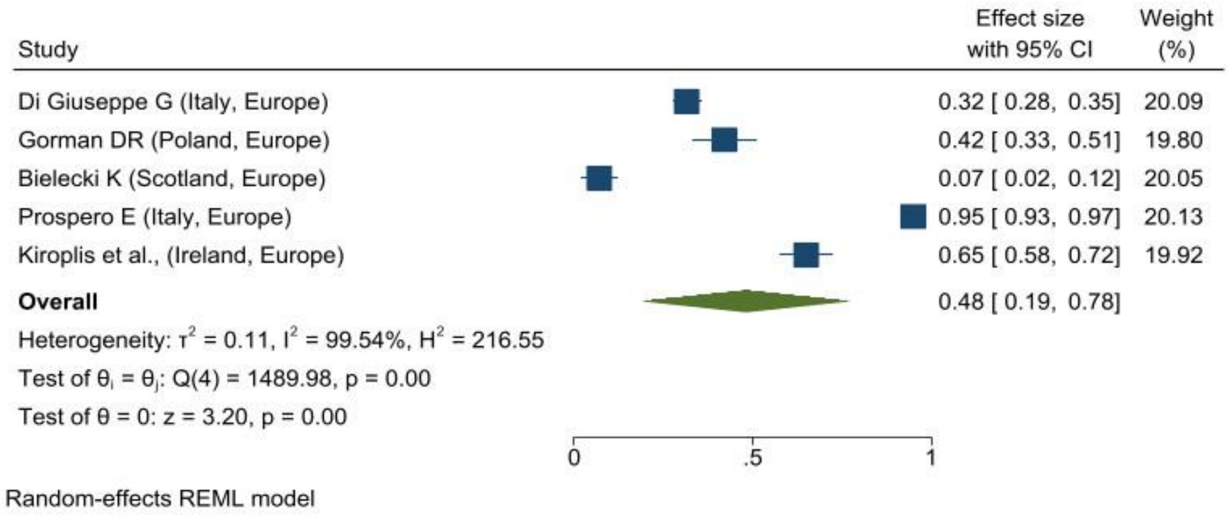

ii. Europe region

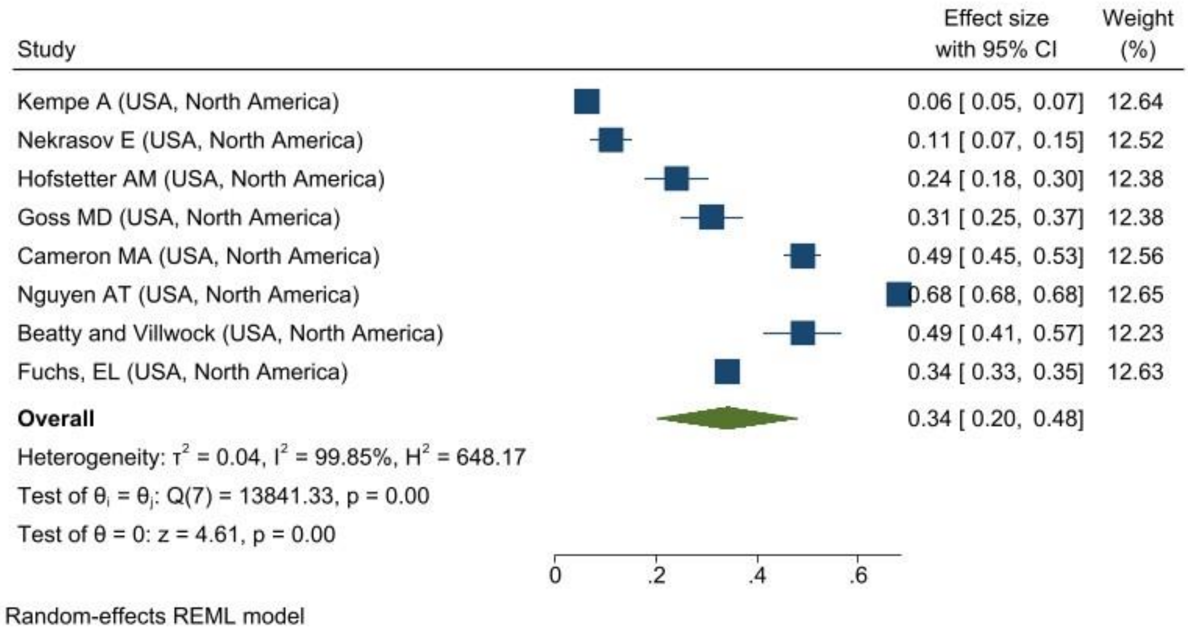

iii. North America

5) Mix vaccine

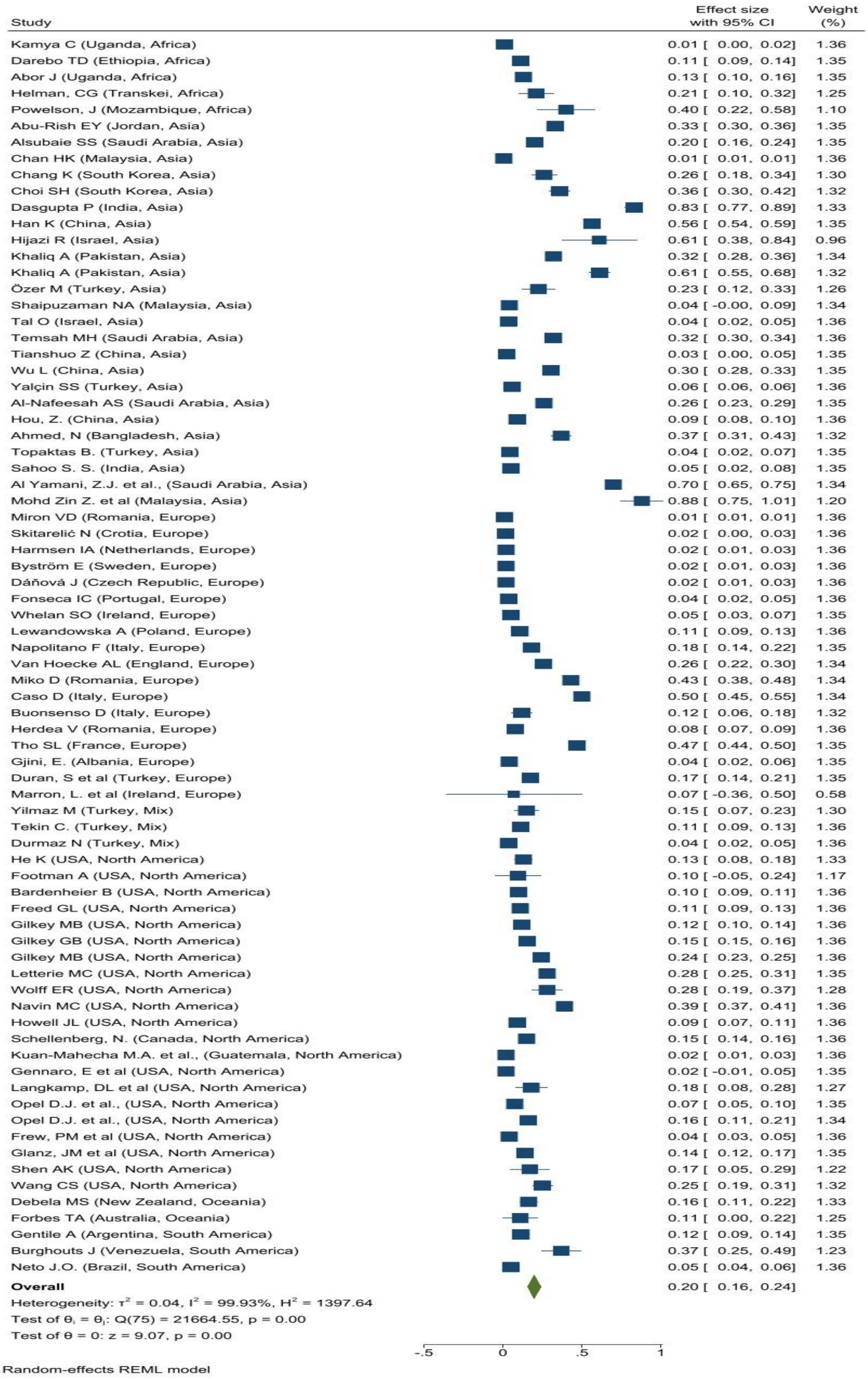

i. All region

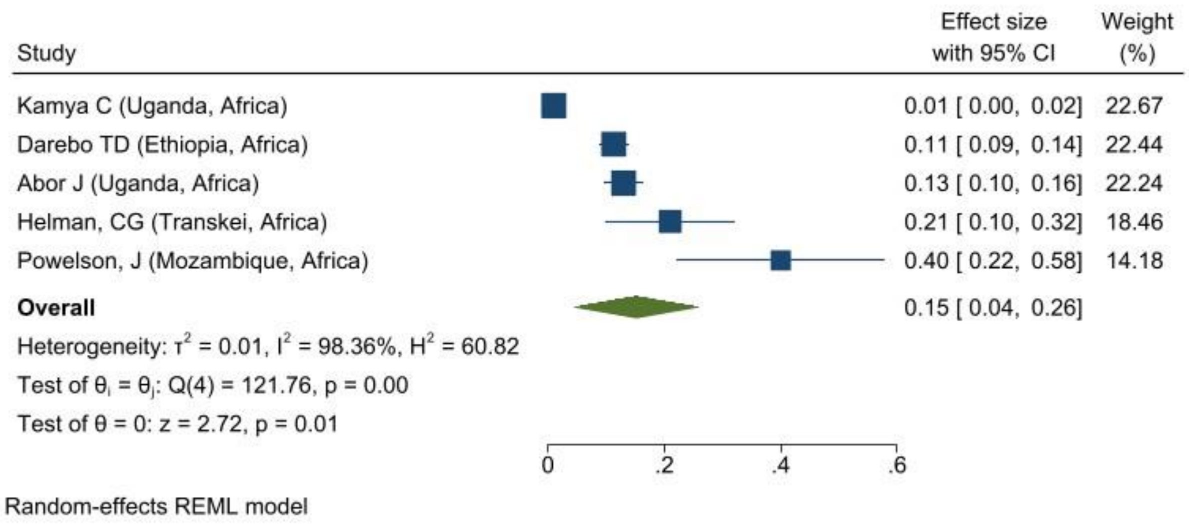

ii. *Africa region*

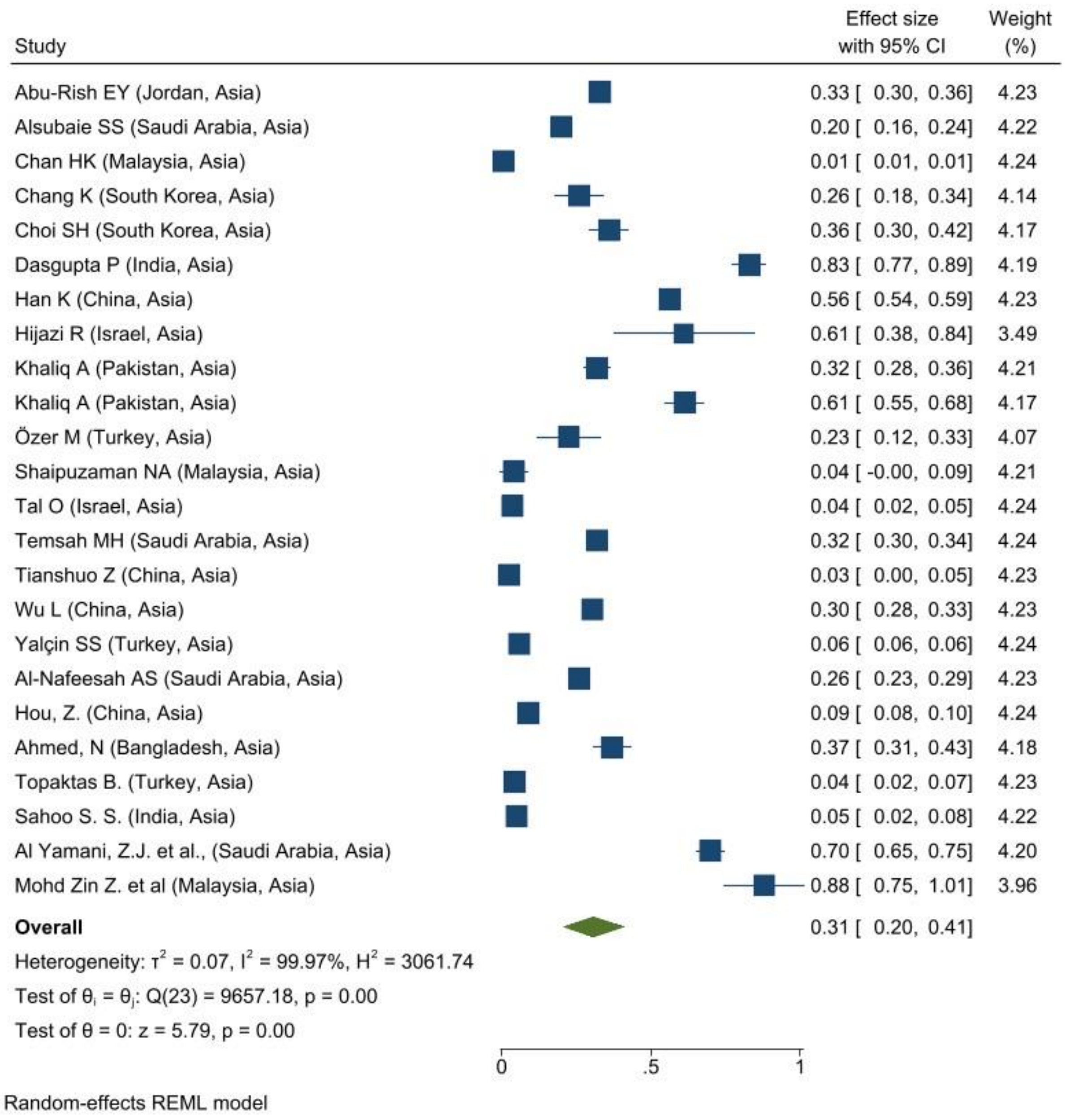

iii. Asia region

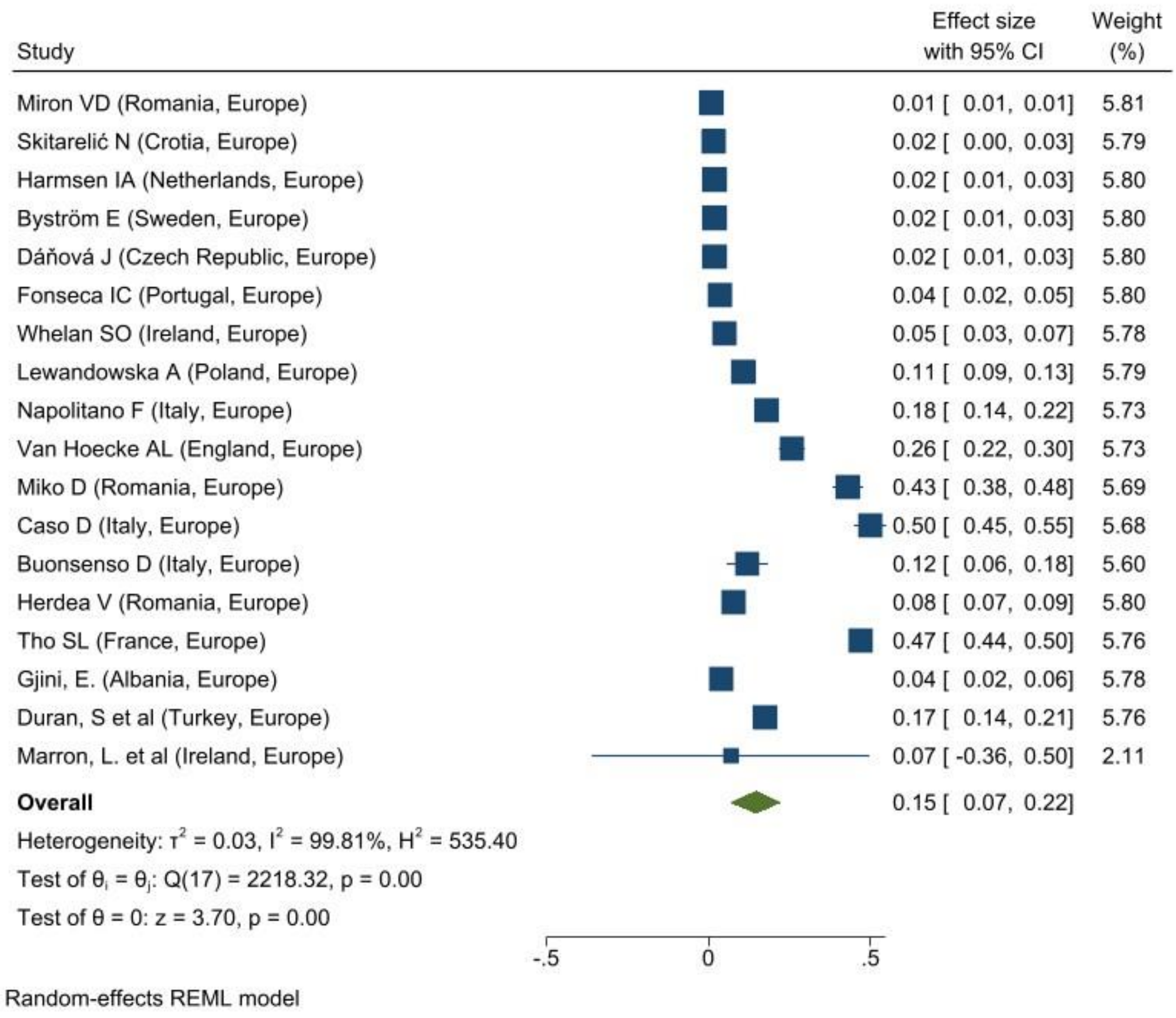

iv. Europe region

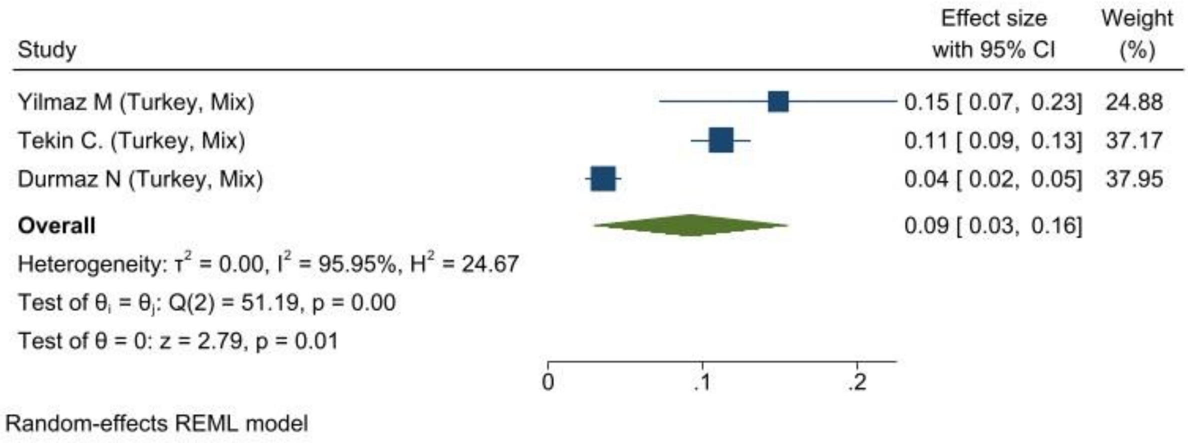

v. Mix region

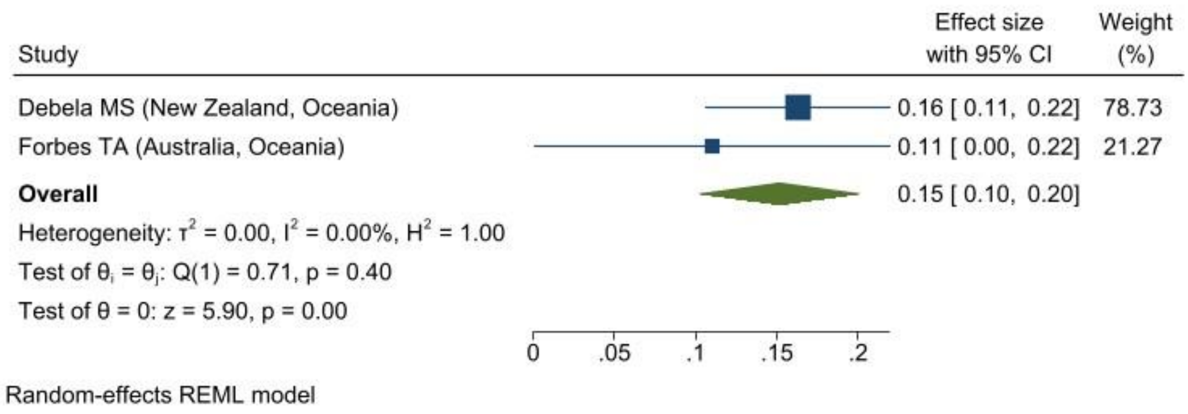

vi. Oceania region

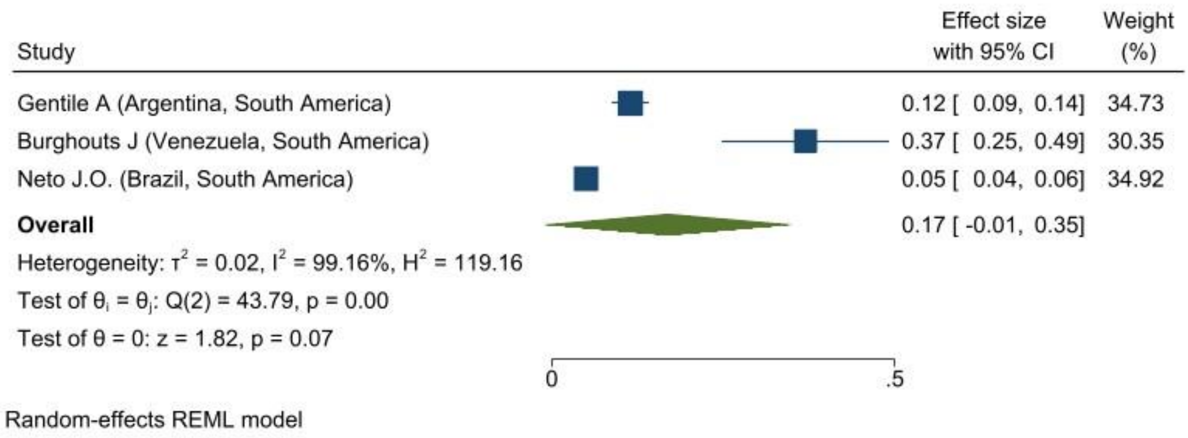

vii. South America region

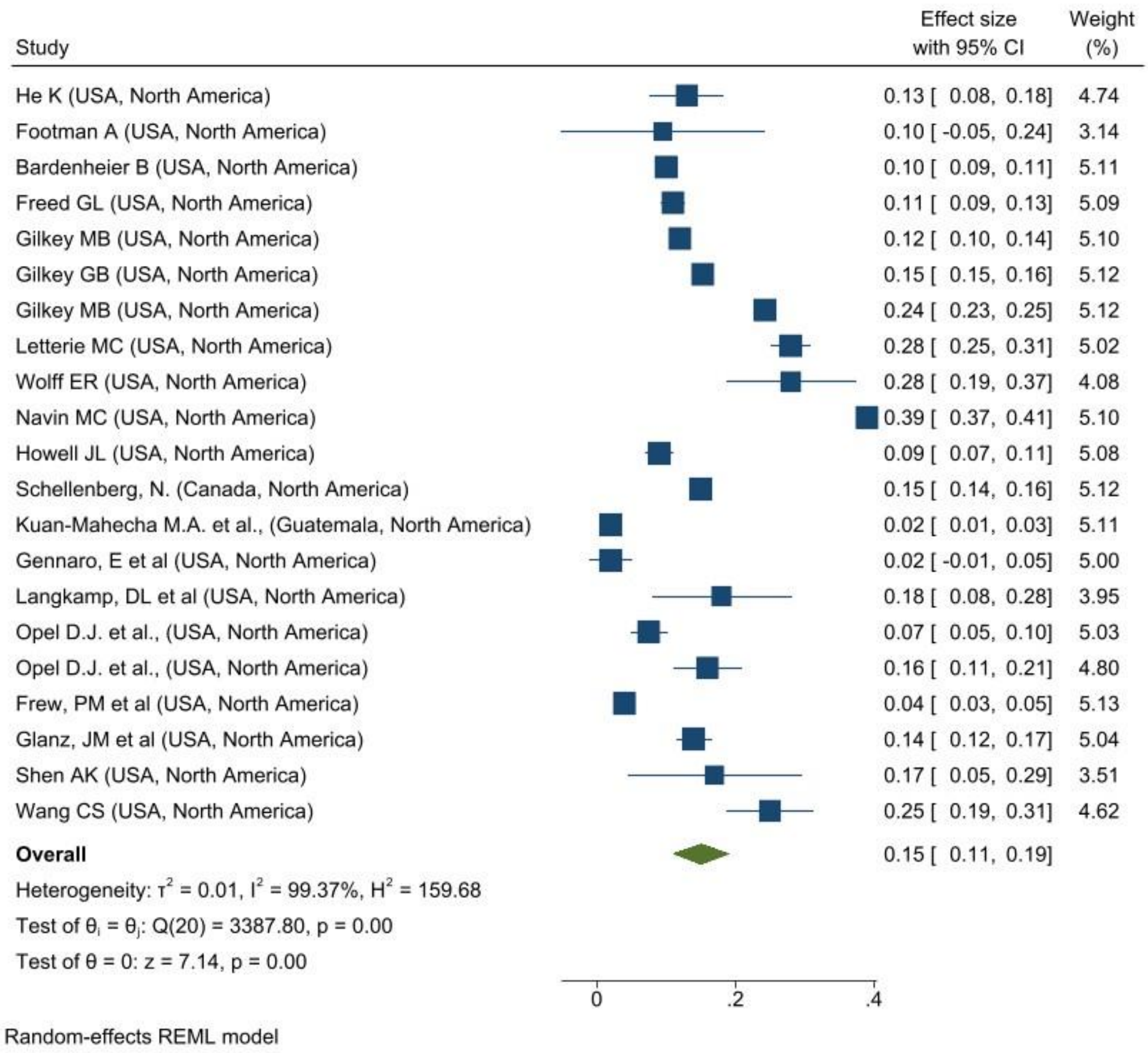

viii. North America region

6) MMR vaccine

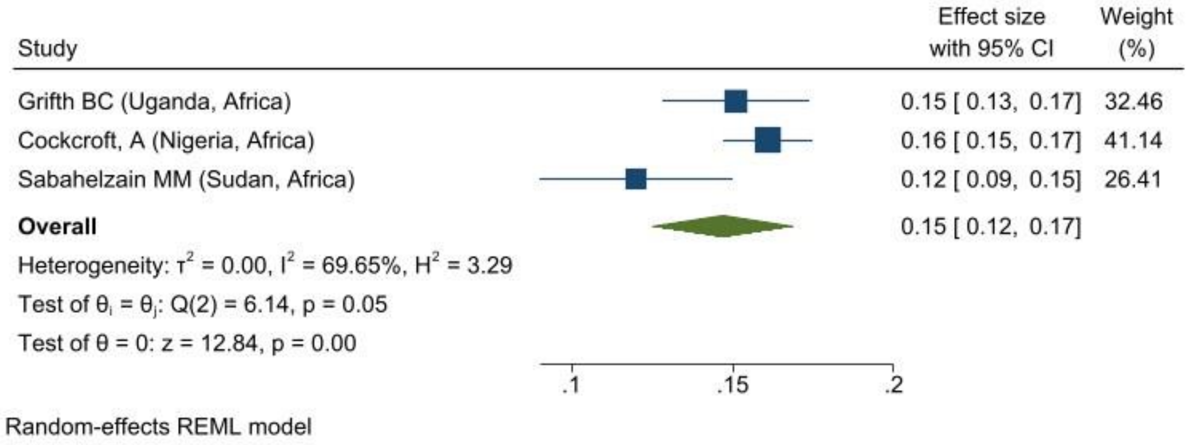

i. Africa region

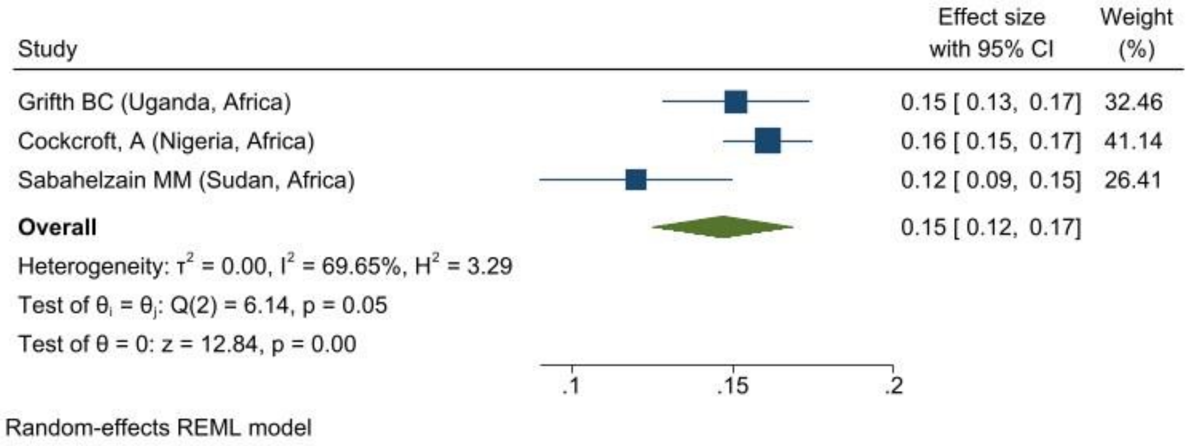

ii. Asia region

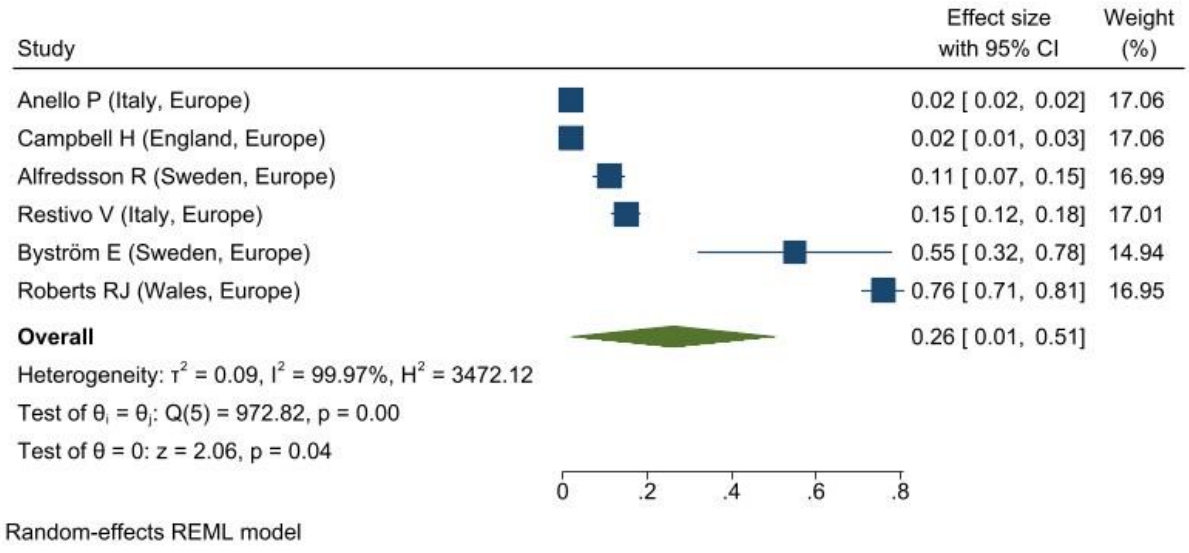

iii. *Europe region*

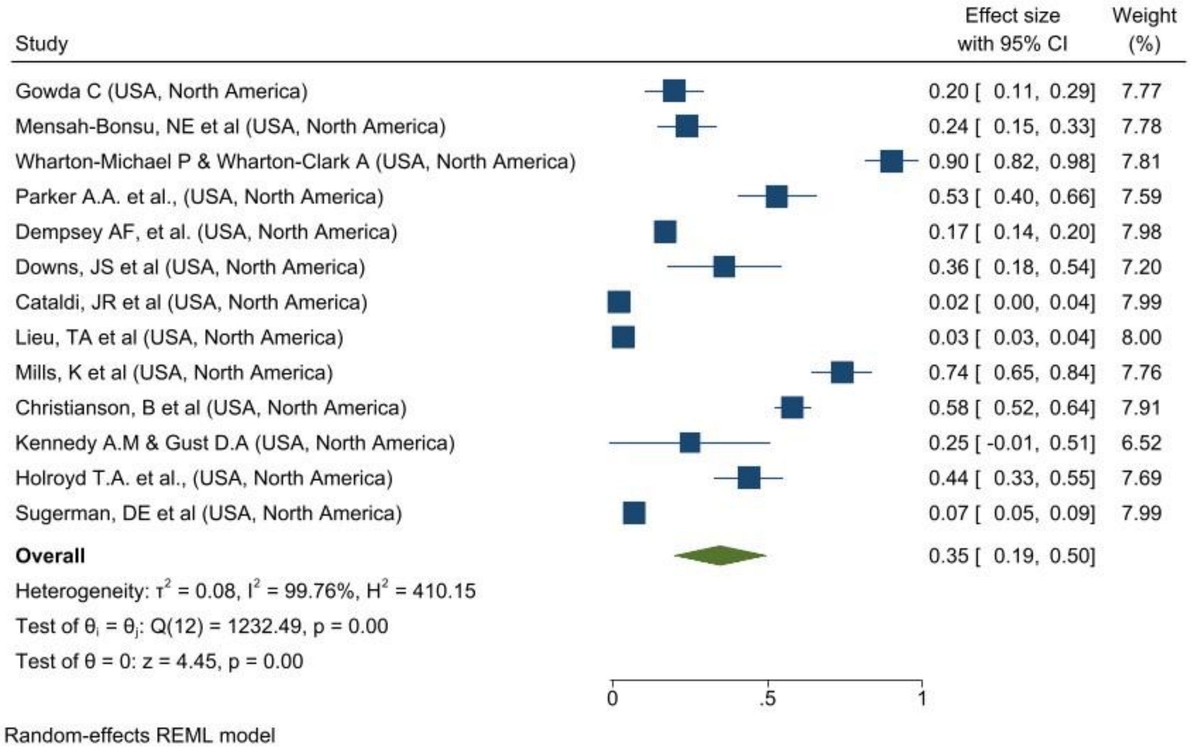

iv. *North America region*

7) Others vaccine

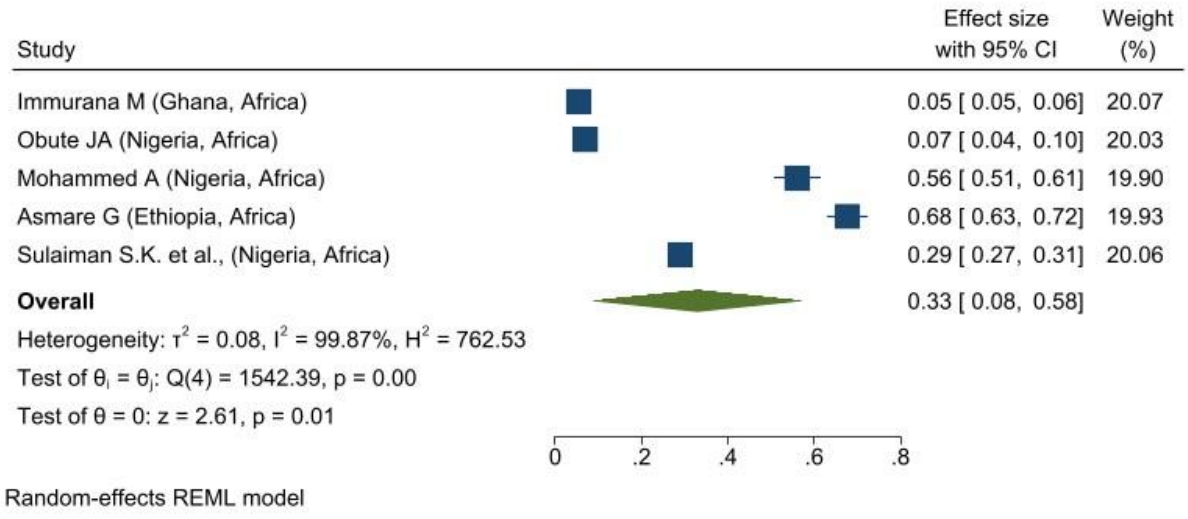

i. Africa region

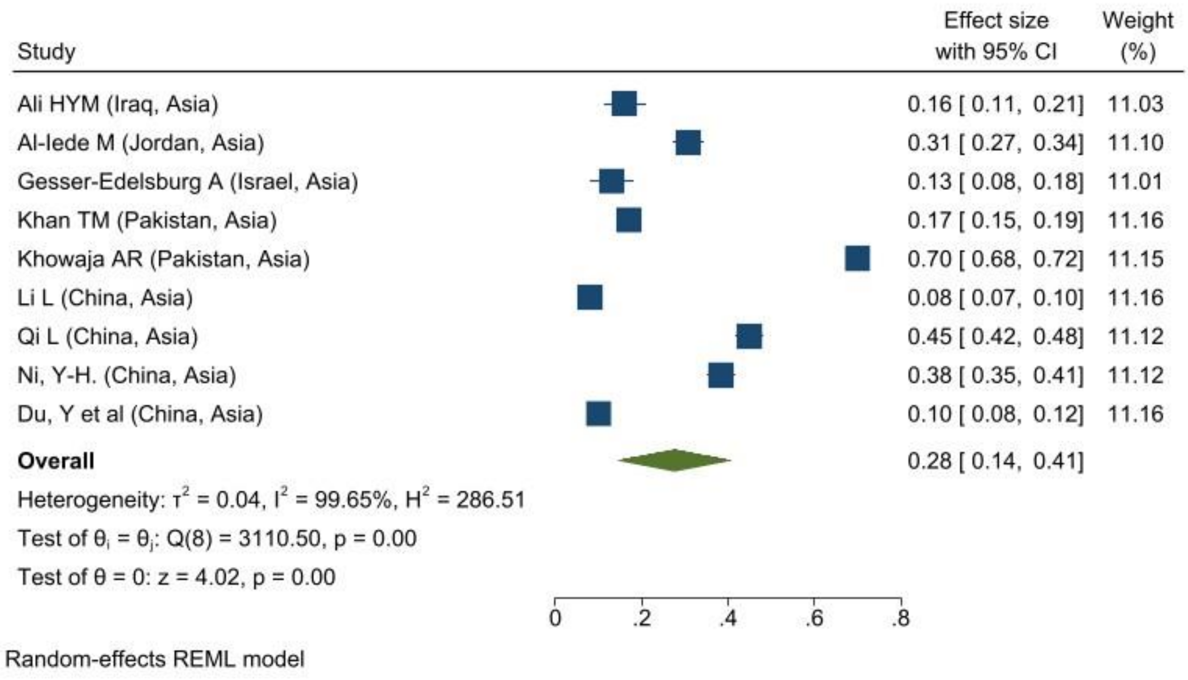

i. Asia region

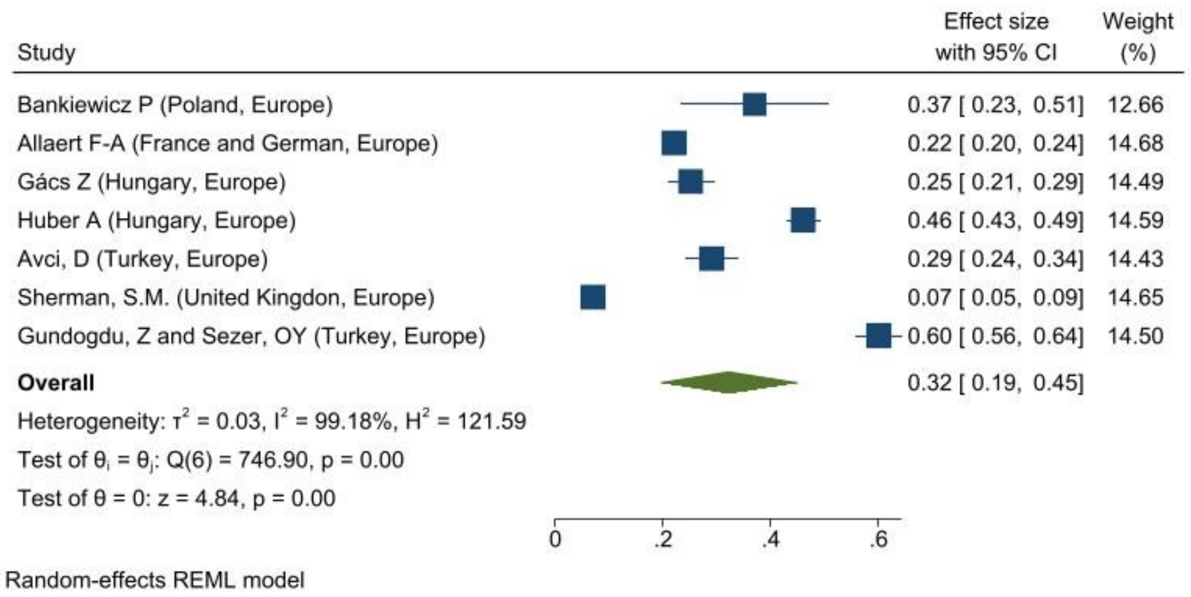

ii. Europe region

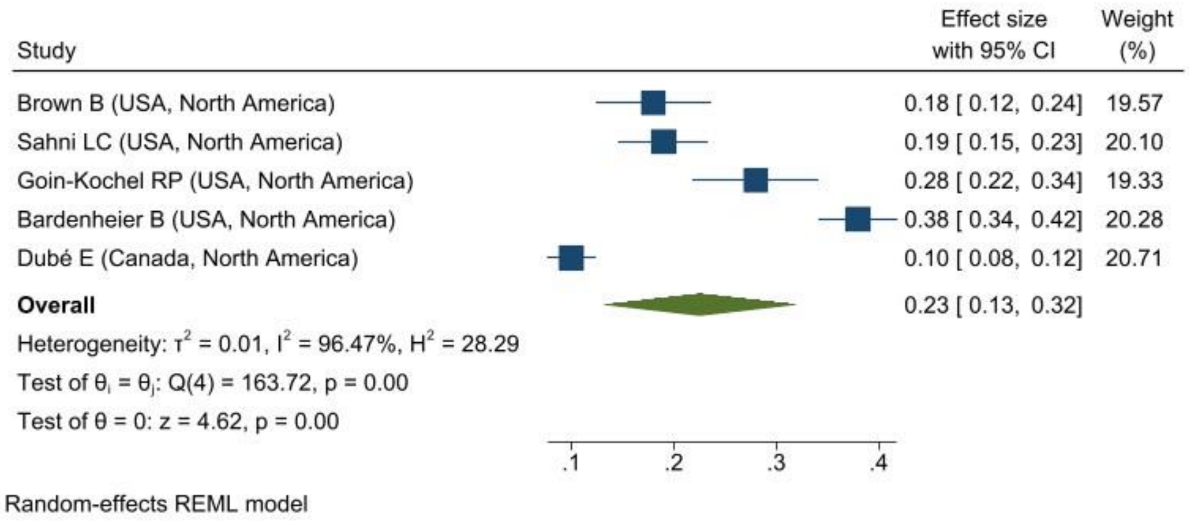

iii. North America region

8) Not stated vaccine

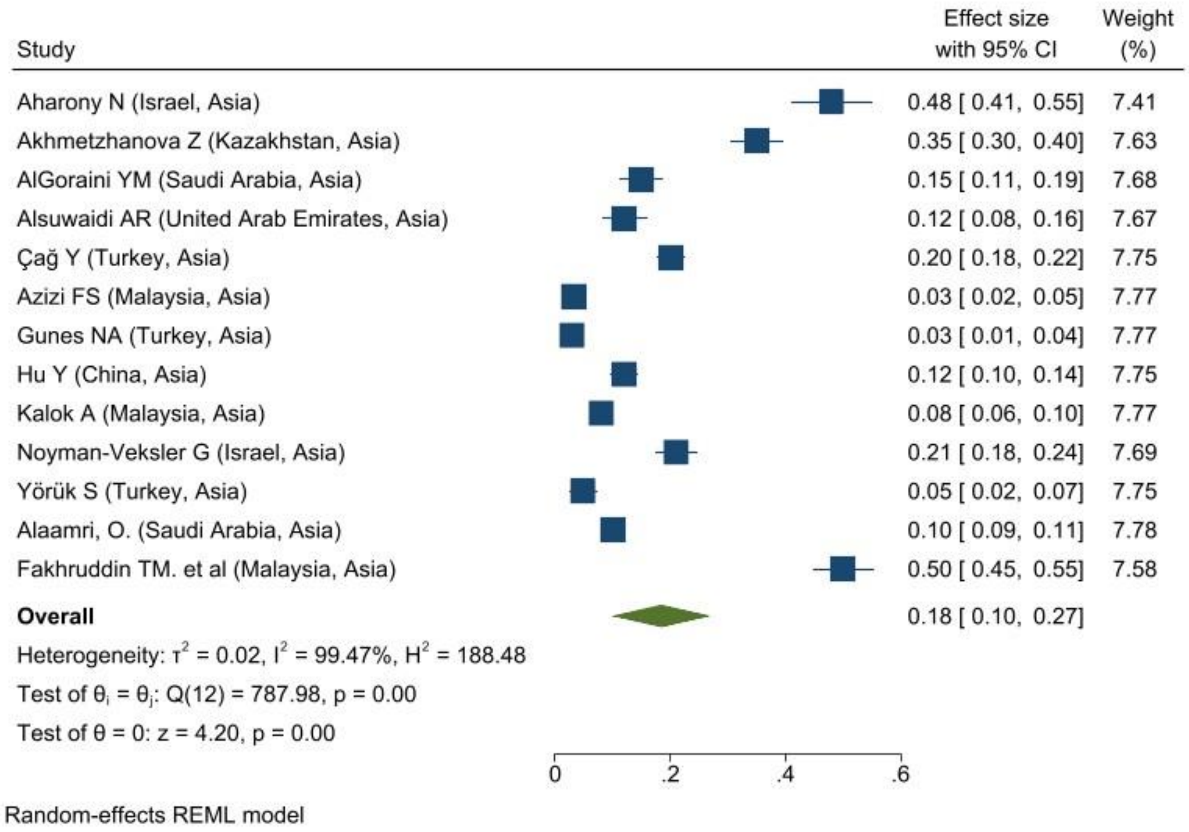

i. Asia region

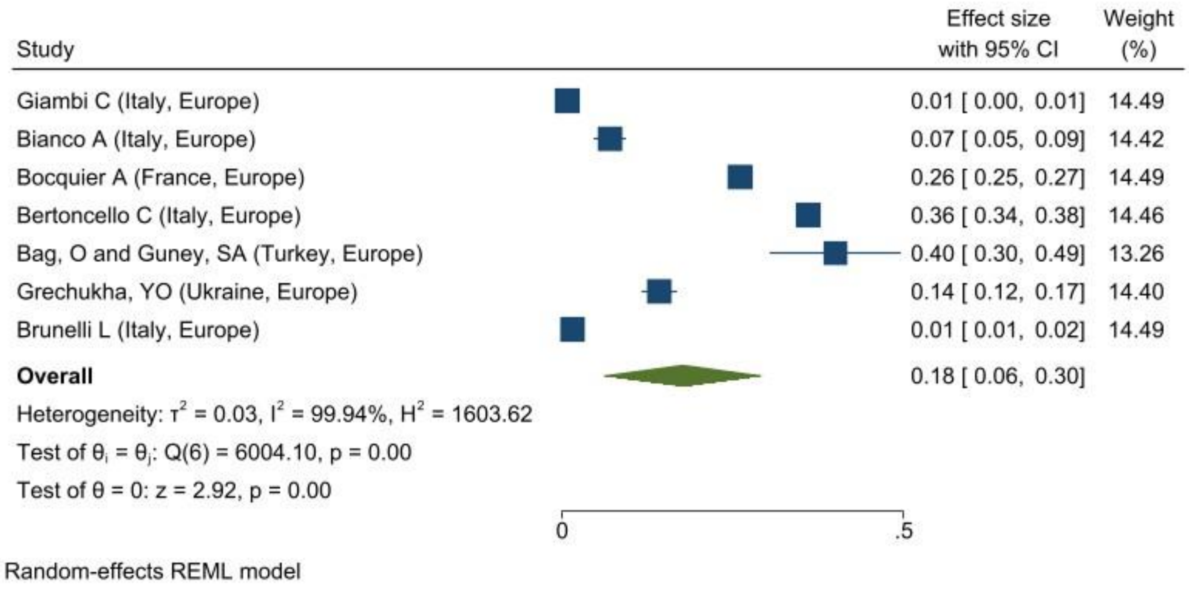

ii. Europe region

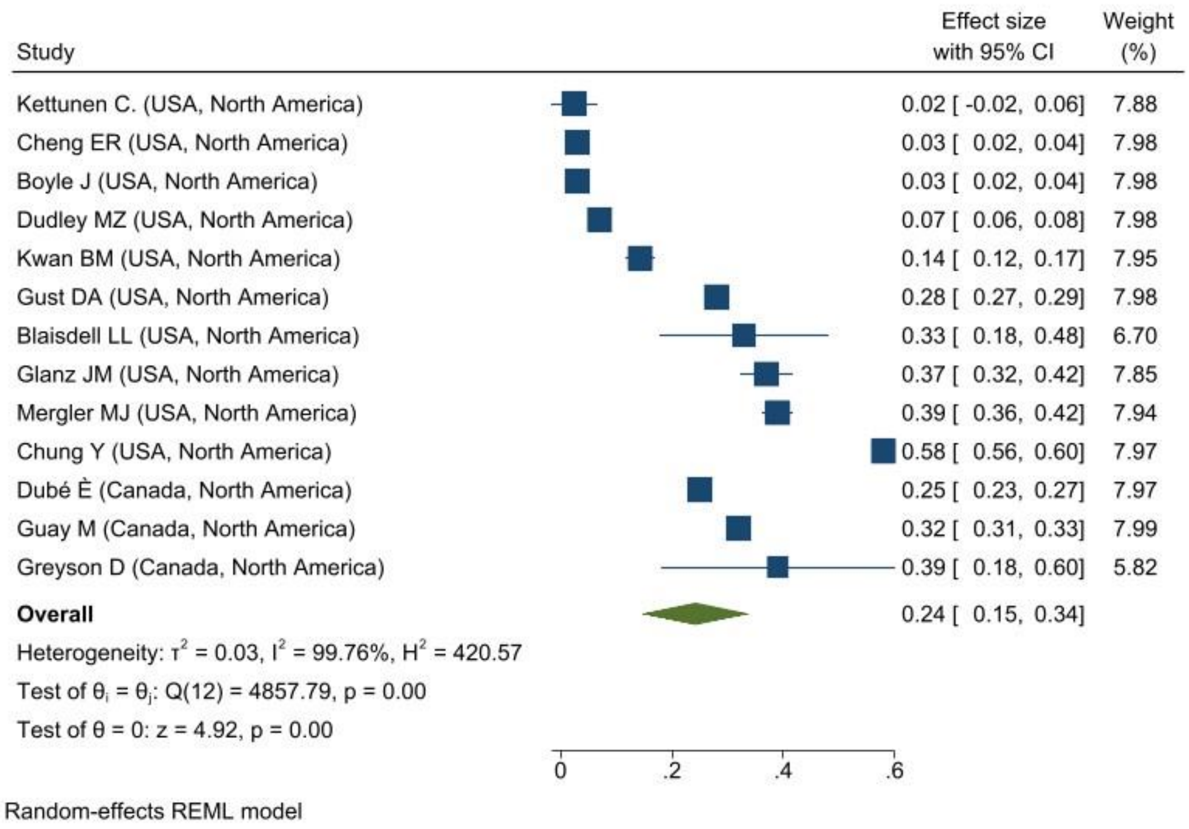

iii. North America region

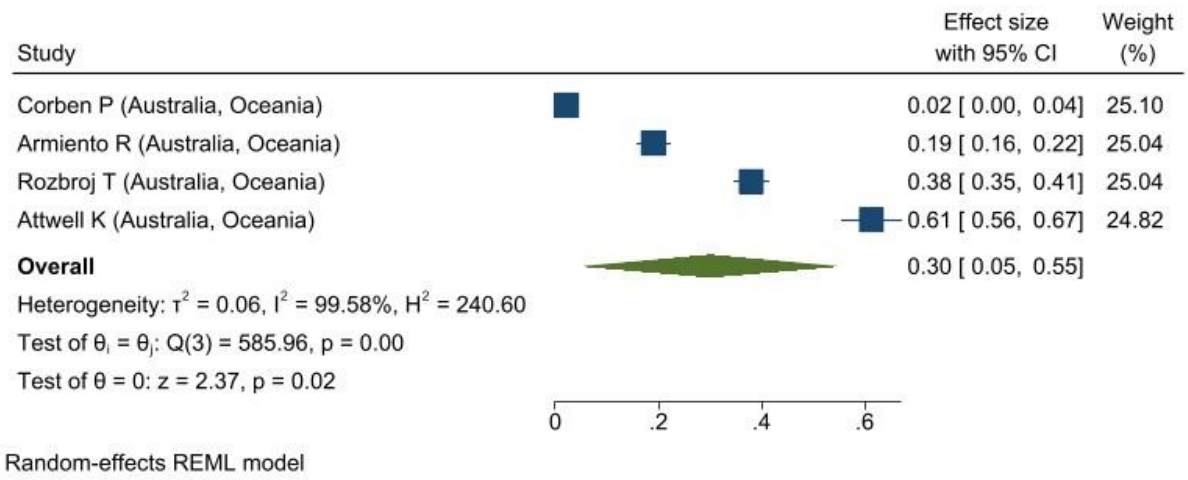

iv. Oceania region
