# Supplementary material for: Prevalence of caregiver hesitancy for vaccinations in children and its associated factors: A systematic review and meta-analysis
Source: PLoS One. 2024 Oct 24;19(10):e0302379. doi: 10.1371/journal.pone.0302379 (PMC11500859; doi:10.1371/journal.pone.0302379)
Supplement: S1 Table — (PDF) [file pone.0302379.s005.pdf]

S1 Table: Key Search Strategy Vaccine hesitancy

**Key search strategy**	**Search date**
The databases searched include PubMed, Google Scholar, ProQuest and Cochrane library.<br><br>**Keywords using PubMed Search**<br>1 Vaccine* Refusal* [Title/Abstract]<br>2 Anti Vaccination Movement* [Title/Abstract]<br>3 Anti vaccin* [Title/Abstract]<br>4 Antivaccin*[Title/Abstract]<br>5 Vaccin*[Title/Abstract]<br>6 Vaccination [Title/Abstract]<br>7 Mass Vaccination*[Title/Abstract]<br>8 Immunization [Title/Abstract]<br>9 Immunization Program* [Title/Abstract]<br>10 Treatment Refusal* [Title/Abstract]<br>11 Patient Acceptance of vaccin* [Title/Abstract]<br>12 Attitude to vaccin* [Title/Abstract]<br>13 Health* [Title/Abstract]<br>14 Knowledge* [Title/Abstract]<br>15 Vaccin* Practice [Title/Abstract]<br>16 Vaccin* Coverage* [Title/Abstract]<br>17 Vaccine* Refusal*[Title/Abstract] or Anti Vaccination Movement* [Title/Abstract]or Anti vaccin*[Title/Abstract]or Antivaccin*[Title/Abstract] or Vaccin*[Title/Abstract] or Vaccination[Title/Abstract] or Mass Vaccination*[Title/Abstract] or Immunization[Title/Abstract] or Immunization Program*[Title/Abstract] or Treatment Refusal*[Title/Abstract] or Patient Acceptance of vaccin*[Title/Abstract] or Attitude to vaccin*[Title/Abstract]or Health* Knowledge*[Title/Abstract] or Vaccin* Practice [Title/Abstract]or Vaccin* Coverage* [Title/Abstract]<br>18 Child*[Title/Abstract]<br>19 Preschool*[Title/Abstract]<br>20 Infant*[Title/Abstract]<br>21 Newborn*[Title/Abstract]<br>22 Neonat*[Title/Abstract]<br>23 Baby*[Title/Abstract]<br>24 Toddler*[Title/Abstract]<br>25 Young adult*[Title/Abstract]<br>26 Teenager*[Title/Abstract]<br>27 Child*[Title/Abstract] or Preschool*[Title/Abstract] or Infant*[Title/Abstract] or Newborn*[Title/Abstract] or Neonat*[Title/Abstract] or Baby* [Title/Abstract]or Toddler*[Title/Abstract] or Young adult*[Title/Abstract] or Teenager*[Title/Abstract]<br>28 Parent*[Title/Abstract]<br>29 Guardian*[Title/Abstract]<br>30 Caregiver*[Title/Abstract]<br>31 Mother*[Title/Abstract]<br>32 Father*[Title/Abstract]<br>33 Parent*[Title/Abstract] or Guardian*[Title/Abstract] or Caregiver*[Title/Abstract] or Mother*[Title/Abstract] or Father*[Title/Abstract]	January 2023 to August 2023

30 Vaccine* Refusal* [Title/Abstract]or Anti Vaccination Movement* [Title/Abstract]or Anti vaccin*[Title/Abstract]or Antivaccin* [Title/Abstract]or Vaccin*[Title/Abstract] or Vaccination or Mass Vaccination*[Title/Abstract] or Immunization or Immunization Program*[Title/Abstract] or Treatment Refusal*[Title/Abstract] or Patient Acceptance of vaccin*[Title/Abstract] or Attitude to vaccin*[Title/Abstract]or Health* Knowledge*[Title/Abstract] or Vaccin* Practice[Title/Abstract] or Vaccin* [Title/Abstract]AND Child*[Title/Abstract] or Preschool*[Title/Abstract] or Infant*[Title/Abstract] or Newborn* [Title/Abstract]or Neonat*[Title/Abstract] or Baby* [Title/Abstract]or Toddler*[Title/Abstract] or Young adult* [Title/Abstract]or Teenager* [Title/Abstract]AND Parent*[Title/Abstract] or Guardian*[Title/Abstract] or Caregiver*[Title/Abstract] or Mother* [Title/Abstract]or Father*[Title/Abstract]	

Note: This review not applied the MESH term during the search strategy.
