# Supplementary material for: Prevalence of caregiver hesitancy for vaccinations in children and its associated factors: A systematic review and meta-analysis
Source: PLoS One. 2024 Oct 24;19(10):e0302379. doi: 10.1371/journal.pone.0302379 (PMC11500859; doi:10.1371/journal.pone.0302379)
Supplement: S2 Table — (PDF) [file pone.0302379.s006.pdf]

S2 Table: Search Strategy Vaccine hesitancy using CoCoPop

**Co**	Condition or problem	Prevalence estimates of caregiver hesitancy
**Co**	Context	Contributing to the outcome such as caregiver knowledge, information, attitudes, safety and efficiency as well as beliefs regarding vaccines
**PoP**	Population	Parents; caregivers; guardians; mothers; fathers; infant; child; preschool child; adolescent; young adult
