# Supplementary material for: Prevalence of caregiver hesitancy for vaccinations in children and its associated factors: A systematic review and meta-analysis
Source: PLoS One. 2024 Oct 24;19(10):e0302379. doi: 10.1371/journal.pone.0302379 (PMC11500859; doi:10.1371/journal.pone.0302379)
Supplement: S3 Table — (PDF) [file pone.0302379.s007.pdf]

S3 Table: Data collection form

No	Author	Year of publication	Country	Region	Study design	Total of number responders	Religious belief	Knowledge	Information	Safety and efficacy	Others	Type of vaccine assess
1	Kyei-Arthur, F	2022	Ghana	Africa	Cross sectional	415	No	Yes	Yes	Yes	Yes	Covid-19
2	Al-Wutayd, O	2022	Egypt	Africa	Cross sectional	1458	No	Yes	Yes	Yes	Yes	Covid-19
3	Carcelen, AC	2022	Zimbabwe	Africa	Cross sectional	2400	No	Yes	Yes	Yes	No	Covid-19
4	Muluye, M	2022	Ethiopia	Africa	Cross sectional	874	No	Yes	Yes	Yes	Yes	Covid-19
5	Lubeya M.K. et al.,	2023	Zambia	Africa	Cross sectional	400	No	Yes	No	No	No	HPV
6	Balogun, FM	2022	Nigeria	Africa	Cross sectional	678	No	Yes	Yes	Yes	Yes	HPV
7	Elit L	2022	Cameroon	Africa	Cross sectional	45	No	Yes	Yes	Yes	Yes	HPV
8	Horn, S	2022	Kenya	Africa	Cross sectional	600	No	No	No	Yes	No	HPV
9	Kolek, C. O.	2022	Kenya	Africa	Cross sectional	195	Yes	No	Yes	Yes	Yes	HPV
10	Mihretie, G. N.	2022	Ethiopia	Africa	Cross sectional	638	No	Yes	Yes	Yes	Yes	HPV
11	Milondzo T. et al.,	2022	South Africa	Africa	Cross sectional	320	No	No	Yes	Yes	Yes	HPV
12	Popelsky, BK	2021	Ghana	Africa	Cross sectional	380	Yes	No	Yes	Yes	Yes	HPV
13	Kamya, C	2022	Uganda	Africa	Cross sectional	590	No	Yes	Yes	Yes	Yes	Mix
14	Darebo, TD	2022	Ethiopia	Africa	Cross sectional	657	No	Yes	Yes	Yes	No	Mix
15	Abor J	2022	Uganda	Africa	Cross sectional	420	No	Yes	Yes	Yes	Yes	Mix
16	Kemeugni Ngandjon, J.	2022	Cameroon	Africa	Cross sectional	160	Yes	Yes	Yes	Yes	Yes	Mix
17	Killion JP	2022	South Africa	Africa	Cross sectional	317	No	No	Yes	Yes	Yes	Mix
18	Tayu B	2022	Ethiopia	Africa	Cross sectional	633	No	Yes	Yes	No	Yes	Mix
19	Idris, I. O.	2022	Sudan	Africa	Cross sectional	428	No	No	Yes	Yes	No	Mix
20	Jalloh, MF	2022	Sierra Leone	Africa	Cross sectional	16	No	Yes	Yes	Yes	Yes	Mix
21	Powelson, J	2022	Mozambique	Africa	Cross sectional	32	No	No	Yes	Yes	Yes	Mix
22	Olaniyan, A. et al.,	2022	Nigeria	Africa	Cross sectional	44	No	Yes	Yes	Yes	Yes	Mix
23	Ames, H.	2021	Cameroon	Africa	Cross sectional	199	No	Yes	Yes	Yes	No	Mix
24	Vasudevan L	2020	Tanzania	Africa	Cross sectional	134	Yes	Yes	Yes	Yes	No	Mix
25	Kagone, M	2018	Burkina Faso	Africa	Cross sectional	32	No	Yes	Yes	Yes	Yes	Mix
26	Abakar, M.F.	2018	Chad	Africa	Cross sectional	47	No	No	Yes	Yes	No	Mix
27	Tadesse, T.	2017	Ethiopia	Africa	Cross sectional	693	No	Yes	Yes	Yes	Yes	Mix
28	Handy, LK	2017	Botswana	Africa	Cross sectional	96	No	Yes	Yes	No	No	Mix
29	Tabana, H	2016	South Africa	Africa	Cross sectional	229	No	Yes	Yes	Yes	No	Mix
30	Zewdie, A	2016	Ethiopia	Africa	Cross sectinoal	28	No	Yes	Yes	Yes	Yes	Mix
31	Babirye, J.N	2011	Uganda	Africa	Cross sectinoal	73	No	No	Yes	Yes	Yes	Mix
32	Schwarz, NG	2009	Gaban	Africa	Cross sectinoal	262	No	No	No	Yes	Yes	Mix
33	Tadesse, H.	2009	Ethiopia	Africa	Cross sectinoal	6	No	Yes	Yes	Yes	Yes	Mix
34	Leach, MA	2008	Guinea	Africa	Cross sectinoal	1550	No	Yes	Yes	Yes	No	Mix
35	Cassell, JA	2006	Gambia	Africa	Cross sectinoal	1600	No	Yes	Yes	Yes	Yes	Mix
36	Helman, CG	2004	Transkei	Africa	Cross sectinoal	60	No	Yes	Yes	Yes	Yes	Mix
37	Eng, E.	1991	Togo	Africa	Cross sectinoal	110	No	Yes	Yes	No	No	Mix
38	Cutts, FT	1990	Guinea	Africa	Cross sectinoal	24	No	Yes	Yes	Yes	Yes	Mix
39	Griffith, BC	2022	Uganda	Africa	Cross sectional	999	No	Yes	No	No	Yes	MMR
40	Hailu, C.	2022	Ethiopia	Africa	Case control	487	No	Yes	Yes	Yes	Yes	MMR
41	Sabahelzain, M.M.	2022	Sudan	Africa	Cross sectional	495	No	Yes	Yes	Yes	No	MMR

42	Gil Cuesta, JG	2021	Guinea	Africa	Cross sectional	68	Yes	Yes	No	Yes	No	MMR
43	Machekanyanga Z	2017	Zimbabwe	Africa	Case control	90	Yes	Yes	Yes	Yes	No	MMR
44	Cockcroft, A	2014	Nigeria	Africa	Cross sectional	2836	No	Yes	Yes	Yes	Yes	MMR
45	Asmare, G.	2022	Ethiopia	Africa	Cross sectional	635	No	Yes	Yes	Yes	Yes	Not stated
46	Dejene, H.	2022	Ethiopia	Africa	Cross sectional	413	No	Yes	Yes	Yes	Yes	Not stated
47	Mebrate, M.	2022	Ethiopia	Africa	Cross sectional	664	No	Yes	Yes	Yes	No	Not stated
48	Abdullahi MF	2020	Somalia	Africa	Cross sectional	48	Yes	Yes	Yes	Yes	No	Not stated
49	Tefera YA	2018	Ethiopia	Africa	Cross sectional	484	Yes	Yes	Yes	Yes	No	Not stated
50	Mcknight, J and Holt, DB	2014	Ethiopia	Africa	Cross sectional	83	No	Yes	Yes	No	Yes	Not stated
51	Dugas M	2009	Burkina Faso	Africa	Cross sectional	2052	Yes	Yes	Yes	Yes	No	Not stated
52	Antai D	2008	Nigeria	Africa	Cross sectional	9966	Yes	Yes	Yes	Yes	No	Not stated
53	Sulaiman S.K. et al.,	2023	Nigeria	Africa	Cross sectional	3377	Yes	No	No	Yes	Yes	Others
54	Asmare, G.	2022	Ethiopia	Africa	Cross sectional	406	Yes	Yes	Yes	Yes	Yes	Others
55	Yeboah, D.	2022	Ghana	Africa	Cross sectional	422	No	Yes	Yes	Yes	No	Others
56	Immurana, M	2021	Ghana	Africa	Cross sectional	3004	No	Yes	Yes	Yes	Yes	Others
57	Abubakar A	2019	Nigeria	Africa	Case Control	465	Yes	Yes	Yes	Yes	No	Others
58	Fleming, JA	2019	Malawi	Africa	Cross sectional	274	No	Yes	Yes	Yes	Yes	Others
59	Stamidis, KV	2019	Ethiopia	Africa	Cross sectional	151	No	Yes	Yes	Yes	No	Others
60	Umeh GC	2018	Nigeria	Africa	Cross sectional	396	Yes	Yes	Yes	Yes	No	Others
61	Closser, S	2016	Ethiopia	Africa	Cross sectional	50	Yes	No	Yes	No	Yes	Others
62	Giles-Vernick, T	2016	Burkina Faso	Africa	Cross sectional	68	No	Yes	Yes	Yes	No	Others
63	Mohammed A	2014	Nigeria	Africa	Case control	121	Yes	Yes	Yes	Yes	No	Others
64	Obute JA	2007	Nigeria	Africa	Cross sectional	353	Yes	Yes	Yes	Yes	No	Others
65	Renne, E	2006	Nigeria	Africa	Cross sectional	27	Yes	Yes	No	Yes	No	Others
66	Yousaf A. R.	2023	United State	North America	Cohort study	94	No	No	Yes	Yes	No	Covid-19
67	Wigle J.	2023	Canada	North America	Cross sectional	20	No	Yes	Yes	Yes	Yes	Covid-19
68	Allen, JD	2023	United State	North America	Cross sectional	591	No	Yes	Yes	Yes	Yes	Covid-19
69	Arrigoni, L	2023	United State	North America	Cohort study	28	No	No	Yes	Yes	No	Covid-19
70	Batra, K.	2023	United State	North America	Cross sectional	263	No	Yes	Yes	Yes	Yes	Covid-19
71	Davidson, CA et al	2023	Canada	North America	Cross sectional	129	No	Yes	Yes	Yes	Yes	Covid-19
72	Durkin, LK	2023	United State	North America	Cross sectional	144	No	No	Yes	Yes	Yes	Covid-19
73	Gooding, GD et al	2023	Canada	North America	Cross sectional	146	No	No	Yes	Yes	No	Covid-19
74	Mondal P & Sinharoy A	2023	United State	North America	Cross sectional	2622	No	No	Yes	Yes	Yes	Covid-19
75	Kohler, RE et al	2023	United State	North America	Cross sectional	22	No	No	Yes	Yes	Yes	Covid-19
76	Shen A.K.	2022	United State	North America	Cross sectional	971	No	No	Yes	Yes	Yes	Covid-19
77	Hill, AV	2022	United State	North America	Cross sectional	299	No	No	No	No	No	Covid- 19
78	Zhu, Y	2022	United State	North America	Cross sectional	714	No	Yes	Yes	Yes	No	Covid 19
79	Phan TL	2022	United America	North America	Cross sectional	513	No	Yes	Yes	Yes	No	Covid-19
80	Ali S	2022	Canada	North America	Cross sectional	331	No	Yes	Yes	No	Yes	Covid-19
81	Baumer-Mouradian, S.H.	2022	United State	North America	Cross sectional	4393	No	No	Yes	Yes	No	Covid-19
82	Cioffredi, LA	2022	United State	North America	Cross sectional	78	No	Yes	Yes	Yes	Yes	Covid-19
83	de St Maurice, A.	2022	United State	North America	Cross sectional	12288	No	Yes	Yes	Yes	No	Covid-19
84	Delgado, JR	2022	United State	North America	Cross sectional	1051	No	Yes	No	No	Yes	Covid-19
85	Drouin, O.	2022	Canada	North America	Cross sectional	305	No	Yes	Yes	Yes	No	Covid-19
86	Egbert N	2022	United State	North America	Cross sectional	702	No	Yes	No	Yes	No	Covid-19
87	Goulding, M	2022	United State	North America	Cross sectional	67	No	Yes	Yes	Yes	No	Covid-19

88	Gray A	2022	United State	North America	Cross sectional	242	No	Yes	Yes	Yes	Yes	Covid-19
89	Guerin, RJ	2022	United State	North America	Cross sectional	7298	No	No	No	No	Yes	Covid-19
90	Hammershaimb, EA	2022	United State	North America	Cross sectional	3230	No	Yes	Yes	Yes	No	Covid-19
91	Head, K.J	2022	United State	North America	Cross sectional	10266	No	No	Yes	Yes	No	Covid-19
92	Hopfer, S	2022	United State	North America	Cross sectional	46	No	Yes	Yes	Yes	Yes	Covid-19
93	Humble, RM	2022	Canada	North America	Cross sectional	1129	No	Yes	Yes	Yes	Yes	Covid-19
94	Kreuter, MW	2022	United State	North America	Cross sectional	1951	No	No	No	Yes	No	Covid-19
95	Lam, C.N	2022	United State	North America	Cross sectional	401	No	Yes	Yes	Yes	No	Covid-19
96	Limbers, CA	2022	United State	North America	Cross sectional	821	No	Yes	Yes	Yes	No	Covid-19
97	Mangat, C	2022	United State	North America	Cross sectional	1301	No	No	No	Yes	No	Covid-19
98	McElfish, P.A.	2022	United State	North America	Cross sectional	369	No	Yes	Yes	Yes	No	Covid-19
99	Nguyen, KH	2022	United State	North America	Cross sectional	11478	No	Yes	Yes	Yes	Yes	Covid-19
100	Nguyen, KH	2022	United State	North America	Cross sectional	59424	No	No	No	Yes	Yes	Covid-19
101	Panchalingam, T.	2022	United State	North America	Cross sectional	1456	No	Yes	Yes	Yes	No	Covid-19
102	Reindl, D.	2022	United State	North America	Cross sectional	582	No	No	Yes	Yes	No	Covid-19
103	Salazar, TL	2022	United State	North America	Cross sectional	93	No	Yes	Yes	Yes	No	Covid-19
104	Scherer, AM	2022	United State	North America	Cross sectional	2031	No	Yes	Yes	Yes	No	Covid-19
105	Schiff, J.	2022	United State	North America	Cross sectional	58	No	Yes	Yes	Yes	Yes	Covid-19
106	Schilling, S	2022	United State	North America	Cross sectional	50	No	Yes	Yes	Yes	No	Covid-19
107	Skeens, M.	2022	United State	North America	Cross sectional	113	No	Yes	Yes	Yes	No	Covid-19
108	Skeens, M. A.	2022	United State	North America	Cross sectional	491	No	No	Yes	Yes	Yes	Covid-19
109	Srivastava, T	2022	United State	North America	Cohort study	334	No	Yes	Yes	Yes	Yes	Covid-19
110	Suvada, KA	2022	United State	North America	Cross sectional	6068	No	Yes	Yes	Yes	Yes	Covid-19
111	Temple, AM	2022	United State	North America	Cross sectional	204	No	Yes	Yes	Yes	No	Covid-19
112	Willis, DE	2022	United State	North America	Cross sectional	665	No	No	No	No	No	Covid-19
113	Ruiz JB	2022	United State	North America	Cross sectional	637	No	Yes	Yes	Yes	Yes	Covid-19
114	Lacy, R.	2022	United State	North America	Cross sectional	41	No	Yes	Yes	Yes	No	Covid-19
115	Lessard, L	2022	United State	North America	Cross sectional	168	No	Yes	Yes	Yes	No	Covid-19
116	Bonuck, K	2022	United State	North America	Cross sectional	352	No	Yes	Yes	Yes	Yes	Covid-19
117	Byrne, A	2022	United State	North America	Cross sectional	2620	No	Yes	Yes	Yes	No	Covid-19
118	Dayton, L.	2022	United State	North America	Cohort study	297	No	Yes	Yes	Yes	Yes	Covid-19
119	Ellithorpe, ME	2022	United State	North America	Cross sectional	682	No	No	Yes	Yes	No	Covid-19
120	Fisher, C.B.	2022	United State	North America	Cross sectional	411	No	Yes	Yes	Yes	Yes	Covid-19
121	O'Dor SL	2022	United State	North America	Cross sectional	254	No	No	Yes	Yes	No	Covid-19
122	M. Baumann, B	2022	United State	North America	Cross sectional	1298	Yes	Yes	Yes	Yes	No	Covid-19
123	Santibanez, TA	2022	United State	North America	Cross sectional	4496	No	Yes	Yes	Yes	Yes	Covid-19
124	Wang, CH	2022	United State	North America	Cross sectional	121	No	Yes	Yes	Yes	No	Covid-19
125	Yeo, J	2022	Canada	North Amerika	Cross sectional	23	No	Yes	Yes	Yes	No	Covid-19
126	Olagoke, A.	2022	United State	North America	Cross sectional	342	Yes	No	No	Yes	No	Covid-19
127	Lachance-Grzela, M. et al.,	2022	Canada	North America	Cross sectional	406	No	No	Yes	Yes	Yes	Covid-19
128	Wojcicki, JM et al	2022	United State	North America	Cohort study	391	No	No	Yes	Yes	Yes	Covid-19
129	Kheil, M.H. et al.,	2022	United State	North America	Cross sectional	1746	Yes	No	No	Yes	Yes	Covid-19
130	Choi, K. et al.,	2022	United State	North America	Cross sectional	322	No	Yes	Yes	Yes	No	Covid-19
131	Alferi, N. L	2021	United State	North America	Cross sectional	1425	No	Yes	Yes	Yes	No	Covid-19
132	Humble, RM	2021	Canada	North America	Cross sectional	1702	No	Yes	Yes	Yes	No	Covid-19
133	Marquez, RR	2021	United State	North America	Cross sectional	97	No	Yes	Yes	Yes	Yes	Covid-19

134	McKinnon, B.	2021	Canada	North America	Cross sectional	809	No	Yes	Yes	Yes	No	Covid-19
135	Szilagyi, PG	2021	United State	North America	Cross sectional	1745	No	Yes	Yes	Yes	No	Covid-19
136	Teasdale, CA	2021	United State	North America	Cross sectional	1119	Yes	No	No	Yes	Yes	Covid-19
137	Teasdale, CA	2021	United State	North America	Cross sectional	2074	Yes	No	No	Yes	Yes	Covid-19
138	Waring, M. E.	2022	United State	North America	Cross sectional	203	No	Yes	Yes	Yes	No	Covid-19
139	Delgado-Gallegos, J.L.	2021	Mexico	North America	Cross sectional	699	No	Yes	Yes	Yes	No	Covid-19
140	Hetherington, E	2021	Canada	North Amerika	Cohort study	1321	No	Yes	Yes	Yes	Yes	Covid-19
141	Lackner, CL	2021	Canada	North Amerika	Cross sectional	455	No	No	Yes	Yes	No	Covid-19
142	Middleman A.B. et al.,	2021	United State	North America	Cohort study	500	No	No	Yes	Yes	Yes	Covid-19
143	Sokol, RL and Grummom, AH	2020	United State	North America	Cross sectional	1893	No	No	Yes	Yes	No	Covid-19
144	Davis, MM et al	2020	United State	North America	Cross sectional	1008	No	No	Yes	Yes	No	Covid-19
145	Zhu X	2023	United State	North America	Cross sectional	342	No	Yes	No	Yes	Yes	HPV
146	Shin, M.B.	2023	United State	North America	Cross sectional	20	No	Yes	Yes	Yes	Yes	HPV
147	Jin, SW et al	2023	United State	North America	Cross sectional	497	No	Yes	No	Yes	No	HPV
148	Anuforo, B.	2022	United State	North America	Cross sectional	77	No	Yes	Yes	Yes	No	HPV
149	Ayash, C	2022	United State	North America	Cross sectional	162	Yes	Yes	Yes	Yes	Yes	HPV
150	Ayres, S	2022	United State	North America	Cross sectional	99	No	Yes	No	No	No	HPV
151	Beavis, AL	2022	United State	North America	Cross sectional	22	No	Yes	Yes	Yes	Yes	HPV
152	Chido-Amajuoyi, OG	2022	United State	North America	Cross sectional	38706	No	No	No	Yes	Yes	HPV
153	Lee, G.	2022	United State	North America	Cross sectional	275	No	No	Yes	Yes	Yes	HPV
154	Rositch, A. F.	2022	United State	North America	Cross sectional	13090	No	Yes	Yes	Yes	Yes	HPV
155	Staras, SA	2022	United State	North America	Cross sectional	25	No	Yes	Yes	Yes	Yes	HPV
156	Thompson, EL	2022	United State	North America	Cross sectional	1192	No	Yes	Yes	No	No	HPV
157	Tsui, J.	2022	United State	North America	Cross sectional	357	No	Yes	Yes	Yes	No	HPV
158	Varisco, T. J.	2022	United State	North America	Cross sectional	416	No	Yes	Yes	Yes	No	HPV
159	Vasudevan, L.	2022	United State	North America	Cross sectional	987	No	Yes	Yes	Yes	No	HPV
160	Vu, M	2022	United State	North America	Cross sectional	32	No	Yes	Yes	Yes	Yes	HPV
161	Xiong, S	2022	United State	North America	Cross sectional	25	No	Yes	Yes	Yes	Yes	HPV
162	Cui Z. et al.,	2022	United State	North America	Cohort study	243	No	No	Yes	Yes	No	HPV
163	Margolis M. A. et al.,	2022	United State	North America	Cross sectional	1263	No	No	Yes	No	Yes	HPV
164	Dundar, Y.	2021	United State	North America	Cross sectional	150	No	Yes	Yes	Yes	No	HPV
165	Myhre A	2020	United State	North America	Cross sectional	355	Yes	Yes	Yes	Yes	No	HPV
166	Khodadadi AB	2020	United State	North America	RCT	317	Yes	Yes	Yes	Yes	No	HPV
167	Newcomer SR	2020	United State	North America	Cross sectional	71	Yes	Yes	Yes	Yes	No	HPV
168	Pomares TD	2020	United State	North America	Cross sectional	1400	Yes	Yes	Yes	Yes	No	HPV
169	Yankey D	2020	United State	North America	Cross sectional	40929	Yes	Yes	Yes	Yes	No	HPV
170	Duchsherer A. et al.,	2020	United State	North America	Cross sectional	343	No	Yes	Yes	Yes	Yes	HPV
171	Auslander BA	2019	United State	North America	Cross sectional	50	Yes	Yes	Yes	Yes	No	HPV
172	Hirth JM	2019	United State	North America	Cross sectional	90866	Yes	Yes	Yes	Yes	No	HPV
173	Thomas TL	2019	United State	North America	Cross sectional	123	Yes	Yes	Yes	Yes	No	HPV
174	Beavis A	2018	United State	North America	Cross sectional	46853	Yes	Yes	Yes	Yes	No	HPV
175	Hanson KE	2018	United State	North America	Cross sectional	76971	Yes	Yes	Yes	Yes	No	HPV
176	Krok-Schoen JL	2018	United State	North America	Cross sectional	337	Yes	Yes	Yes	Yes	No	HPV
177	Lee Y.M	2018	United State	North America	Cross sectional	74	Yes	Yes	Yes	Yes	No	HPV
178	Albright K	2017	United State	North America	Cross sectional	41	Yes	Yes	Yes	Yes	No	HPV
179	Brown B	2017	United State	North America	Cross sectional	200	Yes	Yes	Yes	Yes	No	HPV

180	Gilkey MB	2017	United State	North America	Cross sectional	1484	Yes	Yes	Yes	Yes	No	HPV
181	Thompson EL	2017	United State	North America	Cross sectional	59897	Yes	Yes	Yes	Yes	No	HPV
182	Gilbert NL	2016	Canada	North America	Cross sectional	5720	Yes	Yes	Yes	Yes	No	HPV
183	Krawczyk A	2015	Canada	North America	Cross sectional	806	Yes	Yes	Yes	Yes	Yes	HPV
184	Perez S	2015	Canada	North America	Cross sectional	2874	Yes	Yes	Yes	Yes	No	HPV
185	Dorell C	2014	United State	North America	Cross sectional	4103	Yes	Yes	Yes	Yes	No	HPV
186	Staras SA	2014	United State	North America	Cross sectional	2127	Yes	Yes	Yes	Yes	No	HPV
187	Pitts MJ	2013	United State	North America	Cross sectional	33	Yes	Yes	Yes	Yes	No	HPV
188	Ogilvie G	2010	Canada	North America	Cross sectional	2025	Yes	Yes	Yes	Yes	No	HPV
189	Dempsey MF	2009	United State	North America	Cross sectional	52	Yes	Yes	Yes	Yes	No	HPV
190	Brabin L	2008	United State	North America	Cohort study	2817	Yes	Yes	Yes	Yes	No	HPV
191	Toffolon-Weiss M	2008	United State	North America	Cross sectional	79	Yes	Yes	Yes	Yes	No	HPV
192	Nguyen, AT	2022	United State	North America	Cross sectional	59424	No	No	No	Yes	Yes	Influenza
193	Fisher, W.A	2022	Canada	North America	Cohort study	207	No	Yes	Yes	Yes	Yes	Influenza
194	Beatty and Villwock	2021	United State	North America	Cross sectional	179	No	No	Yes	Yes	No	Influenza
195	Fogel BN	2020	United State	North America	Cross sectional	141	Yes	Yes	Yes	Yes	No	Influenza
196	Kempe A	2020	United State	North America	Cross sectional	2176	Yes	Yes	Yes	Yes	No	Influenza
197	Nekrasov E	2020	United State	North America	Cross sectional	257	Yes	Yes	Yes	Yes	No	Influenza
198	Goss MD	2020	United State	North America	Cross sectional	244	Yes	Yes	Yes	Yes	No	Influenza
199	Hofstetter AM	2018	United State	North America	Cross sectional	199	Yes	Yes	Yes	Yes	No	Influenza
200	Baumgaetner, B et al	2018	United State	North America	Cross sectional	1006	No	Yes	Yes	Yes	Yes	Influenza
201	Cameron MA	2016	United State	North America	Cross sectional	786	Yes	Yes	Yes	Yes	No	Influenza
202	Santibanez TA	2016	United State	North America	Cross sectional	3548	Yes	Yes	Yes	Yes	No	Influenza
203	Fuchs, EL	2016	United State	North America	Cohort study	4022	No	No	Yes	Yes	No	Influenza
204	Strelitz B	2015	United State	North America	Cross sectional	152	Yes	Yes	Yes	Yes	No	Influenza
205	Frew PM	2011	United State	North America	Cross sectional	223	Yes	Yes	Yes	Yes	No	Influenza
206	Schellenberg, N.	2023	Canada	North America	Cross sectional	6125	Yes	Yes	No	Yes	Yes	Mix
207	Shen, AK	2023	United State	North America	Cross sectional	41	Yes	Yes	Yes	Yes	Yes	Mix
208	Baumer-Mouradian, S.H.	2022	United State	North America	Cross sectional	589	No	Yes	Yes	Yes	Yes	Mix
209	Ellithorpe, M	2022	United State	North America	Cross sectional	779	No	No	No	Yes	No	Mix
210	Freeman RE	2022	United State	North America	Cross sectional	15333	No	No	No	No	Yes	Mix
211	He, K	2022	United State	North America	Cross sectional	175	No	Yes	Yes	Yes	Yes	Mix
212	Howell, JL	2022	United State	North America	Cross sectional	863	Yes	Yes	Yes	Yes		Mix
213	Hsu, C	2022	United State	North America	Cross sectional	41	No	Yes	Yes	Yes	Yes	Mix
214	Kaufmann J	2022	United State	North America	Cross sectional	35444	No	No	No	No	No	Mix
215	Letterie MC	2022	United State	North America	Cross sectional	1066	No	No	Yes	No	Yes	Mix
216	Nguyen, K.H	2022	United State	North America	Cross sectional	7645	No	Yes	Yes	Yes	Yes	Mix
217	Opel, D. J.	2022	United State	North America	Cross sectional	4562	No	No	Yes	Yes	No	Mix
218	Shah, MD	2022	United State	North America	Cross sectional	1009	No	Yes	No	Yes	No	Mix
219	Wang, CS	2022	United State	North America	Cross sectional	207	No	Yes	Yes	Yes	No	Mix
220	Anderson-Chavarria, M	2022	Puerto Rico	North America	Cross sectional	35	No	No	Yes	Yes	No	Mix
221	Boyce, TG	2022	United State	North America	Cross sectional	536	Yes	Yes	Yes	Yes	No	Mix
222	Footman, A	2022	United State	North America	Cross sectional	21	No	Yes	Yes	Yes	Yes	Mix
223	Cole J.W. et al.,	2022	United State	North America	Cohort study	2504	No	No	Yes	Yes	No	Mix
224	Gennaro, E et al	2021	United State	North America	Cross sectional	135	No	No	Yes	Yes	Yes	Mix
225	Newcomer S.R. et al.,	2021	United State	North America	Cohort study	31422	No	No	No	No	Yes	Mix

226	Langkamp, DL et al	2020	United State	North America	Cross sectional	63	No	No	No	Yes	Yes	Mix
227	Glanz, JM et al	2020	United State	North America	RCT	824	No	No	Yes	Yes	No	Mix
228	Navin MC	2019	United State	North America	Cross sectional	4098	Yes	Yes	Yes	Yes	No	Mix
229	Carpiano RM	2019	Canada	North America	Cross sectional	12478	Yes	Yes	Yes	Yes	No	Mix
230	Deas J	2018	United State	North America	Cross sectional	39	Yes	Yes	Yes	Yes	No	Mix
231	Gilkey MB	2016	United State	North America	Cross sectional	9354	Yes	Yes	Yes	Yes	No	Mix
232	Gilkey MB	2016	United State	North America	Cross sectional	9018	Yes	Yes	Yes	Yes	No	Mix
233	Wolf E. et al.,	2016	United State	North America	Cohort study	277098	No	No	No	No	Yes	Mix
234	William S.E. et al.,	2016	United State	North America	Cross sectional	158	No	No	No	No	No	Mix
235	Frew, PM et al	2016	United State	North America	Cross sectional	5121	No	No	Yes	Yes	No	Mix
236	Greenfield LS	2015	United State	North America	Cross sectional	202	Yes	Yes	Yes	Yes	No	Mix
237	Roberts JR	2015	United State	North America	Cross sectional	363	Yes	Yes	Yes	Yes	No	Mix
238	Wolff ER	2014	United State	North America	Cross sectional	99	Yes	Yes	Yes	Yes	No	Mix
239	Darden PM	2013	United State	North America	Cross sectional	98086	Yes	Yes	Yes	Yes	No	Mix
240	Gilkey MB	2013	United State	North America	Cross sectional	1847	Yes	Yes	Yes	Yes	No	Mix
241	Opel D.J. et al.,	2013	United State	North America	Cross sectional	437	No	No	No	Yes	Yes	Mix
242	Opel D.J. et al.,	2013	United State	North America	Cross sectional	237	No	No	Yes	Yes	Yes	Mix
243	Bazzano A	2012	United State	North America	Cross sectional	197	Yes	Yes	Yes	Yes	No	Mix
244	Freed GL	2010	United State	North America	Cross sectional	1552	Yes	Yes	Yes	Yes	No	Mix
245	Smith P.J. et al.,	2010	United State	North America	Cross sectional	2921	No	No	Yes	Yes	Yes	Mix
246	Salmon DA	2009	United State	North America	Cross sectional	963	Yes	Yes	Yes	Yes	No	Mix
247	Wu AC	2008	United State	North America	Cross sectional	228	Yes	Yes	Yes	Yes	No	Mix
248	Salmon DA	2005	United State	North America	Case control	1253	Yes	Yes	Yes	Yes	No	Mix
249	Bardenheier B	2004	United State	North America	Case control	2315	Yes	Yes	Yes	Yes	No	Mix
250	Xu, Y.	2021	United State	North America	Cross sectional	1803	No	No	Yes	Yes	Yes	MMR
251	Mensah-Bonsu, NE et al	2021	United State	North America	Cross sectional	89	Yes	No	No	Yes	Yes	MMR
252	Doll, MK et al	2021	United State	North America	Cohort study	34,471,357	No	Yes	No	No	Yes	MMR
253	Holroyd T.A. et al.,	2021	United State	North America	Cross sectional	37	No	Yes	Yes	Yes	Yes	MMR
254	Wharton-Michael P & Wharton-Clark A	2020	United State	North America	Cross sectional	20	No	No	Yes	No	No	MMR
255	Rodriguez-Nava G. et al.,	2020	United State	North America	Cross sectional	158602	No	No	No	Yes	No	MMR
256	Qian M. et al.,	2020	United State	North America	Cross sectional	271487	No	No	Yes	Yes	Yes	MMR
257	Mills, K et al	2020	United State	North America	Cross sectional	89	No	Yes	Yes	Yes	Yes	MMR
258	Christianson, B et al	2020	United State	North America	Cross sectional	300	No	No	Yes	Yes	Yes	MMR
259	Estep, K and Greenberg, P.	2020	United State	North America	Cross sectional	747	No	No	No	Yes	Yes	MMR
260	Nyathi S. et al.,	2019	United State	North America	Cross sectional	-	No	No	No	Yes	Yes	MMR
261	Moyer-Guse E. et al.,	2018	United State	North America	Cross sectional	187	No	No	Yes	Yes	No	MMR
262	Krishna A	2017	United State	North America	Cross sectional	435	Yes	Yes	Yes	Yes	No	MMR
263	Kang G.J. et al.,	2017	United State	North America	Cross sectional	50	No	No	Yes	Yes	Yes	MMR
264	Cacciatore MA	2016	United State	North America	Cross sectional	1000	Yes	Yes	Yes	Yes	No	MMR
265	Cataldi, JR et al	2016	United State	North America	Cross sectional	306	No	Yes	No	No	No	MMR
266	McNutt, LA	2016	United State	North America	Cross sectional	565	Yes	No	No	No	Yes	MMR
267	Lieu, TA et al	2015	United State	North America	Cross sectional	50233	No	Yes	Yes	Yes	No	MMR
268	Nyhan B, et al	2014	United State	North America	Cross sectional	4058	No	No	Yes	Yes	No	MMR
269	Gahr, P et al	2014	United State	North America	Cohort study	21	No	Yes	Yes	Yes	No	MMR
270	Gowda C	2013	United State	North America	Cross sectional	79	Yes	Yes	Yes	Yes	No	MMR

271	Downs, JS et al	2013	United state	North America	Cross sectional	30	No	Yes	Yes	Yes	No	MMR
272	Dempsey AF, et al.	2011	United State	North America	Cross sectional	748	No	No	No	Yes	No	MMR
273	Sugerman, DE et al	2010	United State	North America	Cohort study	839	No	No	Yes	Yes	No	MMR
274	Kennedy A.M & Gust D.A	2008	United State	North America	Cross sectional	12	Yes	Yes	Yes	Yes	Yes	MMR
275	Parker A.A. et al.,	2006	United State	North America	Cohort study	66	No	No	No	Yes	Yes	MMR
276	Kwan, B. M.	2022	United State	North America	RCT	824	No	No	Yes	Yes	No	Not stated
277	Boyle J	2020	United State	North America	Cross sectional	1029	Yes	Yes	Yes	Yes	No	Not stated
278	Buttenheim AM	2020	United State	North America	Cross sectional	257	Yes	Yes	Yes	Yes	No	Not stated
279	Dudley MZ	2020	United State	North America	Cross sectional	2196	Yes	Yes	Yes	Yes	No	Not stated
280	Finkelstein SR	2020	United State	North America	Cross sectional	300	Yes	Yes	Yes	Yes	No	Not stated
281	Mohanty S	2020	United State	North America	Cross sectional	140	Yes	Yes	Yes	Yes	No	Not stated
282	Buckman, C. et al	2020	United State	North America	Cross sectional	-	Yes	No	No	No	Yes	Not stated
283	Arora G	2019	United State	North America	Cross sectional	87	Yes	Yes	Yes	Yes	No	Not stated
284	Cataldi JR	2019	United State	North America	Cross sectional	295	Yes	Yes	Yes	Yes	No	Not stated
285	Cheng ER	2019	United State	North America	Cross sectional	1047	Yes	Yes	Yes	Yes	No	Not stated
286	Eller NM	2019	United State	North America	Cross sectional	488	Yes	Yes	Yes	Yes	No	Not stated
287	McCoy JD	2019	United State	North America	Cross sectional	14	Yes	Yes	Yes	Yes	No	Not stated
288	McDonald P	2019	United State	North America	Cross sectional	23	Yes	Yes	Yes	Yes	No	Not stated
289	Dubé	2019	Canada	North America	Cross sectional	2645	Yes	Yes	Yes	Yes	No	Not stated
290	Guay M	2019	Canada	North America	Cross sectional	8737	Yes	Yes	Yes	Yes	No	Not stated
291	Carrion ML	2018	United State	North America	Cross sectional	50	Yes	Yes	Yes	Yes	No	Not stated
292	Carrion ML	2018	United State	North America	Cross sectional	50	No	Yes	Yes	Yes	No	Not stated
293	Delamater PL	2018	United State	North America	Cohort study	4177	Yes	Yes	Yes	Yes	No	Not stated
294	Estep KA	2018	United State	North America	Cross sectional	77244	Yes	Yes	Yes	Yes	No	Not stated
295	Reich J A	2018	United State	North America	Cross sectional	28	Yes	Yes	Yes	Yes	No	Not stated
296	Amin AB	2017	United State	North America	Cross sectional	1417	Yes	Yes	Yes	Yes	No	Not stated
297	Chung Y	2017	United State	North America	Cross sectional	2603	Yes	Yes	Yes	Yes	No	Not stated
298	Henrikson NB	2017	United State	North America	Cohort study	391	Yes	Yes	Yes	Yes	No	Not stated
299	Kettunen C.	2017	United State	North America	Cross sectional	84	Yes	Yes	Yes	Yes	No	Not stated
300	Dubé E	2017	Canada	North America	Cross sectional	2013	Yes	Yes	Yes	Yes	No	Not stated
301	Greyson D	2017	Canada	North America	Cohort study	23	Yes	Yes	Yes	Yes	No	Not stated
302	Blaisdell LL	2016	United State	North America	Cross sectional	42	Yes	Yes	Yes	Yes	No	Not stated
303	Lee C	2016	United State	North America	Cross sectional	1253	Yes	Yes	Yes	Yes	No	Not stated
304	Wang E	2015	United State	North America	Cross sectional	23	Yes	Yes	Yes	Yes	No	Not stated
305	Dubé E	2015	Canada	North America	Cross sectional	54	Yes	Yes	Yes	Yes	No	Not stated
306	MacDonald SE	2014	Canada	North America	Cross sectional	247	Yes	Yes	Yes	Yes	No	Not stated
307	Glanz JM	2013	United State	North America	Cross sectional	443	Yes	Yes	Yes	Yes	No	Not stated
308	Mergler M.J	2013	United State	North America	Cross sectional	1367	Yes	Yes	Yes	Yes	No	Not stated
309	Gaudinoa JA	2012	United State	North America	Cohort study	1567	Yes	Yes	Yes	Yes	No	Not stated
310	Luthy KE	2012	United State	North America	Cross sectional	287	Yes	Yes	Yes	Yes	No	Not stated
311	Smith PJ	2011	United State	North America	Cross sectional	11206	Yes	Yes	Yes	Yes	No	Not stated
312	Luthy KE	2010	United State	North America	Cross sectional	86	Yes	Yes	Yes	Yes	No	Not stated
313	Gullion JS	2008	United State	North America	Cross sectional	25	Yes	Yes	Yes	Yes	No	Not stated
314	Gust D. A	2008	United State	North America	Cross sectional	3924	Yes	Yes	Yes	Yes	No	Not stated
315	Fredrickson DD	2004	United State	North America	Cross sectional	544	Yes	Yes	Yes	Yes	No	Not stated
316	Kulig JC	2002	Canada	North America	Cross sectional	47	Yes	Yes	Yes	Yes	No	Not stated

317	Goin-Kochel RP	2020	United State	North America	Cross sectional	225	Yes	Yes	Yes	Yes	No	Others
318	Sahni LC	2020	United State	North America	Cross sectional	338	Yes	Yes	Yes	Yes	No	Others
319	Gromis A & Liu K.Y	2020	United State	North America	Cross sectional	4763	No	No	No	No	Yes	Others
320	Dubé E	2016	Canada	North America	Cohort study	510	Yes	Yes	Yes	Yes	No	Others
321	Bardenheier B	2003	United State	North America	Cross sectional	648	Yes	Yes	Yes	Yes	No	Others
322	Fair E	2002	United State	North America	Cohort study	15	Yes	Yes	Yes	Yes	No	Others
323	Zhou T	2023	China	Asia	Cross sectional	2630	No	Yes	Yes	Yes	Yes	Covid 19
324	Zhang H.	2023	China	Asia	Cross sectional	346	No	Yes	No	Yes	Yes	Covid 19
325	Zhang K	2023	China	Asia	Cross sectional	437	No	Yes	Yes	Yes	No	Covid-19
326	Bourguiba, A	2023	UAE	Asia	Cross sectional	437	No	Yes	Yes	Yes	Yes	Covid-19
327	Alhuzaimi, AN	2023	Saudi Arabia	Asia	Cross sectional	873	No	No	Yes	Yes	Yes	Covid-19
328	Almuqbil, M.	2023	Saudi Arabia	Asia	Cross sectional	699	Yes	No	Yes	Yes	Yes	Covid-19
329	Ashour, HA	2023	Saudi Arabia	Asia	Cross sectional	293	No	Yes	Yes	Yes	Yes	Covid-19
330	Chawanpaiboon S.	2023	Thailand	Asia	Cross sectional	400	No	Yes	Yes	Yes	Yes	Covid-19
331	Dao, T.L. et al.,	2023	Vietnam	Asia	Cross sectional	602	No	No	Yes	Yes	No	Covid-19
332	Deng, JS et al	2023	Taiwan	Asia	Cross sectional	384	No	Yes	No	Yes	Yes	Covid-19
333	Ghazy, RM et al	2023	Egypt	Asia	Cross sectional	321	No	No	Yes	Yes	Yes	Covid-19
334	Maneesriwongul W. et al.,	2023	Thailand	Asia	Cross sectional	455	No	Yes	Yes	Yes	Yes	Covid-19
335	Khatrawi, EM and Sayed, AA	2023	Saudi Arabia	Asia	Cross sectional	344	No	No	Yes	Yes	Yes	Covid-19
336	Khoodoruth, MAS et al	2023	Qatar	Asia	Cross sectional	488	No	Yes	No	Yes	Yes	Covid-19
337	Zheng, Z	2023	China	Asia	Cross sectional	707	No	Yes	Yes	Yes	Yes	Covid-19
338	Tang, S.	2023	China	Asia	Cross sectional	2054	No	Yes	Yes	Yes	No	Covid-19
339	Zhou , Y	2023	China	Asia	Cross sectional	792	No	Yes	No	Yes	Yes	Covid-19
340	Zhou Y	2022	China	Asia	Cross sectional	3541	No	Yes	No	Yes	Yes	Covid-19
341	Tavakoli, N	2022	Iran	Asia	Cohort study	11042	No	Yes	Yes	Yes	No	Covid 19
342	Zhou, M	2022	China	Asia	Cross sectional	1602	No	Yes	Yes	Yes	No	Covid-19
343	Abdalla, SM	2022	Saudi Arabia	Asia	Cross sectional	420	No	Yes	Yes	Yes	No	Covid 19
344	Alhazza, SF	2022	Saudi Arabia	Asia	Cross sectional	1052	No	No	No	No	Yes	Covid-19
345	Ali M.	2022	Bangladesh	Asia	Cross sectional	396	No	Yes	Yes	Yes	No	Covid-19
346	Ali, M	2022	Bangladesh	Asia	Cross sectional	2633	Yes	Yes	Yes	Yes	No	Covid-19
347	Ali-Saleh, O	2022	Israel	Asia	Cross sectional	2483	No	Yes	Yes	Yes	No	Covid-19
348	Almalki OS	2022	Saudi Arabia	Asia	Cross sectional	4135	No	Yes	Yes	Yes	No	Covid-19
349	Chia MY	2022	Singapore	Asia	Cross sectional	1481	No	Yes	No	No	Yes	Covid-19
350	Choi, U. I.	2022	China	Asia	Cross sectional	1217	Yes	Yes	Yes	Yes	Yes	Covid-19
351	Duong, A.H	2022	Vietnam	Asia	Cross sectional	5357	No	No	Yes	Yes	Yes	Covid-19
352	Gunes, O	2022	Turkey	Asia	Cross sectional	369	No	No	Yes	Yes	No	Covid-19
353	Hou, Z.	2022	China	Asia	Cross sectional	3897	No	Yes	Yes	Yes	Yes	Covid-19
354	Huang, L.L	2022	China	Asia	Cross sectional	514	No	Yes	Yes	Yes	Yes	Covid-19
355	Khan, YH	2022	Saudi Arabia	Asia	Cross sectional	444	Yes	Yes	Yes	Yes	Yes	Covid-19
356	Kitro, A	2022	Thailand	Asia	Cross sectional	1064	No	Yes	Yes	Yes	No	Covid-19
357	Lee, M.	2022	South Korea	Asia	Cross sectional	113450	No	No	Yes	Yes	No	Covid-19
358	Li, JB	2022	China	Asia	Cross sectional	11141	No	Yes	Yes	Yes	No	Covid-19
359	Low, JM	2022	Singapore	Asia	Cross sectional	628	No	Yes	Yes	Yes	No	Covid-19
360	Lu, L	2022	China	Asia	Cross sectional	868	No	Yes	Yes	Yes	No	Covid-19
361	Ma, L.	2022	China	Asia	Cross sectional	9424	No	Yes	Yes	Yes	Yes	Covid-19
362	Ma, Y	2022	China	Asia	Cross sectional	424	No	Yes	Yes	Yes	Yes	Covid-19

363	Mohan, R.	2022	India	Asia	Cross sectional	204	No	Yes	Yes	Yes	Yes	Covid-19
364	Ng, DL	2022	Malaysia	Asia	Cross sectional	3528	Yes	Yes	Yes	Yes	Yes	Covid-19
365	Parinyarux, P.	2022	Thailand	Asia	Cross sectional	488	No	Yes	Yes	Yes	No	Covid-19
366	Qin C	2022	China	Asia	Cross sectional	1724	No	Yes	No	Yes	Yes	Covid-19
367	Rehman, T	2022	India	Asia	Cross sectional	1565	No	Yes	No	No	Yes	Covid-19
368	Samudyatha, UC	2022	India	Asia	Cross sectional	272	No	Yes	Yes	Yes	No	Covid-19
369	Shahani, R	2022	Pakistan	Asia	Cross sectional	454	No	Yes	Yes	Yes	Yes	Covid-19
370	Shwethashree, M	2022	India	Asia	Cross sectional	246	No	Yes	Yes	Yes	Yes	Covid-19
371	Tung, T. H.	2022	China	Asia	Cross sectional	1788	No	Yes	Yes	Yes	Yes	Covid-19
372	Wang, K	2022	China	Asia	Cross sectional	1299	No	Yes	Yes	Yes	No	Covid-19
373	Wang, L	2022	China	Asia	Cross sectional	2019	No	Yes	Yes	Yes	No	Covid-19
374	Wang, LJ	2022	Taiwan	Asia	Cross sectional	443	No	Yes	Yes	Yes	No	Covid-19
375	Wang, Q	2022	China	Asia	Cross sectional	5102	No	Yes	Yes	Yes	No	Covid-19
376	Wong, LP	2022	Malaysia	Asia	Cross sectional	1003	No	Yes	Yes	Yes	Yes	Covid-19
377	Wong, WH	2022	China	Asia	Cross sectional	545	No	Yes	Yes	Yes	No	Covid-19
378	Yang, J	2022	China	Asia	Cross sectional	12872	No	No	No	Yes	No	Covid-19
379	Zheng, M	2022	China	Asia	Cross sectional	2624	No	Yes	Yes	Yes	Yes	Covid-19
380	Zhou, X	2022	China	Asia	Cross sectional	2057	No	Yes	Yes	Yes	Yes	Covid-19
381	Khatatbeh, M	2022	Iraq, Jordan, Kuwait, Lebanon, Palestine, Qatar, Saudi Arabia (KSA), and the United Arab Emirates (UAE)	Asia	Cross sectional	3744	No	Yes	Yes	Yes	No	Covid-19
382	Huang, L.L	2022	China	Asia	Cross sectional	514	No	Yes	Yes	Yes	Yes	Covid-19
383	Abuhammad, S	2022	Jordan	Asia	Cross sectional	1078	No	Yes	Yes	Yes	Yes	Covid-19
384	Aedh, AI	2022	Saudi Arabia	Asia	Cross sectional	464	No	Yes	Yes	Yes	Yes	Covid-19
385	AlKetbi, LMB	2022	United Arab Emirates	Asia	Cross sectional	2510	No	Yes	Yes	Yes	No	Covid-19
386	Al-khlaiwi, T	2022	Saudi Arabia	Asia	Cross sectional	1304	No	Yes	Yes	Yes	Yes	Covid-19
387	Almansour, A	2022	Saudi Arabia	Asia	Cross sectional	500	No	Yes	Yes	Yes	Yes	Covid-19
388	Al-Qerem, W	2022	Jordan	Asia	Cross sectional	819	No	Yes	Yes	Yes	Yes	Covid-19
389	Alsulaiman, JW	2022	Jordan	Asia	Cross sectional	564	No	Yes	Yes	Yes	Yes	Covid-19
390	Bord, S	2022	Israel	Asia	Cross sectional	581	No	Yes	Yes	Yes	No	Covid-19
391	Elkhadry, S.W.	2022	Egypt	Asia	Cross sectional	173	No	Yes	Yes	Yes	Yes	Covid-19
392	ElSayed, D.A	2022	Egypt	Asia	Cross sectional	223	No	Yes	Yes	Yes	No	Covid-19
393	Ennaceur, S.	2022	Saudi Arabia	Asia	Cross sectional	379	No	Yes	Yes	Yes	Yes	Covid-19
394	Kharaba, Z	2022	United Arab Emirates	Asia	Cross sectional	1049	No	Yes	Yes	Yes	No	Covid-19
395	Mohammed, AH	2022	Iraq, Jordan, United Arab Emirates, Oman, and Yemen.	Asia	Cross sectional	1154	No	Yes	Yes	Yes	No	Covid-19

396	Morozov, N. G.	2022	Qatar	Asia	Cross sectional	1514	No	No	Yes	Yes	Yes	Covid-19
397	Reagu, S.	2022	Israel	Asia	Cross sectional	6882	No	Yes	Yes	Yes	Yes	Covid-19
398	Savitsky, B.	2022	Israel	Asia	Cross sectional	138	No	Yes	Yes	Yes	Yes	Covid-19
399	Shati, A.A.	2022	Saudi Arabia	Asia	Cross sectional	1463	No	Yes	Yes	Yes	No	Covid-19
400	Swed, S	2022	Syria	Asia	Cross sectional	283	No	Yes	Yes	Yes	No	Covid-19
401	Akgün O	2022	Turkey	Asia	Cross sectional	201	No	Yes	Yes	Yes	Yes	Covid-19
402	AL-Iede M	2022	Jordan	Asia	Cross sectional	2628	No	Yes	Yes	Yes	Yes	Covid-19
403	Al-Rasheedi AT	2022	Saudi Arabia	Asia	Cross sectional	597	No	No	No	No	Yes	Covid-19
404	Bas K	2022	Turkey	Asia	Cross sectional	950	No	No	No	Yes	Yes	Covid-19
405	Çağ Y	2022	Jordan	Asia	Cross sectional	1018	No	No	Yes	Yes	Yes	Covid-19
406	Lau, EY	2022	Hong Kong	Asia	Cross sectional	11141	No	Yes	Yes	Yes	No	Covid-19
407	Li, T	2022	China	Asia	Cross sectional	3342	No	Yes	Yes	Yes	No	Covid-19
408	Al-Qerem, W	2022	Iraq	Asia	Cross sectional	491	No	No	Yes	Yes	Yes	Covid-19
409	Kocamaz, EB	2022	Turkey	Asia	Cross sectional	384	No	Yes	Yes	Yes	No	Covid-19
410	Tsai, C-S. et al	2022	Taiwan	Asia	Cross sectional	252	No	Yes	No	Yes	No	Covid-19
411	Cho, HK. et at	2022	Taiwan	Asia	Cross sectional	1019	No	Yes	No	No	Yes	Covid-19
412	Li K. & Zhou F.	2022	China	Asia	Cross sectional	687	No	No	Yes	Yes	Yes	Covid-19
413	Aljamaan, F. et al.,	2022	Saudi Arabia	Asia	Cross sectional	1340	No	Yes	Yes	Yes	Yes	Covid-19
414	Zhang MX	2021	China	Asia	Cohort study	1788	Yes	Yes	Yes	Yes	No	Covid-19
415	Çelik, M.Y.	2021	Turkey	Asia	Cross sectional	274	No	No	Yes	Yes	Yes	Covid 19
416	Almusbah, Z	2021	Saudi Arabia	Asia	Cross sectional	1000	No	Yes	Yes	Yes	No	Covid-19
417	Altulahi, BA	2021	Saudi Arabia	Asia	Cross sectional	333	No	Yes	Yes	Yes	Yes	Covid-19
418	Atad, E	2021	Israel	Asia	Cross sectional	456	No	Yes	Yes	Yes	No	Covid-19
419	Padhi, BK	2021	India	Asia	Cross sectional	770	No	Yes	Yes	Yes	No	Covid-19
420	Choi SH	2021	South Korea	Asia	Cross sectional	226	No	Yes	Yes	Yes	No	Covid-19
421	Wan, X.	2021	China	Asia	Cross sectional	468	No	Yes	Yes	Yes	No	Covid-19
422	Xu, Y	2021	China	Asia	Cross sectional	4748	No	Yes	Yes	Yes	Yes	Covid-19
423	Yigit, M	2021	Turkey	Asia	Cross sectional	428	Yes	Yes	Yes	Yes	No	Covid-19
424	Yilmaz, M.	2021	Turkey	Asia	Cross sectional	1035	No	Yes	Yes	Yes	Yes	Covid-19
425	Zhou, Y	2021	China	Asia	Cross sectional	1071	No	Yes	Yes	Yes	No	Covid-19
426	Musa, S.	2021	Qatar	Asia	Cross sectional	4023	No	Yes	Yes	Yes	No	Covid-19
427	Aldakhil, H.	2021	Saudi Arabia	Asia	Cross sectional	270	No	No	Yes	Yes	Yes	Covid-19
428	Samannodi, M.	2021	Saudi Arabia	Asia	Cross sectional	581	No	Yes	Yes	Yes	Yes	Covid-19
429	Shmueli, L.	2021	Israel	Asia	Cross sectional	1012	No	No	Yes	Yes	No	Covid-19
430	Gendler Y	2021	Israel	Asia	Cross sectional	520	No	No	Yes	No	No	Covid-19
431	Wang, Z.	2021	China	Asia	Cross sectional	1332	No	Yes	No	Yes	Yes	Covid-19
432	Altulahi, N.	2021	Saudi Arabia	Asia	Cross sectional	3038	No	No	No	Yes	No	Covid-19
433	Feng, H	2021	China	Asia	Cross sectional	3703	No	No	No	Yes	Yes	Covid-19
434	Wang, X.	2021	China	Asia	Cross sectional	941	No	Yes	No	No	Yes	Covid-19
435	Yoda, T. et al.,	2021	Japan	Asia	Cross sectional	1100	No	No	Yes	Yes	Yes	Covid-19
436	Horiuchi, S. et al.,	2021	Japan	Asia	Cross sectional	1200	No	No	Yes	Yes	Yes	Covid-19
437	Lin Y et al	2021	China	Asia	Cross sectional	2026	No	No	Yes	Yes	Yes	Covid-19
438	Lu X	2021	China	Asia	Cross sectional	13451	No	No	Yes	Yes	Yes	Covid-19
439	Al-Qahtani, AM	2020	Saudi Arabia	Asia	Cross sectional	528	Yes	Yes	Yes	Yes	Yes	Covid-19
440	Zhang, KC	2020	China	Asia	Cross sectional	1052	No	Yes	Yes	Yes	No	Covid-19
441	Xie, H.	2023	China	Asia	Cross sectional	1431	No	Yes	Yes	No	Yes	HPV

442	Babi A.	2023	Kazakhstan	Asia	Cross sectional	141	No	Yes	Yes	Yes	No	HPV
443	Choi, J.	2023	Korea	Asia	Cross sectional	10	No	Yes	Yes	Yes	Yes	HPV
444	Frianto, D.	2022	Indonesia	Asia	Cross sectional	286	Yes	Yes	Yes	Yes	Yes	HPV
445	Pearl, CA	2022	India	Asia	Cross sectional	45	No	Yes	Yes	Yes	No	HPV
446	Tubas F	2022	Turkey	Asia	Cross sectional	200	No	Yes	Yes	Yes	Yes	HPV
447	Zhang, Z	2022	China	Asia	Cross sectional	5215	No	Yes	No	No	No	HPV
448	Akca, G	2022	Turkey	Asia	Cross sectional	330	No	Yes	Yes	Yes	No	HPV
449	Huang, Z	2022	China	Asia	Cross sectional	1021	No	Yes	Yes	Yes	Yes	HPV
450	Zach, R	2022	Israel	Asia	Cross sectional	10	Yes	Yes	Yes	Yes	No	HPV
451	Nguyen, LH	2022	Vietnam	Asia	Cross sectional	785	No	No	Yes	Yes	No	HPV
452	Imanishi Y	2022	Japan	Asia	Cross sectional	60	No	Yes	Yes	No	No	HPV
453	Yagi	2022	Japan	Asia	Cross sectional	1576	No	Yes	No	No	No	HPV
454	Suzuki	2022	Japan	Asia	RCT	2175	No	Yes	Yes	Yes	No	HPV
455	Alkalash, SH	2022	Saudi Arabia	Asia	Cross sectional	343	No	Yes	Yes	Yes	Yes	HPV
456	Choi. J	2021	South Korea	Asia	Cross sectional	906	No	Yes	Yes	No	No	HPV
457	Shuto	2021	Japan	Asia	Cross sectional	1646	No	No	Yes	Yes	Yes	HPV
458	Ugumori	2021	Japan	Asia	Cross sectional	59	No	No	Yes	No	No	HPV
459	Huang Y	2021	China	Asia	Cross sectional	1125	Yes	Yes	Yes	Yes	No	HPV
460	Kobayashi	2021	Japan	Asia	Cross sectional	242	No	Yes	Yes	Yes	Yes	HPV
461	Miyoshi	2020	Japan	Asia	Cross sectional	1648	No	Yes	Yes	Yes	Yes	HPV
462	Egawa-Takata	2020	Japan	Asia	Cross sectional	450	No	Yes	Yes	Yes	Yes	HPV
463	Degarege A	2018	India	Asia	Cross sectional	1609	Yes	Yes	Yes	Yes	No	HPV
464	Yuen WW	2018	China	Asia	Cross-sectional	1160	Yes	Yes	Yes	Yes	No	HPV
465	Yagi A	2018	Japan	Asia	Cross sectional	4260	No	Yes	Yes	No	No	HPV
466	Shida	2015	Japan	Asia	Cross sectional	125	No	Yes	Yes	Yes	No	HPV
467	Egawa-Takata	2015	Japan	Asia	Cross sectional	2828	No	Yes	No	No	Yes	HPV
468	Hanley	2014	Japan	Asia	Cross sectional	27	No	Yes	Yes	No	Yes	HPV
469	Hanley S	2012	Japan	Asia	Cross sectional	862	No	No	No	Yes	Yes	HPV
470	Al-Qerem, W	2023	Jordon	Asia	Cross sectional	667	No	Yes	Yes	Yes	Yes	Influenza
471	Alharbi, I.	2023	Saudi Arabia	Asia	Cross sectional	334	No	Yes	Yes	Yes	Yes	Influenza
472	Fan, J.	2022	China	Asia	Cross sectional	5016	No	Yes	Yes	Yes	Yes	Influenza
473	Lai, X	2022	China	Asia	Cross sectional	6668	No	Yes	Yes	Yes	Yes	Influenza
474	Liao, Q	2022	China	Asia	Cross sectional	291	No	Yes	Yes	Yes	No	Influenza
475	Zhang, H	2022	China	Asia	Cross sectional	7323	No	Yes	Yes	Yes	Yes	Influenza
476	Abed Elhadi Shahbari N	2022	Israel	Asia	Cross sectional	693	Yes	No	Yes	Yes	No	Influenza
477	Alenazi, K. A	2022	Saudi Arabia	Asia	Cross sectional	539	No	Yes	Yes	Yes	No	Influenza
478	AlOmran, HA	2022	Saudi Arabia	Asia	Cross sectional	510	No	Yes	Yes	Yes	Yes	Influenza
479	Hussein, YH	2022	Egypt	Asia	Cross sectional	287	No	Yes	Yes	Yes	No	Influenza
480	Jiang, M	2022	China	Asia	Cross sectional	1206	No	Yes	Yes	Yes	No	Influenza
481	Tsang, TK	2022	China	Asia	RCT	829	No	No	No	No	No	Influenza
482	Zakhour R	2021	Lubnan	Asia	Cross sectional	306	Yes	Yes	Yes	Yes	Yes	Influenza
483	Salawati, E.	2021	Saudi Arabia	Asia	Cross sectional	2501	No	Yes	Yes	Yes	No	Influenza
484	Alolayan A	2019	Saudi Arabia	Asia	Cross sectional	399	Yes	Yes	Yes	Yes	No	Influenza
485	Alabbad AA	2018	Saudi Arabia	Asia	Cross sectional	100	Yes	Yes	Yes	Yes	No	Influenza
486	Buyuktiryaki B	2014	Turkey	Asia	Cross sectional	625	Yes	Yes	Yes	Yes	No	Influenza
487	Akis S	2011	Turkey	Asia	Cross sectional	611	Yes	Yes	Yes	Yes	No	Influenza

488	Ahmed, N	2023	Bangladesh	Asia	Cross sectional	244	No	Yes	Yes	No	No	Mix
489	Sahoo S. S.	2023	India	Asia	Cross sectional	196	No	No	Yes	Yes	Yes	Mix
490	Mishra, K.	2023	India	Asia	Cross sectional	106	No	No	Yes	Yes	Yes	Mix
491	Du, M.	2022	China	Asia	Cross sectional	3011	No	Yes	Yes	Yes	No	Mix
492	Han, K.	2022	China	Asia	Cross sectional	2081	No	No	Yes	Yes	Yes	Mix
493	Ji, M	2022	China	Asia	Cohort study	972	No	No	No	No	Yes	Mix
494	Khaliq, A	2022	Pakistan	Asia	Cross sectional	230	No	Yes	Yes	Yes	Yes	Mix
495	Shaipuzaman, N. A	2022	Malaysia	Asia	Cross sectional	97	No	No	Yes	Yes	No	Mix
496	Sinuraya, R. K.	2022	Indonesia	Asia	Cross sectional	276	No	Yes	Yes	Yes	No	Mix
497	Summan, A.	2022	India	Asia	Cross sectional	59144	No	No	Yes	Yes	No	Mix
498	Tianshuo Z	2022	China	Asia	Cross-sectional	199	No	Yes	No	Yes	No	Mix
499	Wachinger, J	2022	Philipine	Asia	Cross sectional	45	No	Yes	Yes	Yes	Yes	Mix
500	Wang, Q	2022	China	Asia	Cross sectional	802	No	No	Yes	Yes	No	Mix
501	Wu, L	2022	China	Asia	Cross sectional	1691	No	Yes	Yes	Yes	Yes	Mix
502	Yalcin, SS	2022	Turkey	Asia	Cross sectional	23756	No	Yes	Yes	Yes	No	Mix
503	Akbulut, S	2022	Turkey	Asia	Cross sectional	460	No	Yes	Yes	Yes	No	Mix
504	Alghamdi, S	2022	Saudi Arabia	Asia	Cross sectional	123	No	No	Yes	Yes	Yes	Mix
505	Al-Regaiey, KA	2022	Saudi Arabia	Asia	Cross sectional	325	No	Yes	Yes	Yes	Yes	Mix
506	Hijazi, R	2022	Israel	Asia	Cross sectional	18	No	Yes	Yes	Yes	No	Mix
507	Ozer, M	2022	Turkey	Asia	Cross sectional	78	No	Yes	Yes	Yes	No	Mix
508	Topaktas B.	2022	Turkey	Asia	Cross sectional	338	No	Yes	No	Yes	No	Mix
509	Al Yamani, Z.J. et al.,	2022	Saudi Arabia	Asia	Cross sectional	375	Yes	No	Yes	No	No	Mix
510	Zin ZM et al	2022	Malaysia	Asia	Cross sectional	27	Yes	No	No	Yes	Yes	Mix
511	Temsah, MH	2021	Saudi Arabia	Asia	Cross sectional	3167	No	Yes	Yes	Yes	No	Mix
512	Wang, Q	2021	China	Asia	Cross sectional	5102	No	Yes	Yes	Yes	No	Mix
513	Yilmazbaz, P	2021	Turkey	Asia	Cross sectional	440	No	Yes	Yes	Yes	No	Mix
514	Tal, O	2021	Israel	Asia	Cross sectional	1010	No	Yes	No	No	Yes	Mix
515	Al-Nafeesah AS	2021	Saudi Arabia	Asia	Cross sectional	1143	No	Yes	Yes	Yes	Yes	Mix
516	Baghdadi, LR	2021	Saudi Arabia	Asia	Cross sectional	577	No	No	Yes	Yes	Yes	Mix
517	Hou, Z.	2021	China	Asia	Cross sectional	1655	No	Yes	No	Yes	No	Mix
518	Alsubaie SS	2019	Saudi Arabia	Asia	Cross sectional	500	Yes	Yes	Yes	Yes	No	Mix
519	Chang K	2019	South Korea	Asia	Cross sectional	129	Yes	Yes	Yes	Yes	No	Mix
520	Jalloh M.F.	2019	Bangladesh	Asia	Cross sectional	105	Yes	Yes	Yes	Yes	No	Mix
521	Amit Aharon A	2018	Israel	Asia	Cross sectional	200	Yes	Yes	Yes	Yes	No	Mix
522	Chan HK	2018	Malaysia	Asia	Cross sectional	117429	Yes	Yes	Yes	Yes	No	Mix
523	Dasgupta P	2018	India	Asia	Cross sectional	194	Yes	Yes	Yes	Yes	No	Mix
524	Khaliq A.	2017	Pakistan	Asia	Cross sectional	484	Yes	Yes	Yes	Yes	No	Mix
525	Abu-rish EY	2016	Jordan	Asia	Cross sectional	568	No	No	No	No	Yes	Mix
526	M Muhsen K	2012	Israel	Asia	Case control	270	Yes	Yes	Yes	Yes	No	Mix
527	Quaiyum MA	2011	Bangladesh	Asia	Cross sectional	2700	Yes	Yes	Yes	Yes	No	Mix
528	Hussein SZ	2022	Malaysia	Asia	Cross sectional	202	No	Yes	Yes	Yes	No	MMR
529	Ashkenazi S	2020	Israel	Asia	Cross sectional	399	Yes	Yes	Yes	Yes	No	MMR
530	Abdullah AC	2018	Malaysia	Asia	Cross sectional	760	Yes	Yes	Yes	Yes	No	MMR
531	Fakhruddin TM et al	2023	Malaysia	Asia	Cross sectional	375	No	Yes	Yes	Yes	No	Not stated
532	Han Y	2022	China	Asia	Cross sectional	2952	No	Yes	Yes	No	No	Not stated
533	Kuan, CI	2022	Taiwan	Asia	Cross sectional	24	No	No	Yes	Yes	Yes	Not stated

534	Shen, X	2022	China	Asia	Cross sectional	1025	No	Yes	Yes	Yes	No	Not stated
535	Alaamri, O.	2022	Saudi Arabia	Asia	Cross sectional	2030	No	Yes	Yes	Yes	Yes	Not stated
536	Yörük S	2021	Turkey	Asia	Cross sectional	370	Yes	Yes	Yes	Yes	No	Not stated
537	Akhmetzhanova Z	2020	Kazakhstan	Asia	Cross sectional	384	Yes	Yes	Yes	Yes	No	Not stated
538	AlGoraini YM	2020	Saudi Arabia	Asia	Cross sectional	384	Yes	Yes	Yes	Yes	No	Not stated
539	Alsuwaidi AR	2020	United Arab Emirates	Asia	Cross sectional	300	Yes	Yes	Yes	Yes	No	Not stated
540	Çağ Y	2020	Turkey	Asia	Cross sectional	1184	Yes	Yes	Yes	Yes	No	Not stated
541	Gunes NA	2020	Turkey	Asia	Cross sectional	614	Yes	Yes	Yes	Yes	No	Not stated
542	Kalok A.	2020	Malaysia	Asia	Cross sectional	1081	Yes	Yes	Yes	Yes	No	Not stated
543	Noyman-Veksler G	2020	Israel	Asia	Cross sectional	555	Yes	Yes	Yes	Yes	No	Not stated
544	Yalçin SS	2020	Turkey	Asia	Cross sectional	14	Yes	Yes	Yes	Yes	No	Not stated
545	Rumetta J	2020	Malaysia	Asia	Cross sectional	14	Yes	Yes	Yes	Yes	Yes	Not stated
546	Hu Y	2019	China	Asia	Cross sectional	770	Yes	Yes	Yes	Yes	No	Not stated
547	Ren J	2018	China	Asia	Cross sectional	1188	Yes	Yes	Yes	Yes	No	Not stated
548	Sun X	2018	China	Asia	Cross sectional	34	Yes	Yes	Yes	Yes	No	Not stated
549	Noor T	2018	Pakistan	Asia	Cross sectional	100	Yes	Yes	Yes	Yes	No	Not stated
550	Aharony N	2017	Israel	Asia	Cross sectional	210	Yes	Yes	Yes	Yes	No	Not stated
551	Azizi FS	2017	Malaysia	Asia	Cross sectional	545	Yes	Yes	Yes	Yes	No	Not stated
552	Ni, Y-H.	2023	China	Asia	Cross sectional	1051	No	No	No	Yes	Yes	Others
553	Yang, Y	2022	China	Asia	Cross sectional	1085	No	Yes	Yes	Yes	Yes	Others
554	Al-Iede, M	2022	Jordan	Asia	Cross sectional	720	No	Yes	Yes	Yes	Yes	Others
555	Du, Y et al	2022	China	Asia	Cross sectional	1110	No	No	Yes	Yes	No	Others
556	Yang, Y	2022	China	Asia	Cross sectional	219	No	Yes	Yes	Yes	Yes	Other
557	Wang, M	2022	China	Asia	Cross sectional	762	No	Yes	Yes	Yes	No	Other
558	Qi L	2019	China	Asia	Cross sectional	992	Yes	Yes	Yes	Yes	No	Others
559	Gesser-Edelsburga A	2016	Israel	Asia	Cross sectional	196	Yes	Yes	Yes	Yes	No	Others
560	Khan TM	2016	Pakistan	Asia	Cross sectional	1775	Yes	Yes	Yes	Yes	No	Other
561	Khowaja AR	2012	Pakistan	Asia	Cross sectional	1017	Yes	Yes	Yes	Yes	No	Others
562	Ali HYM	2004	Iraq	Asia	Cross sectional	254	Yes	Yes	Yes	Yes	No	Others
563	Li L	2022	China	Asia	Cross sectional	1409	No	Yes	No	Yes	Yes	Others
564	Sharif Nia et al.,	2023	Australia, Iran, China, Turkey	Mix	Cross sectional	673	No	No	No	No	Yes	Covid-19
565	Goldman, RD	2022	United State, Canada, Israel, Japan, Spain, Switzerland	Mix	Cross sectional	2687	No	No	No	No	Yes	Covid-19
566	Goldman, RD	2022	USA, Canada, Israel, and Switzerland	Mix	Cohort study	882	No	No	No	No	Yes	Covid-19
567	Goldman, RD	2022	United State, Canada, Israel	Mix	Cross sectional	1956	No	No	Yes	Yes	No	Covid-19
568	Sabra, HK	2022	Egypt	Mix	Cross sectional	1032	No	Yes	Yes	Yes	No	Covid-19
569	Skjefte, M.	2021	United States, India, Brazil, Russia, Spain, Argentina,	Mix	Cross sectional	17871	No	Yes	Yes	Yes	Yes	Covid-19

			Colombia, UK, Mexico, Peru, South Africa, Italy, Chile, Philippines, Australia and New Zealand									
570	Goldman, RD	2021	United State, Canada, Israel	Mix	Cross sectional	2769	No	Yes	Yes	Yes	No	Covid-19
571	Urrunaga-Pastor, D.	2021	Latin America and the Caribbean	Mix	Cross sectional	227740	No	Yes	Yes	Yes	No	Covid-19
572	Goldman RD	2020	United State, Canada, Japan, Spain, Switzeland	Mix	Cross sectional	1005	No	Yes	Yes	Yes	Yes	Covid-19
573	Yilmaz M	2023	Turkey	Mix	Cross sectional	94	Yes	No	Yes	Yes	Yes	Mix
574	Tekin C.	2023	Turkey	Mix	Cross sectional	1100	No	No	Yes	Yes	Yes	Mix
575	Borras-Bermejo B	2022	Afghanistan, Democratic Republic of the Congo, Mauritania, Niger, Pakistan and South Sudan.	Mix	Cross sectional	2706	No	Yes	Yes	Yes	No	Mix
576	Durmaz, N.	2022	Turkey	Mix	Cross sectional	1087	No	No	Yes	Yes	Yes	Mix
577	Tan L.	2023	USA,UK, France, Germany, Italy, Brazil, Argentina, and Australia;	Mix	Cross sectional	4962	No	No	Yes	Yes	Yes	Others
578	Reuben R	2020	United States, Canada, England	Mix	Cross sectional	484	Yes	Yes	Yes	Yes	No	Not stated
579	Bolsewicz, KT et al	2023	Australia	Oceania	Cross sectional	21	No	No	Yes	Yes	Yes	Covid-19
580	Wen, LM	2022	Australia	Oceania	Cross sectional	491	No	Yes	Yes	Yes	No	Covid-19
581	Jeffs, E	2021	New Zealand	Oceania	Cross sectional	1191	No	Yes	Yes	Yes	No	Covid-19
582	Evans, S	2021	Australia	Oceania	Cross sectional	1094	No	Yes	Yes	Yes	No	Covid-19
583	Carlsona, SJ	2022	Australia	Oceania	Cohort study	87	No	Yes	Yes	Yes	No	Influenza
584	Biezen	2018	Australia	Oceania	Cross sectional	50	Yes	Yes	Yes	Yes	No	Influenza
585	Jones K	1992	Australia	Oceania	Cross sectional	171	Yes	Yes	Yes	Yes	No	Influenza
586	Wright, D	2022	Australia	Oceania	Cross sectional	1050	No	Yes	Yes	Yes	Yes	Mix
587	Debela, M. S.	2022	New Zealand	Oceania	Cross sectional	178	No	Yes	Yes	Yes	No	Mix
588	Enkel SL	2018	Australia	Oceania	Cross sectional	10	Yes	Yes	Yes	Yes	No	Mix
589	Forbes TA	2015	Australia	Oceania	Cross sectional	171	Yes	Yes	Yes	Yes	No	Mix

590	Armiento R	2020	Australia	Oceania	Cohort study	759	Yes	Yes	Yes	Yes	No	Not stated
591	Tuckerman J	2020	Australia	Oceania	Cross sectional	539	Yes	Yes	Yes	Yes	No	Not stated
592	Bryden GM	2019	Australia	Oceania	Cross sectional	897	Yes	Yes	Yes	Yes	No	Not stated
593	Helps C	2019	Australia	Oceania	Cross sectional	32	Yes	Yes	Yes	Yes	No	Not stated
594	Rossen I	2019	Australia	Oceania	Cross sectional	296	Yes	Yes	Yes	Yes	No	Not stated
595	Rozbroj T	2019	Australia	Oceania	Cross sectional	904	Yes	Yes	Yes	Yes	No	Not stated
596	Attwell K	2018	Australia	Oceania	Cross sectional	32	Yes	Yes	Yes	Yes	No	Not stated
597	Attwell K	2018	Australia	Oceania	Cross sectional	29	Yes	Yes	Yes	Yes	No	Not stated
598	Corben P	2018	Australia	Oceania	Cross sectional	231	Yes	Yes	Yes	Yes	No	Not stated
599	Frawley J.E	2018	Australia	Oceania	Cross sectional	429	Yes	Yes	Yes	Yes	No	Not stated
600	Attwell K	2017	Australia	Oceania	Cross sectional	27	Yes	Yes	Yes	Yes	No	Not stated
601	Attwell K	2015	Australia	Oceania	Cross sectional	304	Yes	Yes	Yes	Yes	No	Not stated
602	Gilmartin CE	2020	Australia	Oceania	Cross sectional	113	Yes	Yes	Yes	Yes	No	Others
603	Ates, BO	2023	Turkiye	Europe	Cross sectional	284	No	No	Yes	Yes	No	Covid-19
604	Bektas, I and Bektas, M	2023	Turkey	Europe	Cross sectional	199	No	No	Yes	Yes	No	Covid-19
605	Celik, T and Dogan, D	2023	Turkey	Europe	Cross sectional	102	No	No	No	Yes	Yes	Covid-19
606	Sasic M. et al.,	2023	Croatia	Europe	Cross sectional	872	No	No	No	No	Yes	Covid-19
607	Sahin, A. et al.,	2023	Turkey	Europe	Cross sectional	396	No	No	Yes	Yes	Yes	Covid-19
608	Esposito, S. et al	2023	Italy	Europe	Cross sectional	3433	No	Yes	Yes	Yes	Yes	Covid-19
609	Ganem, F et al	2023	Spain	Europe	Cross sectional	3383	No	No	Yes	Yes	Yes	Covid-19
610	Smith LE	2022	United Kingdom	Europe	Cohort study	270	No	No	No	Yes	Yes	Covid -19
611	Ko ́zlarek, M.	2022	Poland	Europe	Cross sectional	402	No	No	Yes	Yes	No	Covid-19
612	Bianco, A.	2022	Italy	Europe	Cross sectional	394	No	Yes	Yes	Yes	No	Covid-19
613	Ceannt, R	2022	Ireland	Europe	Cohort study	482	No	No	Yes	Yes	No	Covid-19
614	Di Giuseppe, G.	2022	Italy	Europe	Cross sectional	607	No	Yes	Yes	Yes	Yes	Covid-19
615	Iannello, P.	2022	Italy	Europe	Cross sectional	415	No	Yes	Yes	Yes	Yes	Covid-19
616	Krakowczyk, J.B.	2022	German	Europe	Cross sectional	2405	No	Yes	Yes	Yes	Yes	Covid-19
617	Lecce, M	2022	Italy	Europe	Cross sectional	604	No	Yes	Yes	Yes	No	Covid-19
618	Manolescu, L.S.C.	2022	Romania	Europe	Cross sectional	1645	No	Yes	Yes	Yes	No	Covid-19
619	Marcau, F.C.	2022	Romania	Europe	Cross sectional	581	No	No	Yes	Yes	Yes	Covid-19
620	Miliordos, K	2022	Greece	Europe	Cross sectional	366	No	Yes	Yes	Yes	Yes	Covid-19
621	Miraglia del Giudice, G	2022	Italy	Europe	Cross sectional	430	No	Yes	Yes	Yes	No	Covid-19
622	Napoli, A.	2022	Italy	Europe	Cross sectional	358	No	Yes	Yes	Yes	No	Covid-19
623	Rees, F	2022	German	Europe	Cross sectional	244	No	No	No	No	No	Covid-19
624	Steletou, E	2022	Greece	Europe	Cross sectional	439	No	Yes	Yes	Yes	Yes	Covid-19
625	Schmidtke, K. A	2022	England	Europe	Cross sectional	954	No	Yes	Yes	Yes	No	Covid-19
626	Skirrow, H.	2022	England	Europe	Cross sectional	1404	No	No	Yes	Yes	No	Covid-19
627	Wagner, A	2022	Switzerland.	Europe	Cross sectional	1318	No	Yes	Yes	Yes	No	Covid-19
628	Savarese G.	2022	Italy	Europe	Cross sectional	1105	No	No	Yes	Yes	Yes	Covid-19
629	Babicki, M	2021	Poland	Europe	Cross sectional	4432	No	Yes	No	Yes	Yes	Covid-19
630	Brandstetter, S	2021	German	Europe	Cross sectional	612	No	Yes	Yes	Yes	No	Covid-19
631	Fedele	2021	Italy	Europe	Cross sectional	640	Yes	Yes	Yes	Yes	No	Covid-19
632	Galanis, P	2021	Greece	Europe	Cross sectional	656	No	Yes	Yes	Yes	Yes	Covid-19
633	Galanis, P	2021	Greece	Europe	Cross sectional	813	No	Yes	Yes	Yes	No	Covid-19
634	Montalti, M.	2021	Italy	Europe	Cohort study	4993	No	Yes	Yes	Yes	No	Covid-19

635	Seiler, M.	2021	Switzerland	Europe	Cross sectional	662	No	No	Yes	Yes	Yes	Covid-19
636	Zona, S. et al.,	2021	Italy	Europe	Cross sectional	1799	No	Yes	Yes	No	No	Covid-19
637	Russo, L. et al.,	2021	Italy	Europe	Cross sectional	1205	No	No	No	Yes	Yes	Covid-19
638	Bell S	2020	England	Europe	Cross sectional	1252	No	Yes	No	Yes	No	Covid-19
639	Smith, L.E.	2019	England	Europe	Cohort study	270	No	No	No	Yes	Yes	Covid-19
640	Sobierajski, T.	2023	Poland	Europe	Cross sectional	360	No	Yes	Yes	Yes	No	HPV
641	Sˇtrbac M. et al.,	2023	Serbia	Europe	Cross sectional	436	No	Yes	Yes	Yes	Yes	HPV
642	Abuduxike G	2022	Cyprus	Europe	Cross sectional	227	No	Yes	Yes	No	Yes	HPV
643	Calagna G	2022	Italy	Europe	Cross sectional	300	No	Yes	Yes	No	No	HPV
644	Lopez, N.	2022	Spain	Europe	Cross sectional	1405	No	No	Yes	Yes	No	HPV
645	Karafillakis, E	2022	France	Europe	Cross sectional	57	No	Yes	Yes	Yes	No	HPV
646	Smolarczyk, K.	2022	Poland	Europe	Cross sectional	288	No	Yes	Yes	Yes	Yes	HPV
647	Taylor, J	2022	England	Europe	Cross sectional	138	No	Yes	Yes	Yes	No	HPV
648	Venderbos, J. R.	2022	Netherlands	Europe	Cross sectional	16	Yes	Yes	Yes	Yes	No	HPV
649	Naoum, P	2022	Greece	Europe	Cross sectional	1000	No	Yes	Yes	Yes	No	HPV
650	Lopez, N.	2022	Spain	Europe	Cross sectional	1405	No	No	Yes	Yes	No	HPV
651	Runngren, E.	2021	Sweden	Europe	Cross sectional	20	No	Yes	Yes	Yes	Yes	HPV
652	Della Polla G	2020	Italy	Europe	Cross sectional	435	Yes	Yes	Yes	Yes	No	HPV
653	Waller J	2020	England & Wales	Europe	Cross sectional	1056	Yes	Yes	Yes	Yes	No	HPV
654	Amdisen L	2018	Denmark	Europe	Cohort study	161528	Yes	Yes	Yes	Yes	No	HPV
655	Grandahl M	2017	Sweden	Europe	Cross sectional	366	Yes	Yes	Yes	Yes	No	HPV
656	Navarro-Illana P	2015	Spain	Europe	Cross sectional	833	Yes	Yes	Yes	Yes	No	HPV
657	Grandahl M	2014	Sweden	Europe	Cross sectional	25	Yes	Yes	Yes	Yes	No	HPV
658	Kornfeld	2013	Spain	Europe	Cross sectional	224	Yes	Yes	Yes	Yes	No	HPV
659	Craciun C	2012	Romania	Europe	Cross sectional	25	Yes	Yes	Yes	Yes	No	HPV
660	Hontelez J.A.C.	2010	Netherlands	Europe	Cross sectional	203	Yes	Yes	Yes	Yes	No	HPV
661	Kiroplis et al.,	2023	Ireland	Europe	Cross sectional	183	No	Yes	Yes	Yes	Yes	Influenza
662	Price, T	2022	England	Europe	Cross sectional	12	No	Yes	Yes	Yes	No	Influenza
663	De Gioia, E.R.	2022	Italy	Europe	Cross sectional	169	No	Yes	Yes	Yes	Yes	Influenza
664	Di Giuseppe, G.	2022	Italy	Europe	Cross sectional	574	No	Yes	Yes	No	No	Influenza
665	Bielecki K	2020	Scotland	Europe	Cross sectional	65	Yes	Yes	Yes	Yes	No	Influenza
666	Gorman D.R	2020	Poland	Europe	Cross sectional	128	Yes	Yes	Yes	Yes	No	Influenza
667	Prospero E	2019	Italy	Europe	Cross sectional	366	Yes	Yes	Yes	Yes	No	Influenza
668	Rodríguez-Blanco N	2019	Spain	Europe	Cross sectional	683	Yes	Yes	Yes	Yes	No	Influenza
669	Paterson P	2018	England	Europe	Cross sectional	913	Yes	Yes	Yes	Yes	No	Influenza
670	Bults M	2011	Netherlands	Europe	Cross sectional	1227	Yes	Yes	Yes	Yes	No	Influenza
671	Brown K.F	2010	England	Europe	Cross sectional	142	Yes	Yes	Yes	Yes	No	Influenza
672	Gjini, E.	2023	Albania	Europe	Cross sectional	475	No	No	Yes	Yes	Yes	Mix
673	Duran, S et al	2023	Turkey	Europe	Cross sectional	610	No	No	Yes	Yes	Yes	Mix
674	Marron, L. et al	2023	Ireland	Europe	Cross sectional	855	No	No	Yes	Yes	Yes	Mix
675	Buonsenso, D	2022	Italy	Europe	Cross sectional	121	No	Yes	No	No	No	Mix
676	Deml, MJ	2022	Switzerland	Europe	Cross sectional	30	No	Yes	Yes	Yes	Yes	Mix
677	Derdemezis, C.	2022	Greece	Europe	Cross sectional	1095	No	Yes	Yes	Yes	No	Mix
678	Ebi, SJ	2022	Switzerland	Europe	Cross sectional	1390	No	Yes	Yes	Yes	Yes	Mix
679	Fakonti, G	2022	Greece	Europe	Cross sectional	1885	No	Yes	Yes	Yes	Yes	Mix

680	Herdea, V	2022	Romania	Europe	Cohort study	5640	No	Yes	Yes	Yes	No	Mix
681	Jafflin, K	2022	Switzerland	Europe	Cross sectional	2425	No	No	Yes	No	No	Mix
682	Miron, V. D.	2022	Romania	Europe	Cross sectional	2550	No	Yes	Yes	Yes	Yes	Mix
683	Napolitano, F	2022	Italy	Europe	Cross sectional	444	No	No	Yes	Yes	Yes	Mix
684	Nurmi, J	2022	Finland	Europe	Cross sectional	38	No	Yes	Yes	Yes	Yes	Mix
685	Skitareli ́c, N.	2022	Croatia	Europe	Cross sectional	300	No	Yes	Yes	Yes	Yes	Mix
686	Van Hoecke, A.L.	2022	England	Europe	Cross sectional	538	No	Yes	Yes	Yes	No	Mix
687	Ourania Z. et al.,	2022	Greece	Europe	Cross sectional	9	No	Yes	Yes	Yes	Yes	Mix
688	Akman N & Yildiz A	2022	Turkey	Europe	Cross sectional	203	Yes	Yes	Yes	Yes	Yes	Mix
689	Caso, D	2022	Italy	Europe	Cross sectional	447	Yes	Yes	Yes	Yes	Yes	Mix
690	Fonseca, IC	2021	Portugal	Europe	Cross sectional	886	No	Yes	Yes	Yes	Yes	Mix
691	Stoeckel, F	2021	European Union	Europe	Cross sectional	18500	No	Yes	Yes	Yes	No	Mix
692	Whelan SO	2021	Ireland	Europe	Cross sectional	436	Yes	Yes	Yes	Yes	No	Mix
693	Ruggiero, KM	2021	France	Europe	Cross sectional	427	Yes	No	Yes	Yes	No	Mix
694	Byström E	2020	Sweden	Europe	Cross sectional	825	Yes	Yes	Yes	Yes	No	Mix
695	Charron J	2020	France	Europe	Cross sectional	3938	Yes	Yes	Yes	Yes	No	Mix
696	Lewandowska A	2020	Poland	Europe	Cohort study	1257	Yes	Yes	Yes	Yes	No	Mix
697	Facciolà A	2019	Italy	Europe	Cross sectional	1093	Yes	Yes	Yes	Yes	No	Mix
698	Miko D	2019	Romania	Europe	Cross sectional	452	Yes	Yes	Yes	Yes	No	Mix
699	Napolitano F	2018	Italy	Europe	Cross sectional	437	Yes	Yes	Yes	Yes	No	Mix
700	Betsch C	2018	German	Europe	Cohort study	943	Yes	Yes	Yes	Yes	No	Mix
701	Olszewska M	2018	Poland	Europe	Cross sectional	300	Yes	Yes	Yes	Yes	No	Mix
702	Tho,SL	2015	France	Europe	Cross sectional	1270	No	Yes	Yes	Yes	Yes	Mix
703	Dáňová J	2015	Czech Republic	Europe	Cross sectional	480	Yes	Yes	Yes	Yes	No	Mix
704	Harmsen IA	2013	Netherlands	Europe	Cross sectional	592	Yes	Yes	Yes	Yes	No	Mix
705	Harmsen IA	2013	Netherlands	Europe	Cross sectional	60	Yes	Yes	Yes	Yes	No	Mix
706	Harmsen IA	2012	Netherlands	Europe	Cross sectional	16	Yes	Yes	Yes	Yes	No	Mix
707	Borràs E	2009	Spain	Europe	Cross sectional	630	Yes	Yes	Yes	Yes	No	Mix
708	Stampi S	2005	Italy	Europe	Cross sectional	350256	Yes	Yes	Yes	Yes	No	Mix
709	Kirkedal, A-B.	2022	Denmark	Europe	Cross sectional	24	No	Yes	Yes	Yes	Yes	MMR
710	Jama A	2018	Sweden	Europe	Cross sectional	13	Yes	Yes	Yes	Yes	No	MMR
711	Anello P	2017	Italy	Europe	Cohort study	86255	Yes	Yes	Yes	Yes	No	MMR
712	Campbell	2017	England	Europe	Cross sectional	1792	Yes	Yes	Yes	Yes	No	MMR
713	Mchale P	2016	England	Europe	Cross sectional	142	Yes	Yes	Yes	Yes	No	MMR
714	Weiss C	2016	Switzerland	Europe	Cross sectional	101	Yes	Yes	Yes	Yes	No	MMR
715	Restivo V	2015	Italy	Europe	Cross sectional	443	Yes	Yes	Yes	Yes	No	MMR
716	Byström E	2014	Sweden	Europe	Cross sectional	20	Yes	Yes	Yes	Yes	No	MMR
717	Casiday R	2006	England	Europe	Cross sectional	996	Yes	Yes	Yes	Yes	No	MMR
718	Cassell JA	2006	England	Europe	Cross sectional	452	Yes	Yes	Yes	Yes	No	MMR
719	Hilton S	2006	England	Europe	Cross sectional	72	Yes	Yes	Yes	Yes	No	MMR
720	Dannetun E	2005	Sweden	Europe	Cross sectional	203	Yes	Yes	Yes	Yes	No	MMR
721	Alfredsson	2004	Sweden	Europe	Cross sectional	300	Yes	Yes	Yes	Yes	No	MMR
722	Evans M	2001	England	Europe	Cross sectional	48	Yes	Yes	Yes	Yes	No	MMR
723	Roberts RJ	1995	Wales	Europe	Cross sectional	307	Yes	Yes	Yes	Yes	No	MMR
724	Bag, O and Guney, SA	2023	Turkey	Europe	Cross sectional	110	No	No	Yes	Yes	Yes	Not stated

725	Grechukha, YO et al	2023	Ukraine	Europe	Cross sectional	797	Yes	Yes	Yes	No	Yes	Not stated
726	Miguel, I et al	2022	Portugal	Europe	Cross sectional	309	No	No	Yes	Yes	Yes	Not stated
727	Sythes L	2022	England	Europe	Cross sectional	10	No	Yes	Yes	Yes	Yes	Not stated
728	Brunelli L	2021	Italy	Europe	Cross sectional	2557	Yes	Yes	Yes	Yes	No	Not stated
729	Bertoncello C	2020	Italy	Europe	Cross sectional	3685	Yes	Yes	Yes	Yes	No	Not stated
730	Selleri P	2020	Italy	Europe	Cross sectional	972	Yes	Yes	Yes	Yes	No	Not stated
731	Seoane MD	2020	Spain	Europe	Case control	30	Yes	Yes	Yes	Yes	No	Not stated
732	Bianco A	2019	Italy	Europe	Cross sectional	575	Yes	Yes	Yes	Yes	No	Not stated
733	Cintulová LL	2019	Slovakia	Europe	Cross sectional	875	Yes	Yes	Yes	Yes	No	Not stated
734	Peretti-Watel. P	2019	France	Europe	Cross sectional	25	Yes	Yes	Yes	Yes	No	Not stated
735	Romijnders K.A,G,J.	2019	Netherlands	Europe	Cross sectional	55	Yes	Yes	Yes	Yes	No	Not stated
736	Wood L	2019	England	Europe	Cross sectional	85	Yes	Yes	Yes	Yes	No	Not stated
737	Bocquier A	2018	France	Europe	Cross sectional	15216	Yes	Yes	Yes	Yes	No	Not stated
738	Vrdelja M	2018	Slovenia	Europe	Cross sectional	750	Yes	Yes	Yes	Yes	No	Not stated
739	Braczkowska	2018	Poland	Europe	Cohort study	1239	No	No	No	Yes	Yes	Not stated
740	Giambi C	2017	Italy	Europe	Cross sectional	3130	Yes	Yes	Yes	Yes	No	Not stated
741	Martinez-Diz S.	2014	Spain	Europe	Cross sectional	16	Yes	Yes	Yes	Yes	No	Not stated
742	Avci, D	2023	Turkiye	Europe	Cross sectional	381	No	Yes	Yes	Yes	Yes	Others
743	Sherman, S.M.	2023	United Kingdom	Europe	Cross sectional	596	No	Yes	Yes	Yes	Yes	Others
744	Gundogdu, Z and Sezer, OY	2023	Turkey	Europe	Cross sectional	564	No	Yes	Yes	Yes	Yes	Others
745	Bankiewicz, P	2022	Poland	Europe	Cross sectional	53	No	Yes	Yes	Yes	Yes	Others
746	Gacs, Z	2022	Hungary	Europe	Cross sectional	430	No	No	No	No	No	Others
747	Huber A.	2020	Hungary	Europe	Cross sectional	1042	Yes	Yes	Yes	Yes	No	Others
748	Erb ML	2019	Switzerland	Europe	Cross sectional	199	Yes	Yes	Yes	Yes	No	Others
749	Mameli C	2014	Italy	Europe	Cross sectional	1842	Yes	Yes	Yes	Yes	No	Others
750	Allaert FA	2009	France and Germany	Europe	Cross sectional	2593	Yes	Yes	Yes	Yes	No	Others
751	Nehab, M. F	2023	Brazil	South America	Cross sectional	15297	No	Yes	Yes	Yes	Yes	Covid-19
752	Benites-Zapata, VA	2022	Colombia and Peru	South America	Cross sectional	68980	No	No	No	No	Yes	Covid-19
753	Martinez, E.Z.	2022	Brazil	South America	Cross sectional	1007	Yes	Yes	Yes	Yes	No	Covid-19
754	Bono, SA	2021	Brazil	South America	Cross sectional	6571	No	Yes	Yes	Yes	Yes	Covid-19
755	Bagateli, L.E.	2021	Brazil	South America	Cross sectional	501	No	Yes	Yes	Yes	No	Covid-19
756	Rodrigues E.S.	2023	Brazil	South America	Cross sectional	182	Yes	Yes	Yes	Yes	Yes	HPV
757	Olbrich Neto J	2023	Brazil	South America	Cross sectional	1261	No	Yes	Yes	No	No	Mix
758	Chung-Delgado, K	2021	Peru	South America	Cross sectional	552	No	Yes	Yes	Yes	No	Mix
759	Gentile	2021	Argentina	South America	Cross sectional	600	Yes	Yes	Yes	Yes	No	Mix
760	Burghouts J	2017	Venezuela	South America	Cross sectional	67	Yes	Yes	Yes	Yes	No	Mix
761	Logullo P	2008	Brazil	South America	Case control	122	Yes	Yes	Yes	Yes	No	Mix
762	González-Block MA	2021	Peru	South America	Cross sectional	160	Yes	Yes	Yes	Yes	No	Not stated
763	Brown AL	2018	Brazil	South America	Cross sectional	952	Yes	Yes	Yes	Yes	No	Not stated
764	Gonzales, A.	2022	Peru	South America	Cross sectional	133884	No	Yes	Yes	Yes	No	Others
765	Kuan-Mahecha M.A. et al.,	2023	Guatemala	South America	Cross sectional	503	Yes	No	Yes	No	Yes	Mix
