# Supplementary material for: Prevalence of caregiver hesitancy for vaccinations in children and its associated factors: A systematic review and meta-analysis
Source: PLoS One. 2024 Oct 24;19(10):e0302379. doi: 10.1371/journal.pone.0302379 (PMC11500859; doi:10.1371/journal.pone.0302379)
Supplement: S4 Table — (PDF) [file pone.0302379.s008.pdf]

S4 Table: **Quality Assessment for Included Study**

Newcastle-Ottawa Quality Assessment Form for Case Control

No	Author	Year	Selection				Comparability	Outcome			Quality score
			S1	S2	S3	S4		O1	O2	O3	
1	Hailu, C.	2022	*	*	*	*	**	*	*		Good
2	Machekanyanga Z	2017		*			**	*			Fair
3	M Muhsen K	2012						*			Poor
4	Seoane MD	2020			*		*	*			Poor
5	Logullo P	2008			*		*	*	*		Poor
6	Bardenheier B	2004			*	*		*	*		Fair
7	Salmon DA	2005		*	*	*	*				Poor
8	Abubakar A	2019	*	*	*		*	*			Fair
9	Mohammed A	2014	*	*	*	*	*	*			Fair

Newcastle-Ottawa Quality Assessment Scale Case Control Studies

Note: A study can be given a maximum of one star for each numbered item within the Selection and Outcome categories. A maximum of two stars can be given for Comparability.

Selection

1. Is the case definition adequate?

. Yes, with independent validation *
a. Yes, eg record linkage or based on self reports
b. No description

2. Representativeness of the cases

a. Consecutive or obviously representative series of cases*

b. Potential for selection biases or not stated

1. Selection of Controls

a. Community controls*

b. Hospital controls

c. No description

2. Definition of Controls

a. No history of disease (endpoint)*
b. No description of source

Comparability

1. Comparability of cases and controls on the basis of the design or analysis

a. Study controls for ________________ (Select the most important factor.)*
b. Study controls for any additional factor***** (This criteria could be modified to indicate specific control for a second important factor.)

Outcome

1. Ascertainment of exposure

a. Secure record (eg surgical records)*

b. Structured interview where blind to case/control status*

c. Interview not blinded to case/control status

d.Written self report or medical record only

e. No description

1. Same method of ascertainment for cases and controls

a. Yes*

b. No

0. Non-Response rate

a. Same rate for both groups*

b. Non respondents described

c. Rate different and no designation

Thresholds for converting the Newcastle-Ottawa scales to AHRQ standards (good, fair, and poor):

Good quality: 3 or 4 stars in selection domain AND 1 or 2 stars in comparability domain AND 2 or 3 stars in outcome/exposure domain

Fair quality: 2 stars in selection domain AND 1 or 2 stars in comparability domain AND 2 or 3 stars in outcome/exposure domain

Poor quality: 0 or 1 star in selection domain OR 0 stars in comparability domain OR 0 or 1 stars in outcome/exposure domain

Modified for Newcastle-Ottawa Quality Assessment Form for Cross sectional

No	Author	Year	Selection			Comparability	Outcome		Quality score
			S1	S2	S3		E1	E2	
1	Zewdie, A	2016	*	*		*			Poor
2	Babirye, J.N	2011	*						Poor
3	Schwarz, NG	2009	*						Poor
4	Tadesse, H.	2009	*						Poor
5	Leach, MA	2008	*						Poor
6	Cassell, JA	2006	*	*		*		*	Fair
7	Helman, CG	2004	*			*			Poor
8	Eng, E.	1991	*			*			Poor
9	Cutts, FT	1990	*			*			Poor
10	Carcelen, AC	2022	*	*		*		*	Good
11	Muluye, M	2022	*	*		**		*	Good
12	Lubeya M.K. et al.,	2023	*			**		*	Fair
13	Balogun, FM	2022	*			**		*	Fair
14	Kolek, C. O.	2022	*			**		*	Fair
15	Mihretie, G. N.	2022	*	*		**		*	Good
16	Milondzo T. et al.,	2022	*	*		*		*	Good
17	Popelsky, BK	2021	*		*	**		*	Good
18	Kamya, C	2022	*			**		*	Fair
19	Darebo, TD	2022	*	*		**		*	Good

20	Abor J	2022	*		*	**		*	Good
21	Killion JP	2022	*			*		*	Fair
22	Tayu B	2022	*			*		*	Fair
23	Jalloh, MF	2022	*			*			Poor
24	Powelson, J	2022	*			*			Poor
25	Olaniyan, A. et al.,	2022	*			*			Poor
26	Ames, H.	2021	*			*			Poor
27	Tabana, H	2016	*			**		*	Fair
28	Sabahelzain, M.M.	2022			*	*	*	*	Fair
29	Gil Cuesta, JG	2021	*			*			Poor
30	Cockcroft, A	2014	*			*	*		Fair
31	Mebrate, M.	2022	*	*		**		*	Fair
32	Sulaiman S.K. et al.,	2023	*			**		*	Fair
33	Yeboah, D.	2022	*		*	**		*	Good
34	Immurana, M	2021	*			**		*	Fair
35	Wigle J.	2023	*			**	*		Fair
36	Shen A.K.	2022	*			**			Poor
37	Batra, K.	2023	*		*	**		*	Good
38	Davidson, CA et al	2023	*		*	**			Poor
39	Durkin, LK	2023	*		*	**			Poor
40	Gooding, GD et al	2023	*			*		*	Fair
41	Mondal P & Sinharoy A	2023	*	*		**		*	Good

42	Kohler, RE et al	2023	*		*	*			Poor
43	Phan TL	2022	*		*	*	*	*	Good
44	Baumer-Mouradian, S.H.	2022	*		*	*		*	Good
45	Cioffredi, LA	2022	*		*	*	*	*	Good
46	Guerin, RJ	2022	*			*	*	*	Good
47	Hopfer, S	2022	*		*	*		*	Good
48	Humble, RM	2022	*	*	*	*	*	*	Good
49	Kreuter, MW	2022	*			*		*	Fair
50	Lam, C.N	2022	*			*	*	*	Good
51	Limbers, CA	2022	*		*	*		*	Good
52	Mangat, C	2022	*	*		*		*	Good
53	McElfish, P.A.	2022	*			*		*	Fair
54	Nguyen, KH	2022	*		*	*	*	*	Good
55	Nguyen, KH	2022	*		*	*	*	*	Good
56	Panchalingam, T.	2022	*	*	*	*		*	Good
57	Reindl, D.	2022	*		*	*		*	Good
58	Salazar, TL	2022	*	*	*	*		*	Good
59	Scherer, AM	2022	*			*		*	Fair
60	Schiff, J.	2022	*		*	*			Poor
61	Schilling, S	2022	*		*	*		*	Fair
62	Skeens, M.	2022	*	*	*	*		*	Good

63	Skeens, M. A.	2022	*		*	*		*	Good
64	Suvada, KA	2022	*	*	*	*		*	Good
65	Willis, DE	2022	*			*		*	Fair
66	Ruiz JB	2022	*	*	*	*		*	Good
67	Lacy, R.	2022	*		*	*		*	Good
68	Lessard, L	2022	*			**		*	Fair
69	Bonuck, K	2022	*			**		*	Fair
70	Byrne, A	2022	*			*		*	Fair
71	Ellithorpe, ME	2022	*		*	*	*	*	Good
72	Fisher, C.B.	2022	*	*	*	*			Poor
73	O’Dor SL	2022	*		*	*		*	Good
74	Baumann, BM	2022	*		*	*	*	*	Good
75	Olagoke, A.	2022	*			*		*	Fair
76	Lachance-Grzela, M. et al.,	2022	*		*	*		*	Good
77	Kheil, M.H. et al.,	2022	*		*	*		*	Good
78	Choi, K. et al.,	2022	*			*		*	Fair
79	Zhu, Y	2022	*						Poor
80	Waring, M. E.	2022	*			*		*	Fair
81	Delgado-Gallegos, J.L.	2021	*	*	*	*		*	Good
82	Lackner, CL	2021	*			**		*	Fair
83	Griffith, BC	2022	*					*	Poor

84	Sokol, RL and Grummom, AH	2020	*			**		*	Fair
85	Davis, MM et al	2020	*			**		*	Fair
86	Shin, M.B.	2023	*			**			Poor
87	Jin, SW et al	2023	*			**		*	Fair
88	Xiong, S	2022	*			*			Poor
89	Margolis M. A. et al.,	2022	*			**		*	Fair
90	Newcomer SR	2020	*				*	*	Poor
91	Pomares TD	2020	*		*	**		*	Fair
92	Yankey D	2020	*			**		*	Fair
93	Duchsherer A. et al.,	2020	*			**			Poor
94	Beavis A	2018	*			**		*	Fair
95	Krok-Schoen JL	2018	*			**		*	Fair
96	Lee Y.M	2018	*			**		*	Fair
97	Brown B	2017	*	*		**		*	Good
98	Gilbert NL	2016	*			**		*	Fair
99	Krawczyk A	2015	*			**			Poor
100	Perez S	2015	*			*			Poor
101	Pitts MJ	2013	*			*			Poor
102	Ogilvie G	2010	*			**		*	Fair
103	Nguyen, AT	2022			*	*	*	*	Good
104	Beatty and Villwock	2021	*		*	**		*	Good

105	Kempe A	2020	*		*	**		*	Good
106	Nekrasov E	2020	*	*	*	**		*	Good
107	Goss MD	2020	*		*	**		*	Good
108	Baumgaertner, B et al	2018	*			**		*	Fair
109	Cameron MA	2016	*			**	*	*	Good
110	Santibanez TA	2016	*			**		*	Fair
111	Schellenberg, N.	2023	*			**	*	*	Good
112	Baumer-Mouradian, S.H.	2022	*	*	*	*		*	Good
113	Ellithorpe, M	2022	*			*		*	Fair
114	Howell, JL	2022	*		*	**		*	Good
115	Hsu, C	2022	*		*	**			Poor
116	Kaufmann J	2022	*			**		*	Fair
117	Letterie MC	2022	*			**		*	Fair
118	Nguyen, K.H	2022	*	*		*	*	*	Good
119	Opel, D. J.	2022	*		*	*		*	Good
120	Shah, MD	2022	*			*		*	Fair
121	Wang, CS	2022	*		*	*	*	*	Good
122	Anderson-Chavarria, M	2022	*			*			Poor
123	Boyce, TG	2022	*		*	*	*	*	Good
124	Footman, A	2022	*			*		*	Fair
125	Shen, AK	2023	*			*			Poor

126	Gennaro, E et al	2021	*			*	*	*	Good
127	Langkamp, DL et al	2020	*		*	*		*	Good
128	Navin MC	2019	*			*	*	*	Good
129	Carpiano RM	2019	*		*	*	*	*	Good
130	Gilkey MB	2016	*		*		*	*	Fair
131	William S.E. et al.,	2016	*		*	*	*	*	Good
132	Frew, PM et al	2016	*		*	*		*	Good
133	Greenfield LS	2015	*		*	*		*	Good
134	Roberts JR	2015	*		*	*	*	*	Good
135	Wolff ER	2014	*		*	*		*	Good
136	Opel D.J. et al.,	2013	*		*	*	*	*	Good
137	Bazzano A	2012	*		*	*		*	Good
138	Smith P.J. et al.,	2010	*			*		*	Fair
139	Salmon DA	2009	*			*		*	Fair
140	Wu AC	2008	*			*		*	Fair
141	Bonsu, NE et al	2021	*		*	*	*	*	Good
142	Holroyd T.A. et al.,	2021	*			*		*	Fair
143	Wharton-Michael P & Wharton-Clark A	2020	*			*			Poor
144	Rodriguez-Nava G. et al.,	2020					*	*	Poor
145	Qian M. et al.,	2020	*			*	*	*	Good
146	Mills, K et al	2020	*			*			Poor

147	Christianson, B et al	2020	*			*	*	*	Good
148	Estep, K and Greenberg, P.	2020	*			*	*	*	Good
149	Nyathi S. et al.,	2019				*	*	*	Poor
150	Krishna A	2017			*	*		*	Fair
151	Kang G.J. et al.,	2017				*			Poor
152	Cacciatore MA	2016	*			*		*	Fair
153	Cataldi, JR et al	2016	*		*	*	*	*	Good
154	McNutt, LA	2016	*			*	*	*	Good
155	Lieu, TA et al	2015			*	*	*	*	Good
156	Nyhan B, et al	2014	*			*		*	Fair
157	Kennedy A.M & Gust D.A	2008	*			*			Poor
158	Boyle J	2020	*			*		*	Fair
159	Buttenheim AM	2020	*	*	*	**		*	Good
160	Mohanty S	2020	*		*	*		*	Good
161	Buckman, C. et al	2020	*			*	*	*	Good
162	Cataldi JR	2019	*		*	*		*	Good
163	Cheng ER	2019	*		*	*		*	Good
164	McCoy JD	2019	*			*		*	Fair
165	McDonald P	2019	*			*		*	Fair
166	Dubé	2019	*	*	*	*		*	Good
167	Guay M	2019				*		*	Poor

168	Carrion ML	2018	*			*			Poor
169	Carrion ML	2018	*			*			Poor
170	Chung Y	2017	*			*		*	Fair
171	Kettunen C.	2017	*			*			Poor
172	Dubé E	2018	*		*	*		*	Good
173	Blaisdell LL	2016	*			*			Poor
174	Lee C	2016	*			**		*	Fair
175	Dubé E	2016	*		*	*			Poor
176	MacDonald SE	2014	*		*	*	*	*	Good
177	Mergler M.J	2013	*			*		*	Fair
178	Luthy KE	2012	*			*			Poor
179	Luthy KE	2010	*			*			Poor
180	Kulig JC	2002	*			*			Poor
181	Sahni LC	2020	*		*	*		*	Good
182	Gromis A & Liu K.Y	2020	*				*	*	Poor
183	Zhou T	2023	*		*	*	*	*	Fair
184	Zhang H.	2023	*	*	*	*	*	*	Good
185	Zhang K	2023	*		*	*	*	*	Good
186	Bourguiba, A	2023	*			*	*	*	Good
187	Alhuzaimi, AN	2023	*	*	*	*	*	*	Good
188	Almuqbil, M.	2023	*	*	*	*	*	*	Good
189	Ashour, HA	2023	*	*	*	*	*	*	Good

190	Chawanpaiboon S.	2023	*	*	*	*	*	*	Good
191	Dao, T.L. et al.,	2023	*			*		*	Fair
192	Deng, JS et al	2023	*	*		*	*	*	Good
193	Ghazy, RM et al	2023	*	*	*	*	*	*	Good
194	Maneesriwongul W.	2023	*		*	*	*	*	Good
195	Khatrawi, EM and Sayed, AA	2023	*	*		*	*	*	Good
196	Khoodoruth, MAS et al	2023	*		*	*	*	*	Good
197	Zhou Y	2021	*		*	*	*	*	Good
198	Zhou, M	2022	*	*	*	*	*	*	Good
199	Abdalla, SM	2022	*			*		*	Fair
200	Alhazza, SF	2022	*	*	*	*	*	*	Good
201	Ali M.	2022	*	*		*	*	*	Good
202	Ali, M	2022	*			*	*	*	Good
203	Ali-Saleh, O	2022	*	*	*	*	*	*	Good
204	Almalki OS	2022	*		*	*	*	*	Good
205	Chia MY	2022	*	*	*	*		*	Good
206	Choi, U. I.	2022	*	*	*	*	*	*	Good
207	Duong, A.H	2022	*	*		*		*	Good
208	Gunes, O	2022	*			*	*	*	Fair
209	Hou, Z.	2022	*		*	*	*	*	Good
210	Huang, L.L	2022	*	*	*	*	*	*	Good

211	Khan, YH	2022	*		*	*	*	*	Good
212	Kitro, A	2022	*		*	*	*	*	Good
213	Lee, M.	2022	*		*	*	*	*	Good
214	Li, JB	2022	*		*	*	*	*	Good
215	Low, JM	2022	*			*	*	*	Good
216	Lu, L	2022	*			*		*	Good
217	Ng, DL	2022	*	*	*	*	*	*	Good
218	Parinyarux, P.	2022	*	*	*	*	*	*	Good
219	Qin C	2022	*	*	*	*	*	*	Good
220	Rehman, T	2022	*	*	*	*	*	*	Good
221	Samudyatha, UC	2022	*			*	*	*	Good
222	Shahani, R	2022	*		*	*	*	*	Good
223	Shwethashree, M	2022	*	*		*	*	*	Good
224	Tang, S.	2023	*		*	*	*	*	Good
225	Tung, T. H.	2022	*	*		*		*	Good
226	Wang, K	2022	*		*	*	*	*	Good
227	Wang, L	2022	*			*		*	Fair
228	Zhou, X	2022	*		*	*	*	*	Good
229	Khatatbeh, M	2022	*		*	*	*	*	Good
230	Huang, L.L	2022	*		*	*	*	*	Good
231	Abuhammad, S	2022	*		*	*	*	*	Good
232	Aedh, AI	2022	*	*	*	*	*	*	Good

233	AlKetbi, LMB	2022	*		*	*	*	*	Good
234	Al-khlaiwi, T	2022	*	*	*	*	*	*	Good
235	Almansour, A	2022	*	*	*	*	*	*	Good
236	Al-Qahtani, AM	2020	*	*	*	*	*	*	Good
237	Al-Qerem, W	2022	*	*	*	*	*	*	Good
238	Alsulaiman, JW	2022	*		*	*	*	*	Good
239	Bord, S	2022	*		*	*	*	*	Good
240	Elkhadry, S.W.	2022	*	*	*	*	*	*	Good
241	ElSayed, D.A	2022	*	*	*	*	*	*	Good
242	Ennaceur, S.	2022	*		*	*	*	*	Good
243	Kharaba, Z	2022	*	*	*	*	*	*	Good
244	Mohammed, AH	2022	*			*		*	Fair
245	Morozov, N. G.	2022	*			*	*	*	Good
246	Reagu, S.	2022	*		*	*	*	*	Good
247	Savitsky, B.	2022	*	*		*		*	Good
248	Swed, S	2022	*	*	*	*		*	Good
249	Akgün O	2022	*			*	*	*	Good
250	AL-Iede M	2022	*			**		*	Fair
251	Al-Rasheedi AT	2022	*	*	*	*	*	*	Good
252	Bas K	2022	*		*	*	*	*	Good
253	Çağ Y	2022	*			*	*	*	Good
254	Lau, EY	2022	*			*	*	*	Good

255	Li, T	2022	*			*	*	*	Good
256	Al-Qerem, W	2022	*	*		*	*	*	Good
257	Kocamaz, EB	2022	*	*	*	*	*	*	Good
258	Tsai, C-S. et al	2022	*		*	*		*	Good
259	Cho, HK. et at	2022	*	*		*	*	*	Good
260	Wong L.P. et al.,	2022	*	*	*	*		*	Good
261	Li K. & Zhou F.	2022	*	*	*	*	*	*	Good
262	Aljamaan, F. et al.,	2022	*	*	*	*	*	*	Good
263	Çelik, M.Y.	2021	*	*	*	*	*	*	Good
264	Almusbah, Z	2021	*			*	*	*	Good
265	Altulaihi, BA	2021	*	*	*	*	*	*	Good
266	Atad, E	2021	*			*	*	*	Good
267	Padhi, BK	2021	*	*		*		*	Good
268	Choi SH	2021	*	*		*	*	*	Good
269	Wan, X.	2021	*		*	*	*	*	Good
270	Yilmaz, M.	2021	*	*		*	*	*	Good
271	Zhou, Y	2021	*			*	*	*	Good
272	Musa, S.	2021	*	*		*	*	*	Good
273	Aldakhil, H.	2021	*	*	*	*	*	*	Good
274	Samannodi, M.	2021	*	*	*	*	*	*	Good
275	Shmueli, L.	2023	*		*	*	*	*	Good
276	Gendler Y	2021	*	*	*	*	*	*	Good

277	Wang, Z.	2021	*	*	*	*	*	*	Good
278	Altulahi, N.	2021	*	*	*	*	*	*	Good
279	Feng, H	2021	*		*	*	*	*	Good
280	Zhou , Y	2021	*			*		*	Fair
281	Wang, X.	2021	*			*		*	Fair
282	Yoda, T. et al.,	2021	*			*	*	*	Good
283	Horiuchi, S. et al.,	2021	*			*		*	Fair
284	Lin Y et al	2021	*			**		*	Fair
285	Lu X	2021	*		*	*	*	*	Good
286	Zhang, KC	2020	*		*	*	*	*	Good
287	Xie, H.	2023	*	*	*	*		*	Good
288	Babi A.	2023	*		*	*		*	Good
289	Choi, J.	2023	*			*			Poor
290	Choi. J	2021	*		*	*		*	Good
291	Frianto, D.	2022	*		*	*	*	*	Good
292	Pearl, CA	2022	*		*	*	*	*	Good
293	Tubas F	2022	*			*		*	Fair
294	Zhang, Z	2023	*			*	*	*	Good
295	Akca, G	2022	*		*	*	*	*	Good
296	Huang, Z	2022	*		*	*	*	*	Good
297	Zach, R	2022	*			*			Poor
298	Imanishi Y	2022	*			*	*	*	Good

299	Yagi, A	2022	*		*	*	*	*	Good
300	Alkalash, SH	2022	*	*	*	*	*	*	Good
301	Shuto	2021	*		*	*	*	*	Good
302	Ugumori	2021	*			*	*	*	Good
303	Huang Y	2021	*	*	*	*	*	*	Good
304	Kobayashi	2021	*		*	*	*	*	Good
305	Miyoshi, A	2020	*			*	*	*	Good
306	Yagi A	2018	*			*		*	Fair
307	Shida, J	2015	*			*		*	Fair
308	Egawa-Takata	2015	*			*	*	*	Good
309	Hanley	2014	*		*	*		*	Good
310	Hanley S	2012	*			*	*	*	Good
311	Al-Qerem, W	2023	*	*	*	*	*	*	Good
312	Alharbi, I.	2023	*	*		*	*	*	Good
313	Fan, J.	2022	*	*	*	*		*	Good
314	Lai, X	2022	*	*	*	*		*	Good
315	Abed Elhadi Shahbari N	2022	*	*	*	*		*	Good
316	Alenazi, K. A	2022	*		*	*	*	*	Good
317	AlOmran, HA	2022	*	*	*	*	*	*	Good
318	Hussein, YH	2022	*		*	*	*	*	Good
319	Jiang, M	2022	*	*	*	*		*	Good
320	Zakhour R	2021	*		*	*	*	*	Good

321	Salawati, E.	2021	*	*	*	*	*	*	Good
322	Alolayan A	2019	*	*	*	*	*	*	Good
323	Alabbad AA	2018	*	*	*	*	*	*	Good
324	Buyuktiryaki B	2014	*			*	*	*	Good
325	Akis S	2011	*			*	*	*	Good
326	Ahmed, N	2023	*		*	*	*	*	Good
327	Sahoo S. S.	2023	*		*	*		*	Good
328	Du, M.	2022	*		*	*		*	Good
329	Han, K.	2022	*			*		*	Fair
330	Khaliq, A	2022	*		*	*		*	Good
331	Shaipuzaman, N. A	2022	*			*		*	Fair
332	Sinuraya, R. K.	2022	*			*		*	Fair
333	Summan, A.	2022	*			*	*	*	Good
334	Wachinger, J	2022	*			*			Poor
335	Wang, Q(a)	2022	*		*	*		*	Good
336	Akbulut, S	2022	*		*	*		*	Good
337	Alghamdi, S	2022	*		*	*		*	Good
338	Al-Regaiey, KA	2022	*		*	*		*	Good
339	Hijazi, R	2022	*			*			Poor
340	Abu-rish EY	2016	*			*		*	Fair
341	Ozer, M	2022	*			*		*	Fair
342	Topaktas B.	2022	*			*		*	Fair

343	Al Yamani, Z.J. et al.,	2022	*		*	*		*	Good
344	Zin ZM. et al	2022	*			*			Poor
345	Temsah, MH	2021	*		*	*		*	Good
346	Wang, Q	2021	*		*	*		*	Good
347	Yilmazbaz, P	2021	*		*	*		*	Good
348	Tal, O	2021	*			*		*	Fair
349	Al-Nafeesah AS	2021	*					*	Poor
350	Baghdadi, LR	2021	*	*	*	*		*	Good
351	Hou, Z.	2021	*			**		*	Fair
352	Alsubaie SS	2019	*		*	*		*	Good
353	Chang K	2019	*		*	*		*	Good
354	Jalloh M.F.	2019	*			*			Poor
355	Amit Aharon A	2018	*			*		*	Fair
356	Chan HK	2018	*			*	*	*	Good
357	Dasgupta P	2018	*		*	*		*	Good
358	Khaliq A.	2017	*			*			Poor
359	Quaiyum MA	2011	*			*			Poor
360	Hussein SZ	2022	*			*		*	Fair
361	Ashkenazi S	2020	*			*		*	Fair
362	Abdullah AC	2018	*		*	*		*	Good
363	Fakhruddin TM et al	2023	*	*	*	*		*	Good
364	Han Y	2022	*		*	*		*	Good

365	Kuan, CI	2022	*			*			Poor
366	Shen, X	2022	*	*	*	*		*	Good
367	Alaamri, O.	2022	*		*	*		*	Good
368	Yörük S	2021	*	*	*	*		*	Good
369	Akhmetzhanova Z	2020	*		*	*		*	Good
370	AlGoraini YM	2020	*	*	*	*			Good
371	Alsuwaidi AR	2020	*		*	**		*	Good
372	Çağ Y	2020	*			*		*	Fair
373	Gunes NA	2020	*		*	*		*	Good
374	Kalok A.	2020	*	*	*	*		*	Good
375	Noyman-Veksler G	2020	*		*	*		*	Good
376	Hu Y	2019	*		*	*		*	Good
377	Ren J	2018	*	*	*	*		*	Good
378	Sun X	2018	*		*	*		*	Good
379	Noor T	2018	*			*		*	Fair
380	Aharony N	2017	*		*				Poor
381	Azizi FS	2017	*	*	*	*		*	Good
382	Ali HYM	2004	*			*	*	*	Good
383	Gesser-Edelsburga A	2016	*			*			Poor
384	Khowaja AR	2012	*			*		*	Fair
385	Qi L	2019	*		*	*		*	Good
386	Yang, Y	2022	*		*	*		*	Good

387	Al-Iede, M	2022	*		*	*		*	Good
388	Ni, Y-H.	2023	*			*	*	*	Good
389	Du, Y et al	2022	*		*	*		*	Good
390	Khan TM	2016	*			*			Poor
391	Wang, M	2022	*		*	*		*	Good
392	Sharif Nia et al.,	2023	*		*	*		*	Good
393	Goldman, RD	2022	*		*			*	Fair
394	Goldman, RD	2022	*	*		*		*	Good
395	Skjefte, M.	2021	*			**		*	Fair
396	Goldman, RD	2021	*					*	Poor
397	Goldman RD	2020	*	*		*		*	Good
398	Yilmaz M	2023	*		*	*		*	Good
399	Tekin C.	2023	*		*	*		*	Good
400	Borras-Bermejo B	2022	*		*	*	*	*	Good
401	Durmaz, N.	2022	*		*	*		*	Good
402	Tan L.	2023	*	*	*	*		*	Good
403	Reuben R	2020	*		*	*		*	Good
404	Bolsewicz, KT et al	2023	*			*			Poor
405	Wen, LM	2022	*	*	*	*		*	Good
406	Jeffs, E	2021	*		*	*		*	Good
407	Evans, S	2021	*	*	*	*		*	Good
408	Biezen R	2018	*		*	*		*	Good

409	Jones K	1992	*			*	*	*	Good
410	Wright, D	2022	*		*	*		*	Good
411	Debela, M. S.	2022	*	*	*	*		*	Good
412	Enkel SL	2018	*			*			Poor
413	Forbes TA	2015	*		*	*	*	*	Good
414	Tuckerman J	2020	*			*			Poor
415	Bryden GM	2019	*			*	*	*	Good
416	Helps C	2019	*			*			Poor
417	Rossen I	2019	*		*	*			Poor
418	Rozbroj T	2019	*		*	*			Poor
419	Attwell K	2018	*			*			Poor
420	Attwell K	2018	*			*			Poor
421	Corben P	2018	*		*	*	*	*	Good
422	Frawley J.E	2018	*		*	*		*	Good
423	Attwell K	2017	*			*			Poor
424	Attwell K	2016	*					*	Poor
425	Gilmartin CE	2020	*			*	*	*	Good
426	Ates, BO	2023			*	*		*	Good
427	Bektas, I and Bektas, M	2023	*		*			*	Poor
428	Celik, T and Dogan, D	2023	*			*		*	Fair
429	Sasic M. et al.,	2023	*			*		*	Fair
430	Sahin, A. et al.,	2023	*			*		*	Fair

431	Esposito, S. et al	2023	*		*	*		*	Good
432	Ganem, F et al	2023	*			*		*	Fair
433	Kozlarek, M.	2022	*			*		*	Fair
434	Bianco, A.	2022	*			*		*	Fair
435	Di Giuseppe, G.	2022	*			*		*	Fair
436	Iannello, P.	2022	*		*	*		*	Good
437	Krakowczyk, J.B.	2022	*		*	*			Poor
438	Lecce, M	2022	*	*		*		*	Good
439	Manolescu, L.S.C.	2022	*	*		*		*	Good
440	Marcau, F.C.	2022	*			*		*	Fair
441	Miliordos, K	2022	*			*		*	Fair
442	Miraglia del Giudice, G	2022	*	*	*	*		*	Good
443	Napoli, A.	2022	*	*		*		*	Good
444	Rees, F	2022	*		*	*		*	Good
445	Steletou, E	2022	*			*		*	Fair
446	Schmidtke, K. A	2022			*	*		*	Good
447	Skirrow, H.	2022	*	*		*		*	Good
448	Wagner, A	2022	*			*		*	Fair
449	Savarese G.	2022	*		*	*		*	Good
450	Babicki, M	2021	*			**		*	Fair
451	Brandstetter, S	2021	*			*		*	Fair
452	Fedele	2021	*			*	*	*	Good

453	Galanis, P	2022	*			*		*	Fair
454	Galanis, P	2021	*			*		*	Fair
455	Seiler, M.	2021	*			*		*	Fair
456	Zona, S. et al.,	2021	*			*		*	Fair
457	Russo, L. et al.,	2021	*	*		*		*	Good
458	Bell S	2020	*			*		*	Fair
459	Sobierajski, T.	2023	*			*		*	Fair
460	Sˇtrbac M. et al.,	2023	*			*		*	Fair
461	Abuduxike G	2022	*			*		*	Fair
462	Calagna G	2022	*			*		*	Fair
463	Lopez, N.	2022	*		*	*		*	Good
464	Karafillakis, E	2022	*			*			Poor
465	Smolarczyk, K.	2022	*			*		*	Fair
466	Taylor, J	2022	*			*		*	Fair
467	Venderbos, J. R.	2022	*			*			Poor
468	Naoum, P	2022	*			*		*	Fair
469	Lopez, N.	2022	*		*	*		*	Good
470	Della Polla G	2020	*		*	*		*	Good
471	Waller J	2020	*			*		*	Fair
472	Teasdale, CA	2021	*					*	Poor
473	Grandahl M	2017	*		*	*		*	Good
474	Navarro-Illana P	2015	*			*		*	Fair

475	Grandahl M	2014	*			*			Poor
476	Kornfeld J	2013	*		*	*		*	Good
477	Craciun C	2012	*			*			Poor
478	Hontelez J.A.C.	2010	*		*	*		*	Good
479	Kiroplis et al.,	2023	*			*		*	Fair
480	Di Giuseppe, G.	2022	*			*			Poor
481	Bielecki K	2020	*			**		*	Fair
482	Gorman D.R	2020	*		*	*		*	Good
483	Prospero E	2019	*			*		*	Fair
484	Rodríguez-Blanco N	2019	*			*		*	Fair
485	P. Paterson	2018	*			*			Poor
486	Bults M	2011	*			*		*	Fair
487	Brown K.F	2010	*			*		*	Fair
488	Gjini, E.	2023	*		*	*		*	Good
489	Duran, S et al	2023	*		*	*	*	*	Good
490	Marron, L. et al	2023	*		*	*	*	*	Good
491	Buonsenso, D	2022	*			*	*	*	Good
492	Deml, MJ	2022	*			*			Poor
493	Derdemezis, C.	2022	*		*	**		*	Good
494	Ebi, SJ	2022	*		*	**		*	Good
495	Fakonti, G	2022	*			*		*	Fair
496	Jafflin, K	2022	*		*	*		*	Good

497	Miron, V. D.	2022	*			*		*	Fair
498	Napolitano, F	2022	*		*	**		*	Good
499	Nurmi, J	2022	*			**			Poor
500	Skitareli´c, N.	2022	*			*			Poor
501	Van Hoecke, A.L.	2022	*	*	*	*		*	Good
502	Ourania Z. et al.,	2022	*			*			Poor
503	Akman N & Yildiz A	2022	*		*	*		*	Good
504	Caso, D	2022	*	*		*		*	Good
505	Fonseca, IC	2021	*		*	*		*	Good
506	Stoeckel, F	2021					*	*	Poor
507	Whelan SO	2021	*		*	*		*	Good
508	Ruggiero, KM	2021	*		*	*		*	Good
509	Byström E	2020	*			*		*	Fair
510	Charron J	2020	*			*		*	Fair
511	Facciolà A	2019	*			*		*	Fair
512	Miko D	2019	*		*	*			Poor
513	Napolitano F	2018	*		*	*		*	Good
514	Olszewska M	2018	*			*	*	*	Good
515	Tho,SL	2015	*			*		*	Fair
516	Dáňová J	2015	*			*	*		Fair
517	Harmsen IA (a)	2013	*			*		*	Fair
518	Harmsen IA (b)	2013	*			*			Poor

519	Harmsen IA	2012	*			*			Poor
520	Borràs E	2009	*			*	*	*	Good
521	Stampi S	2005				*	*		Poor
522	Kirkedal, A-B.	2022	*			*			Poor
523	Jama A	2018	*			*			Poor
524	Campbell	2017	*			*	*		Fair
525	Mchale P	2016	*			*	*		Fair
526	Weiss C	2016	*			*		*	Fair
527	Restivo V	2015	*			*	*	*	Fair
528	Byström E	2014	*			*			Poor
529	Casiday R	2006	*			*	*	*	Good
530	Cassell JA	2006	*	*		*	*	*	Good
531	Hilton S	2006	*			*			Poor
532	Dannetun E	2005	*			*	*	*	Good
533	Alfredsson	2004	*			*	*	*	Good
534	Evans M	2001	*			*			Poor
535	Roberts RJ	1995	*			*	*	*	Good
536	Bag, O and Guney, SA	2023	*			*		*	Fair
537	Grechukha, YO et al	2023	*			*		*	Fair
538	Miguel, I et al	2022	*		*	*		*	Good
539	Sythes L	2022	*			*			Poor
540	Bertoncello C	2020	*			*		*	Fair

541	Selleri P	2020	*			*		*	Fair
542	Bianco A	2019	*		*	*		*	Good
543	Cintulová LL	2019	*			*		*	Fair
544	Peretti-Watel. P	2019	*			*			Poor
545	Romijnders K.A,G,J.	2019	*			*			Poor
546	Wood L	2019	*		*	*		*	Good
547	Bocquier A	2018	*			*		*	Fair
548	Vrdelja M	2018	*			*			Poor
549	Giambi C	2017	*			*		*	Fair
550	Martinez-Diz S.	2014	*			*			Poor
551	Avci, D	2023		*				*	Poor
552	Sherman, S.M.	2023	*	*		*		*	Good
553	Gundogdu, Z and Sezer, OY	2023	*			**		*	Fair
554	Bankiewicz, P	2022	*			*			Poor
555	Gacs, Z	2022	*			*		*	Fair
556	Huber A.	2020	*			*		*	Fair
557	Erb ML	2019	*	*		*	*	*	Good
558	Mameli C	2014	*			*		*	Fair
559	Allaert FA	2009	*			*		*	Fair
560	Benites-Zapata, VA	2022	*	*	*	*		*	Good
561	Martinez, E.Z.	2022		*	*	*		*	Good

562	Nehab, M. F	2023	*			*		*	Fair
563	Bono, SA	2021	*			*		*	Fair
564	Rodrigues E.S.	2023	*			*	*	*	Good
565	Olbrich Neto J	2023	*			*		*	Fair
566	Chung-Delgado, K	2021	*		*	*			Poor
567	Kyei-Arthur, F	2022	*			*		*	Fair
568	Al-Wutayd, O	2022	*			*		*	Fair
569	Elit L	2022	*			*			Poor
570	Horn, S	2022	*			**		*	Fair
571	Kemeugni Ngandjon, J.	2022	*			*		*	Fair
572	Idris, I. O.	2022	*			**		*	Fair
573	Vasudevan L	2020	*			*		*	Fair
574	Kagone, M	2018	*			*			Poor
575	Abakar, M.F.	2018	*			**			Poor
576	Tadesse, T.	2017	*			*			Poor
577	Handy, LK	2017	*			*			Poor
578	Asmare, G.	2022	*		*	*	*	*	Good
579	Dejene, H.	2022	*			*		*	Fair
580	Abdullahi MF	2020	*			*			Poor
581	Tefera YA	2018	*		*	*		*	Fair
582	Mcknight, J and Holt, DB	2014	*			*			Poor

583	Dugas M	2009	*			*			Poor
584	Antai D	2008			*	*			Poor
585	Asmare, G.	2022	*	*	*	*		*	Good
586	Opel D.J. et al.,	2013	*		*			*	Poor
587	Fleming, JA	2019	*			*	*		Fair
588	Stamidis, KV	2019	*			*			Poor
589	Umeh GC	2018	*			*			Poor
590	Closser, S	2016	*			*	*		Fair
591	Giles-Vernick, T	2016	*			*			Poor
592	Gilkey MB	2013	*		*	*		*	Fair
593	Obute JA	2007	*			*			Poor
594	Renne, E	2006				*			Poor
595	Allen, JD	2023	*		*	*	*	*	Good
596	Hill, AV	2022	*			*			Poor
597	Ali S	2022	*			*	*	*	Good
598	de St Maurice, A.	2022	*			*		*	Fair
599	Delgado, JR	2022	*			*			Poor
600	Drouin, O.	2022	*		*	*		*	Fair
601	Egbert N	2022	*			*			Poor
602	Goulding, M	2022	*			*			Poor
603	Gray A	2022	*			*		*	Fair
604	Hammershaimb, EA	2022	*			*		*	Fair

605	Head, K.J	2022	*			*			Poor
606	Temple, AM	2022	*			*	*	*	Good
607	Santibanez, TA	2022	*			*	*	*	Good
608	Wang, CH	2022	*			*		*	Fair
609	Yeo, J	2022	*			*			Poor
610	Alfieri, N. L	2021	*			*		*	Fair
611	Humble, RM	2021	*	*		**		*	Good
612	Marquez, RR	2021	*			*		*	Fair
613	McKinnon, B.	2021	*			*		*	Fair
614	Szilagyi, PG	2021	*	*		**		*	Good
615	Teasdale, CA	2021	*					*	Poor
616	Anuforo, B.	2022	*			*		*	Fair
617	Ayash, C	2022	*			*		*	Fair
618	Ayres, S	2022						*	Poor
619	Beavis, AL	2022	*		*	*			Poor
620	Dundar, Y.	2021	*					*	Poor
621	Lee, G.	2022	*			*		*	Fair
622	Rositch, A. F.	2022	*		*	*	*	*	Good
623	Thompson, EL	2022	*		*	*		*	Good
624	Tsui, J.	2022	*		*	*		*	Good
625	Varisco, T. J.	2022	*			*		*	Fair
626	Vasudevan, L.	2022	*			*		*	Fair

627	Vu, M	2022	*		*	*		*	Good
628	Myhre A	2020	*			*		*	Fair
629	Auslander BA	2019	*			*			Poor
630	Hirth JM	2019				*	*	*	Poor
631	Thomas TL	2019	*		*	*	*		Good
632	Hanson KE	2018	*			*	*	*	Good
633	Albright K	2017	*			*			Poor
634	Gilkey MB	2017	*			*		*	Fair
635	Thompson EL	2017	*			*	*	*	Fair
636	Dorell C	2014	*			**		*	Fair
637	Staras SA	2014	*		*			*	Poor
638	Dempsey AF	2009	*			*	*		Fair
639	Toffolon-Weiss M	2008	*			*			Poor
640	Fogel BN	2020	*			*		*	Fair
641	Hofstetter AM	2018	*		*	*		*	Good
642	Strelitz B	2015	*		*	*		*	Good
643	Frew PM	2011	*			**		*	Fair
644	Freeman RE	2022				*	*	*	Good
645	He, K	2022	*		*	*		*	Good
646	Kuan-Mahecha M.A. et al.,	2023	*		*	**	*	*	Good
647	Deas J	2018	*			*			poor

648	Darden PM	2013	*				*	*	Poor
649	Freed GL	2010	*			*		*	Fair
650	Gowda C	2013	*			*		*	Fair
651	Downs, JS et al	2013	*	*		*		*	Good
652	Dempsey AF, et al.	2011	*			*		*	Fair
653	Dudley MZ	2020	*		*	*		*	Good
654	Finkelstein SR	2020	*		*	*		*	Good
655	Arora G	2019	*			*		*	Fair
656	Eller NM	2019	*		*	*		*	Good
657	Gilkey MB	2016	*				*		Poor
658	Estep KA	2018	*			*	*	*	Good
659	Amin AB	2017	*		*	*		*	Good
660	Wang E	2015	*			* *			Poor
661	Glanz JM	2013	*			*	*	*	Good
662	Smith PJ	2011	*			*		*	Fair
663	Gullion JS	2008	*			*			Poor
664	Gust D. A	2008	*			*	*	*	Good
665	Fredrickson DD	2004	*			*			Poor
666	Goin-Kochel RP	2020	*		*	*	*	*	Good
667	Bardenheier B	2003	*			*		*	Fair
668	Staras, SA	2022	*		*				Poor
669	Ma, L.	2022	*			*		*	Fair

670	Ma, Y	2022	*		*	*		*	Good
671	Mohan, R.	2022	*		*	*		*	Good
672	Wang, LJ	2022	*		*	*		*	Good
673	Wang, Q (a)	2022	*		*	*		*	Good
674	Wong, WH	2022	*			*		*	Fair
675	Yang, J	2022	*		*	*		*	Good
676	Zheng, M	2022	*			*		*	Fair
677	Zheng, Z	2023	*			*		*	Fair
678	Shati, A.A.	2022	*			*		*	Fair
679	Xu, Y	2021	*			*		*	Fair
680	Yigit, M	2021	*			*		*	Fair
681	Nguyen, LH	2022	*			*		*	Fair
682	Degarege A	2018	*	*	*	*		*	Good
683	Liao, Q	2022	*			*		*	Fair
684	Zhang, H	2022	*	*		*	*	*	Good
685	Mishra, K.	2023	*	*		*	*	*	Good
686	Wu, L	2022	*	*	*	*		*	Good
687	Yalcin, SS	2022	*			*		*	Fair
688	Yalçin SS	2020	*			*			Poor
689	Li, L	2022	*	*		*		*	Good
690	Wang, X	2022	*			*		*	Fair
691	Sabra, HK	2022	*			*		*	Fair

692	Urrunaga-Pastor, D.	2021	*			*		*	Fair
693	Runngren, E.	2021	*			*			Poor
694	Price, T	2022	*			*			Poor
695	De Gioia, E.R.	2022	*			*		*	Fair
696	Brunelli L	2021	*			*			Poor
697	Bagateli, L.E.	2021	*	*	*	*		*	Good
698	Gentile	2021	*		*	*	*	*	Good
699	Burghouts J	2017	*			*	*		Fair
700	González-Block MA	2021	*			**		*	Fair
701	Brown AL	2018	*	*		*		*	Good
702	Gonzales, A.	2022	*		*	*	*	*	Good
703	Tianshuo Z	2022	*		*	*		*	Good
704	Yuen WW	2018	*			*		*	Fair
705	Zhu X	2023	*		*	*		*	Good
706	Chido-Amajuoyi, OG	2022	*				*	*	Poor
707	Xu Y	2021	*		*	*		*	Good
708	Moyer-Guse E	2018	*		*	*		*	Good
709	Egawa-Takata	2020	*			**	*	*	Good
710	Reich J A	2020	*				*		Poor
711	Rumetta J	2020	*	*	*	**			Poor

NEWCASTLE - OTTAWA QUALITY ASSESSMENT SCALE

(adapted for cross-sectional studies)

Selection: (Maximum 3 stars)

1) Representativeness of the sample:

a) Truly representative of the average in the target population. * (all subjects or random sampling)

b) Somewhat representative of the average in the target population. * (non-random sampling)

c) Selected group of users.

d) No description of the sampling strategy.

2) Non-respondents:

a) Comparability between respondents and non-respondents characteristics is established, and the response rate is satisfactory. *

b) The response rate is unsatisfactory, or the comparability between respondents and non-respondents is unsatisfactory.

c) No description of the response rate or the characteristics of the responders and the non-responders.

3) Ascertainment of the exposure (risk factor):

a) Validated measurement tool.*

b) Non-validated measurement tool, but the tool is available or described.

c) No description of the measurement tool.

Comparability: (Maximum 2 stars)

1) The subjects in different outcome groups are comparable, based on the study design or analysis.

Confounding factors are controlled.

a) The study controls for the most important factor (select one).*

b) The study control for any additional factor.*

Outcome: (Maximum 2 stars)

1) Assessment of the outcome:

a) Independent blind assessment.*

b) Record linkage.*

c) Self report.

d) No description.

2) Statistical test:

a) The statistical test used to analyze the data is clearly described and appropriate, and the measurement of the association is presented, including confidence intervals and the probability level (p value). *

b) The statistical test is not appropriate, not described or incomplete.

This scale has been adapted from the Newcastle-Ottawa Quality Assessment Scale for cohort and case-control studies to perform a quality assessment of cross-sectional studies for the systematic review, “Exposure to second-hand smoke and the risk of tuberculosis in children and adults: systematic review and a meta-analysis of 18 observational studies”. This scale was a modified version of the NOS scale, as also used by several other studies that have felt the need to adapt the NOS scale so as to appropriately assess the quality of cross-sectional studies.

We did a comprehensive search on literature and found that a NOS score of 7 or more can be considered a “good” study (see McPheeters et al. 2012; see Appendix G page 103-104 in http://www.ncbi.nlm.nih.gov/pubmedhealth/PMH0049229/). So we used this criterion as a cut off for good quality study.

References

Wells, G. A., Shea, B., O'Connell, D., Peterson, J., Welch, V., et al. The Newcastle-Ottawa Scale (NOS) for assessing the quality of nonrandomized studies in meta-analysis. 2011. http://www.ohri.ca/programs/clinical_epidemiology/oxford.asp

McPheeters, M.L., Kripalini, S., Peterson, N.B., Idowu, R.T. et al. (2012). Quality Improvement Interventions To Address Health Disparities. Evidence Report/ technology Assessment. Rockville (MD): Agency for Healthcare Research and Quality (US).

http://www.ncbi.nlm.nih.gov/pubmedhealth/PMH0049222/pdf/TOC.pd

Newcastle-Ottawa Quality Assessment Form for Cohort

No	Author	Year	Selection				Comparability	Outcome			Quality score
			S1	S2	S3	S4		O1	O2	O3	
1	Gahr, P et al	2014	*		*	*		*	*		Poor
2	Yousaf A. R.	2023	*	*	*	*	**		*	*	Good
3	Wojcicki, JM et al	2022	*	*	*		*			*	Fair
4	Arrigoni, L	2023	*			*			*	*	Fair
5	Cole JW	2022			*	*	**	*		*	Fair
6	Sugerman, DE et al	2010	*		*	*		*			Fair
7	Amdisen L	2018	*		*	*	*	*		*	Good
8	Hetherington, E	2021	*	*	*		*		*	*	Good
9	Armiento R	2020	*	*	*	*	**	*	*	*	Good
10	Brabin L	2008	*	*	*	*		*	*		Fair
11	Fair E	2002			*	*	*	*			Fair
12	Dubé E	2016		*			*	*	*		Fair
13	Delamater PL	2018	*	*	*	*	*	*	*	*	Good
14	Srivastava, T	2022	*	*	*	*	**	*		*	Good
15	Parker A.A. et al.,	2006	*		*	*	*	*			Fair
16	Dayton, L.	2022	*	*		*	**		*		Fair
17	Fisher, W.A	2022	*		*	*					Poor
18	Cui Z. et al.,	2022	*	*		*	**	*			Fair
19	Fuchs, EL	2016	*	*	*	*		*			Fair
20	Middleman A.B. et al.,	2021	*			*	**	*			Fair

21	Newcomer S.R. et al.,	2021	*		*	*		*			Fair
22	Wolf E. et al.,	2016	*		*	*		*			Fair
23	Doll, MK et al	2021	*		*	*		*	*		Good
24	Greyson D	2017	*		*	*					Poor
25	Henrikson NB	2017	*	*	*	*	*	*		*	Good
26	Betsch C	2018	*	*	*	*	**	*		*	Good
27	Gaudino JA	2012	*			*					Poor
28	Tavakoli, N	2022	*			*	*				Fair
29	Zhang MX	2021	*	*		*	*				Fair
30	Ji, M	2022	*		*	*			*		Fair
31	Carlsona, SJ	2022	*	*		*	*		*		Good
32	Smith LE	2022	*	*		*	*				Fair
33	Ceannt, R	2022	*	*		*					Fair
34	Smith, L.E.	2019	*		*	*	*		*		Good
35	Montalti, M.	2021	*	*		*	*		*		Good
36	Herdea, V	2022	*	*	*	*		*	*		Good
37	Lewandowska A	2020	*	*		*	*		*		Good
38	Anello P	2017	*		*		*	*	*		Fair
39	Braczkowska	2018	*	*		*			*		Good
40	Goldman, RD	2022	*	*		*	*		*		Good

Newcastle-Ottawa Quality Assessment Form for Cohort Studies

Note: A study can be given a maximum of one star for each numbered item within the Selection and Outcome categories. Amaximum of two stars can be given for Comparability.

Selection

1) Representativeness of the exposed cohort
   - ***a)*** Truly representative ***(one star)***
   - ***b)*** Somewhat representative ***(one star)***
   - ***c)*** Selected group
   - ***d)*** No description of the derivation of the cohort

2) Selection of the non-exposed cohort
   - ***a)*** Drawn from the same community as the exposed cohort ***(one star)***
   - ***b)*** Drawn from a different source
   - ***c)*** No description of the derivation of the non exposed cohort

3) Ascertainment of exposure
   - ***a)*** Secure record (e.g., surgical record) ***(one star)***
   - ***b)*** Structured interview ***(one star)***
   - ***c)*** Written self report
   - ***d)*** No description
   - ***e)*** Other

4) Demonstration that outcome of interest was not present at start of study
   - ***a)*** Yes ***(one star)***
   - ***b)*** No

Comparability

1) Comparability of cohorts on the basis of the design or analysis controlled for confounders
   - ***a)*** The study controls for age, sex and marital status ***(one star)***
   - ***b)*** Study controls for other factors (list)___________________________***(one star)***
   - ***c)*** Cohorts are not comparable on the basis of the design or analysis controlled for confounders

Outcome

1) Assessment of outcome
   - ***a)*** Independent blind assessment ***(one star)***
   - ***b)*** Record linkage ***(one star)***
   - ***c)*** Self report
   - ***d)*** No description
   - ***e)*** Other

2) Was follow-up long enough for outcomes to occur
   - ***a)*** Yes ***(one star)***
   - ***b)*** No
   - c)

Indicate the median duration of follow-up and a brief rationale for the assessment above:__________

3) Adequacy of follow-up of cohorts

a) Complete follow up- all subject accounted for ***(one star)***

b) Subjects lost to follow up unlikely to introduce bias- number lost less than or equal to 20% or description of those lostsuggested no different from those followed. ***(one star)***

c) Follow up rate less than 80% and no description of those lost

d) No statement

Thresholds for converting the Newcastle-Ottawa scales to AHRQ standards (good, fair, and poor):

Good quality: 3 or 4 stars in selection domain AND 1 or 2 stars in comparability domain AND 2 or 3 stars in outcome/exposure domain

Fair quality: 2 stars in selection domain AND 1 or 2 stars in comparability domain AND 2 or 3 stars in outcome/exposure domain

Poor quality: 0 or 1 star in selection domain OR 0 stars in comparability domain OR 0 or 1 stars in outcome/exposure domain

Critical Appraisal Skills Programme or CASP Randomized Clinical Trial

No	Author	Year	Section A			Section B			Section C			Section D		Appraisal Summary
			S1	S2	S3	S1	S2	S3	S1	S2	S3	S1	S2	
1	Glanz, JM	2020	Yes	Yes	Yes	Yes	Yes	Yes	Yes	Yes	Yes	Yes	Yes	Good
2	Kwan, B. M.	2022	Yes	Yes	Yes	Yes	Yes	Yes	Yes	No	Yes	Yes	Yes	Good
3	Suzuki	2022	Yes	Yes	Yes	Yes	Yes	Yes	Yes	Yes	Yes	Yes	Yes	Good
4	Tsang, TK	2022	Yes	Yes	Yes	No	No	No	Yes	Yes	No	Yes	Yes	Moderate
5	Khodadadi AB	2020	Yes	Yes	Yes	Yes	Yes	Yes	Yes	No	Yes	Yes	Yes	Good

CASP

Critical Appraisal Skills Programme

CASP Randomised Controlled Trial Standard Checklist:

11 questions to help you make sense of a randomised controlled trial (RCT)

Main issues for consideration: Several aspects need to be considered when appraising a randomised controlled trial:

- Is the basic study design valid for a randomised controlled trial? (Section A)
- Was the study methodologically sound? (Section B) What are the results? (Section C)
- Will the results help locally? (Section D)

The 11 questions in the checklist are designed to help you think about these aspects systematically.

How to use this appraisal tool: The first three questions (Section A) are screening questions about the validity of the basic study design and can be answered quickly. If, in light of your responses to Section A, you think the study design is valid, continue to Section B to assess whether the study was methodologically sound and if it is worth continuing with the appraisal by answering the remaining questions in Sections C and D.

Record 'Yes', 'No' or 'Can't tell' in response to the questions. Prompts below all but one of the questions highlight the issues it is important to consider. Record the reasons for your answers in the space provided. As CASP checklists were designed to be used as educational/teaching tools in a workshop setting, we do not recommend using a scoring system.

About CASP Checklists: The CASP RCT checklist was originally based on JAMA Users' guides to the medical literature 1994 (adapted from Guyatt GH, Sackett DL and Cook DJ), and piloted with healthcare practitioners. This version has been updated taking into account the CONSORT 2010 guideline (http://www.consort-statement.org/consort-2010, accessed 16 September 2020).

Citation: CASP recommends using the Harvard style, i.e. *Critical Appraisal Skills Programme (2020). CASP (insert name of checklist i.e. Randomised Controlled Trial) Checklist. [online] Available at: insert URL. Accessed: insert date accessed.*

©CASP this work is licensed under the Creative Commons Attribution – Non-Commercial- Share A like. To view a copy of this licence, visit https://creativecommons.org/licenses/by-sa/4.0/

Study and citation: ....

Section A: Is the basic study design valid for a randomised [illegible]

**1.** Did the study address a clearly focused research question?<br>*CONSIDER:*<br>*Was the study designed to assess the outcomes of an intervention?*<br>*Is the research question 'focused' in terms of:*<br>• *Population studied*<br>• *Intervention given*<br>• *Comparator chosen*<br>• *Outcomes measured?*	Yes ☐ No ☐ Can't tell ☐
2. Was the assignment of participants to interventions randomised? *CONSIDER:*<br>• *How was randomisation carried out? Was the method appropriate?*<br>• *Was randomisation sufficient to eliminate systematic bias?*<br>• *Was the allocation sequence concealed from investigators and participants?*	Yes ☐ No ☐ Can't tell ☐
**3.** Were all participants who entered the study accounted for at its conclusion?<br>*CONSIDER:*<br>• *Were losses to follow-up and exclusions after randomisation accounted for?*<br>• *Were participants analysed in the study groups to which they were randomised (intention-to-treat analysis)?*<br>• *Was the study stopped early? If so, what was the reason?*	Yes ☐ No ☐ Can't tell ☐

Section B: Was the study methodologically sound?

4.<br>• Were the participants 'blind' to intervention they were given?<br>• Were the investigators 'blind' to the intervention they were giving to participants?<br>• Were the people assessing/analysing outcome/s 'blinded'?	Yes ☐ No ☐ Can't tell ☐<br>☐ ☐ ☐<br>☐ ☐ ☐<br>☐ ☐ ☐

5. Were the study groups similar at the start of the randomised controlled trial?<br>*CONSIDER:*<br>• *Were the baseline characteristics of each study group (e.g. age, sex, socio-economic group) clearly set out?*<br>• *Were there any differences between the study groups that could affect the outcome/s?*	Yes ☐ No ☐ Can't tell ☐

**6.** Apart from the experimental intervention, did each study group receive the same level of care (that is, were they treated equally)?<br><br>*CONSIDER:*<br>• *Was there a clearly defined study protocol?*<br>• *If any additional interventions were given (e.g. tests or treatments), were they similar between the study groups?*<br>• *Were the follow-up intervals the same for each study group?*	Yes ☐ No ☐ Can't tell ☐

Section C: What are the results?

**7.** Were the effects of intervention reported comprehensively?<br>*CONSIDER:*<br>• *Was a power calculation undertaken?*<br>• *What outcomes were measured, and were they clearly specified?*<br>• *How were the results expressed? For binary outcomes, were relative and absolute effects reported?*<br>• *Were the results reported for each outcome in each study group at each follow-up interval?*<br>• *Was there any missing or incomplete data?*<br>• *Was there differential drop-out between the study groups that could affect the results?*<br>• *Were potential sources of bias identified?*<br>• *Which statistical tests were used?*<br>• *Were p values reported?*	Yes ☐ No ☐ Can't tell ☐
**8.** Was the precision of the estimate of the intervention or treatment effect reported?<br><br>*CONSIDER:*<br>• *Were confidence intervals (CIs) reported?*	Yes ☐ No ☐ Can't tell ☐

☐ ☐ ☐

9. Do the benefits of the experimental intervention outweigh the harms and costs?<br><br>*CONSIDER:*<br>• *What was the size of the intervention or treatment effect?*<br>• *Were harms or unintended effects reported for each study group?*<br>• *Was a cost-effectiveness analysis undertaken? (Cost-effectiveness analysis allows a comparison to be made between different interventions used in the care of the same condition or problem.)*	Yes ☐ No ☐ Can't tell ☐

Section D: Will the results help locally?

10.	Can the results be applied to your local population/in your context?<br><br>*CONSIDER:*<br>• *Are the study participants similar to the people in your care?*<br>• *Would any differences between your population and the study participants alter the outcomes reported in the study?*<br>• *Are the outcomes important to your population?*<br>• *Are there any outcomes you would have wanted information on that have not been studied or reported?*<br>• *Are there any limitations of the study that would affect your decision?*	Yes ☐ No ☐ Can't tell ☐
11.	Would the experimental intervention provide greater value to the people in your care than any of the existing interventions?<br><br>*CONSIDER:*<br>• *What resources are needed to introduce this intervention taking into account time, finances, and skills development or training needs?*<br>• *Are you able to disinvest resources in one or more existing interventions in order to be able to re-invest in the new intervention?*	Yes ☐ No ☐ Can't tell ☐

APPRAISAL SUMMARY: *Record key points from your critical appraisal in this box. What is your conclusion about the paper? Would you use it to change your practice or to recommend changes to care/interventions used by your organisation? Could you judiciously implement this intervention without delay?*
