# Supplementary material for: Prevalence of caregiver hesitancy for vaccinations in children and its associated factors: A systematic review and meta-analysis
Source: PLoS One. 2024 Oct 24;19(10):e0302379. doi: 10.1371/journal.pone.0302379 (PMC11500859; doi:10.1371/journal.pone.0302379)
Supplement: S5 Table — (PDF) [file pone.0302379.s009.pdf]

S5 Table: Prevalence of vaccine hesitancy among parents across the region

No	Author	Year	Region	Total respondent	Hesitancy	Prevalence*	Lower CI	Upper CI	Vaccine
1	Kyei-Arthur F	2022	Africa	415	110	0.267	0.223198	0.310309	Covid-19
2	Balogun FM	2022	Africa	678	22	0.032	0.020445	0.048717	HPV
3	Kolek CO	2022	Africa	341	34	0.1	0.070043	0.136539	HPV
4	Kamya C	2022	Africa	590	6	0.01	0.003741	0.022003	Mix
5	Darebo TD	2022	Africa	657	74	0.113	0.089898	0.139958	Mix
6	Abor J	2022	Africa	420	55	0.13	0.100197	0.16702	Mix
7	Griffith BC	2022	Africa	999	151	0.151	0.129492	0.174888	MMR
8	Sabahelzain MM	2022	Africa	500	60	0.12	0.0928	0.1518	MMR
9	Tefera YA	2018	Africa	540	292	0.54	0.4977	0.5834	Not stated
10	Immurana M	2021	Africa	3004	162	0.054	0.046122	0.062616	Others
11	Obute JA	2007	Africa	422	31	0.07	0.0505	0.1027	Others
12	Mohammed A	2014	Africa	360	211	0.56	0.5333	0.6375	Others
13	Asmare G	2022	Africa	406	274	0.677	0.62693	0.720252	Others
14	Cockcroft, A	2014	Africa	2836	458	0.161	0.14812	0.175562	MMR
15	Helman, CG	2004	Africa	60	13	0.21	0.120	0.341	Mix
16	Powelson, J	2022	Africa	32	13	0.40	0.236	0.593	Mix
17	Milondzo T. et al.,	2022	Africa	320	196	0.61	0.556	0.666	HPV
18	Sulaiman S.K. et al.,	2023	Africa	3377	1010	0.29	0.283	0.314	Others
19	Abuhammad S	2022	Asia	1078	241	0.224	0.199009	0.249634	Covid-19
20	Aedh AI	2022	Asia	464	72	0.155	0.123437	0.191379	Covid-19
21	Akgün Ö	2022	Asia	540	67	0.12	0.0975	0.1549	Covid-19
22	Aldakhil H	2021	Asia	270	119	0.44	0.380609	0.502187	Covid-19
23	Ali M	2022	Asia	2633	170	0.0646	0.055477	0.074636	Covid-19
24	Al-Iede M	2022	Asia	2628	1216	0.46	0.443506	0.481995	Covid-19
25	Al-Khlaiwi T	2022	Asia	1304	382	0.293	0.268349	0.318477	Covid-19
26	Almansour A	2022	Asia	500	120	0.24	0.203197	0.27991	Covid-19
27	Almusbah Z	2021	Asia	1000	346	0.346	0.316507	0.376403	Covid-19
28	Al-Qahtani AM	2022	Asia	528	203	0.3844	0.342776	0.427464	Covid-19
29	Al-Qerem W	2022	Asia	491	175	0.356	0.31401	0.400562	Covid-19
30	Al-Qerem W	2022	Asia	819	396	0.484	0.448793	0.518359	Covid-19
31	Altulaihi BA	2021	Asia	333	90	0.27	0.223301	0.321379	Covid-19
32	Bord S	2022	Asia	581	62	0.107	0.082799	0.134708	Covid-19
33	Çağ Y	2022	Asia	1440	26	0.018	0.0118	0.0263	Covid-19
34	Choi UI	2022	Asia	1217	292	0.24	0.216183	0.264949	Covid-19
35	Duong AH	2022	Asia	5357	176	0.033	0.028243	0.037983	Covid-19
36	ElSayed DA	2022	Asia	223	125	0.5625	0.492717	0.626728	Covid-19
37	Hou Z	2022	Asia	3897	386	0.099	0.089846	0.108861	Covid-19
38	Huang LL	2022	Asia	514	37	0.072	0.051187	0.097858	Covid-19
39	Khan YH	2022	Asia	444	95	0.214	0.176706	0.255082	Covid-19
40	Kharaba Z	2022	Asia	1049	307	0.293	0.265264	0.321224	Covid-19
41	Khatatbeh M	2022	Asia	3744	1361	0.364	0.348085	0.379158	Covid-19
42	Kocamaz EB	2022	Asia	384	103	0.268	0.224551	0.315522	Covid-19
43	Lau EY	2022	Asia	11141	9425	0.846	0.839136	0.852631	Covid-19
44	Lee M	2022	Asia	113450	43174	0.381	0.377729	0.383388	Covid-19
45	Li T	2022	Asia	3342	66	0.02	0.015306	0.025057	Covid-19
46	Lu L	2022	Asia	868	208	0.23	0.211582	0.269461	Covid-19
47	Ma Y	2022	Asia	424	165	0.389	0.34248	0.437381	Covid-19
48	Mohammed AH	2022	Asia	1154	508	0.44	0.411317	0.469404	Covid-19
49	Mohan R	2022	Asia	204	31	0.15	0.105645	0.208709	Covid-19
50	Morozov NG	2022	Asia	1514	848	0.56	0.540927	0.586794	Covid-19
51	Ng DL	2022	Asia	3528	486	0.138	0.126545	0.14957	Covid-19
52	Padhi BK	2021	Asia	770	164	0.213	0.184564	0.243627	Covid-19
53	Parinyarux P	2022	Asia	488	45	0.092	0.068057	0.121441	Covid-19
54	Qin C	2022	Asia	1724	199	0.1154	0.10072	0.131461	Covid-19
55	Reagu S	2022	Asia	6882	2023	0.294	0.283208	0.304877	Covid-19
56	Samannodi M	2021	Asia	581	22	0.038	0.02388	0.05677	Covid-19
57	Samudyatha UC	2022	Asia	272	18	0.066	0.039689	0.102573	Covid-19
58	Savitsky B	2022	Asia	138	37	0.266	0.196326	0.350136	Covid-19
59	Shati AA	2022	Asia	1463	187	0.128	0.111129	0.14602	Covid-19
60	Shmueli L	2021	Asia	1012	233	0.23	0.204621	0.257433	Covid-19
61	Swed S	2022	Asia	283	127	0.449	0.389855	0.508756	Covid-19
62	Tung TH	2022	Asia	1788	606	0.339	0.316986	0.361397	Covid-19
63	Wan X	2021	Asia	468	62	0.1325	0.103101	0.166593	Covid-19
64	Wang L	2022	Asia	2019	179	0.089	0.076616	0.101909	Covid-19
65	Wang Q	2022	Asia	5102	447	0.08	0.079994	0.095707	Covid-19
66	Wong LP	2022	Asia	1003	46	0.046	0.033702	0.060583	Covid-19
67	Wong WH	2022	Asia	545	183	0.34	0.296197	0.377155	Covid-19
68	Yang J	2022	Asia	12872	566	0.044	0.040494	0.047657	Covid-19

69	Yigit M	2021	Asia	428	344	0.804	0.762894	0.840323	Covid-19
70	Zhang Z	2023	Asia	1788	948	0.53	0.5068	0.553549	Covid-19
71	Zheng M	2022	Asia	2624	131	0.05	0.041907	0.058963	Covid-19
72	Zhou M	2022	Asia	1602	203	0.127	0.11081	0.144005	Covid-19
73	Zhou Y	2021	Asia	1071	914	0.853	0.825274	0.877392	Covid-19
74	Akca G	2022	Asia	330	108	0.327	0.276885	0.380795	HPV
75	Choi J	2022	Asia	906	256	0.283	0.253438	0.313105	HPV
76	Frianto D	2022	Asia	286	200	0.699	0.642534	0.751882	HPV
77	Pearl CA	2022	Asia	45	33	0.73	0.580553	0.853959	HPV
78	Huang Y	2020	Asia	1125	1,068	0.95	0.9349	0.961403	HPV
79	Akis S	2011	Asia	611	386	0.63	0.5921	0.6701	Influenza
80	Alabbad AA	2018	Asia	100	17	0.17	0.1023	0.2582	Influenza
81	Alenazi KA	2022	Asia	539	18	0.033	0.01991	0.052267	Influenza
82	Alolayan A	2019	Asia	399	21	0.053	0.0329	0.07933	Influenza
83	AlOmran HI	2022	Asia	510	382	0.75	0.709031	0.786093	Influenza
84	Fan J	2022	Asia	5016	420	0.0837	0.076211	0.091742	Influenza
85	Hussein YH	2022	Asia	287	179	0.624	0.564856	0.679948	Influenza
86	Lai X	2022	Asia	6668	360	0.054	0.048686	0.059687	Influenza
87	Liao Q	2022	Asia	291	84	0.29	0.237267	0.344415	Influenza
88	Salawati E	2021	Asia	2501	378	0.15	0.137317	0.165785	Influenza
89	Zakhour R	2021	Asia	306	214	0.7	0.6446	0.750205	Influenza
90	Zhang H	2022	Asia	7323	3713	0.507	0.495513	0.518547	Influenza
91	Abu-Rish EY	2016	Asia	1107	362	0.33	0.2994	0.3555	Mix
92	Alsubaie SS	2019	Asia	500	100	0.2	0.1658	0.2378	Mix
93	Chan HK	2018	Asia	117429	705	0.0058	0.0054	0.0063	Mix
94	Chang K	2019	Asia	129	34	0.26	0.1899	0.34837	Mix
95	Choi SH	2021	Asia	226	81	0.36	0.2959	0.4247	Mix
96	Dasgupta P	2018	Asia	194	161	0.83	0.76949	0.879922	Mix
97	Han K	2022	Asia	2081	1173	0.5637	0.542046	0.585117	Mix
98	Hijazi R	2022	Asia	18	11	0.61	0.357451	0.827014	Mix
99	Khaliq A	2017	Asia	484	153	0.32	0.2749	0.3596	Mix
100	Khaliq A	2022	Asia	230	141	0.613	0.546795	0.676338	Mix
101	Özer M	2022	Asia	78	22	0.225	0.185938	0.395331	Mix
102	Shaipuzaman NA	2022	Asia	97	4	0.041	0.011348	0.102228	Mix
103	Tal O	2021	Asia	1010	36	0.036	0.025087	0.049006	Mix
104	Temsah MH	2021	Asia	3167	1013	0.32	0.303631	0.336424	Mix
105	Tianshuo Z	2022	Asia	199	5	0.025	0.008208	0.057658	Mix
106	Wu L	2022	Asia	1691	515	0.304	0.282678	0.32711	Mix
107	Yalçin SS	2022	Asia	23756	1401	0.059	0.055967	0.061996	Mix
108	Ashkenazi S	2020	Asia	399	92	0.23	0.1901	0.275076	MMR
109	Aharony N	2017	Asia	210	101	0.48	0.4117	0.5508	Not stated
110	Akhmetzhanova Z	2020	Asia	442	155	0.35	0.3062	0.3972	Not stated
111	AlGoraini YM	2022	Asia	384	57	0.15	0.1144	0.188	Not stated
112	Alsuwaidi AR	2020	Asia	300	36	0.12	0.0855	0.162244	Not stated
113	Çağ Y	2022	Asia	1184	238	0.201	0.178529	0.224993	Not stated
114	Azizi FS	2017	Asia	545	17	0.032	0.0201	0.049	Not stated
115	Gunes NA	2020	Asia	614	18	0.029	0.0175	0.045937	Not stated
116	Hu Y	2019	Asia	770	92	0.12	0.0974	0.144508	Not stated
117	Kalok A	2020	Asia	1081	86	0.08	0.0641	0.09732	Not stated
118	Noyman-Veksler G	2020	Asia	555	115	0.21	0.1742	0.2433	Not stated
119	Yörük S	2021	Asia	370	18	0.048	0.0291	0.0758	Not stated
120	Ali HYM	2004	Asia	254	41	0.16	0.1184	0.2125	Others
121	Al-Iede M	2022	Asia	720	222	0.308	0.274751	0.343495	Others
122	Gesser-Edelsburg A	2016	Asia	196	25	0.13	0.0843	0.182509	Others
123	Khan TM	2016	Asia	1775	293	0.17	0.1481	0.1832	Others
124	Khowaja AR	2012	Asia	1017	712	0.7	0.6800	0.7192	Others
125	Li L	2022	Asia	1254	101	0.0805	0.066079	0.097006	Others
126	Qi L	2019	Asia	1080	482	0.45	0.4164	0.4765	Others
127	Imanishi Y	2022	Asia	161	60	0.37	0.297	0.542	HPV
128	Yagi	2018	Asia	4260	3843	0.90	0.892	0.910	HPV
129	Hanley S	2012	Asia	862	64	0.07	0.057	0.093	HPV
130	Hanley	2014	Asia	27	2	0.074	0.009	0.242	HPV
131	Egawa-Takata	2015	Asia	2828	176	0.06	0.053	0.717	HPV
132	Shuto	2021	Asia	1646	643	0.39	0.366	0.414	HPV
133	Kobayashi	2020	Asia	242	111	0.46	0.394	0.523	HPV
134	Miyoshi	2020	Asia	1648	626	0.37	0.356	0.403	HPV
135	Egawa-Takata	2020	Asia	450	233	0.518	0.470	0.564	HPV
136	Ugumori	2021	Asia	59	1	0.02	0.0004	0.090	HPV
137	Suzuki	2022	Asia	2175	1984	0.912	0.899	0.923	HPV
138	Çelik, M.Y.	2021	Asia	274	128	0.467	0.406	0.528	Covid-19
139	Wang, Z.	2021	Asia	1332	739	0.555	0.527	0.581	Covid-19

140	Altulahi, N.	2021	Asia	3038	1446	0.476	0.458	0.493	Covid-19
141	Feng, H	2021	Asia	3703	596	0.161	0.149	0.173	Covid-19
142	Al-Nafeesah AS	2021	Asia	1143	297	0.26	0.234	0.286	Mix
143	Hou, Z.	2021	Asia	1655	158	0.09	0.081	0.110	Mix
144	Zhou T	2023	Asia	2630	205	0.078	0.067	0.888	Covid-19
145	Zhang H	2023	Asia	346	39	0.11	0.081	0.150	Covid-19
146	Zhang K	2023	Asia	437	38	0.08	0.062	0.117	Covid-19
147	Ahmed, N	2023	Asia	244	90	0.369	0.308	0.432	Mix
148	Bourguiba, A	2023	Asia	437	225	0.515	0.466	0.562	Covid-19
149	Al-Qerem, W	2023	Asia	667	403	0.604	0.565	0.641	Influenza
150	Ni, Y-H.	2023	Asia	1051	403	0.383	0.353	0.413	Others
151	Alaamri, O.	2022	Asia	2030	213	0.10	0.091	0.119	Not stated
152	Alharbi, I.	2022	Asia	334	18	0.054	0.0322	0.083	Influenza
153	Xie, H.	2023	Asia	1431	216	0.151	0.132	0.170	HPV
154	Alhuzaimi, AN	2023	Asia	873	179	0.20	0.178	0.233	Covid-19
155	Alkhalas, SH	2022	Asia	343	52	0.152	0.115	0.194	HPV
156	Almuqbil, M.	2023	Asia	699	174	0.248	0.217	0.282	Covid-19
157	Topaktas B.	2022	Asia	338	15	0.044	0.025	0.072	Mix
158	Ashour, HA	2023	Asia	293	183	0.62	0.566	0.680	Covid-19
159	Sobierajski, T.	2023	Asia	360	15	0.04	0.023	0.067	HPV
160	Wang, X.	2021	Asia	941	471	0.501	0.468	0.532	Covid-19
161	Babi A.	2023	Asia	141	63	0.44	0.363	0.532	HPV
162	Sahoo S. S.	2023	Asia	196	10	0.05	0.024	0.091	Mix
163	Chawanpaiboon S.	2023	Asia	400	28	0.07	0.047	0.099	Covid-19
164	Tsai, C-S. et al	2022	Asia	252	4	0.01	0.004	0.040	Covid-19
165	Cho, HK. et at	2022	Asia	1019	444	0.43	0.405	0.466	Covid-19
166	Choi, J.	2023	Asia	10	6	0.6	0.262	0.878	HPV
167	Yoda, T. et al.,	2021	Asia	1100	158	0.14	0.123	0.165	Covid-19
168	Dao, T.L. et al.,	2023	Asia	602	104	0.172	0.143	0.205	Covid-19
169	Deng, JS et al	2023	Asia	384	246	0.68	0.638	0.733	Covid-19
170	Du, Y et al	2022	Asia	1110	121	0.10	0.091	0.128	Others
171	Fakhruddin TM. et al	2023	Asia	375	189	0.50	0.452	0.555	Not stated
172	Ghazy, RM et al	2023	Asia	321	205	0.63	0.583	0.691	Covid-19
173	Al Yamani, Z.J. et al.,	2022	Asia	375	262	0.699	0.649	0.744	Mix
174	Mohd Zin Z. et al	2022	Asia	27	24	0.88	0.708	0.976	Mix
175	Maneesriwongul W. et al.,	2023	Asia	455	62	0.13	0.106	0.171	Covid-19
176	Khatrawi, EM and Sayed, AA	2023	Asia	344	73	0.21	0.17	0.259	Covid-19
177	Khoodoruth, MAS et al	2023	Asia	433	158	0.36	0.319	0.412	Covid-19
178	Horiuchi, S. et al.,	2021	Asia	1200	424	0.35	0.326	0.381	Covid-19
179	Aljamaan, F. et al.,	2022	Asia	1340	911	0.68	0.654	0.704	Covid-19
180	Yulan Lin et al	2021	Asia	2026	1573	0.77	0.757	0.794	Covid-19
181	Lu X	2021	Asia	13451	981	0.073	0.068	0.077	Covid-19
182	Wagner A	2022	Europe	1318	54	0.041	0.030926	0.053123	Covid-19
183	Bianco A	2022	Europe	394	49	0.124	0.09344	0.161067	Covid-19
184	Ceannt R	2022	Europe	482	86	0.178	0.145267	0.215583	Covid-19
185	Krakowczyk JB	2022	Europe	2405	464	0.193	0.177334	0.20928	Covid-19
186	Manolescu LS	2022	Europe	1645	334	0.203	0.18384	0.223305	Covid-19
187	Iannello P	2022	Europe	415	86	0.207	0.169235	0.249456	Covid-19
188	Lecce M	2022	Europe	604	138	0.23	0.195562	0.264072	Covid-19
189	Miraglia del Giudice G	2022	Europe	430	104	0.24	0.20212	0.285194	Covid-19
190	Babicki M	2021	Europe	4432	1142	0.258	0.244846	0.270817	Covid-19
191	Musa S	2021	Europe	4023	1045	0.2597	0.24626	0.273602	Covid-19
192	Skirrow H	2022	Europe	1404	176	0.125	0.108478	0.143819	Covid-19
193	Mărcău FC	2022	Europe	581	188	0.323	0.285658	0.363308	Covid-19
194	Galanis P	2021	Europe	813	272	0.335	0.302165	0.368167	Covid-19
195	Napoli A	2022	Europe	358	155	0.433	0.38098	0.48606	Covid-19
196	Steletou E	2022	Europe	439	199	0.453	0.406048	0.501191	Covid-19
197	Miliordos K	2022	Europe	366	190	0.519	0.466596	0.571343	Covid-19
198	Muluye M	2022	Europe	874	482	0.551	0.517816	0.58481	Covid-19
199	Galanis P	2022	Europe	813	593	0.729	0.697448	0.759673	Covid-19
200	Fedele F	2021	Europe	640	467	0.73	0.69349	0.76375	Covid-19
201	Koźlarek M	2022	Europe	402	101	0.25	0.209568	0.296625	Covid-19
202	Taylor J	2022	Europe	138	5	0.036	0.011867	0.082526	HPV
203	Kornfeld J	2013	Europe	224	13	0.06	0.03126	0.097198	HPV
204	Runngren E	2021	Europe	20	2	0.1	0.012349	0.316983	HPV
205	Hontelez JA	2010	Europe	203	32	0.16	0.11039	0.215185	HPV
206	Naoum P	2022	Europe	1000	328	0.328	0.298944	0.358073	HPV
207	Navarro-Illana P	2015	Europe	833	192	0.23	0.202287	0.260618	HPV
208	Della Polla G	2020	Europe	435	144	0.33	0.286943	0.377443	HPV
209	Di Giuseppe G	2022	Europe	574	181	0.315	0.277483	0.355093	Influenza

210	Gorman DR	2020	Europe	128	54	0.42	0.335144	0.512305	Influenza
211	Bielecki K	2020	Europe	65	5	0.07	0.0327	0.1293	Influenza
212	Prospero E	2019	Europe	366	358	0.95	0.9224	0.9694	Influenza
213	Miron VD	2022	Europe	2550	27	0.01	0.006989	0.015368	Mix
214	Skitarelić N	2022	Europe	300	5	0.0166	0.005433	0.0384	Mix
215	Harmsen IA	2013	Europe	592	12	0.02	0.010517	0.03514	Mix
216	Byström E	2020	Europe	825	17	0.02	0.012049	0.032788	Mix
217	Dáňová J	2015	Europe	480	10	0.02	0.010035	0.037979	Mix
218	Durmaz N	2022	Mix	1087	38	0.0356	0.024855	0.04767	Mix
219	Fonseca IC	2021	Europe	886	32	0.036	0.024833	0.050607	Mix
220	Whelan SO	2021	Europe	436	22	0.05	0.031889	0.075401	Mix
221	Lewandowska A	2020	Europe	1257	136	0.108	0.091556	0.126689	Mix
222	Napolitano F	2018	Europe	437	79	0.18	0.145818	0.220127	Mix
223	Van Hoecke AL	2022	Europe	538	139	0.258	0.22186	0.297547	Mix
224	Miko D	2019	Europe	452	190	0.43	0.3744	0.4674	Mix
225	Caso D	2021	Europe	447	223	0.5	0.451551	0.546226	Mix
226	Buonsenso D	2022	Europe	121	15	0.12	0.071073	0.196211	Mix
227	Herdea V	2022	Europe	5640	448	0.078	0.072506	0.08679	Mix
228	Tho SL	2015	Europe	1270	599	0.47	0.443898	0.499541	Mix
229	Anello P	2017	Europe	86255	1647	0.0191	0.018192	0.02003	MMR
230	Campbell H	2017	Europe	1792	36	0.02	0.0143	0.027704	MMR
231	Alfredsson R	2004	Europe	300	33	0.11	0.0769	0.151	MMR
232	Restivo V	2015	Europe	443	66	0.15	0.1189	0.184	MMR
233	Byström E	2014	Europe	20	11	0.55	0.3153	0.7694	MMR
234	Roberts RJ	1995	Europe	307	233	0.76	0.707114	0.805727	MMR
235	Giambi C	2017	Europe	3130	22	0.007	0.00441	0.010622	Not stated
236	Bianco A	2019	Europe	575	44	0.07	0.0561	0.1014	Not stated
237	Bocquier A	2018	Europe	15216	3956	0.26	0.253034	0.267037	Not stated
238	Bertoncello C	2020	Europe	3685	1327	0.36	0.3446	0.3759	Not stated
239	Bankiewicz P	2022	Europe	53	20	0.37	0.247872	0.521061	Others
240	Allaert F-A	2009	Europe	2593	570	0.22	0.204014	0.236267	Others
241	Gács Z	2022	Europe	430	108	0.251	0.210843	0.294945	Others
242	Huber A	2020	Europe	1042	479	0.46	0.429104	0.490511	Others
243	Gjini, E.	2023	Europe	475	23	0.04	0.030	0.071	Mix
244	Seiler, M.	2021	Europe	622	549	0.88	0.854	0.906	Covid-19
245	Smith, L.E.	2022	Europe	270	60	0.22	0.174	0.276	Covid-19
246	Ates, BO	2023	Europe	284	9	0.03	0.014	0.059	Covid-19
247	Avci, D	2023	Europe	381	114	0.29	0.253	0.347	Others
248	Sherman, S.M.	2023	Europe	596	42	0.07	0.051	0.094	Others
249	Yılmaz, M.	2021	Europe	1035	659	0.637	0.606	0.666	Covid-19
250	Savarese G.	2022	Europe	1105	987	0.894	0.873	0.910	Covid-19
251	Bag, O and Guney, SA	2023	Europe	110	44	0.4	0.307	0.497	Not stated
252	Bektas, I and Bektas, M	2023	Europe	199	40	0.20	0.147	0.263	Covid-19
253	Celik, T and Dogan, D	2023	Europe	102	47	0.46	0.361	0.562	Covid-19
254	Duran, S et al	2023	Europe	610	107	0.175	0.146	0.207	Mix
255	Sasic M. et al.,	2023	Europe	872	306	0.351	0.319	0.383	Covid-19
256	Sahin, A. et al.,	2023	Europe	396	165	0.417	0.367	0.466	Covid-19
257	Esposito, S. et al	2023	Europe	3433	751	0.21	0.305	0.232	Covid-19
258	Grechukha, YO	2023	Europe	797	113	0.142	0.118	0.167	Not stated
259	Gundogdu, Z and Sezer, OY	2023	Europe	564	344	0.60	0.568	0.650	Others
260	Zona, S. et al.,	2021	Europe	1799	796	0.44	0.419	0.465	Covid-19
261	Russo, L. et al.,	2021	Europe	1205	403	0.33	0.307	0.361	Covid-19
262	Marron, L. et al	2023	Europe	855	61	0.07	0.055	0.907	Mix
263	Halima Y.A. et al.,	2023	Europe	183	119	0.65	0.576	0.719	Influenza
264	Goldman RD	2020	Mix	1005	332	0.33	0.301305	0.360389	Covid-19
265	Goldman, RD	2022	Mix	882	171	0.194	0.168278	0.22154	Covid-19
266	Goldman, RD	2022	Mix	1956	1226	0.627	0.604923	0.648275	Covid-19
267	Urrunaga-Pastor D	2021	Mix	227740	17763	0.078	0.076899	0.079106	Covid-19
268	Sabra HK	2022	Mix	1302	511	0.393	0.365834	0.419599	Covid-19
269	Yilmaz M	2023	Mix	94	14	0.149	0.083	0.237	Mix
270	Tekin C.	2023	Mix	1100	123	0.112	0.093	0.131	Mix
271	Hill, AV	2022	North America	299	81	0.27	0.221349	0.325061	Covid- 19
272	Skeens, M.	2022	North America	113	18	0.16	0.097226	0.240018	Covid-19
273	Guerin RJ	2022	North America	7298	1387	0.19	0.181107	0.199245	Covid-19
274	Suvada KA	2022	North America	6068	508	0.084	0.076868	0.090971	Covid-19
275	Hetherington E	2021	North America	1321	113	0.086	0.071016	0.101941	Covid-19
276	Hopfer S	2022	North America	46	5	0.11	0.036248	0.235697	Covid-19
277	McKinnon B	2021	North America	809	100	0.124	0.101716	0.148287	Covid-19
278	Limbers CA	2022	North America	821	35	0.043	0.029871	0.058793	Covid-19
279	Willis DE	2022	North America	665	73	0.11	0.087038	0.13604	Covid-19

280	Bonuck K	2022	North America	352	53	0.15	0.114865	0.192276	Covid-19
281	Teasdale CA	2021	North America	1119	18	0.016	0.009561	0.025304	Covid-19
282	Schiff J	2022	North America	58	9	0.155	0.073493	0.274232	Covid-19
283	McElfish PA	2022	North America	369	63	0.17	0.13376	0.213084	Covid-19
284	Lessard L	2022	North America	168	30	0.179	0.123844	0.245002	Covid-19
285	Skeens MA	2022	North America	491	90	0.183	0.150052	0.220414	Covid-19
286	Wang CH	2022	North America	121	21	0.174	0.110761	0.252977	Covid-19
287	Drouin O	2022	North America	305	58	0.191	0.147683	0.23878	Covid-19
288	Panchalingam T	2022	North America	1456	296	0.203	0.182896	0.224901	Covid-19
289	Humble RM	2022	North America	1129	230	0.204	0.18058	0.228415	Covid-19
290	Byrne A	2022	North America	2620	576	0.22	0.204119	0.236205	Covid-19
291	Teasdale CA	2021	North America	2074	531	0.256	0.237358	0.275388	Covid-19
292	Cioffredi LA	2022	North America	78	16	0.2	0.122037	0.31163	Covid-19
293	Reindl D	2022	North America	582	157	0.27	0.234088	0.307787	Covid-19
294	Scherer AM	2022	North America	2031	571	0.281	0.261672	0.301246	Covid-19
295	Teasdale CA	2022	North America	1119	166	0.148	0.12802	0.170542	Covid-19
296	de St Maurice A	2022	North America	12288	3686	0.3	0.291873	0.308157	Covid-19
297	Dayton L	2022	North America	297	93	0.31	0.260804	0.369234	Covid-19
298	Szilagyi PG	2021	North America	1745	574	0.329	0.306912	0.351544	Covid-19
299	O’Dor SL	2022	North America	254	51	0.2	0.153307	0.255399	Covid-19
300	Goldman, RD	2022	North America	2687	906	0.34	0.319304	0.35541	Covid-19
301	Baumer-Mouradian SH	2022	North America	589	295	0.5	0.459703	0.541986	Covid-19
302	Santibanez TA	2022	North America	4496	935	0.208	0.196181	0.220125	Covid-19
303	Marquez RR	2021	North America	97	38	0.392	0.294176	0.496125	Covid-19
304	Mangat C	2022	North America	1301	516	0.397	0.369911	0.423794	Covid-19
305	Delgado JR	2022	North America	1051	438	0.417	0.386733	0.447227	Covid-19
306	Head KJ	2022	North America	10266	4332	0.422	0.412397	0.431599	Covid-19
307	Fisher CB	2022	North America	411	190	0.462	0.413293	0.511829	Covid-19
308	Lam CN	2022	North America	401	206	0.514	0.463604	0.563624	Covid-19
309	Schilling S	2022	North America	50	31	0.62	0.471749	0.753499	Covid-19
310	Temple AM	2022	North America	204	140	0.68	0.617774	0.749265	Covid-19
311	Nguyen KH	2022	North America	11478	1435	0.125	0.119023	0.131211	Covid-19
312	Humble RM	2021	North America	1702	628	0.369	0.346002	0.392406	Covid-19
313	Hammershaimb EA	2022	North America	3230	1304	0.404	0.386734	0.420871	Covid-19
314	Salazar TL	2022	North America	93	14	0.15	0.084816	0.239661	Covid-19
315	Srivastava T	2022	North America	334	23	0.069	0.04415	0.101538	Covid-19
316	Brabin L	2008	North America	2817	228	0.08	0.071127	0.091628	HPV
317	Myhre A	2020	North America	355	45	0.13	0.093983	0.165918	HPV
318	Dorell C	2014	North America	4103	86	0.021	0.016799	0.025822	HPV
319	Beavis A	2018	North America	46853	10776	0.23	0.226192	0.233834	HPV
320	Lee YM	2018	North America	74	19	0.26	0.162204	0.371565	HPV
321	Gilkey MB	2017	North America	1484	416	0.28	0.257588	0.303932	HPV
322	Thompson EL	2017	North America	59897	18867	0.315	0.311271	0.318728	HPV
323	Dundar Y	2022	North America	150	49	0.327	0.252404	0.407933	HPV
324	Ogilvie G	2010	North America	2025	717	0.354	0.333222	0.37535	HPV
325	Khodadadi AB	2020	North America	317	111	0.35	0.297688	0.405458	HPV
326	Staras SAS	2022	North America	25	9	0.36	0.179717	0.574794	HPV
327	Dempsey AF	2009	North America	52	19	0.36	0.236244	0.510441	HPV
328	Thompson EL	2022	North America	1192	463	0.388	0.360639	0.416759	HPV
329	Krok-Schoen JL	2018	North America	337	131	0.39	0.336377	0.443045	HPV
330	Hirth JM	2019	North America	90866	57427	0.63	0.628851	0.635134	HPV
331	Ayash C	2022	North America	162	26	0.16	0.107599	0.226251	HPV
332	Kempe A	2020	North America	2176	137	0.063	0.053119	0.073997	Influenza
333	Nekrasov E	2020	North America	257	30	0.11	0.08016	0.162433	Influenza
334	Hofstetter AM	2018	North America	199	46	0.24	0.174482	0.296023	Influenza
335	Goss MD	2019	North America	244	76	0.31	0.253931	0.37367	Influenza
336	Cameron MA	2016	North America	786	385	0.49	0.454333	0.525388	Influenza
337	Nguyen AT	2022	North America	59424	40580	0.68	0.67913	0.68663	Influenza
338	He K	2022	North America	175	23	0.13	0.085173	0.190655	Mix
339	Footman A	2022	North America	21	2	0.095	0.011749	0.303774	Mix
340	Bardenheier B	2004	North America	2315	232	0.1	0.088279	0.113175	Mix
341	Freed GL	2009	North America	1552	171	0.11	0.095029	0.126824	Mix
342	Gilkey MB	2013	North America	1847	222	0.12	0.105706	0.135902	Mix
343	Gilkey GB	2016	North America	9354	1431	0.153	0.145743	0.160439	Mix
344	Gilkey MB	2016	North America	9018	2191	0.243	0.234136	0.251948	Mix
345	Letterie MC	2022	North America	1066	298	0.28	0.25278	0.307543	Mix
346	Wolff ER	2014	North America	99	28	0.28	0.196862	0.382216	Mix
347	Navin MC	2019	North America	4098	1598	0.39	0.374974	0.405076	Mix
348	Howell JL	2022	North America	863	74	0.09	0.067928	0.106453	Mix
349	Gowda C	2013	North America	79	16	0.2	0.120427	0.307957	MMR
350	Kettunen C.	2017	North America	84	2	0.024	0.002897	0.083375	Not stated

351	Cheng ER	2019	North America	1047	31	0.03	0.020204	0.041765	Not stated
352	Boyle J	2020	North America	1029	36	0.03	0.024621	0.048108	Not stated
353	Dudley MZ	2020	North America	2196	154	0.07	0.059799	0.08162	Not stated
354	Kwan BM	2022	North America	824	118	0.143	0.119991	0.169002	Not stated
355	Gust DA	2008	North America	3924	1099	0.28	0.266062	0.294409	Not stated
356	Blaisdell LL	2015	North America	42	14	0.33	0.195668	0.495488	Not stated
357	Glanz JM	2013	North America	443	164	0.37	0.325869	0.417951	Not stated
358	Mergler MJ	2013	North America	1367	533	0.39	0.363945	0.41634	Not stated
359	Chung Y	2017	North America	2603	1510	0.58	0.560867	0.599153	Not stated
360	Brown B	2017	North America	200	36	0.18	0.129369	0.240381	Others
361	Sahni LC	2020	North America	338	64	0.19	0.148985	0.235255	Others
362	Goin-Kochel RP	2020	North America	225	63	0.28	0.222362	0.343537	Others
363	Bardenheier B	2003	North America	648	246	0.38	0.342115	0.418246	Others
364	Beatty and Villwock	2021	North America	179	87	0.49	0.410	0.561	Influenza
365	Sokol, RL and Grummom, AH	2020	North America	1893	757	0.40	0.377	0.422	Covid-19
366	Zhu X	2023	North America	342	133	0.410	0.356	0.466	HPV
367	Yousaf A. R.	2023	North America	94	35	0.37	0.274	0.478	Covid-19
368	Wigle J.	2023	North America	20	3	0.15	0.032	0.378	Covid-19
369	Arrigoni, L and Strohm-Fraber, J	2023	North America	28	3	0.107	0.022	0.282	Covid-19
370	Shen A.K.	2023	North America	971	352	0.36	0.332	0.393	Covid-19
371	Schellenberg, N.	2021	North America	6125	941	0.15	0.144	0.162	Mix
372	Batra, K.	2023	North America	263	110	0.41	0.357	0.480	Covid-19
373	Alfieri, N.L. et al.	2021	North America	1425	470	0.33	0.305	0.354	Covid-19
374	Lachance-Grzela, M. et al.,	2022	North America	406	34	0.08	0.058	0.115	Covid-19
375	Davidson, CA et al	2023	North America	129	17	0.13	0.078	0.202	Covid-19
376	Gooding, GD et al	2023	North America	146	51	0.35	0.272	0.432	Covid-19
377	Mondal P & Sinharoy A	2023	North America	2622	894	0.34	0.322	0.359	Covid-19
378	Wojcicki, JM et al	2022	North America	391	104	0.26	0.222	0.312	Covid-19
379	Middleman A.B. et al.,	2021	North America	500	120	0.24	0.203	0.279	Covid-19
380	Margolis M. A. et al.,	2022	North America	1263	447	0.35	0.327	0.381	HPV
381	Kohler, RE et al	2023	North America	22	10	0.45	0.243	0.677	Covid-19
382	Kheil, M.H. et al.,	2022	North America	1746	105	0.06	0.049	0.072	Covid-19
383	Choi, K. et al.,	2021	North America	322	209	0.65	0.594	0.701	Covid-19
384	Mensah-Bonsu, NE et al	2021	North America	89	21	0.24	0.152	0.337	MMR
385	Wharton-Michael P & Wharton-Clark A	2019	North America	20	20	1.0	0.831	1	MMR
386	Parker A.A. et al.,	2006	North America	66	35	0.53	0.403	0.654	MMR
387	Dempsey AF, et al.	2011	North America	748	127	0.17	0.143	0.198	MMR
388	Downs, JS et al	2008	North America	30	11	0.36	0.199	0.561	MMR
389	Cataldi, JR et al	2016	North America	306	6	0.02	0.007	0.042	MMR
390	Lieu, TA et al	2015	North America	50233	1752	0.034	0.033	0.036	MMR
391	Mills, K et al	2020	North America	89	66	0.741	0.637	0.828	MMR
392	Gennaro, E et al	2021	North America	135	3	0.02	0.004	0.063	Mix
393	Langkamp, DL et al	2020	North America	63	11	0.18	0.090	0.290	Mix
394	Opel D.J. et al.,	2013	North America	437	33	0.075	0.525	0.104	Mix
395	Opel D.J. et al.,	2011	North America	237	37	0.16	0.112	0.208	Mix
396	Christianson, B et al	2020	North America	300	176	0.58	0.528	0.642	MMR
397	Frew, PM et al	2016	North America	5121	207	0.04	0.035	0.046	Mix
398	Fuchs, EL	2016	North America	4022	1404	0.34	0.334	0.364	Influenza
399	Kennedy A.M & Gust D.A	2008	North America	12	3	0.25	0.054	0.571	MMR
400	Holroyd T.A. et al.,	2021	North America	85	37	0.44	0.328	0.546	MMR
401	Glanz, JM et al	2020	North America	824	118	0.14	0.119	0.169	Mix
402	Sugerman, DE et al	2010	North America	839	63	0.07	0.058	0.095	MMR
403	Duchsherer A. et al.,	2020	North America	343	52	0.15	0.115	0.194	HPV
404	Davis, MM et al	2020	North America	1008	403	0.40	0.369	0.430	Covid-19
405	Gilbert NL	2016	North America	5720	800	0.14	0.1396	0.1484	HPV
406	Phan TL	2022	North America	513	77	0.15	0.120306	0.183988	Covid-19
407	Ruiz JB	2022	North America	637	52	0.082	0.061566	0.105674	Covid-19
408	Shen AK	2022	North America	41	7	0.17	0.071515	0.320561	Mix
409	Wang CS	2022	North America	207	52	0.25	0.193664	0.316025	Mix
410	Dubé È	2019	North America	2645	653	0.25	0.230547	0.263777	Not stated
411	Guay M	2019	North America	8737	2796	0.32	0.310241	0.329916	Not stated
412	Greyson D	2017	North America	23	9	0.39	0.1971	0.6146	Not stated
413	Dubé E	2016	North America	510	51	0.1	0.0789	0.1245	Others
414	Perez S	2015	North America	2874	172	0.06	0.051453	0.069151	HPV
415	Krawczyk A	2015	North America	806	100	0.12	0.0986	0.1439	HPV
416	Evans S	2021	Oceania	1094	101	0.093	0.075826	0.111047	Covid-19
417	Jeffs E	2021	Oceania	1173	356	0.304	0.277278	0.330702	Covid-19

418	Jones K	1992	Oceania	84	14	0.17	0.094224	0.2638	Influenza
419	Debela MS	2022	Oceania	178	29	0.163	0.111908	0.225528	Mix
420	Forbes TA	2015	Oceania	38	4	0.11	0.029435	0.248049	Mix
421	Corben P	2018	Oceania	231	5	0.022	0.007065	0.049787	Not stated
422	Armiento R	2020	Oceania	607	115	0.19	0.159032	0.222937	Not stated
423	Rozbroj T	2019	Oceania	904	347	0.38	0.352017	0.416442	Not stated
424	Attwell K	2016	Oceania	304	185	0.613	0.551202	0.663766	Not stated
425	Bolsewicz, KT et al	2023	Oceania	21	2	0.09	0.0117	0.3037	Covid-19
426	Kuan-Mahecha M.A. et al.,	2023	South America	503	9	0.02	0.008	0.033	Mix
427	Benites-Zapata VA	2022	South America	68980	4925	0.0713	0.069487	0.073344	Covid-19
428	Martinez EZ	2022	South America	1007	331	0.329	0.299726	0.358678	Covid-19
429	Gentile A	2021	South America	600	69	0.115	0.090587	0.143276	Mix
430	Burghouts J	2017	South America	67	25	0.37	0.257974	0.499895	Mix
431	Brunelli L	2020	South America	2557	38	0.015	0.010538	0.020342	Not stated
432	Brown AL	2018	South America	952	256	0.269	0.240968	0.298285	Not stated
433	Bono, SA	2022	South America	6571	464	0.07	0.064	0.077	Covid-19
434	Neto J.O.	2023	South America	1261	63	0.05	0.038	0.063	Mix
