# Supplementary material for: Prevalence of caregiver hesitancy for vaccinations in children and its associated factors: A systematic review and meta-analysis
Source: PLoS One. 2024 Oct 24;19(10):e0302379. doi: 10.1371/journal.pone.0302379 (PMC11500859; doi:10.1371/journal.pone.0302379)
Supplement: S6 Table — (PDF) [file pone.0302379.s010.pdf]

S6 Table: Overall prevalence of the vaccine hesitancy across the regions

Vaccine	Overall prevalence	CI value	I^2	p-value	Eagger test
**All vaccine (N= 434)**					
All regions	0.25	0.22, 0.27	99.91	0.001	0.0001
Asia	0.31	0.27, 0.34	99.95	0.001	0.0243
North America	0.25	0.23, 0.28	99.87	0.001	0.0014
South America	0.18	0.08, 0.27	99.85	0.001	0.0146
Africa	0.26	0.16, 0.36	99.78	0.001	0.0988
Europe	0.27	0.22, 0.32	99.94	0.001	0.1759
Oceania	0.22	0.11, 0.33	99.00	0.001	0.6386
Mix	0.25	0.13, 0.37	99.85	0.001	0.9028
**Covid – 19 (N = 189)**					
All regions	0.29	0.26, 0.32	99.91	0.001	0.1609
Africa	0.41	0.13, 0.69	99.03	0.001	NA
Asia	0.29	0.25, 0.34	99.91	0.001	0.3878
North America	0.26	0.22, 0.30	99.56	0.001	0.1003
South America	0.16	0.01, 0.32	99.95	0.001	0.0001
Europe	0.35	0.27, 0.43	99.71	0.001	0.4733
Oceania	0.17	0.02, 0.31	98.31	0.001	0.6410
Mix	0.33	0.18, 0.48	99.79	0.001	0.2575
**HPV (N = 53)**					
All regions	0.31	0.24, 0.37	99.93	0.001	0.7449
Africa	0.25	-011, 0.60	99.75	0.001	0.0124
Europe	0.16	0.07, 0.25	97.94	0.001	0.7986
North America	0.26	0.20, 0.33	99.92	0.001	0.2725
Asia	0.43	0.30, 0.56	99.82	0.001	0.5152
**Influenza (N = 28)**					
All	0.36	0.26, 0.46	99.92	0.001	0.4424
Asia	0.34	0.19, 0.48	99.92	0.001	0.0856
Europe	0.48	0.19, 0.78	99.54	0.001	0.6573
North America	0.34	0.20, 0.48	99.85	0.001	0.8714
**Mix (N = 76)**					
All	0.20	0.16, 0.24	99.93	0.001	0.0014
Africa	0.15	0.04, 0.26	98.36	0.001	0.0001
Asia	0.31	0.20, 0.41	99.97	0.001	0.0029
Europe	0.15	0.07, 0.22	99.81	0.001	0.6776
Mix	0.09	0.03, 0.16	95.95	0.001	0.2189
Oceania	0.15	0.10, 0.20	0.00	0.40	NA
South America	0.17	-0.01, 0.35	99.16	0.001	0.0001
North America	0.15	0.11, 0.19	99.37	0.001	0.5112
**MMR (N = 23)**					
All	0.29	0.18, 0.40	99.99	0.001	0.0164
Africa	0.15	0.12, 0.17	69.65	0.05	0.0212
Europe	0.26	0.01, 0.51	99.97	0.001	0.1613
North America	0.35	0.19, 0.50	99.76	0.001	0.2790
**Not stated (N = 39)**					
All	0.23	0.17, 0.28	99.84	0.001	0.0044

Asia	0.18	0.10, 0.27	99.47	0.001	0.0001
Europe	0.18	0.06, 0.30	99.94	0.001	0.0843
Oceania	0.30	0.05, 0.55	99.58	0.001	0.0002
North America	0.24	0.15, 0.34	99.76	0.001	0.3188
**Others (N = 26)**					
All	0.29	0.21, 0.36	99.60	0.001	0.2346
Africa	0.33	0.08, 0.58	99.87	0.001	0.0045
Asia	0.28	0.14, 0.41	99.65	0.001	0.7187
Europe	0.32	0.19, 0.45	99.18	0.001	0.5708
North America	0.23	0.13, 0.32	96.47	0.001	0.5312

*Overall prevalence was performed using random effect model; reported in percentage value; CI value in decimal value; I^2 heterogeneity value. NA: region have 2 or 3 publications.
