# Supplementary material for: Prevalence of caregiver hesitancy for vaccinations in children and its associated factors: A systematic review and meta-analysis
Source: PLoS One. 2024 Oct 24;19(10):e0302379. doi: 10.1371/journal.pone.0302379 (PMC11500859; doi:10.1371/journal.pone.0302379)
Supplement: S7 Table — (PDF) [file pone.0302379.s011.pdf]

S7 Table: Reasons for the parental hesitancy towards vaccine

	Religious	Knowledge	Information	Vaccine safety & efficacy	Others
**All vaccine**					
All continents (N = 765)	36.2	77.9	88.1	91.4	38.5
Africa (N = 65)	27.7	81.5	89.2	89.2	56.9
Asia (N = 241)	22.0	80.5	87.1	90.5	46.1
Europe (N = 148)	45.9	81.1	92.6	93.2	33.1
North America (N = 257)	43.4	72.1	85.3	91.9	31.9
South America (N = 15)	53.3	86.7	93.3	80.0	33.3
Oceania (N = 24)	70.8	95.8	100.0	100.0	8.3
Mix continent (N = 15)	13.3	46.7	80.0	80.0	60.0
**COVID-19 vaccine**					
All continents (N = 256)	5.9	69.9	83.6	92.6	52.0
Africa (N = 4)	100.0	100.0	100.0	100.0	75.0
Asia (N = 118)	6.8	78.0	84.7	94.1	58.5
Europe (N = 37)	2.7	56.8	78.4	91.9	48.6
North America (N = 79)	6.3	63.3	84.8	93.7	43.0
South America (N = 5)	20.0	80.0	80.0	80.0	60.0
Oceania (N = 4)	0.0	75.0	100.0	100.0	25.0
Mix continent (N = 9)	0.0	55.6	66.7	66.7	55.6
**HPV vaccine**					
All continents (N = 106)	42.5	86.8	90.6	85.8	34.9
Asia (N = 29)	17.2	86.2	86.2	69.0	44.8
Europe (N = 21)	47.2	90.5	100.0	90.5	19.0
North America (N = 47)	57.4	91.5	91.5	93.6	27.7
South America (N = 1)	100.0	100.0	100.0	100.0	100.0
Africa (N = 8)	25.0	50.0	75.0	87.5	75.0
**Influenza vaccine**					
All continents (N = 46)	52.2	89.1	95.7	95.7	26.1
Asia (N = 18)	33.3	88.9	94.4	94.4	38.9
Europe (N = 11)	63.6	100.0	100.0	90.9	18.2
North America (N = 14)	64.3	78.6	92.9	100.0	21.4
Oceania (N =3)	66.7	100.0	100.0	100.0	0.0
**MMR vaccine**					
All continents (N = 50)	48.0	70.0	78.0	90.0	34.0
Asia (N = 3)	66.7	100.0	100.0	100.0	0.0
North America (N = 26)	23.1	42.3	65.4	84.6	50.0
Europe (N = 15)	93.3	100.0	100.0	100.0	6.7
Africa (N = 6)	33.3	100.0	66.7	83.3	50.0
**Mix vaccine***					
All continents (N = 161)	36.0	72.0	88.2	88.8	41.9
Africa (N = 26)	7.7	76.9	96.2	88.5	65.4
Asia (N = 40)	27.5	70.0	82.5	87.5	35.0
Europe (N = 37)	48.6	83.8	97.3	94.6	40.5
North America (N = 45)	46.7	60.0	77.8	84.4	38.6
South America (N = 5)	60.0	100.0	100.0	80.0	0.0
Oceania (N = 4)	50.0	100.0	100.0	100.0	25.0
Mix continent (N = 4)	25.0	25.0	100.0	100.0	75.0

**Others vaccine****					
All continents (N = 43)	53.5	83.7	88.4	93.0	37.2
Asia (N = 12)	50.0	100.0	100.0	100.0	25.0
Europe (N = 9)	41.7	83.3	91.7	100.0	41.7
North America (N = 6)	83.3	83.3	83.3	83.3	16.7
Africa (N =13)	61.5	84.6	84.6	92.3	38.5
Mix (N = 1)	0.0	0.0	100.0	100.0	100.0
Oceania (N = 1)	100.0	100.0	100.0	100.0	0.0
South America (N = 1)	0.0	100.0	100.0	100.0	0.0
**Not stated vaccine*****					
All continents (N = 103)	85.4	94.2	98.1	96.1	11.7
Asia (N = 21)	76.2	95.2	100.0	95.2	14.3
Europe (N = 18)	77.8	83.3	94.4	94.4	27.8
North America (N = 41)	95.1	95.1	97.6	97.6	2.4
Africa (N = 8)	50.0	100.0	100.0	87.5	37.5
Oceania (N = 12)	100.0	100.0	100.0	100.0	0.0
South America (N = 2)	100.0	100.0	100.0	100.0	0.0
Mix (N = 1)	100.0	100.0	100.0	100.0	0.0

All the number is presented in percentage.
* Mix vaccine: Children received more than 1 type of vaccine during the survey for example Influenza & COVID-10 or child immunization program.
** Other vaccine for example Hepatitis A & B, Malaria, Polio, Diphtheria, pneumococcal and others
*** Not stated vaccine – respondents not stated type of vaccine they received during the survey.
