# Supplementary material for: Prevalence of caregiver hesitancy for vaccinations in children and its associated factors: A systematic review and meta-analysis
Source: PLoS One. 2024 Oct 24;19(10):e0302379. doi: 10.1371/journal.pone.0302379 (PMC11500859; doi:10.1371/journal.pone.0302379)
Supplement: S1 Appendix — (PDF) [file pone.0302379.s012.pdf]

S1 Appendix: List of the included study

1. Kamya C, Namugaya F, Opio C, Katamba P, Carnahan E, Katahoire A, Nankabirwa J, Okiring J, Waiswa P. Coverage and Drivers to Reaching the Last Child With Vaccination in Urban Settings: A Mixed-Methods Study in Kampala, Uganda. Global Health: Science and Practice. 2022 Aug 30;10(4).
2. Balogun FM, Omotade OO. Parental intention to vaccinate adolescents with HPV vaccine in selected communities in Ibadan, Southwest Nigeria: an application of Integrated Behavioral Model. Human Vaccines & Immunotherapeutics. 2022 Nov 30;18(5):2069959.
3. Immurana M, Boachie MK, Klu D, Dalaba MA, Manyeh AK, Alhassan RK. Determinants of willingness to accept child vaccination against malaria in Ghana. The International Journal of Health Planning and Management. 2022 May;37(3):1439-53.
4. Obute JA, Arulogun OS. Parents' awareness and perception of the polio eradication programme in Gombe Local Government Area, Gombe State, Nigeria. International Journal of Health Promotion and Education. 2007 Jan 1;45(3):81-6.
5. Darebo TD, Oshe BB, Diro CW. Full vaccination coverage and associated factors among children aged 12 to 23 months in remote rural area of Demba Gofa District, Southern Ethiopia. PeerJ. 2022 Mar 14;10:e13081.
6. Abor J, Kabunga A, Nabasirye CK. Predictors of Adherence to Routine Immunization Schedule Among Caretakers of Children Aged 10 to 18 Months in Lira City, Uganda. Global Pediatric Health. 2022 Dec;9:2333794X221140518.
7. Griffith BC, Cusick SE, Searle KM, Negoescu DM, Basta NE, Banura C. Does mothers’ and caregivers' access to information on their child’s vaccination card impact the timing of their child’s measles vaccination in Uganda?. BMC public health. 2022 Apr 26;22(1):834.
8. Kyei-Arthur F, Kyei-Gyamfi S, Agyekum MW, Afrifa-Anane GF, Amoh BA. Parents’ and guardians’ acceptability of COVID-19 vaccination for children in Ghana: An online survey. Plos one. 2022 Aug 29;17(8):e0272801.
9. Tefera YA, Wagner AL, Mekonen EB, Carlson BF, Boulton ML. Predictors and barriers to full vaccination among children in Ethiopia. Vaccines. 2018 Apr 10;6(2):22.
10. Mohammed A, Sabitu K, Nguku P, Abanida E, Sheidu S, Dalhat M, Dankoli R, Gidado S, Suleiman I. Characteristics of persons refusing oral polio vaccine during the immunization plus days–Sokoto, Nigeria 2011. The Pan African Medical Journal. 2014;18(Suppl 1).
11. Asmare G. Willingness to accept malaria vaccine among caregivers of under-5 children in Southwest Ethiopia: a community based cross-sectional study. Malaria Journal. 2022 Dec;21(1):1-8.
12. Abdullahi MF, Stewart Williams J, Sahlèn KG, Bile K, Kinsman J. Factors contributing to the uptake of childhood vaccination in Galkayo District, Puntland, Somalia. Global health action. 2020 Dec 31;13(1):1803543.
13. Abubakar A, Dalhat M, Mohammed A, Ilesanmi OS, Anebonam U, Barau N, Salami S, Ajayi O, Shehu A, Oladimeji A, Gidado S. Outbreak of suspected pertussis in Kaltungo, Gombe State, Northern Nigeria, 2015: the role of sub-optimum routine immunization coverage. The Pan African Medical Journal. 2019;32(Suppl 1).

14. Al-Wutayd O, Al-Batanony M, Badr N, Abdelwanees S. Parents' Intentions and Associated Factors to Vaccinating Their Children Aged 12–17 Years with COVID-19 Vaccines: A Cross Sectional Study. Vaccines. 2022 Jun 8;10(6):912.
15. Antai D, Ghilagaber G, Wedrén S, Macassa G, Moradi T. Inequities in under-five mortality in Nigeria: differentials by religious affiliation of the mother. Journal of religion and health. 2009 Sep;48(3):290-304.
16. Asmare G, Madalicho M, Sorsa A. Disparities in full immunization coverage among urban and rural children aged 12-23 months in southwest Ethiopia: A comparative cross-sectional study. Human Vaccines & Immunotherapeutics. 2022 Nov 30;18(6):2101316.
17. Dejene H, Girma D, Adugna L, Legese E. Vaccination Timeliness and Associated Factors Among Children Age 12-23 Months in Debrelebanos District of North Shewa Zone, Oromia Regional State, Ethiopia. Frontiers in Pediatrics. 2022 Jul 27:1229.
18. Dugas M, Dubé E, Kouyaté B, Sanou A, Bibeau G. Portrait of a lengthy vaccination trajectory in Burkina Faso: from cultural acceptance of vaccines to actual immunization. BMC International Health and Human Rights. 2009 Oct;9(1):1-1.
19. Elit L, Ngalla C, Afugchwi GM, Tum E, Domgue JF, Nouvet E. Assessing knowledge, attitudes and belief toward HPV vaccination of parents with children aged 9–14 years in rural communities of Northwest Cameroon: a qualitative study. BMJ open. 2022 Nov 1;12(11):e068212.
20. Hailu C, Fisseha G, Gebreyesus A. Determinants of measles vaccination dropout among 12− 23 months aged children in pastoralist community of Afar, Ethiopia. BMC Infectious Diseases. 2022 Dec;2Ha2(1):1-9.
21. Horn S, Chapman GB, Chouhan K. Doctor recommendations and parents' HPV vaccination intentions in Kenya: A randomized survey. Preventive Medicine Reports. 2022 Feb 1;25:101659.
22. Kemeugni Ngandjon J, Ostermann T, Kenmoue V, Laengler A. Insights into Predictors of Vaccine Hesitancy and Promoting Factors in Childhood Immunization Programs—A Cross-Sectional Survey in Cameroon. International Journal of Environmental Research and Public Health. 2022 Feb 26;19(5):2721.
23. Killion JP, Silverman DT, Evans D, Coetzee L, Tarullo AR, Hamer DH, Rockers PC. Vaccination coverage at seven months of age in Limpopo Province, South Africa: A cross-sectional survey. Global Health Promotion. 2022 Aug 4:17579759221107037.
24. Machekanyanga Z, Ndiaye S, Gerede R, Chindedza K, Chigodo C, Shibeshi ME, Goodson J, Daniel F, Zimmerman L, Kaiser R. Qualitative assessment of vaccination hesitancy among members of the apostolic church of Zimbabwe: a case study. Journal of religion and health. 2017 Oct;56:1683-91.
25. Tayu B, Melaku N, Tefera K, Gebretsadik A. Incomplete immunization and associated factors among children 12–23 months in Aletawondo district, Sidama zone, South Ethiopia: Across-sectional study. Journal of Child Health Care. 2022 Oct 25:13674935221133463.
26. Umeh GC, Nomhwange TI, Shamang AF, Zakari F, Musa AI, Dogo PM, Gugong V, Iliyasu N. Attitude and subjective wellbeing of non-compliant mothers to childhood oral polio vaccine supplemental immunization in Northern Nigeria. BMC public health. 2018 Dec;18(1):1-7.

27. Vasudevan L, Baumgartner JN, Moses S, Ngadaya E, Mfinanga SG, Ostermann J. Parental concerns and uptake of childhood vaccines in rural Tanzania–a mixed methods study. BMC Public Health. 2020 Dec;20(1):1-1.
28. Yeboah D, Owusu-Marfo J, Agyeman YN. Predictors of malaria vaccine uptake among children 6–24 months in the Kassena Nankana Municipality in the Upper East Region of Ghana. Malaria Journal. 2022 Dec;21(1):1-0.
29. Carcelen AC, Prosperi C, Mutembo S, Chongwe G, Mwansa FD, Ndubani P, Simulundu E, Chilumba I, Musukwa G, Thuma P, Kapungu K. COVID-19 vaccine hesitancy in Zambia: a glimpse at the possible challenges ahead for COVID-19 vaccination rollout in sub-Saharan Africa. Human vaccines & immunotherapeutics. 2022 Jan 31;18(1):1-6.
30. Kolek CO, Opanga SA, Okalebo F, Birichi A, Kurdi A, Godman B, Meyer JC. Impact of Parental Knowledge and Beliefs on HPV Vaccine Hesitancy in Kenya—Findings and Implications. Vaccines. 2022 Jul 26;10(8):1185.
31. Mebrate M, Workicho A, Alemu S, Gelan E. Vaccination Status and Its Determinants Among Children Aged 12 to 23 Months in Mettu and Sinana Districts, Oromia Region, Ethiopia: A Comparative Cross Sectional Study. Pediatric Health, Medicine and Therapeutics. 2022 Dec 31:335-48.
32. Mihretie GN, Liyeh TM, Ayele AD, Belay HG, Yimer TS, Miskr AD. Knowledge and willingness of parents towards child girl HPV vaccination in Debre Tabor Town, Ethiopia: a community-based cross-sectional study. Reproductive Health. 2022 Dec;19(1):1-2.
33. Muluye M, Oljira L, Eyeberu A, Getachew T, Debella A, Deressa A, Dheresa M. Partial vaccination and associated factors among children aged 12–23 months in eastern Ethiopia. BMC pediatrics. 2022 Dec;22(1):1-0.
34. Sabahelzain MM, Moukhyer M, Bosma H, van den Borne B. Determinants of Measles Vaccine Hesitancy among Sudanese Parents in Khartoum State, Sudan: A Cross-Sectional Study. Vaccines. 2021 Dec 22;10(1):6.
35. Idris IO, Tapkigen J, Kabutaulaka G, Ayeni GO, Ayomoh FI, Obwoya JG. Are children on track with their routine immunization schedule in a fragile and protracted conflict state of South Sudan? A community-based cross-sectional study. BMC pediatrics. 2022 Mar 21;22(1):147.
36. Popelsky BK, Asare M, Agyei-Baffour P, Koranteng A, Commeh ME. Assessing Attitudes and Beliefs Toward HPV Vaccination among Ghanaian Parents with Unvaccinated Adolescents: Application of Multi-Theory Model of Behavior Change. Asian Pacific Journal of Cancer Prevention. 2022 Jun 1;23(6):1901-11.
37. Jalloh MF, Patel P, Sutton R, Kulkarni S, Toure M, Wiley K, Sessay T, Lahuerta M. Qualitative assessment of caregiver experiences when navigating childhood immunisation in urban communities in Sierra Leone. BMJ open. 2022 May 1;12(5):e058203.
38. Abakar MF, Seli D, Lechthaler F, Schelling E, Tran N, Zinsstag J, Muñoz DC. Vaccine hesitancy among mobile pastoralists in Chad: a qualitative study. International journal for equity in health. 2018 Dec;17:1-0.
39. Ames H, Njang DM, Glenton C, Fretheim A, Kaufman J, Hill S, Oku A, Cliff J, Cartier Y, Bosch-Capblanch X, Rada G. Stakeholder perceptions of communication about

vaccination in two regions of Cameroon: A qualitative case study. PLoS One. 2017 Aug 31;12(8):e0183721.
40. Babirye JN, Rutebemberwa E, Kiguli J, Wamani H, Nuwaha F, Engebretsen IM. More support for mothers: a qualitative study on factors affecting immunisation behaviour in Kampala, Uganda. BMC public health. 2011 Dec;11:1-1.
41. Cassell JA, Leach M, Fairhead JR, Small M, Mercer CH. The social shaping of childhood vaccination practice in rural and urban Gambia. Health policy and planning. 2006 Sep 1;21(5):373-91.
42. Closser S, Rosenthal A, Maes K, Justice J, Cox K, Omidian PA, Mohammed IZ, Dukku AM, Koon AD, Nyirazinyoye L. The global context of vaccine refusal: insights from a systematic comparative ethnography of the global polio eradication initiative. Medical anthropology quarterly. 2016 Sep;30(3):321-41.
43. Cockcroft A, Usman MU, Nyamucherera OF, Emori H, Duke B, Umar NA, Andersson N. Why children are not vaccinated against measles: a cross-sectional study in two Nigerian States. Archives of Public health. 2014 Dec;72:1-0.
44. Cutts FT, Glik DC, Gordon A, Parker K, Diallo S, Haba F, Stone R. Application of multiple methods to study the immunization programme in an urban area of Guinea. Bulletin of the World Health Organization. 1990;68(6):769.
45. Eng E, Naimoli J, Naimoli G, Parker KA, Lowenthal N. The acceptability of childhood immunization to Togolese mothers: A sociobehavioral perspective. Health education quarterly. 1991 Apr;18(1):97-110.
46. Fleming JA, Munthali A, Ngwira B, Kadzandira J, Jamili-Phiri M, Ortiz JR, Lambach P, Hombach J, Neuzil KM, Stepanchak M, Bhat N. Maternal immunization in Malawi: A mixed methods study of community perceptions, programmatic considerations, and recommendations for future planning. Vaccine. 2019 Jul 26;37(32):4568-75.
47. Gil Cuesta J, Whitehouse K, Kaba S, Nanan-N’Zeth K, Haba B, Bachy C, Panunzi I, Venables E. ‘When you welcome well, you vaccinate well’: a qualitative study on improving vaccination coverage in urban settings in Conakry, Republic of Guinea. International Health. 2021 Nov;13(6):586-93.
48. Giles-Vernick T, Traoré A, Bainilago L. Incertitude, hepatitis B, and infant vaccination in west and Central Africa. Medical Anthropology Quarterly. 2016 Jun;30(2):203-21.
49. Handy LK, Maroudi S, Powell M, Nfila B, Moser C, Japa I, Monyatsi N, Tzortzi E, Kouzeli I, Luberti A, Theodoridou M. The impact of access to immunization information on vaccine acceptance in three countries. PloS one. 2017 Aug 3;12(8):e0180759.
50. Helman CG, Yogeswaran P. Perceptions of childhood immunisations in rural Transkei-a qualitative study. South African Medical Journal. 2004 Oct 1;94(10):835-8.
51. Kagoné M, Yé M, Nébié E, Sié A, Müller O, Beiersmann C. Community perception regarding childhood vaccinations and its implications for effectiveness: a qualitative study in rural Burkina Faso. BMC public health. 2018 Dec;18:1-0.
52. Leach MA, Fairhead JR, Millimouno D, Diallo AA. New therapeutic landscapes in Africa: parental categories and practices in seeking infant health in the Republic of Guinea. Social science & medicine. 2008 May 1;66(10):2157-67.
53. Renne E. Perspectives on polio and immunization in Northern Nigeria. Social science & medicine. 2006 Oct 1;63(7):1857-69.

54. Schwarz NG, Gysels M, Pell C, Gabor J, Schlie M, Issifou S, Lell B, Kremsner PG, Grobusch MP, Pool R. Reasons for non-adherence to vaccination at mother and child care clinics (MCCs) in Lambarene, Gabon. Vaccine. 2009 Aug 27;27(39):5371-5.
55. Tabana H, Dudley LD, Knight S, Cameron N, Mahomed H, Goliath C, Eggers R, Wiysonge CS. The acceptability of three vaccine injections given to infants during a single clinic visit in South Africa. BMC Public Health. 2016 Dec;16(1):1-0.
56. Tadesse H, Deribew A, Woldie M. Explorative assessment of factors affecting child immunization in Wonago district, Gedeo zone, South Ethiopia. Archives of Medical Science. 2009 Apr 1;5(2):233-40.
57. Tadesse T, Getachew K, Assefa T, Ababu Y, Simireta T, Birhanu Z, Hailemichael Y. Factors and misperceptions of routine childhood immunization service uptake in Ethiopia: findings from a nationwide qualitative study. Pan African Medical Journal. 2017;28(1).
58. Zewdie A, Letebo M, Mekonnen T. Reasons for defaulting from childhood immunization program: a qualitative study from Hadiya zone, Southern Ethiopia. BMC public health. 2016 Dec;16(1):1-9.
59. Powelson J, Magadzire BP, Draiva A, Denno D, Ibraimo A, Benate BB, Jahar LC, Marrune Z, Chilundo B, Chinai JE, Emerson M. Determinants of immunisation dropout among children under the age of 2 in Zambézia province, Mozambique: a community-based participatory research study using Photovoice. BMJ open. 2022 Mar 1;12(3):e057245.
60. Stamidis KV, Bologna L, Bisrat F, Tadesse T, Tessema F, Kang E. Trust, communication, and community networks: how the CORE Group Polio Project community volunteers led the fight against polio in Ethiopia’s most at-risk areas. The American Journal of Tropical Medicine and Hygiene. 2019 Oct;101(4 Suppl):59.
61. Olaniyan A, Isiguzo C, Agbomeji S, Akinlade-Omeni O, Ifie B, Hawk M. Barriers, facilitators, and recommendations for childhood immunisation in Nigeria: perspectives from caregivers, community leaders, and healthcare workers. The Pan African Medical Journal. 2022;43.
62. Milondzo T, Meyer JC, Dochez C, Burnett RJ. Human papillomavirus vaccine hesitancy highly evident among caregivers of girls attending South African private schools. Vaccines. 2022 Mar 24;10(4):503.
63. Lubeya MK, Chibwesha CJ, Mwanahamuntu M, Mukosha M, Maposa I, Kawonga M. Correlates of Parental Consent to Human Papillomavirus Vaccine Uptake by Their Adolescent Daughters in ZAMBIA: Application of the Health Belief Model. Vaccines. 2023 Apr 28;11(5):912.
64. Sulaiman SK, Tsiga-Ahmed FI, Musa MS, Sulaiman AK, Dayyab FM, Khan MA, Ahmad SI, Akpan UA, Usman UI, Bako AT. Prevalence, determinants, and reasons for malaria vaccine hesitancy among caregivers of under-five children in Nigeria: Results from a nationwide cross-sectional survey. Vaccine. 2023 Feb 17;41(8):1503-12.
65. McKnight J, Holt DB. Designing the Expanded Programme on Immunisation (EPI) as a service: Prioritising patients over administrative logic. Global Public Health. 2014 Nov 26;9(10):1152-66.
66. Albright K, Barnard J, O'Leary ST, Lockhart S, Jimenez-Zambrano A, Stokley S, Dempsey A, Kempe A. Noninitiation and noncompletion of HPV vaccine among

English-and Spanish-speaking parents of adolescent girls: a qualitative study. Academic pediatrics. 2017 Sep 1;17(7):778-84.

67. Alfieri NL, Kusma JD, Heard-Garris N, Davis MM, Golbeck E, Barrera L, Macy ML. Parental COVID-19 vaccine hesitancy for children: vulnerability in an urban hotspot. BMC Public Health. 2021 Dec;21:1-9.
68. Ali S, Kammerer E, Thompson G, Mater A, Rajagopal M, Bone JN, Birnie KA, Oberlander T, Chambers CT, Goldman RD, International COVID-19 Parental Attitude Study (COVIPAS) Group. A multicentre Canadian survey of caregiver perspectives on COVID vaccine-related pain and stress for their family. British Journal of Pain. 2022 Oct;16(5):490-7.
69. Myhre A, Xiong T, Vogel RI, Teoh D. Associations between risk-perception, self-efficacy and vaccine response-efficacy and parent/guardian decision-making regarding adolescent HPV vaccination. Papillomavirus Research. 2020 Dec 1;10:100204.
70. Amin AB, Bednarczyk RA, Ray CE, Melchiori KJ, Graham J, Huntsinger JR, Omer SB. Association of moral values with vaccine hesitancy. Nature Human Behaviour. 2017 Dec;1(12):873-80.
71. Anuforo B, McGee-Avila JK, Toler L, Xu B, Kohler RE, Manne S, Tsui J. Disparities in HPV vaccine knowledge and adolescent HPV vaccine uptake by parental nativity among diverse multiethnic parents in New Jersey. BMC public health. 2022 Jan 29;22(1):195.
72. Arora G, Lehman D, Charlu S, Ross N, Ardy A, Gordon B, Pannaraj PS. Vaccine health beliefs and educational influences among pediatric residents. Vaccine. 2019 Feb 4;37(6):857-62.
73. Auslander BA, Meers JM, Short MB, Zimet GD, Rosenthal SL. A qualitative analysis of the vaccine intention–behaviour relationship: parents' descriptions of their intentions, decision-making behaviour and planning processes towards HPV vaccination. Psychology & health. 2019 Mar 4;34(3):271-88.
74. Ayash C, Raad N, Finik J, Attia N, Nourredine S, Aragones A, Gany F. Arab American Mothers' HPV Vaccination Knowledge and Beliefs. Journal of Community Health. 2022 Aug;47(4):716-25.
75. Ayres S, Gee A, Kim S, Hashibe M, Praag A, Kaiser D, Chang CP, Brandt HM, Kepka D. Human Papillomavirus Vaccination Knowledge, Barriers, and Recommendations Among Healthcare Provider Groups in the Western United States. Journal of Cancer Education. 2022 Dec 1:1-8.
76. Bardenheier B, González IM, Washington ML, Bell BP, Averhoff F, Massoudi MS, Hyams I, Simard EP, Yusuf H. Parental knowledge, attitudes, and practices associated with not receiving hepatitis A vaccine in a demonstration project in Butte County, California. Pediatrics. 2003 Oct;112(4):e269-.
77. Bardenheier B, Yusuf H, Schwartz B, Gust D, Barker L, Rodewald L. Are parental vaccine safety concerns associated with receipt of measles-mumps-rubella, diphtheria and tetanus toxoids with acellular pertussis, or hepatitis B vaccines by children?. Archives of pediatrics & adolescent medicine. 2004 Jun 1;158(6):569-75.
78. Baumer-Mouradian SH, Hart RJ, Visotcky A, Fraser R, Prasad S, Levas M, Nimmer M, Brousseau DC. Understanding Influenza and SARS-CoV-2 Vaccine Hesitancy in Racial and Ethnic Minority Caregivers. Vaccines. 2022 Nov 20;10(11):1968.

79. Baumer-Mouradian SH, Hart RJ, Bone JN, Seiler M, Olson P, Keitel K, Manzano S, Gualco G, Krupik D, Schroter S, Weigert RM. Should COVID-19 vaccines be mandated in schools?-an international caregiver perspective. Vaccine. 2022 Aug 26;40(36):5384-90.
80. Bazzano A, Zeldin A, Schuster E, Barrett C, Lehrer D. Vaccine-related beliefs and practices of parents of children with autism spectrum disorders. American Journal on Intellectual and Developmental Disabilities. 2012 May;117(3):233-42.
81. Beavis A, Krakow M, Levinson K, Rositch AF. Reasons for lack of HPV vaccine initiation in NIS-teen over time: shifting the focus from gender and sexuality to necessity and safety. Journal of Adolescent Health. 2018 Nov 1;63(5):652-6.
82. Beavis AL, Meek K, Moran MB, Fleszar L, Adler S, Rositch AF. Exploring HPV vaccine hesitant parents' perspectives on decision-making and motivators for vaccination. Vaccine: X. 2022 Dec 1;12:100231.
83. Blaisdell LL, Gutheil C, Hootsmans NA, Han PK. Unknown risks: parental hesitation about vaccination. Medical Decision Making. 2016 May;36(4):479-89.
84. Boyle J, Berman L, Nowak GJ, Iachan R, Middleton D, Deng Y. An assessment of parents' childhood immunization beliefs, intentions, and behaviors using a smartphone panel. Vaccine. 2020 Feb 28;38(10):2416-23.
85. Brabin L, Roberts SA, Stretch R, Baxter D, Chambers G, Kitchener H, McCann R. Uptake of first two doses of human papillomavirus vaccine by adolescent schoolgirls in Manchester: prospective cohort study. Bmj. 2008 May 8;336(7652):1056-8.
86. Brown B, Gabra MI, Pellman H. Reasons for acceptance or refusal of human papillomavirus vaccine in a California pediatric practice. Papillomavirus Research. 2017 Jun 1;3:42-5.
87. Buttenheim AM, Joyce CM, Ibarra J, Agas J, Feemster K, Handy LK, Amin AB, Omer SB. Vaccine exemption requirements and parental vaccine attitudes: an online experiment. Vaccine. 2020 Mar 4;38(11):2620-5.
88. Cacciatore MA, Nowak G, Evans NJ. Exploring the impact of the US measles outbreak on parental awareness of and support for vaccination. Health Affairs. 2016 Feb 1;35(2):334-40.
89. Cameron MA, Bigos D, Festa C, Topol H, Rhee KE. Missed opportunity: why parents refuse influenza vaccination for their hospitalized children. Hospital pediatrics. 2016 Sep;6(9):507-12.
90. Carrion ML. "You need to do your research": Vaccines, contestable science, and maternal epistemology. Public Understanding of Science. 2018 Apr;27(3):310-24.
91. Carrion ML. An ounce of prevention: identifying cues to (in) action for maternal vaccine refusal. Qualitative health research. 2018 Dec;28(14):2183-94.
92. Cataldi JR, Sevick C, Pyrzanowski J, Wagner N, Brewer SE, Narwaney KJ, Shoup JA, Resnicow K, Glanz J, Dempsey A, Kwan BM. Addressing personal parental values in decisions about childhood vaccination: measure development. Vaccine. 2019 Sep 10;37(38):5688-97.
93. Wu AC, Wisler-Sher DJ, Griswold K, Colson E, Shapiro ED, Holmboe ES, Benin AL. Postpartum mothers' attitudes, knowledge, and trust regarding vaccination. Maternal and child health journal. 2008 Nov;12:766-73.

94. Cheng ER, Ranchoff B, Declercq ER. Distinguishing Subgroups in Opposition to Infant Immunization: Results From the Listening to Mothers III Survey. The Journal of Perinatal Education. 2019 Jul 8;28(3):126-30.
95. Chido-Amajuoyi OG, Talluri R, Jackson I, Shete SS, Domgue JF, Shete S. The influence of parent–child gender on intentions to refuse HPV vaccination due to safety concerns/side effects, National Immunization Survey–Teen, 2010–2019. Human Vaccines & Immunotherapeutics. 2022 Nov 30;18(5):2086762.
96. Chung Y, Schamel J, Fisher A, Frew PM. Influences on immunization decision-making among US parents of young children. Maternal and child health journal. 2017 Dec;21(12):2178-87.
97. Cioffredi LA, Kohlasch KL, Thomas E, Potter AS. Persistently low readiness to vaccinate young children against COVID among vaccine adherent mothers in Vermont, USA. Preventive Medicine Reports. 2022 Aug 1;28:101841.
98. Darden PM, Thompson DM, Roberts JR, Hale JJ, Pope C, Naifeh M, Jacobson RM. Reasons for not vaccinating adolescents: National Immunization Survey of Teens, 2008–2010. Pediatrics. 2013 Apr;131(4):645-51.
99. de St Maurice A, Block Jr R, Sanchez G, Szilagyi PG, Collaborative AA, 2021 COVID Group. Parental COVID-19 Vaccine Hesitancy in Diverse Communities: A National Survey. Academic pediatrics. 2022 Nov 1;22(8):1399-406.
100. Deas J, Bean SJ, Sokolovska I, Fautin C. Childhood vaccine attitudes and information sources among Oregon parents and guardians. Health Promotion Practice. 2019 Jul;20(4):529-38.
101. Delamater PL, Leslie TF, Yang YT. Examining the spatiotemporal evolution of vaccine refusal: nonmedical exemptions from vaccination in California, 2000–2013. BMC Public Health. 2018 Dec;18(1):1-3.
102. Delgado JR, Szilagyi PG, Peralta JB, Shah MD, Thomas K, Vizueta N, Cui Y, Vangala S, Shetgiri R, Kapteyn A. Influence of Perceived adolescent vaccination Desire on parent Decision for adolescent COVID-19 vaccination. Journal of Adolescent Health. 2022 Apr 1;70(4):567-70.
103. Dempsey AF, Abraham LM, Dalton V, Ruffin M. Understanding the reasons why mothers do or do not have their adolescent daughters vaccinated against human papillomavirus. Annals of epidemiology. 2009 Aug 1;19(8):531-8.
104. Dorell C, Yankey D, Jeyarajah J, Stokley S, Fisher A, Markowitz L, Smith PJ. Delay and refusal of human papillomavirus vaccine for girls, National Immunization Survey–Teen, 2010. Clinical pediatrics. 2014 Mar;53(3):261-9.
105. Drouin O, Fontaine P, Arnaud Y, Montmarquette C, Prud'homme A, Da Silva RB. Parental decision and intent towards COVID-19 vaccination in children with asthma: an econometric analysis. BMC Public Health. 2022 Dec;22(1):1-2.
106. Dudley MZ, Limaye RJ, Omer SB, O'Leary ST, Ellingson MK, Spina CI, Chamberlain AT, Brewer SE, Bednarczyk RA, Malik F, Frew PM. Latent class analysis of maternal vaccine attitudes and beliefs. Health Education & Behavior. 2020 Oct;47(5):765-81.
107. Dundar Y, Eldem I, Schwartz C, Pomeroy L, Cordero J, Arslan O, Levent F. Screening awareness of HPV-related oropharyngeal cancers and attitudes and concerns towards HPV vaccination among parents: HPV and oropharyngeal cancer. Journal of Cancer Education. 2021 Jan 7:1-9.

108. Egbert N, Zhu Y, Choi M, Beam MA, Smith TC. Family Communication Patterns and Parents' Intentions to Vaccinate Their Child Against COVID-19. Health Communication. 2022 Aug 26:1-8.
109. Eller NM, Henrikson NB, Opel DJ. Vaccine information sources and parental trust in their child's health care provider. Health Education & Behavior. 2019 Jun;46(3):445-53.
110. Ellithorpe ME, Adams R, Aladé F. Parents' behaviors and experiences associated with four vaccination behavior groups for childhood vaccine hesitancy. Maternal and Child Health Journal. 2022 Feb 1:1-9.
111. Estep KA. Neighborhood political composition and personal belief exemptions from immunization requirements in California Kindergartens, 2000–2015. Vaccine. 2018 Jul 5;36(29):4298-303.
112. Fair E, Murphy TV, Golaz A, Wharton M. Philosophic objection to vaccination as a risk for tetanus among children younger than 15 years. Pediatrics. 2002 Jan;109(1):e2-.
113. Finkelstein SR, Boland WA, Vallen B, Connell PM, Sherman GD, Feemster KA. Psychological reactance impacts ratings of pediatrician vaccine-related communication quality, perceived vaccine safety, and vaccination priority among US parents. Human Vaccines & Immunotherapeutics. 2020 May 3;16(5):1024-9.
114. Fogel BN, Hicks SD. "Flu-floppers": factors influencing families' fickle flu vaccination patterns. Clinical Pediatrics. 2020 May;59(4-5):352-9.
115. Fredrickson DD, Davis TC, Arnould CL, Kennen EM, Humiston SG, Cross JT, Bocchini JA. Childhood immunization refusal: provider and parent perceptions. Family Medicine-Kansas City-. 2004 Jun 1;36:431-9.
116. Freed GL, Clark SJ, Butchart AT, Singer DC, Davis MM. Parental vaccine safety concerns in 2009. Pediatrics. 2010 Apr;125(4):654-9.
117. Freeman RE, Thaker J, Daley MF, Glanz JM, Newcomer SR. Vaccine timeliness and prevalence of undervaccination patterns in children ages 0–19 months, US, National Immunization Survey-Child 2017. Vaccine. 2022 Jan 31;40(5):765-73.
118. Frew PM, Hixson B, del Rio C, Esteves-Jaramillo A, Omer SB. Pediatrics. 2011 May;127(Supplement_1):S113-9.
119. Gaudino JA, Robison S. Risk factors associated with parents claiming personal-belief exemptions to school immunization requirements: community and other influences on more skeptical parents in Oregon, 2006. Vaccine. 2012 Feb 1;30(6):1132-42.
120. Gilkey MB, Calo WA, Marciniak MW, Brewer NT. Parents who refuse or delay HPV vaccine: differences in vaccination behavior, beliefs, and clinical communication preferences. Human Vaccines & Immunotherapeutics. 2017 Mar 4;13(3):680-6.
121. Gilkey MB, McRee AL, Brewer NT. Forgone vaccination during childhood and adolescence: findings of a statewide survey of parents. Preventive medicine. 2013 Mar 1;56(3-4):202-6.
122. Gilkey MB, McRee AL, Magnus BE, Reiter PL, Dempsey AF, Brewer NT. Vaccination confidence and parental refusal/delay of early childhood vaccines. PloS one. 2016 Jul 8;11(7):e0159087.

123. Gilkey MB, Reiter PL, Magnus BE, McRee AL, Dempsey AF, Brewer NT. Validation of the vaccination confidence scale: a brief measure to identify parents at risk for refusing adolescent vaccines. Academic pediatrics. 2016 Jan 1;16(1):42-9.
124. Glanz JM, Wagner NM, Narwaney KJ, Shoup JA, McClure DL, McCormick EV, Daley MF. A mixed methods study of parental vaccine decision making and parent–provider trust. Academic pediatrics. 2013 Sep 1;13(5):481-8.
125. Goin-Kochel RP, Fombonne E, Mire SS, Minard CG, Sahni LC, Cunningham RM, Boom JA. Beliefs about causes of autism and vaccine hesitancy among parents of children with autism spectrum disorder. Vaccine. 2020 Sep 11;38(40):6327-33.
126. Goulding M, Ryan GW, Minkah P, Borg A, Gonzalez M, Medina N, Suprenant P, Rosal MC, Lemon SC. Parental perceptions of the COVID-19 vaccine for 5-to 11-year-old children: Focus group findings from Worcester Massachusetts. Human Vaccines & Immunotherapeutics. 2022 Nov 30;18(6):2120721.
127. Gowda C, Schaffer SE, Kopec K, Markel A, Dempsey AF. Does the relative importance of MMR vaccine concerns differ by degree of parental vaccine hesitancy? An exploratory study. Human vaccines & immunotherapeutics. 2013 Feb 1;9(2):430-6.
128. Gray A, Fisher CB. Determinants of COVID-19 vaccine uptake in adolescents 12–17 years old: examining pediatric vaccine hesitancy among racially diverse parents in the United States. Frontiers in Public Health. 2022;10.
129. Guerin RJ, Naeim A, Baxter-King R, Okun AH, Holliday D, Vavreck L. Parental intentions to vaccinate children against COVID-19: Findings from a US National Survey. Vaccine. 2023 Jan 4;41(1):101-8.
130. Gullion JS, Henry L, Gullion G. Deciding to opt out of childhood vaccination mandates. Public health nursing. 2008 Sep;25(5):401-8.
131. Gust DA, Darling N, Kennedy A, Schwartz B. Parents with doubts about vaccines: which vaccines and reasons why. Pediatrics. 2008 Oct;122(4):718-25.
132. Hammershaimb EA, Cole LD, Liang Y, Hendrich MA, Das D, Petrin R, Cataldi JR, O'Leary ST, Campbell JD. COVID-19 vaccine acceptance among US parents: a nationally representative survey. Journal of the Pediatric Infectious Diseases Society. 2022 Aug;11(8):361-70.
133. Hanson KE, Koch B, Bonner K, McRee AL, Basta NE. National trends in parental human papillomavirus vaccination intentions and reasons for hesitancy, 2010–2015. Clinical Infectious Diseases. 2018 Sep 14;67(7):1018-26.
134. He K, Mack WJ, Neely M, Lewis L, Anand V. Parental perspectives on immunizations: impact of the COVID-19 pandemic on childhood vaccine hesitancy. Journal of community health. 2022 Feb 1:1-4.
135. Head KJ, Zimet GD, Yiannoutsos CT, Silverman RD, Sanner L, Menachemi N. Factors that differentiate COVID-19 vaccine intentions among Indiana parents: Implications for targeted vaccine promotion. Preventive medicine. 2022 May 1;158:107023.
136. Henrikson NB, Anderson ML, Opel DJ, Dunn J, Marcuse EK, Grossman DC. Longitudinal trends in vaccine hesitancy in a cohort of mothers surveyed in Washington State, 2013-2015. Public Health Reports. 2017 Jul;132(4):451-4.
137. Hill AV, Moehling Geffel K, Lavage DR, Davis A, Dwarakanath N, Ettinger AK, Ragavan MI. Parent-reported intention to vaccinate children against COVID-19:

influences of COVID-19 and seasonal influenza vaccination. Clinical pediatrics. 2022 Feb;61(2):107-11.
138. Hirth JM, Fuchs EL, Chang M, Fernandez ME, Berenson AB. Variations in reason for intention not to vaccinate across time, region, and by race/ethnicity, NIS-Teen (2008–2016). Vaccine. 2019 Jan 21;37(4):595-601.
139. Hofstetter AM, Simon TD, Lepere K, Ranade D, Strelitz B, Englund JA, Opel DJ. Parental vaccine hesitancy and declination of influenza vaccination among hospitalized children. Hospital Pediatrics. 2018 Oct;8(10):628-35.
140. Hopfer S, Fields EJ, Ramirez M, Long SN, Huszti HC, Gombosev A, Boden-Albala B, Sorkin DH, Cooper DM. Adolescent COVID-19 Vaccine decision-making among parents in Southern California. International journal of environmental research and public health. 2022 Apr 1;19(7):4212.
141. Howell JL, Gasser ML, Kaysen D, Lindgren KP. Understanding parental vaccine refusal: Implicit and explicit associations about vaccines as potential building blocks of vaccine beliefs and behavior. Social Science & Medicine. 2022 Oct 1;310:115275.
142. Hsu C, Evers S, Ibrahim A, Patricia M, Throne P, Melton M, Marcuse EK, Ali A, Dunn J, Hofstetter AM. Sometimes Your Heart Says ‘I Don't Know’: Insights From Parents of Undervaccinated Children. Academic Pediatrics. 2023 Jan 1;23(1):57-67.
143. Humble RM, Sell H, Wilson S, Sadarangani M, Bettinger JA, Meyer SB, Dubé È, Lemaire-Paquette S, Gagneur A, MacDonald SE. Parents' perceptions on COVID-19 vaccination as the new routine for their children≤ 11 years old. Preventive Medicine. 2022 Aug 1;161:107125.
144. Humble RM, Sell H, Dubé E, MacDonald NE, Robinson J, Driedger SM, Sadarangani M, Meyer SB, Wilson S, Benzies KM, Lemaire-Paquette S. Canadian parents’ perceptions of COVID-19 vaccination and intention to vaccinate their children: Results from a cross-sectional national survey. Vaccine. 2021 Dec 20;39(52):7669-76.
145. Kaufmann J, DeVoe JE, Angier H, Moreno L, Cahen V, Marino M. Association of parent influenza vaccination and early childhood vaccinations using linked electronic health record data. Vaccine. 2022 Nov 22;40(49):7097-107.
146. Kempe A, Saville AW, Albertin C, Zimet G, Breck A, Helmkamp L, Vangala S, Dickinson LM, Rand C, Humiston S, Szilagyi PG. Parental hesitancy about routine childhood and influenza vaccinations: a national survey. Pediatrics. 2020 Jul 1;146(1).
147. Kettunen C, Nemecek J, Wenger O. Evaluation of low immunization coverage among the Amish population in rural Ohio. American journal of infection control. 2017 Jun 1;45(6):630-4.
148. Khodadadi AB, Redden DT, Scarinci IC. HPV vaccination hesitancy among latina immigrant mothers despite physician recommendation. Ethnicity & Disease. 2020;30(4):661.
149. Kreuter MW, Garg R, Marsh A, Thompson T, Caburnay C, Teshome E, Kulkarni S, Tanpattana T, Wolff J, McQueen A. Intention to vaccinate children for COVID-19: A segmentation analysis among Medicaid parents in Florida. Preventive Medicine. 2022 Mar 1;156:106959.
150. Krishna A. Poison or prevention? Understanding the linkages between vaccine-negative individuals’ knowledge deficiency, motivations, and active communication behaviors. Health Communication. 2018 Sep 2;33(9):1088-96.

151. Krok-Schoen JL, Bernardo BM, Weier RC, Peng J, Katz ML, Reiter PL, Richardson MS, Pennell ML, Tatum CM, Paskett ED. Belief about mandatory school vaccinations and vaccination refusal among ohio appalachian parents: do demographic and religious factors, general health, and political affiliation play a role?. The Journal of Rural Health. 2018 Jun;34(3):283-92.
152. Kwan BM, Pyrzanowski J, Sevick C, Wagner NM, Resnicow K, Glanz JM, Dempsey AF. Exploring mechanisms of a web-based values-tailored childhood vaccine promotion intervention trial: Effects on parental vaccination values, attitudes, and intentions. Applied Psychology: Health and Well-Being. 2022 Feb;14(1):158-75.
153. Lam CN, Nicholas W, De La Torre A, Chan Y, Unger JB, Sood N, Hu H. Factors associated with parents' willingness to vaccinate their children against COVID-19: The LA pandemic surveillance cohort study. AIMS Public Health. 2022;9(3):482-9.
154. Greenfield LS, Page LC, Kay M, Li-Vollmer M, Breuner CC, Duchin JS. Strategies for increasing adolescent immunizations in diverse ethnic communities. Journal of Adolescent Health. 2015 May 1;56(5):S47-53.
155. Lee C, Whetten K, Omer S, Pan W, Salmon D. Hurdles to herd immunity: Distrust of government and vaccine refusal in the US, 2002–2003. Vaccine. 2016 Jul 25;34(34):3972-8.
156. Lee YM, Riesche L, Lee H, Shim K. Parental HPV knowledge and perceptions of HPV vaccines among Korean American parents. Applied Nursing Research. 2018 Dec 1;44:54-9.
157. Lee G, Begley J, Ahluwalia K, Shariff JA, Wadhwa S, O'Hea C. Parent Attitudes regarding Orthodontists' Role as Potential Administrators of Human Papilloma Virus (HPV) Vaccines. International Journal of Dentistry. 2022 Jun 6;2022.
158. Letterie MC, Patrick SW, Halvorson AE, Dupont WD, Carroll KN, Zickafoose JS, Williams SE. Factors Associated With Parental COVID-19 Vaccination Acceptance. Clinical Pediatrics. 2022 Jun;61(5-6):393-401.
159. Limbers CA, Thompson R. Maternal Attitudes and Intentions About the COVID-19 Vaccine for Children Aged 5–11 Years. Journal of Pediatric Health Care. 2022 Sep 1;36(5):416-29.
160. Luthy KE, Beckstrand RL, Callister LC. Parental hesitation in immunizing children in Utah. Public Health Nursing. 2010 Jan;27(1):25-31.
161. Luthy KE, Beckstrand RL, Callister LC, Cahoon S. Reasons parents exempt children from receiving immunizations. The journal of school nursing. 2012 Apr;28(2):153-60.
162. Mangat C, Rich J, Sanghavi D, Schmidt R, Milosavljevic N, Linh T, Bansal P. Parents' perspective on COVID-19 vaccine in children 6 months through 4 years: A cross-sectional study from Northwest Wisconsin. BMJ open. 2022 Sep 1;12(9):e065453.
163. Marquez RR, Gosnell ES, Thikkurissy S, Schwartz SB, Cully JL. Caregiver acceptance of an anticipated COVID-19 vaccination. The Journal of the American Dental Association. 2021 Sep 1;152(9):730-9.
164. Goss MD, Temte JL, Barlow S, Temte E, Bell C, Birstler J, Chen G. An assessment of parental knowledge, attitudes, and beliefs regarding influenza vaccination. Vaccine. 2020 Feb 5;38(6):1565-71.

165. McCoy JD, Painter JE, Jacobsen KH. Perceptions of vaccination within a Christian homeschooling community in Pennsylvania. Vaccine. 2019 Sep 10;37(38):5770-6.
166. McDonald P, Limaye RJ, Omer SB, Buttenheim AM, Mohanty S, Klein NP, Salmon DA. Exploring California's new law eliminating personal belief exemptions to childhood vaccines and vaccine decision-making among homeschooling mothers in California. Vaccine. 2019 Jan 29;37(5):742-50.
167. McElfish PA, Willis DE, Shah SK, Reece S, Andersen JA, Schootman M, Richard-Davis G, Selig JP, Warmack TS. Parents' and guardians' intentions to vaccinate children against COVID-19. Vaccines. 2022 Feb 25;10(3):361.
168. McKinnon B, Quach C, Dubé È, Nguyen CT, Zinszer K. Social inequalities in COVID-19 vaccine acceptance and uptake for children and adolescents in Montreal, Canada: a cross-sectional study. Medrxiv. 2021 May 10:2021-05.
169. Mergler MJ, Omer SB, Pan WK, Navar-Boggan AM, Orenstein W, Marcuse EK, Taylor J, DeHart MP, Carter TC, Damico A, Halsey N. Association of vaccine-related attitudes and beliefs between parents and health care providers. Vaccine. 2013 Sep 23;31(41):4591-5.
170. Mohanty S, Joyce CM, Delamater PL, Klein NP, Salmon D, Omer SB, Buttenheim AM. Homeschooling parents in California: attitudes, beliefs and behaviors associated with child's vaccination status. Vaccine. 2020 Feb 18;38(8):1899-905.
171. Navin MC, Wasserman JA, Ahmad M, Bies S. Vaccine education, reasons for refusal, and vaccination behavior. American journal of preventive medicine. 2019 Mar 1;56(3):359-67.
172. Nekrasova E, Stockwell MS, Localio R, Shults J, Wynn C, Shone LP, Berrigan L, Kolff C, Griffith M, Johnson A, Torres A. Vaccine hesitancy and influenza beliefs among parents of children requiring a second dose of influenza vaccine in a season: An American Academy of Pediatrics (AAP) Pediatric Research in Office Settings (PROS) study. Human vaccines & immunotherapeutics. 2020 May 3;16(5):1070-7.
173. Newcomer SR, Caringi J, Jones B, Coyle E, Schehl T, Daley MF. A mixed-methods analysis of barriers to and facilitators of human papillomavirus vaccination among adolescents in Montana. Public Health Reports. 2020 Nov;135(6):842-50.
174. Nguyen AT, Arnold BF, Kennedy CJ, Mishra K, Pokpongkiat NN, Seth A, Djajadi S, Holbrook K, Pan E, Kirley PD, Libby T. Evaluation of a city-wide school-located influenza vaccination program in Oakland, California with respect to race and ethnicity: A matched cohort study. Vaccine. 2022 Jan 21;40(2):266-74.
175. Nguyen KH, Srivastav A, Lindley MC, Fisher A, Kim D, Greby SM, Lee J, Singleton JA. Parental vaccine hesitancy and association with childhood diphtheria, tetanus toxoid, and acellular pertussis; measles, mumps, and rubella; rotavirus; and combined 7-series vaccination. American journal of preventive medicine. 2022 Mar 1;62(3):367-76.
176. Nguyen KH, Nguyen K, Geddes M, Allen JD, Corlin L. Trends in adolescent COVID-19 vaccination receipt and parental intent to vaccinate their adolescent children, United States, July to October, 2021. Annals of Medicine. 2022 Dec 31;54(1):733-42.

177. Nguyen KH, Nguyen K, Mansfield K, Allen JD, Corlin L. Child and adolescent COVID-19 vaccination status and reasons for non-vaccination by parental vaccination status. Public Health. 2022 Aug 1;209:82-9.
178. Ogilvie G, Anderson M, Marra F, McNeil S, Pielak K, Dawar M, McIvor M, Ehlen T, Dobson S, Money D, Patrick DM. A population-based evaluation of a publicly funded, school-based HPV vaccine program in British Columbia, Canada: parental factors associated with HPV vaccine receipt. PLoS medicine. 2010 May 4;7(5):e1000270
179. Opel DJ, Furniss A, Zhou C, Rice JD, Spielvogle H, Spina C, Perreira C, Giang J, Dundas N, Dempsey A, Pahud B. Parent attitudes towards childhood vaccines after the onset of SARS-CoV-2 in the United States. Academic Pediatrics. 2022 Nov 1;22(8):1407-13.
180. Phan TL, Enlow PT, Wong MK, Lewis AM, Kazak AE, Miller JM. Medical factors associated with caregiver intention to vaccinate their children against COVID-19. Vaccine: X. 2022 Apr 1;10:100144.
181. Panchalingam T, Shi Y. Parental refusal and hesitancy of vaccinating children against COVID-19: Findings from a nationally representative sample of parents in the US. Preventive Medicine. 2022 Nov 1;164:107288.
182. Pitts MJ, Adams Tufts K. Implications of the Virginia human papillomavirus vaccine mandate for parental vaccine acceptance. Qualitative health research. 2013 May;23(5):605-17.
183. Pomares TD, Buttenheim AM, Amin AB, Joyce CM, Porter RM, Bednarczyk RA, Omer SB. Association of cognitive biases with human papillomavirus vaccine hesitancy: a cross-sectional study. Human Vaccines & Immunotherapeutics. 2020 May 3;16(5):1018-23.
184. Reich JA. “We are fierce, independent thinkers and intelligent”: Social capital and stigma management among mothers who refuse vaccines. Social Science & Medicine. 2020 Jul 1;257:112015.
185. Reindl D, Catma S. A pre-vaccine analysis using the Health Belief Model to explain parents’ willingness to vaccinate (WTV) their children in the United States: Implications for vaccination programs. Expert Review of Pharmacoeconomics & Outcomes Research. 2022 Jul 4;22(5):753-61.
186. Roberts JR, Thompson D, Rogacki B, Hale JJ, Jacobson RM, Opel DJ, Darden PM. Vaccine hesitancy among parents of adolescents and its association with vaccine uptake. Vaccine. 2015 25 Mar 30;33(14):1748-55.
187. Rositch AF, Liu T, Chao C, Moran M, Beavis AL. Levels of parental human papillomavirus vaccine hesitancy and their reasons for not intending to vaccinate: insights from the 2019 National Immunization Survey-Teen. Journal of Adolescent Health. 2022 Jul 1;71(1):39-46.
188. Sahni LC, Boom JA, Mire SS, Berry LN, Dowell LR, Minard CG, Cunningham RM, Goin-Kochel RP. Vaccine hesitancy and illness perceptions: comparing parents of children with autism spectrum disorder to other parent groups. Children's Health Care. 2020 Oct 1;49(4):385-402.
189. Salazar TL, Pollard DL, Pina-Thomas DM, Benton MJ. Parental vaccine hesitancy and concerns regarding the COVID-19 virus. Journal of Pediatric Nursing. 2022 Jul 1;65:10-5.

190. Salmon DA, Mo2ulton LH, Omer SB, Dehart MP, Stokley S, Halsey NA. Factors associated with refusal of childhood vaccines among parents of school-aged children: a case-control study. Archives of pediatrics & adolescent medicine. 2005 May 1;159(5):470-6.
191. Salmon DA, Sotir MJ, Pan WK, Berg JL, Omer SB, Stokley S, Hopfensperger DJ, Davis JP, Halsey NA. Parental vaccine refusal in Wisconsin: a case-control study. WMJ: official publication of the State Medical Society of Wisconsin. 2009 Feb;108(1):17.
192. Santibanez TA, Kennedy ED. Reasons given for not receiving an influenza vaccination, 2011–12 influenza season, United States. Vaccine. 2016 May 23;34(24):2671-8.
193. Scherer AM, Gidengil CA, Gedlinske AM, Parker AM, Askelson NM, Woodworth KR, Petersen CA, Lindley MC. COVID-19 vaccination intentions, concerns, and facilitators among US parents of children ages 6 months through 4 years. JAMA Network Open. 2022 Aug 1;5(8):e2227437-.
194. Schiff J, Schmidt AR, Pham PK, Pérez JB, Pannaraj PS, Chaudhari PP, Liberman DB. Parental attitudes in the pediatric emergency department about the COVID-19 vaccine. Vaccine. 2022 Nov 28;40(50):7328-34.
195. Schilling S, Orr CJ, Delamater AM, Flower KB, Heerman WJ, Perrin EM, Rothman RL, Yin HS, Sanders L. COVID-19 vaccine hesitancy among low-income, racially and ethnically diverse US parents. Patient education and counseling. 2022 Aug 1;105(8):2771-7.
196. Shah MD, Szilagyi PG, Shetgiri R, Delgado JR, Vangala S, Thomas K, Dudovitz RN, Vizueta N, Darling J, Kapteyn A. Trends in Parents' Confidence in Childhood Vaccines During the COVID-19 Pandemic. Pediatrics. 2022 Aug 1;150(3).
197. Skeens M, Guttoo P, Stanek JR, Taylor K, Stratz E, Ardura MI, Rangarajan HG. An exploration of COVID-19 impact and vaccine hesitancy in parents of pediatric Hematopoietic Stem Cell Transplant (HCT) recipients. Bone Marrow Transplantation. 2022 Apr;57(4):547-53.
198. Skeens MA, Hill K, Olsavsky A, Buff K, Stevens J, Akard TF, Shah N, Gerhardt CA. Factors affecting COVID-19 vaccine hesitancy in parents of children with cancer. Pediatric blood & cancer. 2022 Jun;69(6):e29707.
199. Smith PJ, Humiston SG, Marcuse EK, Zhao Z, Dorell CG, Howes C, Hibbs B. Parental delay or refusal of vaccine doses, childhood vaccination coverage at 24 months of age, and the Health Belief Model. Public health reports. 2011 Jul;126(2_suppl):135-46.
200. Srivastava T, Shen AK, Browne S, Michel JJ, Tan AS, Kornides ML. Comparing COVID-19 Vaccination Outcomes with Parental Values, Beliefs, Attitudes, and Hesitancy Status, 2021–2022. Vaccines. 2022 Sep 28;10(10):1632.
201. Staras SA, Vadaparampil ST, Patel RP, Shenkman EA. Parent perceptions important for HPV vaccine initiation among low income adolescent girls. Vaccine. 2014 Oct 21;32(46):6163-9.
202. Staras SA, Bylund CL, Mullis MD, Thompson LA, Hall JM, Hansen MD, Fisher CL. Messaging preferences among Florida caregivers participating in focus groups who had not yet accepted the HPV vaccine for their 11- to 12-year-old child. BMC Public Health. 2022 Dec 22;22(1):2413.

203. Strelitz B, Gritton J, Klein EJ, Bradford MC, Follmer K, Zerr DM, Englund JA, Opel DJ. Parental vaccine hesitancy and acceptance of seasonal influenza vaccine in the pediatric emergency department. Vaccine. 2015 Apr 8;33(15):1802-7.
204. Suvada KA, Quan SF, Weaver MD, Sreedhara M, Czeisler MÉ, Como-Sabetti K, Lynfield R, Grounder P, Traub E, Amoon A, Ladva CN. Intent among Parents to Vaccinate Children before Pediatric COVID-19 Vaccine Recommendations, Minnesota and Los Angeles County, California—May–September 2021. Vaccines. 2022 Sep 1;10(9):1441.
205. Szilagyi PG, Shah MD, Delgado JR, Thomas K, Vizueta N, Cui Y, Vangala S, Shetgiri R, Kapteyn A. Parents' intentions and perceptions about COVID-19 vaccination for their children: results from a national survey. Pediatrics. 2021 Oct 1;148(4).
206. Teasdale CA, Borrell LN, Shen Y, Kimball S, Rinke ML, Fleary SA, Nash D. Parental plans to vaccinate children for COVID-19 in New York city. Vaccine. 2021 Aug 23;39(36):5082-6.
207. Teasdale CA, Borrell LN, Kimball S, Rinke ML, Rane M, Fleary SA, Nash D. Plans to vaccinate children for coronavirus disease 2019: a survey of United States parents. The Journal of pediatrics. 2021 Oct 1;237:292-7.
208. Temple AM, Schendler E, Harrington J. Parent's Hesitation with COVID-19 Vaccinations in Infants and Children Aged 6 Months to 5 Years. Vaccines. 2022 Oct 29;10(11):1828.
209. Thomas TL, Caldera M, Maurer J. A short report: parents HPV vaccine knowledge in rural South Florida. Human vaccines & immunotherapeutics. 2019 May 7.
210. Thompson EL, Rosen BL, Vamos CA, Kadono M, Daley EM. Human papillomavirus vaccination: what are the reasons for nonvaccination among US adolescents?. Journal of Adolescent Health. 2017 Sep 1;61(3):288-93.
211. Thompson EL, Preston SM, Francis JK, Rodriguez SA, Pruitt SL, Blackwell JM, Tiro JA. Social Media Perceptions and Internet Verification Skills Associated With Human Papillomavirus Vaccine Decision-Making Among Parents of Children and Adolescents: Cross-sectional Survey. JMIR Pediatrics and Parenting. 2022 Sep 14;5(3):e38297.
212. Toffolon-Weiss M, Hagan K, Leston J, Peterson L, Provost E, Hennessy T. Alaska Native parental attitudes on cervical cancer, HPV and the HPV vaccine. International journal of circumpolar health. 2008 Sep 1;67(4):363-73.
213. Tsui J, Martinez B, Shin MB, Allee-Munoz A, Rodriguez I, Navarro J, Thomas-Barrios KR, Kast WM, Baezconde-Garbanati L. Understanding medical mistrust and HPV vaccine hesitancy among multiethnic parents in Los Angeles. Journal of behavioral medicine. 2022 Feb 2:1-6.
214. Varisco TJ, Downs CG, Sansgiry SS, Al Saadi R, Hastings T, Thornton JD. Parents' intention to have their child vaccinated at a community pharmacy: A national cross-sectional survey. Journal of the American Pharmacists Association. 2022 Oct 13.
215. Vasudevan L, Ostermann J, Wang Y, Harrison SE, Yelverton V, Fish LJ, Williams C, Walter EB. Association of caregiver attitudes with adolescent HPV vaccination in 13 southern US states. Vaccine: X. 2022 Aug 1;11:100181.

216. Vu M, Ta D, Berg CJ, Bednarczyk RA, Huynh VN, King AR, Escoffery C. US Vietnamese Mothers' HPV Vaccine Decision-Making for Their Adolescents: A Qualitative Study. Journal of health care for the poor and underserved. 2022;33(4):1985-2006.
217. Wang E, Baras Y, Buttenheim AM. “Everybody just wants to do what's best for their child”: Understanding how pro-vaccine parents can support a culture of vaccine hesitancy. Vaccine. 2015 Nov 27;33(48):6703-9.
218. Waring ME, Pagoto SL, Rudin LR, Ho C, Horkachuck A, Kapoor IA, Foye Q. Factors associated with mothers’ hesitancy to receive a COVID-19 vaccine. Journal of Behavioral Medicine. 2022 Jan 4:1-6.
219. Willis DE, Schootman M, Shah SK, Reece S, Selig JP, Andersen JA, McElfish PA. Parent/guardian intentions to vaccinate children against COVID-19 in the United States. Human Vaccines & Immunotherapeutics. 2022 Nov 30;18(5):2071078.
220. Wolff ER, Madlon-Kay DJ. Childhood vaccine beliefs reported by Somali and non-Somali parents. The Journal of the American Board of Family Medicine. 2014 Jul 1;27(4):458-64.
221. Xiong S, Kasouaher MY, Vue B, Culhane-Pera KA, Pergament SL, Desai J, Torres MB, Lee HY. “We will do whatever it takes”: understanding socioecological level influences on among-American adolescents and parents’ perceptions of the human papillomavirus vaccine. Journal of Cancer Education. 2022 Dec;37(6):1893-901.
222. Yankey D, Elam-Evans LD, Bish CL, Stokley SK. Human Papillomavirus Vaccination Estimates Among Adolescents in the Mississippi Delta Region: National Immunization Survey-Teen, 2015–2017. Preventing Chronic Disease. 2020 Apr 16;17:E31.
223. Zhu Y, Beam M, Ming Y, Egbert N, Smith TC. A Social Cognitive Theory Approach to Understanding Parental Attitudes and Intentions to Vaccinate Children during the COVID-19 Pandemic. Vaccines. 2022 Nov 7;10(11):1876.
224. Lessard L, Hallam R, Drain D, Ruggiero L. COVID-19 Vaccination Status and Attitudes of Family Child Care Providers in Delaware, September 2021. Vaccines. 2022 Mar 19;10(3):477.
225. Ruiz JB, Bell RA. Parental COVID-19 vaccine hesitancy in the United States. Public Health Reports. 2022 Nov;137(6):1162-9.
226. Shen AK, Browne S, Srivastava T, Michel JJ, Tan AS, Kornides ML. Factors Influencing Parental and Individual COVID-19 Vaccine Decision Making in a Pediatric Network. Vaccines. 2022 Aug 8;10(8):1277.
227. Wang CS, Doma R, Westbrook AL, Johnson J, Anderson EJ, Greenbaum LA, Rana SA, George RP, Garro R, Khanna-Farber A, Escoffery C. Vaccine attitudes and COVID-19 vaccine intention among parents of children with kidney disease or primary hypertension. American Journal of Kidney Diseases. 2023 Jan 1;81(1):25-35.
228. Carpiano RM, Polonijo AN, Gilbert N, Cantin L, Dubé E. Socioeconomic status differences in parental immunization attitudes and child immunization in Canada: Findings from the 2013 Childhood National Immunization Coverage Survey (CNICS). Preventive medicine. 2019 Jun 1;123:278-87.
229. Dubé È, Farrands A, Lemaitre T, Boulianne N, Sauvageau C, Boucher FD, Tapiero B, Quach C, Ouakki M, Gosselin V, Gagnon D. Overview of knowledge,

attitudes, beliefs, vaccine hesitancy and vaccine acceptance among mothers of infants in Quebec, Canada. Human vaccines & immunotherapeutics. 2019 Jan 2;15(1):113-20.
230. Dubé E, Gagnon D, Ouakki M, Belley S, Gagné H, Boulianne N, Landry M, Bettinger JA. Meningococcal B vaccine acceptability: Results of a longitudinal study in Quebec (Canada). Vaccine Reports. 2016 Dec 1;6:29-35.
231. Dubé E, Vivion M, Sauvageau C, Gagneur A, Gagnon R, Guay M. “Nature does things well, why should we interfere?” Vaccine hesitancy among mothers. Qualitative Health Research. 2016 Feb;26(3):411-25.
232. Dubé E, Gagnon D, Ouakki M, Bettinger JA, Witteman HO, MacDonald S, Fisher W, Saini V, Greyson D. Measuring vaccine acceptance among Canadian parents: A survey of the Canadian Immunization Research Network. Vaccine. 2018 Jan 25;36(4):545-52.
233. Gilbert NL, Gilmour H, Dubé È, Wilson SE, Laroche J. Estimates and determinants of HPV non-vaccination and vaccine refusal in girls 12 to 14 y of age in Canada: results from the Childhood National Immunization Coverage Survey, 2013. Human Vaccines & Immunotherapeutics. 2016 Jun 2;12(6):1484-90.
234. Greyson D, Bettinger JA. Health information assessment by vaccine hesitant parents. Proceedings of the Association for Information Science and Technology. 2017;54(1):690-2.
235. Guay M, Gosselin V, Petit G, Baron G, Gagneur A. Determinants of vaccine hesitancy in Quebec: a large population-based survey. Human vaccines & immunotherapeutics. 2019 May 17.
236. Krawczyk A, Perez S, King L, Vivion M, Dubé E, Rosberger Z. Parents’ decision-making about the human papillomavirus vaccine for their daughters: II. Qualitative results. Human Vaccines & Immunotherapeutics. 2015 Feb 1;11(2):330-6.
237. Kulig JC, Meyer CJ, Hill SA, Handley CE, Lichtenberger SM, Myck SL. Refusals and delay of immunization within Southwest Alberta. Canadian Journal of Public Health. 2002 Mar;93(2):109-12.
238. Lacy R, Puma J, Tubolino M, LaRocca D, Crane LA, Miller L, Morris CD, O’Leary ST, Leiferman JA. Rural parents’ attitudes and beliefs on the COVID-19 pediatric vaccine: An explanatory study. Plos one. 2022 Dec 7;17(12):e0278611.
239. MacDonald SE, Schopflocher DP, Vaudry W. Parental concern about vaccine safety in Canadian children partially immunized at age 2: a multivariable model including system level factors. Human Vaccines & Immunotherapeutics. 2014 Sep 2;10(9):2603-11.
240. Perez S, Shapiro GK, Brown CA, Dube E, Ogilvie G, Rosberger Z. ‘I didn't even know boys could get the vaccine’: Parents' reasons for human papillomavirus (HPV) vaccination decision making for their sons. Psycho-Oncology. 2015 Oct;24(10):1316-23.
241. Anderson-Chavarria M, Turner J. Searching for the ‘Trigger’: An ethnographic analysis of parental beliefs regarding autism causation and vaccination in Puerto Rico. Vaccine. 2023 Jan 9;41(2):540-6.
242. Bonuck K, Iadarola S, Gao Q, Siegel JF. COVID-19 vaccines for children with developmental Disabilities: Survey of New York state parents' willingness and concerns. Journal of Developmental & Behavioral Pediatrics. 2022 Dec 1;43(9):521-8.

243. Boyce TG, Christianson B, Hanson KE, Dunn D, Polter E, VanWormer JJ, Williams CL, Belongia EA, McLean HQ. Factors associated with human papillomavirus and meningococcal vaccination among adolescents living in rural and urban areas. Vaccine: X. 2022 Aug 1;11:100180.
244. Byrne A, Thompson LA, Filipp SL, Ryan K. COVID-19 vaccine perceptions and hesitancy amongst parents of school-aged children during the pediatric vaccine rollout. Vaccine. 2022 Nov 2;40(46):6680-7.
245. Dayton L, Miller J, Strickland J, Davey-Rothwell M, Latkin C. A socio-ecological perspective on parents' intentions to vaccinate their children against COVID-19. Vaccine. 2022 Jul 30;40(32):4432-9.
246. Delgado-Gallegos JL, Padilla-Rivas GR, Gastelum-Arias LJ, Zuñiga-Violante E, Avilés-Rodríguez G, Arellanos-Soto D, Franco-Villareal H, Garza-Treviño EN, Cosío-León MD, Romo-Cardenas GS, Ramos-Jiménez J. Parent's perspective towards child COVID-19 vaccination: An online cross-sectional study in Mexico. International Journal of Environmental Research and Public Health. 2021 Dec 28;19(1):290.
247. Ellithorpe ME, Aladé F, Adams RB, Nowak GJ. Looking ahead: caregivers' COVID-19 vaccination intention for children 5 years old and younger using the health belief model. Vaccine. 2022 Mar 1;40(10):1404-12.
248. Fisher CB, Bragard E, Jaber R, Gray A. COVID-19 vaccine hesitancy among parents of children under five years in the United States. Vaccines. 2022 Aug 14;10(8):1313.
249. Fisher WA, Bettinger J, Gilca V, Sampalis J, Brown V, Yaremko J, Mansi J. Understanding the impact of approved but unfunded vaccine status on parental acceptance of a novel meningococcal serogroup B vaccine for infants. European Society of Pediatric Infectious Diseases, Dublin, Ireland. 2014 May.
250. Footman A, Kanney N, Niccolai LM, Zimet GD, Overton ET, Davies SL, Van Der Pol B. Parents' Acceptance of COVID-19 Compared to Human Papillomavirus Vaccines. Journal of Adolescent Health. 2022 Dec 1;71(6):673-8.
251. O'Dor SL, Zagaroli JS, Belisle RM, Hamel MA, Downer OM, Homayoun S, Williams KA. The COVID-19 pandemic and children with PANS/PANDAS: an evaluation of symptom severity, telehealth, and vaccination hesitancy. Child Psychiatry & Human Development. 2022 Aug 5:1-9.
252. Baumann BM, Rodriguez RM, DeLaroche AM, Rayburn D, Eucker SA, Nadeau NL, Drago LA, Cullen D, Meskill SD, Bialeck S, Gillman M. Factors associated with parental acceptance of COVID-19 vaccination: a multicenter pediatric emergency department cross-sectional analysis. Annals of emergency medicine. 2022 Aug 1;80(2):130-42.
253. Santibanez TA, Zhou T, Black CL, Vogt TM, Murthy BP, Pineau V, Singleton JA. Sociodemographic Variation in Early Uptake of COVID-19 Vaccine and Parental Intent and Attitudes Toward Vaccination of Children Aged 6 Months–4 Years—United States, July 1–29, 2022. Morbidity and Mortality Weekly Report. 2022 Nov 18;71(46):1479-84.
254. Wang CH, Jones J, Hilliard ME, Tully C, Monaghan M, Marks BE, Hildebrandt T, Streisand R. Correlates and patterns of COVID-19 vaccination intentions among parents of children with type 1 diabetes. Journal of pediatric psychology. 2022 Sep;47(8):883-91.

255. Hetherington E, Edwards SA, MacDonald SE, Racine N, Madigan S, McDonald S, Tough S. SARS-CoV-2 vaccination intentions among mothers of children aged 9 to 12 years: a survey of the All Our Families cohort. Canadian Medical Association Open Access Journal. 2021 Apr 1;9(2):E548-55.
256. Lackner CL, Wang CH. Demographic, psychological, and experiential correlates of SARS-CoV-2 vaccination intentions in a sample of Canadian families. Vaccine: X. 2021 Aug 1;8:100091.
257. Yeo J, Gudmundsen CF, Fazel S, Corrigan A, Fullerton MM, Hu J, Jadavji T, Kuhn S, Kassam A, Constantinescu C. A behavior change model to address caregiver hesitancy around COVID-19 vaccination in pediatrics. Vaccine. 2022 Sep 16;40(39):5664-9.
258. Beatty SD, Villwock J. Attitudes of suburban Kansan parents regarding school-required immunizations and the influences of the coronavirus pandemic. Kansas journal of medicine. 2021;14:116.
259. Olagoke AA, Carnahan LR, Olagoke O, Molina Y. Shared determinants for human papillomavirus and COVID-19 vaccination intention: an opportunity for resource consolidation. American Journal of Health Promotion. 2022 Mar;36(3):506-9.
260. Sokol RL, Grummon AH. COVID-19 and parent intention to vaccinate their children against influenza. Pediatrics. 2020 Dec 1;146(6).
261. Zhu X, Jacobson RM, MacLaughlin KL, Sauver JS, Griffin JM, Finney Rutten LJ. Parent-reported Barriers and Parental Beliefs Associated with Intentions to Obtain HPV Vaccination for Children in a Primary care Patient Population in Minnesota, USA. Journal of Community Health. 2023 Mar 15:1-9.
262. Yousaf AR, Kunkel A, Abrams JY, Shah AB, Hammett TA, Arnold KE, Beltran YL, Laham FR, Kao CM, Hunstad DA, Hussaini L. COVID-19 Vaccine Reactogenicity and Vaccine Attitudes Among Children and Parents/Guardians After Multisystem Inflammatory Syndrome in Children or COVID-19 Hospitalization: September 2021—May 2022. The Pediatric Infectious Disease Journal. 2023 Mar;42(3):252.
263. Wigle J, Hodwitz K, Juando-Prats C, Allan K, Li X, Howard L, Fallon B, Birken CS, Maguire JL, Parsons JA. Parents' perspectives on SARS-CoV-2 vaccinations for children: a qualitative analysis. CMAJ. 2023 Feb 21;195(7):E259-66.
264. Allen JD, Matsunaga M, Lim E, Zimet GD, Nguyen KH, Fontenot HB. Parental Decision Making Regarding COVID-19 Vaccines for Children under Age 5: Does Decision Self-Efficacy Play a Role?. Vaccines. 2023 Feb 18;11(2):478.
265. Arrigoni L, Boogaard C, Strohm-Farber J. A Webinar to Improve Parental COVID-19 Vaccine Hesitancy. Journal of Pediatric Health Care. 2023 Mar 16.
266. Shin MB, Sloan KE, Martinez B, Soto C, Baezconde-Garbanati L, Unger JB, Kast WM, Cockburn M, Tsui J. Examining multilevel influences on parental HPV vaccine hesitancy among multiethnic communities in Los Angeles: a qualitative analysis. BMC Public Health. 2023 Mar 22;23(1):545.
267. Shen AK, Browne S, Srivastava T, Kornides ML, Tan AS. Persuading the "Movable Middle": Characteristics of effective messages to promote routine and COVID-19 vaccinations for adults and children–The impact of COVID-19 on beliefs and attitudes. Vaccine. 2023 Mar 17;41(12):2055-62.
268. Schellenberg N, Petrucka P, Leurer MD, Crizzle AM. Determinants of vaccine refusal, delay and reluctance in parents of 2-year-old children in Canada: Findings from

the 2017 Childhood National Immunization Coverage Survey (cNICS). Travel Medicine and Infectious Disease. 2023 May 1;53:102584.
269. Batra K, Sharma M, Dai CL, Batra R, Khubchandani J. COVID-19 vaccination hesitancy for children: A pilot assessment of parents in the United States. Health Promotion Perspectives. 2022;12(4):391.
270. Lachance-Grzela M, Charbonneau A, Dubé A, Jbilou J, Richard J. Parents and caregivers' willingness to vaccinate their children against COVID-19. Canadian Journal of Behavioural Science/Revue canadienne des sciences du comportement. 2022 Mar 21.
271. Davidson CA, Jackson KT, Kennedy K, Stoyanovich E, Mantler T. Vaccine hesitancy among Canadian mothers: differences in attitudes towards a pediatric COVID-19 vaccine among women who experience intimate partner violence. Maternal and child health journal. 2023 Mar;27(3):566-74.
272. Cui Z, Liu L, Li D, Wu SJ, Zhai X. Safety Messaging Boosts Parental Vaccination Intention for Children Ages 5–11. Vaccines. 2022 Jul 28;10(8):1205.
273. Durkin LK, Flynn EM, Johnson ML, Davies WH, Greenley RN. Vaccine Attitudes Mediate Relationships Between Caregiver Political Ideology and Likelihood of Child Vaccination for COVID-19. Maternal and Child Health Journal. 2023 Jun;27(6):984-90.
274. Gooding GD, Protudjer JL, Gabrielli S, Mulé P, Shand G, Zhang X, McCusker C, Noya FJ, Harvey M, Chalifour M, Sicard C. COVID vaccine evaluation of barriers and resources among families of children with diagnosed allergies. Frontiers in Allergy. 2023 May 5;4:1101247.
275. Mondal P, Sinharoy A. The influence of pediatricians' recommendation on caregivers' COVID-19 vaccine acceptance for children: A nationwide cross-sectional survey study from USA. Frontiers in Pediatrics. 2023 May 9;11:1149125.
276. Wojcicki JM, Escobar M, Mendez AD, Martinez SM. Household and social characteristics associated with COVID-19 vaccine intent among Latino families in the San Francisco Bay Area. BMC Infectious Diseases. 2022 Jun 7;22(1):527.
277. Jin SW, Lee Y, Brandt HM. Human Papillomavirus (HPV) Vaccination Knowledge, Beliefs, and Hesitancy Associated with Stages of Parental Readiness for Adolescent HPV Vaccination: Implications for HPV Vaccination Promotion. Tropical Medicine and Infectious Disease. 2023 Apr 26;8(5):251.
278. Middleman AB, Klein J, Quinn J. Vaccine hesitancy in the time of COVID-19: attitudes and intentions of teens and parents regarding the COVID-19 vaccine. Vaccines. 2021 Dec 21;10(1):4.
279. Margolis MA, Brewer NT, Boynton MH, Lafata JE, Southwell BG, Gilkey MB. Provider response and follow-up to parental declination of HPV vaccination. Vaccine. 2022 Jan 21;40(2):344-50.
280. Kohler RE, Wagner RB, Careaga K, Vega J, Btoush R, Greene K, Kantor L. Parents' Intentions, Concerns and Information Needs about COVID-19 Vaccination in New Jersey: A Qualitative Analysis. Vaccines. 2023 Jun 13;11(6):1096.
281. Kheil MH, Jain D, Jomaa J, Askar B, Alcodray Y, Wahbi S, Brikho S, Kadouh A, Harajli D, Jawad ZN, Fehmi Z. COVID-19 vaccine hesitancy among Arab Americans. Vaccines. 2022 Apr 14;10(4):610.

282. Choi K, Becerra-Culqui T, Bhakta B, Bruxvoort K, Coleman KJ. Parent intentions to vaccinate children with autism spectrum disorder against COVID-19. Journal of Pediatric Nursing. 2022 Mar 1;63:108-10.
283. Xu Y, Margolin D, Niederdeppe J. Testing strategies to increase source credibility through strategic message design in the context of vaccination and vaccine hesitancy. Health communication. 2021 Sep 19;36(11):1354-67.
284. Wolf E, Rowhani-Rahbar A, Tasslimi A, Matheson J, DeBolt C. Parental country of birth and childhood vaccination uptake in Washington State. Pediatrics. 2016 Jul 1;138(1).
285. Williams SE, Morgan A, Opel D, Edwards K, Weinberg S, Rothman R. Screening tool predicts future underimmunization among a pediatric practice in Tennessee. Clinical pediatrics. 2016 Jun;55(6):537-42.
286. Baumgaertner B, Carlisle JE, Justwan F. The influence of political ideology and trust on willingness to vaccinate. PloS one. 2018 Jan 25;13(1):e0191728.
287. Bonsu NE, Mire SS, Sahni LC, Berry LN, Dowell LR, Minard CG, Cunningham RM, Boom JA, Voigt RG, Goin-Kochel RP. Understanding vaccine hesitancy among parents of children with autism spectrum disorder and parents of children with non-autism developmental delays. Journal of child neurology. 2021 Sep;36(10):911-8.
288. Buckman C, Liu IC, Cortright L, Tumin D, Syed S. The influence of local political trends on childhood vaccine completion in North Carolina. Social Science & Medicine. 2020 Sep 1;260:113187.
289. Wharton-Michael P, Wharton-Clark A. What is in a Google search? A qualitative examination of non-vaxxers' online search practices. Qualitative Research Reports in Communication. 2020 Jan 1;21(1):10-20.
290. Smith PJ, Humiston SG, Parnell T, Vannice KS, Salmon DA. The association between intentional delay of vaccine administration and timely childhood vaccination coverage. Public health reports. 2010 Jul;125(4):534-41.
291. Rodriguez-Nava G, Trelles-Garcia DP, Yanez-Bello MA, Imani-Ramos T, Trelles-Garcia VP, Bustamante-Soliz DS, Patiño-Salamea E. MMR Vaccine Adverse Drug Reactions Reports in the CDC WONDER System, 1989–2019. InOpen Forum Infectious Diseases 2020 Aug (Vol. 7, No. 8, p. ofaa211). US: Oxford University Press.
292. Qian M, Chou SY, Lai EK. Confirmatory bias in health decisions: Evidence from the MMR-autism controversy. Journal of health economics. 2020 Mar 1;70:102284.
293. Parker AA, Staggs W, Dayan GH, Ortega-Sánchez IR, Rota PA, Lowe L, Boardman P, Teclaw R, Graves C, LeBaron CW. Implications of a 2005 measles outbreak in Indiana for sustained elimination of measles in the United States. New England Journal of Medicine. 2006 Aug 3;355(5):447-55.
294. Dempsey AF, Schaffer S, Singer D, Butchart A, Davis M, Freed GL. Alternative vaccination schedule preferences among parents of young children. Pediatrics. 2011 Nov 1;128(5):848-56.
295. Doll MK, Weitzen SD, Morrison KT. Trends in the uptake of pediatric measles-containing vaccine in the United States: A Disneyland effect?. Vaccine. 2021 Jan 8;39(2):357-63.
296. Downs JS, de Bruin WB, Fischhoff B. Parents' vaccination comprehension and decisions. InRisk Analysis and Human Behavior 2013 Jun 17 (pp. 274-297). Routledge.

297. Cataldi JR, Dempsey AF, O'Leary ST. Measles, the media, and MMR: impact of the 2014–15 measles outbreak. Vaccine. 2016 Dec 7;34(50):6375-80.
298. Lieu TA, Ray GT, Klein NP, Chung C, Kulldorff M. Geographic clusters in underimmunization and vaccine refusal. Pediatrics. 2015 Feb 1;135(2):280-9.
299. Mills K, Nilsen K. Kansas family physicians perceptions of parental vaccination hesitancy. Kansas journal of medicine. 2020;13:248.
300. McNutt LA, Desemone C, DeNicola E, El Chebib H, Nadeau JA, Bednarczyk RA, Shaw J. Affluence as a predictor of vaccine refusal and underimmunization in California private kindergartens. Vaccine. 2016 Mar 29;34(14):1733-8.
301. Gennaro E, Caleb S, Torres R, Alexander-Parrish R, Thoburn E, McLaughlin JM, Fu LY. Parental Beliefs, Logistical Challenges, and Improvement Opportunities for Vaccination among Children Ages 19-35 Months Experiencing Homelessness. The Journal of Pediatrics. 2021 Sep 1;236:246-52.
302. Langkamp DL, Dusseau A, Brown MF. Vaccine hesitancy and low immunization rates in children with down syndrome. The Journal of pediatrics. 2020 Aug 1;223:64-7.
303. Opel DJ, Taylor JA, Zhou C, Catz S, Myaing M, Mangione-Smith R. The relationship between parent attitudes about childhood vaccines survey scores and future child immunization status: a validation study. JAMA pediatrics. 2013 Nov 1;167(11):1065-71.
304. Nyhan B, Reifler J, Richey S, Freed GL. Effective messages in vaccine promotion: a randomized trial. Pediatrics. 2014 Apr 1;133(4):e835-42.
305. Opel DJ, Taylor JA, Mangione-Smith R, Solomon C, Zhao C, Catz S, Martin D. Validity and reliability of a survey to identify vaccine-hesitant parents. Vaccine. 2013 Sep 2;29(38):6598-605.
306. Nyathi S, Karpel HC, Sainani KL, Maldonado Y, Hotez PJ, Bendavid E, Lo NC. The 2016 California policy to eliminate nonmedical vaccine exemptions and changes in vaccine coverage: An empirical policy analysis. PLoS medicine. 2019 Dec 23;16(12):e1002994.
307. Newcomer SR, Freeman RE, Wehner BK, Anderson SL, Daley MF. Timeliness of early childhood vaccinations and undervaccination patterns in Montana. American journal of preventive medicine. 2021 Jul 1;61(1):e21-9.
308. Christianson B, Sharif-Mohamed F, Heath J, Roddy M, Bahta L, Omar H, Rockwood T, Kenyon C. Parental attitudes and decisions regarding MMR vaccination during an outbreak of measles among an undervaccinated Somali community in Minnesota. Vaccine. 2020 Oct 21;38(45):6979-84.
309. Estep K, Greenberg P. Opting out: individualism and vaccine refusal in pockets of socioeconomic homogeneity. American Sociological Review. 2020 Dec;85(6):957-91.
310. Moyer-Gusé E, Robinson MJ, Mcknight J. The role of humor in messaging about the MMR vaccine. Journal of Health Communication. 2018 Jun 3;23(6):514-22.
311. Frew PM, Fisher AK, Basket MM, Chung Y, Schamel J, Weiner JL, Mullen J, Omer SB, Orenstein WA. Changes in childhood immunization decisions in the United States: Results from 2012 & 2014 National Parental Surveys. Vaccine. 2016 Nov 4;34(46):5689-96.

312. Fuchs EL. Self-reported prenatal influenza vaccination and early childhood vaccine series completion. Preventive medicine. 2016 Jul 1;88:8-12.
313. Kennedy AM, Gust DA. Measles outbreak associated with a church congregation: a study of immunization attitudes of congregation members. Public Health Reports. 2008 Mar;123(2):126-34.
314. Kang GJ, Ewing-Nelson SR, Mackey L, Schlitt JT, Marathe A, Abbas KM, Swarup S. Semantic network analysis of vaccine sentiment in online social media. Vaccine. 2017 Jun 22;35(29):3621-38.
315. Holroyd TA, Howa AC, Delamater PL, Klein NP, Buttenheim AM, Limaye RJ, Proveaux TM, Omer SB, Salmon DA. Parental vaccine attitudes, beliefs, and practices: initial evidence in California after a vaccine policy change. Human Vaccines & Immunotherapeutics. 2021 Jun 3;17(6):1675-80.
316. Gromis A, Liu KY. The emergence of spatial clustering in medical vaccine exemptions following California Senate Bill 277, 2015–2018. American Journal of Public Health. 2020 Jul;110(7):1084-91.
317. Gahr P, DeVries AS, Wallace G, Miller C, Kenyon C, Sweet K, Martin K, White K, Bagstad E, Hooker C, Krawczynski G. An outbreak of measles in an undervaccinated community. Pediatrics. 2014 Jul 1;134(1):e220-8.
318. Glanz JM, Wagner NM, Narwaney KJ, Pyrzanowski J, Kwan BM, Sevick C, Resnicow K, Dempsey AF. Web-based tailored messaging to increase vaccination: A randomized clinical trial. Pediatrics. 2020 Nov 1;146(5).
319. Cole JW, Chen AM, McGuire K, Berman S, Gardner J, Teegala Y. Motivational interviewing and vaccine acceptance in children: The MOTIVE study. Vaccine. 2022 Mar 15;40(12):1846-54.
320. Sugerman DE, Barskey AE, Delea MG, Ortega-Sanchez IR, Bi D, Ralston KJ, Rota PA, Waters-Montijo K, LeBaron CW. Measles outbreak in a highly vaccinated population, San Diego, 2008: role of the intentionally undervaccinated. Pediatrics. 2010 Apr 1;125(4):747-55.
321. Duchsherer A, Jason M, Platt CA, Majdik ZP. Immunized against science: Narrative community building among vaccine refusing/hesitant parents. Public Understanding of Science. 2020 May;29(4):419-35.
322. Davis MM, Zickafoose JS, Halvorson AE, Patrick SW. Parents' likelihood to vaccinate their children and themselves against COVID-19. MedRxiv. 2020 Nov 13:2020-11.
323. Degarege A, Krupp K, Fennie K, Li T, Stephens DP, Marlow LA, Srinivas V, Arun A, Madhivanan P. Urban-rural inequities in the parental attitudes and beliefs towards human papillomavirus infection, cervical cancer, and human papillomavirus vaccine in Mysore, India. Journal of pediatric and adolescent gynecology. 2018 Oct 1;31(5):494-502.
324. Abdalla SM, Ahmad MS, Al-Baradie NS, Alshuwaish LM, Al-Issa RA, Alrashidi SS. Assessment of parent knowledge and perception towards the importance of child immunization in Sudair region, Saudi Arabia. European Review for Medical and Pharmacological Sciences. 2022 Mar 1;26(6):1803-8.
325. Amit Aharon A, Nehama H, Rishpon S, Baron-Epel O. A path analysis model suggesting the association between health locus of control and compliance with

childhood vaccinations. Human vaccines & immunotherapeutics. 2018 Jul 3;14(7):1618-25.
326. Aharony N, Goldman R. E-health literacy and the vaccination dilemma: an Israeli perspective. 2017
327. Akhmetzhanova Z, Sazonov V, Riethmacher D, Aljofan M. Vaccine adherence: the rate of hesitancy toward childhood immunization in Kazakhstan. Expert review of vaccines. 2020 Jun 2;19(6):579-84.
328. Akıs S, Velıpasaoglu S, Camurdan AD, Beyazova U, Sahın F. Factors associated with parental acceptance and refusal of pandemic influenza A/H1N1 vaccine in Turkey. European journal of pediatrics. 2011 Sep;170(9):1165-72.
329. Alabbad AA, Alsaad AK, Al Shaalan MA, Alola S, Albanyan EA. Prevalence of influenza vaccine hesitancy at a tertiary care hospital in Riyadh, Saudi Arabia. Journal of infection and public health. 2018 Jul 1;11(4):491-9.
330. AlGoraini YM, AlDujayn NN, AlRasheed MA, Bashawri YE, Alsubaie SS, AlShahrani DA. Confidence toward vaccination as reported by parents of children admitted to a tertiary care hospital in Riyadh, Saudi Arabia: A cross sectional study. Vacunas (English Edition). 2020 Jul 1;21(2):95-104.
331. Alhazza SF, Altalhi AM, Alamri KM, Alenazi SS, Alqarni BA, Almohaya AM. Parents' hesitancy to vaccinate their children against COVID-19, a country-wide survey. Frontiers in Public Health. 2022;10.
332. Ali HYM. Hepatitis B infection among Iraqi children: the impact of sanctions. EMHJ - Eastern Mediterranean Health Journal, 10 (1-2), 6-11, 2004 [Internet] 2004; Available from: https://apps.who.int/iris/handle/10665/119374
333. Ali M, Ahmed S, Bonna AS, Sarkar AS, Islam MA, Urmi TA, Proma TS. Parental coronavirus disease vaccine hesitancy for children in Bangladesh: a cross-sectional study. F1000Research. 2022;11.
334. Ali M, Proma TS, Tasnim Z, Islam MA, Urmi TA, Ahmed S, Sarkar AS, Bonna AS, Khan US. Parental COVID-19 vaccine hesitancy for children with neurodevelopmental disorders: a cross-sectional survey. Tropical Medicine and Health. 2022 Mar 21;50(1):24.
335. Ali-Saleh O, Bord S, Basis F. Factors associated with decisions of Arab minority parents in Israel to vaccinate their children against COVID-19. Vaccines. 2022 May 29;10(6):870.
336. Almalki OS, Alfayez OM, Al Yami MS, Asiri YA, Almohammed OA. Parents' hesitancy to vaccinate their 5–11-year-old children against COVID-19 in Saudi Arabia: predictors from the health belief model. Frontiers in public health. 2022:728.
337. Almusbah Z, Alhajji Z, Alshayeb Z, Alhabdan R, Alghafli S, Almusabah M, Almuqarrab F, Aljazeeri I, Almuhawas F. Caregivers' willingness to vaccinate their children against COVID-19 in Saudi Arabia: a cross-sectional survey. Cureus. 2021 Aug 17;13(8).
338. Alolayan A, Almotairi B, Alshammari S, Alhearri M, Alsuhaibani M. Seasonal influenza vaccination among Saudi children: Parental barriers and willingness to vaccinate their children. International journal of environmental research and public health. 2019 Nov;16(21):4226.
339. Alsubaie SS, Gosadi IM, Alsaadi BM, Albacker NB, Bawazir MA, Bin-Daud N, Almanie WB, Alsaadi MM, Alzamil FA. Vaccine hesitancy among Saudi parents

and its determinants: Result from the WHO SAGE working group on vaccine hesitancy survey tool. Saudi medical journal. 2019 Dec;40(12):1242.
340. Alsuwaidi AR, Elbarazi I, Al-Hamad S, Aldhaheri R, Sheek-Hussein M, Narchi H. Vaccine hesitancy and its determinants among Arab parents: a cross-sectional survey in the United Arab Emirates. Human vaccines & immunotherapeutics. 2020 Dec 1;16(12):3163-9.
341. Altulaihi BA, Alaboodi T, Alharbi KG, Alajmi MS, Alkanhal H, Alshehri A. Perception of parents towards COVID-19 vaccine for children in Saudi population. Cureus. 2021 Sep 28;13(9).
342. Ashkenazi S, Livni G, Klein A, Kremer N, Havlin A, Berkowitz O. The relationship between parental source of information and knowledge about measles/measles vaccine and vaccine hesitancy. Vaccine. 2020 Oct 27;38(46):7292-8.
343. Atad E, Netzer I, Peleg O, Landsman K, Dalyot K, Reuven SE, Baram-Tsabari A. Vaccine-hesitant parents' considerations regarding Covid-19 vaccination of adolescents. medRxiv. 2021 May 27:2021-05.
344. Buyuktiryaki B, Soyer OU, Erkocoglu M, Dogan A, Azkur D, Kocabas CN, Dallar Y, Tuncer A, Sekerel BE. What a pandemic teaches us about vaccination attitudes of parents of children with asthma. Vaccine. 2014 Apr 25;32(20):2275-80.
345. Çağ Y. Parental attitudes toward vaccination in Turkey: a face-to-face survey. Journal of Pediatric Infectious Diseases. 2020 Jul;15(04):184-8.
346. Chan HK, Soelar SA, Md Ali SM, Ahmad F, Abu Hassan MR. Trends in vaccination refusal in children under 2 years of age in Kedah, Malaysia: a 4-year review from 2013 to 2016. Asia Pacific Journal of Public Health. 2018 Mar;30(2):137-46.
347. Chang K, Lee SY. Why do some Korean parents hesitate to vaccinate their children?. Epidemiology and health. 2019;41.
348. Abdullah AC, NA M, Rosliza AM. Predictors for inadequate knowledge and negative attitude towards childhood immunization among parents in Hulu Langat, Selangor, Malaysia. Malaysian J Public Heal Med. 2018;18(1):102-2.
349. Chia MY, Komar J, Chua TB, Tay LY. Associations between Parent Attitudes and on-and off-Screen Behaviours of Preschool Children in Singapore. International Journal of Environmental Research and Public Health. 2022 Sep 13;19(18):11508.
350. Choi UI, Pang Y, Zheng Y, Tang PK, Hu H, Ung CO. Parents' intention for their children to receive COVID-19 vaccine: Implications for vaccination program in Macao. Frontiers in Pediatrics. 2022;10.
351. Choi J, Kim S, Lee SJ, Bae S, Kim S. Human papillomavirus (HPV) vaccination intent among mothers of adolescent sons: a national survey on HPV knowledge, attitudes and beliefs in South Korea. The World Journal of Men's Health. 2021 Dec 27;40.
352. Dasgupta P, Bhattacherjee S, Mukherjee A, Dasgupta S. Vaccine hesitancy for childhood vaccinations in slum areas of Siliguri, India. Indian journal of public health. 2018 Oct 1;62(4):253.
353. Du M, Tao L, Liu J. Association between risk perception and influenza vaccine hesitancy for children among reproductive women in China during the COVID-19 pandemic: a national online survey. BMC Public Health. 2022 Dec;22(1):1-0.

354. Duong AH, Duong GH, Pham HT. The Willingness of Parents to Vaccinate Their Children Aged from Five to under Twelve Years with COVID-19 Vaccines between February and March 2022 in Vietnam. Vaccines. 2022 Oct 22;10(11):1775.
355. Azizi FS, Kew Y, Moy FM. Vaccine hesitancy among parents in a multi-ethnic country, Malaysia. Vaccine. 2017 May
356. Fan J, Ye C, Wang Y, Qi H, Li D, Mao J, Xu H, Shi X, Zhu W, Zhou Y. Parental Seasonal Influenza Vaccine Hesitancy and Associated Factors in Shanghai, China, during the COVID-19 Pandemic: A Cross-Sectional Study. Vaccines. 2022 Dec;10(12):2109.
357. Frianto D, Setiawan D, Diantini A, Suwantika AA. Parental Acceptance of Human Papillomavirus (HPV) Vaccination in Districts with High Prevalence of Cervical Cancer in West Java, Indonesia. Patient preference and adherence. 2022 Jan 1:2709-20.
358. Gesser-Edelsburg A, Shir-Raz Y, Green MS. Why do parents who usually vaccinate their children hesitate or refuse? General good vs. individual risk. Journal of Risk Research. 2016 Apr 20;19(4):405-24.
359. Gunes NA. Parents' perspectives about vaccine Hesitancies and vaccine rejection, in the West of turkey. Journal of Pediatric Nursing. 2020 Jul 1;53:e186-94.
360. Günes Ö, Gülhan B, Guney AY, Üçkardeş F, Ozen S, Guder L, Mustafaoglu O, Bayraktar P, Yahşi A, Erat T, Kanik-Yuksek S. Do parents vaccinated against COVID-19 protect their children from hospitalization due to COVID-19?. Journal of Tropical Pediatrics. 2023 Feb;69(1):fmac105.
361. Han Y, Wang Q, Zhao S, Wang J, Dong S, Cui T, Liu M, Shi N, Yang L, Han Y, Xiu S. Parental category B vaccine hesitancy and associated factors in China: an online cross-sectional survey. Expert Review of Vaccines. 2022 Jan 2;21(1):145-53.
362. Han K, Hou Z, Tu S, Wang Q, Hu S, Xing Y, Du J, Zang S, Chantler T, Larson H. Childhood Influenza Vaccination and Its Determinants during 2020–2021 Flu Seasons in China: A Cross-Sectional Survey. Vaccines. 2022 Nov 23;10(12):1994.
363. Hou Z, Song K, Wang Q, Zang S, Tu S, Chantler T, Larson HJ. Childhood COVID-19 vaccine acceptance and preference from caregivers and healthcare workers in China: A survey experiment. Preventive Medicine. 2022 Aug 1;161:107138.
364. Hu Y, Chen Y, Wang Y, Liang H. Measuring childhood vaccination acceptance of mother in Zhejiang province, East China. Human vaccines & immunotherapeutics. 2019 Feb 1;15(2):287-94.
365. Huang LL, Tung TH, Jiang YH, Hu WW, Yang YP. Determinants of the willingness of medical staff to vaccinate their children with a booster dose of the COVID-19 vaccine in Taizhou, China. Human Vaccines & Immunotherapeutics. 2022 Nov 30;18(6):2139098.
366. Hussein SZ, Mardia N, Amirah M, Hashim R, Bakar SH. Knowledge and Practice of Parents Towards Measles, Mumps and Rubella Vaccination. The Malaysian Journal of Medical Sciences: MJMS. 2022 Jun;29(3):90.
367. Jalloh MF, Bennett SD, Alam D, Kouta P, Lourenço D, Alamgir M, Feldstein LR, Ehlman DC, Abad N, Kapil N, Vandenent M. Rapid behavioral assessment of barriers and opportunities to improve vaccination coverage among displaced Rohingyas in Bangladesh, January 2018. Vaccine. 2019 Feb 4;37(6):833-8.

368. Ji M, Huang Z, Ren J, Wagner AL. Vaccine hesitancy and receipt of mandatory and optional pediatric vaccines in Shanghai, China. Human Vaccines & Immunotherapeutics. 2022 Nov 30;18(5):2043025.
369. Jiang M, Gong Y, Fang Y, Yao X, Feng L, Zhu S, Peng J, Shi X. Parental Preferences of Influenza Vaccination for Children in China: A National Survey with a Discrete Choice Experiment. International Journal of Environmental Research and Public Health. 2022 Feb 14;19(4):2145.
370. Kalok A, Loh SY, Chew KT, Aziz NH, Shah SA, Ahmad S, Ismail NA, Mahdy ZA. Vaccine hesitancy towards childhood immunisation amongst urban pregnant mothers in Malaysia. Vaccine. 2020 Feb 24;38(9):2183-9.
371. Khaliq A, Sayed SA, Hussaini SA, Azam K, Qamar M. Missed immunization opportunities among children under 5 years of age dwelling In Karachi city. Journal of Ayub Medical College, Abbottabad: JAMC. 2017;29(4):645.
372. Khaliq A, Elahi AA, Zahid A, Lassi ZS. A survey exploring reasons behind immunization refusal among the parents and caregivers of children under two years living in urban slums of Karachi, Pakistan. International Journal of Environmental Research and Public Health. 2022 Sep 15;19(18):11631.
373. Khan TM, Sahibzada MU. Challenges to health workers and their opinions about parents' refusal of oral polio vaccination in the Khyber Pakhtoon Khawa (KPK) province, Pakistan. Vaccine. 2016 Apr 19;34(18):2074-81.
374. Khan YH, Mallhi TH, Salman M, Tanveer N, Butt MH, Mustafa ZU, Aftab RA, Alanazi AS. Parental Perceptions and Barriers towards Childhood COVID-19 Vaccination in Saudi Arabia: A Cross-Sectional Analysis. Vaccines. 2022 Dec 7;10(12):2093.
375. Khowaja AR, Khan SA, Nizam N, Omer SB, Zaidi A. Parental perceptions surrounding polio and self-reported non-participation in polio supplementary immunization activities in Karachi, Pakistan: a mixed methods study. Bulletin of the World Health Organization. 2012;90:822-30.
376. Kitro A, Sirikul W, Dilokkhamaruk E, Sumitmoh G, Pasirayut S, Wongcharoen A, Panumasvivat J, Ongprasert K, Sapbamrer R. COVID-19 vaccine hesitancy and influential factors among Thai parents and guardians to vaccinate their children. Vaccine: X. 2022 Aug 1;11:100182.
377. Kuan CI. Vaccine hesitancy and emerging parental norms: A qualitative study in Taiwan. Sociology of Health & Illness. 2022 Mar;44(3):692-709.
378. Lai X, Li M, Hou Z, Guo J, Zhang H, Wang J, Fang H. Factors associated with caregivers' hesitancy to vaccinate children against influenza: A cross-sectional survey in China. Vaccine. 2022 Jun 23;40(29):3975-83.
379. Lee M, Seo S, Choi S, Park JH, Kim S, Choe YJ, Choi EH, Kwon GY, Shin JY, Choi SY, Jeong MJ. Parental acceptance of COVID-19 vaccination for children and its association with information sufficiency and credibility in South Korea. JAMA Network Open. 2022 Dec 1;5(12):e2246624-.
380. Li JB, Lau EY, Chan DK. Why do Hong Kong parents have low intention to vaccinate their children against COVID-19? Testing health belief model and theory of planned behavior in a large-scale survey. Vaccine. 2022 Apr 26;40(19):2772-80.
381. Li L, Ma Y, Li W, Tang G, Jiang Y, Li H, Jiang S, Zhou Y, Yang Y, Zhang T, Yang W. Caregiver Willingness to Vaccinate Children with Pneumococcal Vaccines

and to Pay in a Low-Resource Setting in China: A Cross-Sectional Study. Vaccines. 2022 Nov 10;10(11):1897.
382. Liao Q, Fielding R, Cheung DY, Lian J, Lam WW. WhatsApp groups to promote childhood seasonal influenza vaccination: a randomised control trial (abridged secondary publication). Hong Kong Medical Journal. 2022 Feb.
383. Low JM, Soo CW, Phuong TA, Zhong Y. Predicting vaccine hesitancy among parents towards COVID-19 vaccination for their children in Singapore. Frontiers in pediatrics. 2022;10.
384. Lu L, Gu W, Xie H, Wang X, Cao L, Shan M, Wu P, Tian Y, Zhou K. Parental Attitudes Towards Vaccination Against COVID-19 in China During Pandemic. Infection and Drug Resistance. 2022 Jan 1:4541-6.
385. Ma L, Yang J, Zhang T, Han X, Huang Q, Yang Y, Feng L, Yang W, Wang C. Willingness toward COVID-19 vaccination, coadministration with other vaccines and receive a COVID-19 vaccine booster: A cross-sectional study on the guardians of children in China. Human vaccines & immunotherapeutics. 2022 Nov 30;18(5):2049169.
386. Ma Y, Liu N, Zhong G, Wang D, Cao L, Bai S, Zhu P, Zhang A, Wang X. Parent acceptance toward inactivated COVID-19 vaccination in children with acute lymphoblastic leukemia: The power of oncologist and alliance. Vaccines. 2022 Dec;10(12):2016.
387. Mishra K, Mohapatra I, Mohapatra PS, Madhusikta S, Parimita P. Challenges and barriers to immunization during COVID-19: An experience of parents/caregivers from a well-baby clinic of a tertiary care hospital of Eastern India. Clinical Epidemiology and Global Health. 2023 Jan 1;19:101200.
388. Mohan R, Pandey V, Kumar A, Gangadevi P, Goel AD, Joseph J, Kurien N. Acceptance and Attitude of Parents Regarding COVID-19 Vaccine for Children: A Cross-Sectional Study. Cureus. 2022 Apr 27;14(4).
389. Muhsen K, El-Hai RA, Amit-Aharon A, Nehama H, Gondia M, Davidovitch N, Goren S, Cohen D. Risk factors of underutilization of childhood immunizations in ultraorthodox Jewish communities in Israel despite high access to health care services. Vaccine. 2012 Mar 9;30(12):2109-15.
390. Ng DL, Gan GG, Chai CS, Anuar NA, Sindeh W, Chua WJ, Said AB, Tan SB. The willingness of parents to vaccinate their children younger than 12 years against COVID-19: a cross-sectional study in Malaysia. BMC Public Health. 2022 Dec;22(1):1-3.
391. Noyman-Veksler G, Greenberg D, Grotto I, Shahar G. Parents’ malevolent personification of mass vaccination solidifies vaccine hesitancy. Journal of Health Psychology. 2021 Oct;26(12):2164-72.
392. Padhi BK, Satapathy P, Rajagopal V, Rustagi N, Vij J, Jain L, Chakrapani V, Patro BK, Kar SS, Singh R, Pala S. Parents' Perceptions and Intention to Vaccinate Their Children Against COVID-19: Results From a Cross-Sectional National Survey in India. Frontiers in medicine. 2022;9.
393. Parinyarux P, Sunkonkit K, Yotsombut K. Parental COVID-19 vaccination hesitancy among parents of children aged 5–18 years in Thailand: a cross-sectional survey study. Journal of Pharmaceutical Policy and Practice. 2022 Dec;15(1):1-9.

394. Pearl CA, Navaneetha M, Malarvilizhi S, Mony K. Online educational program for parents of adolescents on human papillomavirus vaccination during COVID pandemic: A feasibility check. Indian Journal of Public Health. 2022 Apr 1;66(2):104.
395. Qi L, Su K, Xia Y, Tang W, Shen T, Li Q. Enterovirus 71 vaccine acceptance among parents of children< 5 years old and their knowledge of hand, foot and mouth disease, Chongqing, China, 2017. PLoS One. 2019 Nov 27;14(11):e0225569.
396. Qin C, Wang R, Tao L, Liu M, Liu J. Association between risk perception and acceptance for a booster dose of COVID-19 vaccine to children among child caregivers in China. Frontiers in public health. 2022 Mar 16;10:834572.
397. Quaiyum MA, Gazi R, Khan AI, Uddin J, Islam M, Ahmed F, Saha NC. Programmatic aspects of dropouts in child vaccination in Bangladesh: findings from a prospective study. Asia Pacific Journal of Public Health. 2011 Mar;23(2):141-50.
398. Rehman T, Mallick A, Ahamed F, Kanungo S, Pati S. Willingness to pay for a COVID-19 vaccine for oneself and one's child among individuals attending a tertiary care centre in West Bengal, India. Nigerian Postgraduate Medical Journal. 2022 Oct 1;29(4):296.
399. Ren J, Wagner AL, Zheng A, Sun X, Boulton ML, Huang Z, Zikmund-Fisher BJ. The demographics of vaccine hesitancy in Shanghai, China. PLoS One. 2018 Dec 13;13(12):e0209117.
400. Samudyatha UC, Balaji B, Singh M, Gowda M. Caregivers' Preferences of COVID-19 Vaccination for Children: A Cross-sectional Study From Rural South India. Medeniyet Medical Journal. 2022 Sep;37(3):248.
401. Choi SH, Jo YH, Jo KJ, Park SE. Pediatric and parents' attitudes towards COVID-19 vaccines and intention to vaccinate for children. Journal of Korean medical science. 2021 Aug 9;36(31).
402. Shahani R, Chu J, Rufai OH, Zawar A, Muhideen S, Dilawar S, Amosun TS. Understanding the Role of Psychosocial Factors in Pakistani Parents' Hesitancy to Vaccinate Their Kids: The Mediating Role of Knowledge and Mistrust of Science about the COVID-19 Vaccine. Vaccines. 2022 Aug 5;10(8):1260.
403. Shaipuzaman NA, Rahman HA. Knowledge and attitude on infant vaccination among university staff in Malaysian public university. Human Vaccines & Immunotherapeutics. 2022 Jan 31;18(1):2029258.
404. Shen X, Wu X, Deng Z, Liu X, Zhu Y, Huang Y, Deng Y, Tian Q, Gan Y, Gong Y, Lu Z. Analysis on vaccine hesitation and its associated factors among parents of preschool children in Songgang Street, Shenzhen. Scientific Reports. 2022 Jun 8;12(1):9467.
405. Shwethashree M, Vanmathi A, Narayanamurthy MR, Gopi A. Did this pandemic trigger a spike in mothers′ hesitancy over their children's routine immunizations?-A cross sectional study. Clinical Epidemiology and Global Health. 2022 May 1;15:101023.
406. Sinuraya RK, Kusuma AS, Pardoel ZE, Postma MJ, Suwantika AA. Parents' Knowledge, Attitude, and Practice on Childhood Vaccination During the COVID-19 Pandemic in Indonesia. Patient preference and adherence. 2022 Jan 14:105-12.
407. Summan A, Nandi A, Shet A, Laxminarayan R. The effect of the COVID-19 pandemic on routine childhood immunization coverage and timeliness in India:

Retrospective analysis of the National Family Health Survey of 2019–2021 data. The Lancet Regional Health-Southeast Asia. 2023 Jan 1;8:100099.
408. Sun X, Huang Z, Wagner AL, Prosser LA, Xu E, Ren J, Wang B, Yan W, Zikmund-Fisher BJ. The role of severity perceptions and beliefs in natural infections in Shanghai parents' vaccine decision-making: a qualitative study. BMC public health. 2018 Dec;18(1):1-9.
409. Tavakoli N, Nafissi N, Shokri S, Fallahpour M, Soleimani S, Riahi T, Kalantari S, Javan A, Goodarzi A, Valizadeh R. Pediatric and adolescent COVID-19 vaccination side effects: A retrospective cohort study of the Iranian teenage group in 2021. Journal of medical virology. 2022 Oct;94(10):4890-900.
410. Tang S, Liu X, Jia Y, Chen H, Zheng P, Fu H, Xiao Q. Education level modifies parental hesitancy about COVID-19 vaccinations for their children. Vaccine. 2023 Jan 9;41(2):496-503.
411. Noor T, Zubair A, Bhatti MA. Causes of Vaccine Hesitancy among the parents: A Survey in Suburban Area of Lahore. Pakistan Journal Of Medical & Health Sciences. 2018 Jul 1;12(3):963-4.
412. Temsah MH, Alhuzaimi AN, Aljamaan F, Bahkali F, Al-Eyadhy A, Alrabiaah A, Alhaboob A, Bashiri FA, Alshaer A, Temsah O, Bassrawi R. Parental attitudes and hesitancy about COVID-19 vs. routine childhood vaccinations: a national survey. Frontiers in public health. 2021 Oct 13;9:752323.
413. Tianshuo Z, Hanyu L, Bingfeng H, Bei L, Jiang L, Juan D, Ninghua H, Qingbin L, Yaqiong L, Fuqiang C. Evaluation of the reliability and validity of a vaccine hesitancy scale on knowledge, attitude, trust and vaccination environment (KATE-S) in Chinese parents. Vaccine. 2022 May 9;40(21):2933-9.
414. Tsang TK, Wang C, Fang VJ, Perera RA, So HC, Ip DK, Peiris JM, Leung GM, Cauchemez S, Cowling BJ. Indirect Protection from Vaccinating Children against Influenza A Virus Infection in Households. Viruses. 2022 Sep 21;14(10):2097.
415. Tubaş F, Dulkadir R, Taplak AŞ, Ünlü E. Knowledge and Attitudes of Physicians and Nurses in Turkey Regarding Human Papillomavirus Vaccination of Their Children. Journal of Community Health. 2022 Oct 28:1-5.
416. Tung TH, Lin XQ, Chen Y, Wu H, Zhang MX, Zhu JS. Why do parents willingness-to-pay to vaccinate their children against COVID-19? A real-world evidence in Taizhou, China. Human Vaccines & Immunotherapeutics. 2022 Jan 31;18(1):1-9.
417. Wachinger J, Reñosa MD, Endoma V, Aligato MF, Landicho-Guevarra J, Landicho J, Bravo TA, McMahon SA. Bargaining and gendered authority: a framework to understand household decision-making about childhood vaccines in the Philippines. BMJ global health. 2022 Sep 1;7(9):e009781.
418. Wan X, Huang H, Shang J, Xie Z, Jia R, Lu G, Chen C. Willingness and influential factors of parents of 3-6-year-old children to vaccinate their children with the COVID-19 vaccine in China. Human Vaccines & Immunotherapeutics. 2021 Nov 2;17(11):3969-74.
419. Wang K, Wong EL, Cheung AW, Chung VC, Wong CH, Dong D, Wong SY, Yeoh EK. Impact of information framing and vaccination characteristics on parental COVID-19 vaccine acceptance for children: A discrete choice experiment. European Journal of Pediatrics. 2022 Nov;181(11):3839-49.

420. Wang L, Wen W, Chen C, Tang J, Wang C, Zhou M, Cheng Y, Zhang X, Wang M, Feng Z, Wang W. Explore the attitudes of children and adolescent parents towards the vaccination of COVID-19 in China. Italian Journal of Pediatrics. 2022 Jul 23;48(1):122.
421. Wang LJ, Kou KC, Tang KS, Lee Y, Chen YC, Lo MH, Lee IK, Chuah SK, Lee CT, Kung CT, Wang CC. Parental Attitudes, Intentions, Decisions, and Psychological Wellbeing Regarding COVID-19 Vaccination: Preschool, School-Age, and Adolescent Caregivers. Vaccines. 2022 Dec;10(12):2114
422. Wang M, Li M, Li X, Chen X, Jiang F, Wang Z, Zhang L, Lu Y, Peng W, Wang W, Fu C. Intention and Attitude to Accept a Pertussis Cocooning Vaccination among Chinese Children's Guardians: A Cross-Sectional Survey. International Journal of Environmental Research and Public Health. 2022 Jan;19(23):16282.
423. Wang Q, Xiu S, Zhao S, Wang J, Han Y, Dong S, Huang J, Cui T, Yang L, Shi N, Liu M. Vaccine hesitancy: COVID-19 and influenza vaccine willingness among parents in Wuxi, China—a cross-sectional study. Vaccines. 2021 Apr 1;9(4):342.
424. Wang Q, Xiu S, Yang L, Han Y, Cui T, Shi N, Liu M, Yi Y, Liu C, Wang X, Yang G. Changes in parental attitudes toward COVID-19 vaccination and routine childhood vaccination during the COVID-19 pandemic: Repeated cross-sectional survey study. JMIR public health and surveillance. 2022 May 13;8(5):e33235.
425. Wang Q, Xiu S, Yang L, Han Y, Cui T, Shi N, Liu M, Yi Y, Liu C, Wang X, Zhou W. Validation of the World Health Organization's parental vaccine hesitancy scale in China using child vaccination data. Human Vaccines & Immunotherapeutics. 2022 Jan 31;18(1):2021060.
426. Wang X, Feng Y, Zhang Q, Ye L, Cao M, Liu P, Liu S, Li S, Zhang J. Parental preference for Haemophilus influenzae type b vaccination in Zhejiang Province, China: A discrete choice experiment. Frontiers in Public Health. 2022;10.
427. Wong LP, Lee HY, Alias H, AbuBakar S. Malaysian parents' willingness to vaccinate their children against COVID-19 infection and their perception of mRNA COVID-19 vaccines. Vaccines. 2022 Oct 25;10(11):1790.
428. Wong WH, So HK, Rosa Duque JS, Tso WW, Chong PC, Kwan MY, Lau YL. Impact of a focus education in Zoom on COVID-19 vaccine hesitancy in Hong Kong parents of the preschoolers. Human Vaccines & Immunotherapeutics. 2022 Nov 30;18(5):2081460.
429. Wu L, Huang Z, Guo X, Liu J, Sun X. Measuring parents' acceptance of non-national immunization program vaccines for children and its influencing factors during the COVID-19 pandemic in Shanghai, China. Human Vaccines & Immunotherapeutics. 2022 Nov 30;18(5):2069427.
430. Xu Y, Zhang R, Zhou Z, Fan J, Liang J, Cai L, Peng L, Ren F, Lin W. Parental psychological distress and attitudes towards COVID-19 vaccination: a cross-sectional survey in Shenzhen, China. Journal of Affective Disorders. 2021 Sep 1;292:552-8.
431. Yalçin SS, Kömürlüoğlu A, Topaç O. Rates of childhood vaccine refusal in Turkey during 2016–2017: Regional causes and solutions. Archives de Pédiatrie. 2022 Nov 1;29(8):594-8.
432. Yalçin SS, Bakacak AG, Topaç O. Unvaccinated children as community parasites in National Qualitative Study from Turkey. BMC Public Health. 2020 Dec;20:1-7.

433. Yuen WW, Lee A, Chan PK, Tran L, Sayko E. Uptake of human papillomavirus (HPV) vaccination in Hong Kong: Facilitators and barriers among adolescent girls and their parents. PLoS One. 2018 Mar 15;13(3):e0194159.
434. Yang J, Zhang T, Qi W, Zhang X, Jia M, Leng Z, Wang Q, Yang Y, Yang W, Ma L, Feng L. COVID-19 vaccination in Chinese children: a cross-sectional study on the cognition, psychological anxiety state and the willingness toward vaccination. Human Vaccines & Immunotherapeutics. 2022 Jan 31;18(1):1-7.
435. Yang Y, Zhang T, Wang Y, Ma L, Xie S, Zhou J, Qi W, Wang Q, Dai P, Du Y, Feng L. Willingness of caregivers to have their children vaccinated with pneumococcal vaccines in the context of an innovative immunization strategy—Weifang City, Shandong province, 2021. China CDC Weekly. 2022 May 5;4(20):421.
436. Yigit M, Ozkaya-Parlakay A, Senel E. Evaluation of COVID-19 vaccine refusal in parents. The Pediatric infectious disease journal. 2021 Apr 1;40(4):e134-6.
437. Yılmaz M, Sahin MK. Parents' willingness and attitudes concerning the COVID-19 vaccine: a cross-sectional study. International journal of clinical practice. 2021 Sep;75(9):e14364.
438. Yılmazbas P, Terzi O, Ozceker D. Did. Covid-19 Pandemic Changed Parents' Approach To Vaccination. Soc Behav. 2021;43(2):130-4.
439. Yörük S, Güler D. Factors associated with pediatric vaccine hesitancy of parents: a cross-sectional study in Turkey. Human Vaccines & Immunotherapeutics. 2021 Nov 2;17(11):4505-11.
440. Huang Y, Xu S, Xu Y, Yao D, Wang L, Zhao Y, Wu Q. A new strategy for cervical cancer prevention among Chinese women: how much do they know and how do they react toward the HPV immunization?. Journal of Cancer Education. 2021 Apr;36(2):386-94.
441. Zakhour R, Tamim H, Faytrouni F, Khoury J, Makki M, Charafeddine L. Knowledge, attitude and practice of influenza vaccination among Lebanese parents: A cross-sectional survey from a developing country. Plos one. 2021 Oct 14;16(10):e0258258.
442. Zhang MX, Lin XQ, Chen Y, Tung TH, Zhu JS. Determinants of parental hesitancy to vaccinate their children against COVID-19 in China. Expert Review of Vaccines. 2021 Oct 3;20(10):1339-49.
443. Zhang H, Ren X, Tian K, Yu J, Zhu A, Zhang L, Gao GF, Li Z. The Impact and Vaccination Coverage of Seasonal Influenza among Children Aged 6–59 Months in China in 2017–2018: An Internet Panel Survey. Vaccines. 2022 Apr 18;10(4):630.
444. Zhang KC, Fang Y, Cao H, Chen H, Hu T, Chen YQ, Zhou X, Wang Z. Parental acceptability of COVID-19 vaccination for children under the age of 18 years: cross-sectional online survey. JMIR pediatrics and parenting. 2020 Dec 30;3(2):e24827.
445. Zhang Z, Shi J, Zhang X, Guo X, Yu W. Willingness of parents of 9-to-18-year-old females in China to vaccinate their daughters with HPV vaccine. Vaccine. 2023 Jan 4;41(1):130-5.
446. Zheng M, Zhong W, Chen X, Wang N, Liu Y, Zhang Q, Cheng Y, Li W, Yu Q, Zhao X, Yuan L. Factors influencing parents' willingness to vaccinate their preschool children against COVID-19: Results from the mixed-method study in China. Human Vaccines & Immunotherapeutics. 2022 Nov 30;18(6):2090776.

447. Zheng Z, Lu Y, Wang M, Luo Y, Wan P, Zhou T, Feng M, Zhu J, Wu J, Ji H, Song Y. Low COVID-19 vaccine coverage and guardian acceptance among pediatric transplant recipients. Journal of Medical Virology. 2023 Jan;95(1):e28377.
448. Zhou Y, Li GX, Zhao TS, Du J, Zhang WX, Xie MZ, Chen LY, Zeng J, Wang C, Liu B, Liu YQ. Parents' willingness to vaccinate themselves and their children with the booster vaccine against SARS-CoV-2: A cross-sectional study in Puyang city, China. Journal of Medical Virology. 2023 Jan;95(1):e28256.
449. Zhou M, Liu L, Gu SY, Peng XQ, Zhang C, Wu QF, Xu XP, You H. Behavioral intention and its predictors toward COVID-19 booster vaccination among Chinese parents: Applying two behavioral theories. International Journal of Environmental Research and Public Health. 2022 Jun 20;19(12):7520.
450. Zhou X, Wang S, Zhang K, Chen S, Chan PS, Fang Y, Cao H, Chen H, Hu T, Chen Y, Wang Z. Changes in Parents' COVID-19 Vaccine Hesitancy for Children Aged 3–17 Years before and after the Rollout of the National Childhood COVID-19 Vaccination Program in China: Repeated Cross-Sectional Surveys. Vaccines. 2022 Sep 6;10(9):1478.
451. Zhou Y, Zhang J, Wu W, Liang M, Wu QS. Willingness to receive future COVID-19 vaccines following the COVID-19 epidemic in Shanghai, China. BMC Public Health. 2021 Dec;21(1):1-9.
452. Akca G, Akca U. Turkish mothers' knowledge and attitude about HPV vaccine. Journal of Pediatric Nursing. 2022 Nov 1;67:83-7.
453. Huang Z, Ji M, Ren J, Sun X, Boulton ML, Zikmund-Fisher BJ, Wagner AL. Effect of the framing of HPV vaccination on parents' willingness to accept an HPV vaccine. Vaccine. 2022 Feb 7;40(6):897-903.
454. Khatatbeh M, Albalas S, Khatatbeh H, Momani W, Melhem O, Al Omari O, Tarhini Z, A'aqoulah A, Al-Jubouri M, Nashwan AJ, Adwan G. Children's rates of COVID-19 vaccination as reported by parents, vaccine hesitancy, and determinants of COVID-19 vaccine uptake among children: a multi-country study from the Eastern Mediterranean Region. BMC Public Health. 2022 Dec;22(1):1-1.
455. Huang LL, Yang YP, Mao HP, Hu WW, Jiang YH, Jiesisibieke ZL, Tung TH. Parental hesitancy towards vaccinating their children with a booster dose against COVID-19: Real-world evidence from Taizhou, China. Journal of Infection and Public Health. 2022 Sep 1;15(9):1006-12.
456. Musa S, Dergaa I, Abdulmalik MA, Ammar A, Chamari K, Saad HB. BNT162b2 COVID-19 vaccine hesitancy among parents of 4023 young adolescents (12–15 years) in Qatar. Vaccines. 2021 Sep 2;9(9):981.
457. Abed Elhadi Shahbari N, Gesser-Edelsburg A, Davidovitch N, Brammli-Greenberg S, Mesch GS. Risk perceptions regarding inclusion of seasonal influenza vaccinations in the school immunization program in Israel: Arab vs. Jewish mothers. Plos one. 2022 Apr 18;17(4):e0267279.
458. Abuhammad S, Khader Y, Hamaideh S. Attitude of parents toward vaccination against COVID-19 for own children in Jordan: a cross-sectional study. Informatics in Medicine Unlocked. 2022 Jan 1;31:101000.
459. Aedh AI. Parents' attitudes, their acceptance of the COVID-19 vaccines for children and the contributing factors in Najran, Saudi Arabia: a cross-sectional survey. Vaccines. 2022 Aug 6;10(8):1264.

460. Akbulut S, Gokce A, Boz G, Saritas H, Unsal S, Ozer A, Akbulut MS, Colak C. Evaluation of Vaccine Hesitancy and Anxiety Levels among Hospital Cleaning Staff and Caregivers during COVID-19 Pandemic. Vaccines. 2022 Aug 30;10(9):1426.
461. Aldakhil H, Albedah N, Alturaiki N, Alajlan R, Abusalih H. Vaccine hesitancy towards childhood immunizations as a predictor of mothers' intention to vaccinate their children against COVID-19 in Saudi Arabia. Journal of infection and public health. 2021 Oct 1;14(10):1497-504.
462. Alenazi KA. Parents' knowledge, attitude and practice towards seasonal influenza vaccination in Riyadh region, Saudi Arabia. The Journal of Infection in Developing Countries. 2022 Oct 31;16(10):1623-9.
463. Alghamdi S. The Attitude of Parents Toward Their Children Receiving the COVID-19 Vaccine. Children. 2022 Aug 28;9(9):1308.
464. Al-Iede M, Foudeh J, Al-Shweiki O, Alshrouf MA, Al-Abdallat T, Aleidi SM, Alqutawneh B. Parents' Willingness to Vaccinate Their Children Against COVID-19: A Cross-Sectional Survey From Jordan. Asia Pacific Journal of Public Health. 2022 Sep;34(6-7):698-701.
465. AlKetbi LM, Al Hosani F, Al Memari S, Al Mazrouei S, Al Shehhi B, AlShamsi N, AlKwuiti MM, Saleheen HN, Al Mutairi H, Al Hajeri OM. Parents' views on the acceptability of a COVID-19 vaccine for their children: A cross-sectional study in Abu Dhabi-United Arab Emirates. Vaccine. 2022 Sep 9;40(38):5562-8.
466. Al-Khlaiwi T, Meo SA, Almousa HA, Almebki AA, Albawardy MK, Alshurafa HH, Althunayan MA, Alsayyari MS. National COVID-19 vaccine program and parent's perception to vaccinate their children: a cross-sectional study. Vaccines. 2022 Jan 22;10(2):168.
467. Almansour A, Hussein SM, Felemban SG, Mahamid AW. Acceptance and hesitancy of parents to vaccinate children against coronavirus disease 2019 in Saudi Arabia. Plos one. 2022 Oct 18;17(10):e0276183.
468. AlOmran HI, Al-Dosary AS, AlGhamdi FM, Alshahrani ZM, Altayar NS. Exploring parents' knowledge and attitudes towards the influenza vaccine in a rural community of Saudi Arabia. Journal of Public Health in Africa. 2022 May 5;13(1).
469. Al-Qahtani AM, Mannasaheb BA, Shaikh MA, Alajlan SA, Alayed MS, Shaikh IA, Asdaq SM, Al-Qahtani FS, Ghazwani EY, Al-Qahtani NS, Abbag BF. Parental Willingness for COVID-19 Vaccination among Children Aged 5 to 11 Years in Riyadh City, Saudi Arabia: A Cross-Sectional Study. Vaccines. 2022 Dec;10(12):1979.
470. Al-Qerem W, Al Bawab AQ, Hammad A, Jaber T, Khdair SI, Kalloush H, Ling J, Mosleh R. Parents' attitudes, knowledge and practice towards vaccinating their children against COVID-19: A cross-sectional study. Human Vaccines & Immunotherapeutics. 2022 Nov 30;18(5):2044257.
471. Al-Regaiey KA, Alshamry WS, Alqarni RA, Albarrak MK, Alghoraiby RM, Alkadi DY, Alhakeem LR, Bashir S, Iqbal M. Influence of social media on parents' attitudes towards vaccine administration. Human Vaccines & Immunotherapeutics. 2022 Jan 31;18(1):1872340.
472. Alsulaiman JW, Mazin M, Al-Shatanawi TN, Kheirallah KA, Allouh MZ. Parental willingness to vaccinate their children against SARS-CoV-2 in Jordan: an explanatory cross-sectional study. Risk Management and Healthcare Policy. 2022 May 10:955-67.

473. Bord S, Satran C, Schor A. The Mediating Role of the Perceived COVID-19 Vaccine Benefits: Examining Israeli Parents' Perceptions Regarding Their Adolescents' Vaccination. Vaccines. 2022 Jun 9;10(6):917.
474. Elkhadry SW, Salem TA, Elshabrawy A, Goda SS, Bahwashy HA, Youssef N, Hussein M, Ghazy RM. COVID-19 Vaccine Hesitancy among Parents of Children with Chronic Liver Diseases. Vaccines. 2022 Dec 7;10(12):2094.
475. ElSayed DA, Bou Raad E, Bekhit SA, Sallam M, Ibrahim NM, Soliman S, Abdullah R, Farag S, Ghazy RM. Validation and cultural adaptation of the parent attitudes about childhood vaccines (PACV) questionnaire in Arabic language widely spoken in a region with a high prevalence of COVID-19 vaccine hesitancy. Tropical Medicine and Infectious Disease. 2022 Sep 8;7(9):234.
476. Ennaceur S, Al-Mohaithef M. Parents' willingness to vaccinate children against COVID-19 in Saudi Arabia: a cross-sectional study. Vaccines. 2022 Jan 21;10(2):156.
477. Hijazi R, Gesser-Edelsburg A, Feder-Bubis P, Mesch GS. Hesitant and anti-vaccination groups: A qualitative study on their perceptions and attitudes regarding vaccinations and their reluctance to participate in academic research-an example during a measles outbreak among a group of Jewish parents in Israel. Frontiers in Public Health. 2022;10.
478. Hussein YH, Ibrahim MH, Badran SG, Eldeeb SM. Hesitancy for influenza vaccine among healthcare workers and mothers of preschool children: A cross-sectional study in Zagazig, Egypt. Journal of Family & Community Medicine. 2022 May;29(2):108.
479. Kharaba Z, Ahmed R, Khalil AM, Al-Ahmed RM, Said AS, Elnour AA, Cherri S, Jirjees F, Afifi H, Ashmawy NS, Mahboub B. Parents' Perception, Acceptance, and Hesitancy to Vaccinate Their Children against COVID-19: Results from a National Study in the UAE. Vaccines. 2022 Aug 31;10(9):1434.
480. Mohammed AH, Hassan BA, Wayyes AM, Gadhban AQ, Blebil A, Alhija SA, Darwish RM, Al-Zaabi AT, Othman G, Jaber AA, Al Shouli BA. Parental health beliefs, intention, and strategies about covid-19 vaccine for their children: A cross-sectional analysis from five Arab countries in the Middle East. Vaccine. 2022 Oct 26;40(45):6549-57.
481. Morozov NG, Dror AA, Daoud A, Eisenbach N, Kaykov E, Barhoum M, Sheleg T, Sela E, Edelstein M. Reasons underlying the intention to vaccinate children aged 5-11 against COVID-19: A cross-sectional study of parents in Israel, November 2021. Human Vaccines & Immunotherapeutics. 2022 Nov 30;18(6):2112879.
482. Reagu S, Mohan S, Awwad J, Alabdulla M. Maternal vaccine hesitancy towards COVID-19 immunisation of children in Qatar: a population-based cross-sectional study. Epidemiology and Health. 2022 Jul 6;44:e2022056.
483. Salawati E, Alwafi H, Samannodi M, Minshawi F, Gari A, Abualnaja S, Almatrafi MA. Parents' willingness to vaccinate their children against seasonal influenza after the COVID-19 pandemic in Saudi Arabia: a retrospective cross-sectional survey. Patient preference and adherence. 2021 Dec 16:2821-35.
484. Samannodi M, Alwafi H, Naser AY, Alabbasi R, Alsahaf N, Alosaimy R, Minshawi F, Almatrafi M, Khalifa R, Ekram R, Salawati E. Assessment of caregiver willingness to vaccinate their children against COVID-19 in Saudi Arabia: a cross-sectional study. Human Vaccines & Immunotherapeutics. 2021 Dec 2;17(12):4857-64.

485. Savitsky B, Shvartsur R, Kagan I. Israeli parentsviews on coronavirus (COVID-19) vaccinations for children: A cross-sectional study. Journal of Pediatric Nursing. 2023 Jan 1;68:79-86.
486. Shati AA, Al-Qahtani SM, Alsabaani AA, Mahmood SE, Alqahtani YA, AlQahtani KM, Aldarami MS, AlAmri FD, Alqahtani AS, AlHadi AM, Ahmad A. Perceptions of Parents towards COVID-19 Vaccination in Children, Aseer Region, Southwestern Saudi Arabia. Vaccines. 2022 Jul 30;10(8):1222.
487. Shmueli L. Parents' intention to vaccinate their 5-to 11-year-old children with the COVID-19 vaccine: rates, predictors and the role of incentives. BMC Public Health. 2023 Dec;23(1):1-0.
488. Swed S, Alibrahim H, Bohsas H, Shoib S, Hasan MM, Motawea KR, Albuni MK, Battikh E, Sawaf B, Elkalagi NK, Mohamed Alsharief Ahmed S. Parents' acceptance to vaccinate children against COVID-19: A Syrian online survey. Frontiers in public health. 2022 Oct 13;10:955362.
489. Tal O, Ne'eman Y, Sadia R, Shmuel R, Schejter E, Bitan M. Parents' attitudes toward children's vaccination as a marker of trust in health systems. Human Vaccines & Immunotherapeutics. 2021 Nov 2;17(11):4518-28.
490. Zach R, Bentwich ME. Reasons for and insights about HPV vaccination refusal among ultra-Orthodox Jewish mothers. Developing World Bioethics. 2022 Oct 6.
491. Abu-Rish EY, Elayeh ER, Mousa LA, Butanji YK, Albsoul-Younes AM. Knowledge, awareness and practices towards seasonal influenza and its vaccine: implications for future vaccination campaigns in Jordan. Family practice. 2016 Aug 27;33(6):690-7.
492. Akgün Ö, Kayaalp GK, Demirkan FG, Çakmak F, Tanatar A, Guliyeva V, Sönmez HE, Ayaz NA. Exploring the attitudes, concerns, and knowledge regarding COVID-19 vaccine by the parents of children with rheumatic disease: Cross-sectional online survey. Vaccine. 2022 Mar 15;40(12):1829-36.
493. Al-Iede M, Khanfar AN, Alshrouf MA, Azzam MI, Haddad TA, Khanfar ON, Al-Tarawneh ZM, Aleidi SM. Parents' attitude towards pneumococcal vaccine: an online survey from Jordan. Journal of International Medical Research. 2022 Oct;50(10):03000605221128151.
494. Al-Rasheedi AT, Elmuttalut MA, Al-Mithn RH, Al-Harbi GS, Al-Ghufaili GS, Al-Mohimeed YH, Al-Qutaymi AA, Al-Arfaj SA. Factors predicting caregivers' readiness for vaccination of 5-11 years old children against SARS-CoV-2-Saudi Arabia, 2022. The Journal of Infection in Developing Countries. 2022 Oct 31;16(10):1533-41.
495. Baş K, Gürarslan Baş N. Parents' Level of COVID-19 Fear, Anxiety and Their Attitudes and Behaviors Toward Vaccination of Their Children. OMEGA-Journal of Death and Dying. 2022 Dec 14:00302228221146377.
496. Çağ Y, Bektemür G, Karabela Ş, Öztürk-Engin D, Çağ Y, Aktaş S, Kart-Yaşar K. Parents' attitudes toward COVID-19 vaccination and childhood vaccines during the COVID-19 pandemic. Asia Pacific Journal of Public Health. 2022 Mar;34(2-3):270-2.
497. Gendler Y, Ofri L. Investigating the influence of vaccine literacy, vaccine perception and vaccine hesitancy on Israeli parents' acceptance of the COVID-19 vaccine for their children: A cross-sectional study. Vaccines. 2021 Dec;9(12):1391.

498. Lau EY, Li JB, King Chung Chan D. Intention to vaccinate young children against COVID-19: a large-scale survey of Hong Kong parents. Human Vaccines & Immunotherapeutics. 2022 Nov 30;18(5):2065838.
499. Nguyen LH, Le TB, Le NQ, Tran NT. Acceptance and Willingness to Pay for Vaccine Against Human Papilloma Virus (HPV) Among Parents of Boys in Central Vietnam. Frontiers in Public Health. 2022;10.
500. Li T, Qiu X, Gong X, Zhan R, Zheng X. The cross-sectional survey on COVID-19 vaccine hesitancy and it predictors among Chinese parents of 3–17 years aged children in Shenzhen City. Annals of Agricultural and Environmental Medicine. 2022;29(1):120.
501. Al-Qerem W, Jarab A, Hammad A, Alasmari F, Ling J, Alsajri AH, Al-Hishma SW, Abu Heshmeh SR. Iraqi parents' knowledge, attitudes, and practices towards vaccinating their children: A cross-sectional study. Vaccines. 2022 May 22;10(5):820.
502. Kocamaz EB, Kocamaz H. Awareness of Covid-19 and attitudes toward vaccination in parents of children between 0 and 18 years: A cross-sectional study. Journal of Pediatric Nursing. 2022 Jul 1;65:75-81.
503. Özer M, Başkaya N, Bostancı İ. Attitudes towards influenza and pneumococcal vaccines in parents of asthmatic children during the COVID-19 pandemic. Pediatric pulmonology. 2022 Apr;57(4):871-7.
504. Imanishi Y, Kinoshita T, Sakamoto M, Ichimiya M, Mitsunami M, Takahashi T, Shigemi D, Song M, Inaba K. Importance of human papillomavirus vaccination leaflets focusing on the safety profile targeted pediatricians in Japan. Vaccine. 2022 Aug 12;40(34):5010-5.
505. Yagi A, Ueda Y, Tomine Y, Ikeda S, Kakuda M, Nakagawa S, Hiramatsu K, Miyoshi A, Kobayashi E, Kimura T, Hirai K. The 'best friend effect': A promising tool to encourage HPV vaccination in Japan. International Journal of Clinical Oncology. 2022 Nov;27(11):1750-7.
506. Hanley SJ, Yoshioka E, Ito Y, Konno R, Hayashi Y, Kishi R, Sakuragi N. Acceptance of and attitudes towards human papillomavirus vaccination in Japanese mothers of adolescent girls. Vaccine. 2012 Aug 24;30(39):5740-7.
507. Shida J, Tsuchiya Y, Inoue K, Maeda K, Yamaki M. Necessary Content of Information Concerning the HPV Vaccine as Assessed by Mothers of Girls. School Health. 2015;11:1-8.
508. Hanley SJ, Yoshioka E, Ito Y, Konno R, Sasaki Y, Kishi R, Sakuragi N. An exploratory study of Japanese fathers' knowledge of and attitudes towards HPV and HPV vaccination: does marital status matter?. Asian Pacific Journal of Cancer Prevention. 2014;15(4):1837-43.
509. Egawa-Takata T, Ueda Y, Morimoto A, Yoshino K, Kimura T, Nishikawa N, Sekine M, Horikoshi Y, Takagi T, Enomoto T. Survey of Japanese mothers of daughters eligible for human papillomavirus vaccination on attitudes about media reports of adverse events and the suspension of governmental recommendation for vaccination. Journal of Obstetrics and Gynaecology Research. 2015 Dec;41(12):1965-71.
510. Shuto M, Kim Y, Okuyama K, Ouchi K, Ueichi H, Nnadi C, Larson HJ, Perez G, Sasaki S. Understanding confidence in the human papillomavirus vaccine in Japan: a web-based survey of mothers, female adolescents, and healthcare professionals. Human vaccines & immunotherapeutics. 2021 Sep 2;17(9):3102-12.

511. Kobayashi KI, Chanyasanha C, Sujirarat D. Parental decision-making on human papillomavirus vaccination for daughters in Japan. International Journal of Adolescent Medicine and Health. 2021 Jun 1;33(3):95-105.
512. Miyoshi A, Takiuchi T, Kimura T. HPV vaccination in Japan: can educational intervention promote a father's intention to encourage his daughter's vaccination?. International journal of clinical oncology. 2020 Apr;25:746-54.
513. Egawa-Takata T, Nakae R, Shindo M, Miyoshi A, Takiuchi T, Miyatake T, Kimura T. Fathers' participation in the HPV vaccination decision-making process doesn't increase parents' intention to make daughters get the vaccine. Human Vaccines & Immunotherapeutics. 2020 Jul 2;16(7):1653-8.
514. Ugumori N, Ueda Y, Yagi A, Abe H, Shiomi M, Nakagawa S, Hiramatsu K, Miyoshi A, Kobayashi E, Kimura T, Kimura T. A potential means to help the HPV vaccine penetrate the Japanese public while under the continued suspension of governmental recommendation. Human Vaccines & Immunotherapeutics. 2021 Sep 2;17(9):3096-101.
515. Yagi A, Ueda Y, Tanaka Y, Nakae R, Kakubari R, Morimoto A, Terai Y, Ohmichi M, Ichimura T, Sumi T, Murata H. Time-dependent changes of the intention of mothers in Japan to inoculate their daughters with the HPV vaccine after suspension of governmental recommendation. Human Vaccines & Immunotherapeutics. 2018 Oct 3;14(10):2497-502.
516. Suzuki Y, Sukegawa A, Ueda Y, Sekine M, Enomoto T, Melamed A, Wright JD, Miyagi E. The Effect of a Web-Based Cervical Cancer Survivor's Story on Parents' Behavior and Willingness to Consider Human Papillomavirus Vaccination for Daughters: Randomized Controlled Trial. JMIR Public Health and Surveillance. 2022 May 25;8(5):e34715.
517. Çelik MY. The thoughts of parents to vaccinate their children against COVID-19: An assessment of situations that may affect them. Journal of Child and Adolescent Psychiatric Nursing. 2022 May;35(2):189-95.
518. Wang Z, She R, Chen X, Li L, Li L, Huang Z, Lau JT. Parental acceptability of COVID-19 vaccination for children under the age of 18 years among Chinese doctors and nurses: a cross-sectional online survey. Human vaccines & immunotherapeutics. 2021 Oct 3;17(10):3322-32.
519. Altulahi N, AlNujaim S, Alabdulqader A, Alkharashi A, AlMalki A, AlSiari F, Bashawri Y, Alsubaie S, AlShahrani D, AlGoraini Y. Willingness, beliefs, and barriers regarding the COVID-19 vaccine in Saudi Arabia: a multiregional cross-sectional study. BMC Family Practice. 2021 Dec;22(1):1-1.
520. Feng H, Zhu H, Zhang H, Cao L, Li L, Wang J, Huang Y, Lai X, Lyu Y, Jing R, Guo J. Caregivers' intentions to COVID-19 vaccination for their children in China: A cross-sectional survey. Human Vaccines & Immunotherapeutics. 2021 Dec 2;17(12):4799-805.
521. Al-Nafeesah AS, Aldamigh AS, Almansoor BA, Al-Wutayd O. The impact of the COVID-19 pandemic on parents' behavior toward scheduled pediatric vaccinations in Saudi Arabia. The Journal of Infection in Developing Countries. 2021 Aug 31;15(08):1054-8.
522. Baghdadi LR, Hassounah MM, Younis A, Al Suwaidan HI, Al Khalifah R. Caregivers' sources of information about immunization as predictors of delayed

childhood vaccinations in saudi arabia during the COVID-19 pandemic: A cross-sectional questionnaire study. Risk Management and Healthcare Policy. 2021 Aug 24:3541-50.
523. Hou Z, Song S, Du F, Shi L, Zhang D, Lin L, Yu H. The influence of the COVID-19 epidemic on prevention and vaccination behaviors among Chinese children and adolescents: cross-sectional online survey study. JMIR Public Health and Surveillance. 2021 May 26;7(5):e26372.
524. Zhou Y, Tang J, Zhang J, Wu Q. Impact of the coronavirus disease 2019 epidemic and a free influenza vaccine strategy on the willingness of residents to receive influenza vaccines in Shanghai, China. Human vaccines & immunotherapeutics. 2021 Jul 3;17(7):2289-92.
525. Zhao T, Wang C, Zhang S, Chen L, Han B, Liu H, Xie M, Cai X, Zhang S, Zhou Y, Li G. What Causes the Discrepancy in SARS-CoV-2 Vaccine Between Parental Hesitancy for Themselves and for Their Children During Lockdown Period?. Journal of Epidemiology and Global Health. 2023 Jun 28:1-3.
526. Zhang H, Chen L, Huang Z, Li D, Tao Q, Zhang F. The effects of parent's health literacy and health beliefs on vaccine hesitancy. Vaccine. 2023 Mar 24;41(13):2120-6.
527. Zhang K, Liang X, Tam KL, Kawuki J, Chan PS, Chen S, Fang Y, Cao H, Zhou X, Chen Y, Hu T. Changes in COVID-19 Vaccine Acceptability among Parents with Children Aged 6–35 Months in China—Repeated Cross-Sectional Surveys in 2020 and 2021. Vaccines. 2023 Jan 12;11(1):170.
528. Ahmed N, Ishtiak AS, Rozars MF, Bonna AS, Alam KP, Hossan ME, Das R, Khan J, Mishu TZ, Afrin S, Sultana N. Factors associated with low childhood immunization coverage among Rohingya refugee parents in Cox's Bazar, Bangladesh. Plos one. 2023 Apr 7;18(4):e0283881.
529. Bourguiba A, AbuHijleh S, Nached Y, Waleed D, Farghaly S, AlOlama F. Assessing Parents' Knowledge, Attitudes, and Practices Toward Vaccinating Children (Five to 15 Years Old) Against COVID-19 in the United Arab Emirates. Cureus. 2022 Dec 17;14(12).
530. Al-Qerem W, Jarab A, Hammad A, Alasmari F, Ling J, Al-Zayadneh E, Al-Iede M, Alazab BA, Hajeer L. Knowledge, Attitudes, and Practices of Influenza Vaccination among Parents of Children with Asthma: A Cross-Sectional Study. Vaccines. 2023 Jun 7;11(6):1074.
531. Ni YH, Xu ZH, Wang J. Understanding vaccine hesitancy with PCV13 in children: Results of a survey in Shanghai, China. Plos one. 2023 Apr 27;18(4):e0284810.
532. Alaamri O, Okmi EA, Suliman Y. Vaccine hesitancy in Saudi Arabia: A cross-sectional study. Tropical Medicine and Infectious Disease. 2022 Apr 12;7(4):60.
533. Alharbi I, Alharthi R, Aljabri S, Alzhrani R, Alzahrani L, Albagami S, Alzhrani RM. Seasonal Influenza Vaccination Among Saudi Children: Parental Barriers and Willingness to Vaccinate Their Children in the Makkah Region. Cureus. 2023 May 11;15(5).
534. Xie H, Zhu HY, Jiang NJ, Yin YN. Awareness of HPV and HPV vaccines, acceptance to vaccination and its influence factors among parents of adolescents 9 to 18 years of age in China: A cross-sectional study. Journal of Pediatric Nursing. 2023 Jul 1;71:73-8.

535. Alhuzaimi AN, Alrasheed AA, Al-Eyadhy A, Aljamaan F, Alhasan K, Batais MA, Jamal A, Alshahrani FS, Alenezi S, Alhaboob A, AlZamil F. Exploring Determinants of COVID-19 Vaccine Acceptance, Uptake, and Hesitancy in the Pediatric Population: A Study of Parents and Caregivers in Saudi Arabia during the Initial Vaccination Phase. InHealthcare 2023 Mar 29 (Vol. 11, No. 7, p. 972). MDPI.
536. Alkalash SH, Alshamrani FA, Alamer EH, Alrabi GM, Almazariqi FA, Shaynawy HM, Alshmrani F, Almazariqi Sr FA, Shaynawy III H. Parents' Knowledge of and Attitude Toward the Human Papillomavirus Vaccine in the Western Region of Saudi Arabia. Cureus. 2022 Dec 19;14(12).
537. Almuqbil M, Al-Asmi R, AlRamly S, Hijazi N, Alotaibi H, AlMubarak A, AlAnezi K, Al-Rowaili M, Al-Yamani M, Duwaidi BS, Alshammari DR. Parental COVID-19 Vaccine Hesitancy for Children and Its Influencing Factors: A Riyadh-Based Cross-Sectional Study. Vaccines. 2023 Feb 23;11(3):518.
538. Topaktaş B, Özdemir Ş, Hasdemir S, Arslan HN, TERZİ O, Dündar C. Assessment of knowledge level and behavior about vaccines of mothers applying to the children's hospital. The European Research Journal. 2022 Apr 11;8(6):828-36.
539. Ashour HA, Alhinti SF, Hawsaoi SA, Alsuwailem AA, AlFarhan A, Abdulmajeed I, Alhinti S, Hawsawi SA, Alsuwailem A. Knowledge, Attitude, and Practice (KAP) of COVID-19 Vaccine Among Saudi Mothers. Cureus. 2023 Mar 28;15(3).
540. Wang X, Yan W, Lu L, Cao L, Tian Y, Zhou K. Chinese parent intention to vaccinate children with special diseases against COVID-19. Frontiers in Public Health. 2021 Oct 27;9:725980.
541. Babi A, Issa T, Issanov A, Akhanova S, Udalova N, Koktova S, Balykov A, Sattarkyzy Z, Imankulova B, Kamzayeva N, Almawi WY. Knowledge and attitudes of mothers toward HPV vaccination: A cross-sectional study in Kazakhstan. Women's Health. 2023 May;19:17455057231172355.
542. Sahoo SS, Parida SP, Singh AK, Palepu S, Sahoo DP, Bhatia V. Decision-making in childhood vaccination: vaccine hesitancy among caregivers of under-5 children from a tertiary care institution in Eastern India. Therapeutic Advances in Vaccines and Immunotherapy. 2023 Feb;11:25151355231152650.
543. Chawanpaiboon S, Anuwutnavin S, Kanjanapongporn A, Pooliam J, Titapant V. Breastfeeding women’s attitudes towards and acceptance and rejection of COVID-19 vaccination: Implementation research. Vaccine. 2023 Feb 3;41(6):1198-208.
544. Tsai CS, Wang LJ, Hsiao RC, Yen CF. Second Wave of the Study of Taiwanese Caregivers of Children with ADHD in the COVID-19 Pandemic: Intentions to Vaccinate Their Children for COVID-19, and Related Factors. Vaccines. 2022 May 11;10(5):753.
545. Cho HK, Lee H, Choe YJ, Kim S, Seo S, Moon J, Choi EH, Kwon GY, Shin JY, Choi SY, Jeong MJ. Parental concerns about COVID-19 vaccine safety and hesitancy in Korea: implications for vaccine communication. Epidemiology and Health. 2023;45.
546. Choi J, Markham C, Tamí-Maury I, Kim S, Cuccaro P. Maternal perceptions of vaccinating boys against human papillomavirus (HPV) in Seoul, South Korea: A descriptive exploratory qualitative study. Plos one. 2023 Mar 10;18(3):e0282811.

547. Yoda T, Katsuyama H. Parents' hesitation about getting their children vaccinated against COVID-19 in Japan. Human vaccines & immunotherapeutics. 2021 Dec 2;17(12):4993-8.
548. Dao TL, Vu Thi H, Gautret P, Al-Tawfiq JA, Nguyen TL, Chu DT, Hoang VT. Willingness and attitudes of parents towards COVID-19 vaccines for children in Vietnam. Journal of Communication in Healthcare. 2023 Jan 2;16(1):75-82.
549. Deng JS, Chen JY, Lin XQ, Huang CL, Tung TH, Zhu JS. Parental hesitancy against COVID-19 vaccination for children and associated factors in Taiwan. BMC Public Health. 2023 Mar 27;23(1):571.
550. Du Y, Wang Y, Zhang T, Ma L, Xie S, Wang Y, Yang Y, Li J, Feng Y, Wang Y, Qi W. Factors associated with PCV13 vaccine hesitancy in parents under an innovative immunization strategy: a cross-sectional study—Weifang city, Shandong province, China, 2021. China CDC Weekly. 2023 Mar 3;5(12):271.
551. Fakhruddin TM, Shafei MN. Knowledge of the Malaysian National Immunisation Programme and its associated factors among parents in Dungun, Terengganu: A cross-sectional study. Malaysian family physician: the official journal of the Academy of Family Physicians of Malaysia. 2023;18:3.
552. Ghazy RM, Sallam M, Fadl N, Bouraad E, Youssef N, Ghoneim OS. Attitude of parents of children with cerebral palsy towards COVID-19 vaccination. International Journal of Environmental Research and Public Health. 2023 Jan 20;20(3):1909.
553. Al Yamani ZJ, AlJohani MM. Vaccine Hesitancy among Parents and its Determinants in PHC in Al Madinah City 2020. The Egyptian Journal of Hospital Medicine. 2022 Apr 1;87(1):1619-25.
554. Zin ZM, Krishnan M, Ilman SS, Jaafar N, Zulkepli MZ, Kadir KA, Ahmad N. Exploring parental refusal of vaccine in Selangor. SEARCH. 2022; 14(2)
555. Maneesriwongul W, Butsing N, Deesamer S. Parental Hesitancy on COVID-19 Vaccination for Children Under Five Years in Thailand: Role of Attitudes and Vaccine Literacy. Patient preference and adherence. 2023 Dec 31:615-28.
556. Khatrawi EM, Sayed AA. The reasons behind COVID-19 vaccination hesitancy among the parents of children aged between 5 to 11 years old in Saudi Arabia. International Journal of Environmental Research and Public Health. 2023 Jan 11;20(2):1345.
557. Khoodoruth MA, Khoodoruth WN, Ramadan AA, Johnson B, Gulistan S, Deluvio RB, Alamri MN, Al-Abdulla M, Ouanes S, Khan YS. Evaluating COVID-19 vaccination intentions and vaccine hesitancy among parents of children with autism spectrum disorder. Scientific Reports. 2023 May 5;13(1):7353.
558. Horiuchi S, Sakamoto H, Abe SK, Shinohara R, Kushima M, Otawa S, Yui H, Akiyama Y, Ooka T, Kojima R, Yokomichi H. Factors of parental COVID-19 vaccine hesitancy: A cross sectional study in Japan. PloS one. 2021 Dec 17;16(12):e0261121.
559. Li K, Zhou F. Influence of Information Sources on Chinese Parents Regarding COVID-19 Vaccination for Children: An Online Survey. International Journal of Environmental Research and Public Health. 2022 Jun 8;19(12):7037.
560. Aljamaan F, Alhaboob A, Saddik B, Bassrawi R, Assiri R, Saeed E, Alhasan K, Alenezi S, Alarabi M, Alrabiaah A, Alkriadees Y. In-person schooling amidst children's COVID-19 vaccination: exploring parental perceptions just after Omicron variant announcement. Vaccines. 2022 May 12;10(5):768.

561. Lin Y, Hu Z, Zhao Q, Alias H, Danaee M, Wong LP. Chinese parents' intentions to vaccinate their children against SARS-CoV-2 infection and vaccine preferences. Human vaccines & immunotherapeutics. 2021 Dec 2;17(12):4806-15.
562. Lu X, Wang J, Hu L, Li B, Lu Y. Association between adult vaccine hesitancy and parental acceptance of childhood COVID-19 vaccines: a web-based survey in a northwestern region in China. Vaccines. 2021 Sep 27;9(10):1088.
563. Rumetta J, Abdul-Hadi H, Lee YK. A qualitative study on parents' reasons and recommendations for childhood vaccination refusal in Malaysia. Journal of infection and public health. 2020 Feb 1;13(2):199-203.
564. Borras-Bermejo B, Panunzi I, Bachy C, Gil-Cuesta J. Missed opportunities for vaccination (MOV) in children up to 5 years old in 19 Médecins Sans Frontières-supported health facilities: a cross-sectional survey in six low-resource countries. BMJ open. 2022 Jul 1;12(7):e059900.
565. Durmaz N, Suman M, Ersoy M, Örün E. Parents' Attitudes toward Childhood Vaccines and COVID-19 Vaccines in a Turkish Pediatric Outpatient Population. Vaccines. 2022 Nov 18;10(11):1958.
566. Goldman RD, Ceballo R, International COVID-19 Parental Attitude Study (COVIPAS) Group. Parental gender differences in attitudes and willingness to vaccinate against COVID-19. Journal of Paediatrics and Child Health. 2022 Jun;58(6):1016-21.
567. Goldman RD, Bone JN, Gelernter R, Krupik D, Klein EJ, Griffiths MA, Mater A, COVIPAS I. Willingness to accept expedited COVID-19 vaccine research for children aged< 12 years after adult vaccine approval. Clinical Therapeutics. 2022 Jan 1;44(1):e1-0.
568. Goldman RD, Krupik D, Ali S, Mater A, Hall JE, Bone, JN, Thompson GC, Yen K, Griffiths MA, Adi K, Klein EJ, Brown JC, Mistry RD, Gelernter R and on behalf of the International COVID-19 Parental Attitude Study (COVIPAS). Caregiver Willingness to Vaccinate Their Children against COVID-19 after Adult Vaccine Approval. International Journal of Environment Research and Public Health. 2021, 18, 10224.
569. Skjefte M, Ngirbabul M, Akeju O, Escudero D, Hernandez-Diaz S, Wyszynski DF, Wu JW. COVID-19 vaccine acceptance among pregnant women and mothers of young children: results of a survey in 16 countries. European journal of epidemiology. 2021 Feb;36:197-211.
570. Goldman RD, Yan TD, Seiler M, Cotanda CP, Brown JC, Klein EJ, Hoeffe J, Gelernter R, Hall JE, Davis AL, Griffiths MA. Caregiver willingness to vaccinate their children against COVID-19: Cross sectional survey. Vaccine. 2020 Nov 10;38(48):7668-73.
571. Goldman RD, Seiler M, Olson PG, Hart RJ, Bone JN, Baumer-Mouradian SH. Factors associated with unvaccinated caregivers who plan to vaccinate their children. Preventive Medicine. 2022 Sep 1;162:107121.
572. Reuben R, Aitken D, Freedman JL, Einstein G. Mistrust of the medical profession and higher disgust sensitivity predict parental vaccine hesitancy. PLoS One. 2020 Sep 2;15(9):e0237755.
573. Urrunaga-Pastor D, Herrera-Añazco P, Uyen-Cateriano A, Toro-Huamanchumo CJ, Rodriguez-Morales AJ, Hernandez AV, Benites-Zapata VA,

Bendezu-Quispe G. Prevalence and factors associated with parents' non-intention to vaccinate their children and adolescents against COVID-19 in Latin America and the Caribbean. Vaccines. 2021 Nov 9;9(11):1303.
574. Sabra HK, Bakr MA, Rageh OE, Khaled A, Elbakliesh OM, Kabbash IA. Parents' perception of COVID-19 risk of infection and intention to vaccinate their children. Vacunas. 2023 Jan 1;24(1):37-44.
575. Yılmaz M, Gökpınar EE, Bozkurt A. Attitudes of the Parents of Children and Adolescents Aged 0-18 with Intellectual Disability to Vaccine. Journal of Pediatric Infection/Cocuk Enfeksiyon Dergisi. 2023 Mar 1;17(1).
576. Tan L, Safadi MA, Horn M, Regojo Balboa C, Moya E, Schanbaum J, Pimenta P, Lambert E, Soumahoro L, Sohn WY, Bruce T. Pandemic's influence on parents' attitudes and behaviors toward meningococcal vaccination. Human Vaccines & Immunotherapeutics. 2023 Dec 31;19(1):2179840.
577. Sharif Nia H, Allen KA, Arslan G, Kaur H, She L, Khoshnavay Fomani F, Gorgulu O, Sivarajan Froelicher E. The predictive role of parental attitudes toward COVID-19 vaccines and child vulnerability: A multi-country study on the relationship between parental vaccine hesitancy and financial well-being. Frontiers in public health. 2023 Feb 16;11:1085197.
578. Tekin Ç, Gokce A, Boz G, Aslan M, Yiğit E. Reasons for parental hesitancy or refusal of childhood vaccination in Türkiye. Eastern Mediterranean Health Journal. 2023;29(5):343-53.
579. Armiento R, Hoq M, Kua E, Crawford N, Perrett KP, Elia S, Danchin M. Impact of Australian mandatory 'No Jab, No Pay'and 'No Jab, No Play'immunisation policies on immunisation services, parental attitudes to vaccination and vaccine uptake, in a tertiary paediatric hospital, the Royal Children's Hospital, Melbourne. Vaccine. 2020 Jul 14;38(33):5231-40.
580. Attwell K, Meyer S, Ward P. The Social Basis of Vaccine Questioning and Refusal: A Qualitative Study Employing Bourdieu's Concepts of 'Capitals' and 'Habitus.' IJERPH 2018; 15:1044.
581. Attwell K, Ward PR, Meyer SB, Rokkas PJ, Leask J. "Do-it-yourself": Vaccine rejection and complementary and alternative medicine (CAM). Social Science & Medicine 2018; 196:106–14
582. Attwell K, Freeman M. I Immunise: An evaluation of a values-based campaign to change attitudes and beliefs. Vaccine 2015; 33:6235–40.
583. Attwell K, Leask J, Meyer SB, Rokkas P, Ward P. Vaccine rejecting parents' engagement with expert systems that inform vaccination programs. Journal of Bioethical Inquiry. 2017 Mar;14(1):65-76
584. Biezen R, Grando D, Mazza D, Brijnath B. Why do we not want to recommend influenza vaccination to young children? A qualitative study of Australian parents and primary care providers. Vaccine. 2018 Feb 1;36(6):859-65.
585. Bryden GM, Browne M, Rockloff M, Unsworth C. The privilege paradox: Geographic areas with highest socio-economic advantage have the lowest rates of vaccination. Vaccine. 2019 Jul 26;37(32):4525-32.
586. Carlsona SJ, McRaec J, Wileya K, Leaske J, Macartneyc K. Knowledge, attitudes and practices regarding influenza vaccination among parents of infants

hospitalised for acute respiratory infection in Australia. Public Health Research & Practice. 2022 Dec 13;32(4):32012202-.
587. Corben P, Leask J. Vaccination hesitancy in the antenatal period: a cross-sectional survey. BMC Public Health. 2018 Dec;18(1):1-3.
588. Enkel SL, Attwell K, Snelling TL, Christian HE. 'Hesitant compliers': Qualitative analysis of concerned fully vaccinating parents. Vaccine. 2018 Oct 22;36(44):6459-63.
589. Forbes TA, McMinn A, Crawford N, Leask J, Danchin M. Vaccination uptake by vaccine-hesitant parents attending a specialist immunization clinic in Australia. Human vaccines & immunotherapeutics. 2015 Dec 2;11(12):2895-903.
590. Frawley JE, Foley H, McIntyre E. The associations between medical, allied and complementary medicine practitioner visits and childhood vaccine uptake. Vaccine. 2018 Feb 1;36(6):866-72.
591. Gilmartin CE, Daley AJ, Leung L. The hepatitis B birth-dose immunisation: Exploring parental refusal. Australian and New Zealand Journal of Obstetrics and Gynaecology. 2020 Feb;60(1):93-100.
592. Helps C, Leask J, Barclay L, Carter S. Understanding non-vaccinating parents' views to inform and improve clinical encounters: a qualitative study in an Australian community. BMJ open. 2019 May 1;9(5):e026299.
593. Rossen I, Hurlstone MJ, Dunlop PD, Lawrence C. Accepters, fence sitters, or rejecters: Moral profiles of vaccination attitudes. Social science & medicine. 2019 Mar 1;224:23-7.
594. Jones K, Fasher B, Hanson R, Burgess M, Isaacs D, Joshi P, Blanch R, Byrne J. Immunization status of Casualty attenders: Risk factors for non-compliance and attitudes to 'on the spot'immunization. Journal of paediatrics and child health. 1992 Dec;28(6):451-4.
595. Rozbroj T, Lyons A, Lucke J. Vaccine-hesitant and vaccine-refusing parents' reflections on the way parenthood changed their attitudes to vaccination. Journal of community health. 2020 Feb;45(1):63-72.
596. Tuckerman J, Crawford NW, Marshall HS. Disparities in parental awareness of children's seasonal influenza vaccination recommendations and influencers of vaccination. PloS one. 2020 Apr 9;15(4):e0230425.
597. Wright D, Rune KT. Underlying motivators for anti-vaccination attitudes amongst regional Sunshine Coast parents in Australia. Health Promotion Journal of Australia. 2022 May 3.
598. Debela MS, Garrett AN, Charania NA. Vaccine hesitancy and its determinants among refugee parents resettled in Aotearoa New Zealand. Human Vaccines & Immunotherapeutics. 2022 Nov 30;18(6):2131336.
599. Jeffs E, Lucas N, Walls T. CoVID-19: parent and caregiver concerns about reopening New Zealand schools. Journal of paediatrics and child health. 2021 Mar;57(3):403-8.
600. Evans S, Klas A, Mikocka-Walus A, German B, Rogers GD, Ling M, Fernando JW, Kothe E, Westrupp EM. "Poison" or "protection"? A mixed methods exploration of Australian parents' COVID-19 vaccination intentions. Journal of psychosomatic research. 2021 Nov 1;150:110626.

601. Wen LM, Xu H, Rissel C, Kerr E, Buchanan L, Taki S, Phongsavan P, Chua RK, Hua M, Wardle K, Simone L. Demographic Predictors of Mothers' Willingness to Vaccinate Young Children Against COVID-19, Get Tested and Isolate: A Cross-Sectional Survey Before and During the Greater Sydney Lockdown 2021, Australia. Frontiers in Public Health. 2022;10.
602. Bolsewicz KT, Steffens MS, King C, Abdi I, Bullivant B, Beard F. A qualitative study on COVID-19 pandemic impacts on parental attitudes and intentions for routine adolescent vaccinations: The role of trust. Vaccine. 2023 Jun 23;41(28):4138-43.
603. Napolitano F, D'Alessandro A, Angelillo IF. Investigating Italian parents' vaccine hesitancy: A cross-sectional survey. Human vaccines & immunotherapeutics. 2018 Jul 3;14(7):1558-65.
604. Paterson P, Chantler T, Larson HJ. Reasons for non-vaccination: Parental vaccine hesitancy and the childhood influenza vaccination school pilot programme in England. Vaccine. 2018 Aug 28;36(36):5397-401.
605. Babicki M, Pokorna-Kałwak D, Doniec Z, Mastalerz-Migas A. Attitudes of parents with regard to vaccination of children against COVID-19 in Poland. A nationwide online survey. Vaccines. 2021 Oct 17;9(10)
606. Sobierajski T, Małecka I, Augustynowicz E. Feminized vaccine? Parents' attitudes toward HPV vaccination of adolescents in Poland: A representative study. Human Vaccines & Immunotherapeutics. 2023 Dec 31;19(1):2186105.
607. Brunelli L, Valent F, Romanese F, Tricarico P, Pellizzaro A, d'Angelo M, Benetollo PP, Iob A, Forgiarini M, Brusaferro S. Parental trust and beliefs after the discovery of a six-year-long failure to vaccinate. Human Vaccines & Immunotherapeutics. 2021 Feb 1;17(2):583-7.
608. Tho SL, Ader F, Ferry T, Floret D, Arnal M, Fargeas S, Chidiac C, Valour F. Vaccination against serogroup B Neisseria meningitidis: Perceptions and attitudes of parents. Vaccine. 2015 Jul 9;33(30):3463-70.
609. Koźlarek M, Błaszczyk N, Grajek M, Jaskulska S. Preventive Health Behaviours among Adolescents and Their Parents during the COVID-19 Outbreak in the Light of the Health Beliefs Model. International Journal of Environmental Research and Public Health. 2022 Jan;19(24):17060.
610. Abuduxike G, Asut O, Cali S, Vaizoglu S. Knowledge and Awareness of Parents Towards Human Papillomavirus (HPV) and HPV Vaccines, and Vaccine Acceptability in Northern Cyprus. Journal of Prevention. 2022 Apr;43(2):225-40.
611. Alfredsson R, Svensson E, Trollfors B, Borres MP. Why do parents hesitate to vaccinate their children against measles, mumps and rubella?. Acta paediatrica. 2004 Sep;93(9):1232-7.
612. Allaert F-A, Blanc A, Megard Y, Bertand I. Parents' attitudes towards varicella vaccination acceptance in France and Germany: effect of vaccine recommendation and reimbursement (a survey). J Public Health 2009; 17:71–6.
613. Amdisen L, Kristensen ML, Rytter D, Mølbak K, Valentiner-Branth P. Identification of determinants associated with uptake of the first dose of the human papillomavirus vaccine in Denmark. Vaccine. 2018 Sep 11;36(38):5747-53.
614. Anello P, Cestari L, Baldovin T, Simonato L, Frasca G, Caranci N, Pascucci MG, Valent F, Canova C. Socioeconomic factors influencing childhood vaccination in two northern Italian regions. Vaccine. 2017 Aug 24;35(36):4673-80.

615. Bankiewicz P, Dworakowska AM, Makarewicz-Wujec M, Kozłowska-Wojciechowska M. Beliefs and sentiments of parents vaccinating their children-small town perspective in Poland: a preliminary study. Central European Journal of Public Health. 2022 Mar 31;30(1):7-12.
616. Bell S, Clarke R, Mounier-Jack S, Walker JL, Paterson P. Parents’ and guardians’ views on the acceptability of a future COVID-19 vaccine: A multi-methods study in England. Vaccine. 2020 Nov 17;38(49):7789-98.
617. Bertoncello C, Ferro A, Fonzo M, Zanovello S, Napoletano G, Russo F, Baldo V, Cocchio S. Socioeconomic determinants in vaccine hesitancy and vaccine refusal in Italy. Vaccines. 2020 Jun 5;8(2):276.
618. Betsch C, Bödeker B, Schmid P, Wichmann O. How baby’s first shot determines the development of maternal attitudes towards vaccination. Vaccine. 2018 May 17;36(21):3018-26.
619. Bianco A, Mascaro V, Zucco R, Pavia M. Parent perspectives on childhood vaccination: How to deal with vaccine hesitancy and refusal? Vaccine. 2019 Feb 8;37(7):984-90.
620. Bianco A, Della Polla G, Angelillo S, Pelullo CP, Licata F, Angelillo IF. Parental COVID-19 vaccine hesitancy: a cross-sectional survey in Italy. Expert review of vaccines. 2022 Apr 3;21(4):541-7.
621. Bielecki K, Craig J, Willocks LJ, Pollock KG, Gorman DR. Impact of an influenza information pamphlet on vaccination uptake among Polish pupils in Edinburgh, Scotland and the role of social media in parental decision making. BMC public health. 2020 Dec;20(1):1-1.
622. Bocquier A, Fressard L, Cortaredona S, Zaytseva A, Ward J, Gautier A, Peretti-Watel P, Verger P. Social differentiation of vaccine hesitancy among French parents and the mediating role of trust and commitment to health: a nationwide cross-sectional study. Vaccine. 2018 Nov 29;36(50):7666-73.
623. Borràs E, Domínguez À, Fuentes M, Batalla J, Cardeñosa N, Plasencia A. Parental knowledge of paediatric vaccination. BMC Public Health. 2009 Dec;9(1):1-7.
624. Brandstetter S, Böhmer MM, Pawellek M, Seelbach-Göbel B, Melter M, Kabesch M, Apfelbacher C. Parents’ intention to get vaccinated and to have their child vaccinated against COVID-19: cross-sectional analyses using data from the KUNO-Kids health study. European journal of pediatrics. 2021 Nov;180(11):3405-10.
625. Brown KF, Kroll JS, Hudson MJ, Ramsay M, Green J, Vincent CA, Fraser G, Sevdalis N. Omission bias and vaccine rejection by parents of healthy children: implications for the influenza A/H1N1 vaccination programme. Vaccine. 2010 Jun 7;28(25):4181-5.
626. Bults M, Beaujean DJ, Richardus JH, van Steenbergen JE, Voeten HA. Pandemic influenza A (H1N1) vaccination in The Netherlands: parental reasoning underlying child vaccination choices. Vaccine. 2011 Aug 26;29(37):6226-35.
627. Buonsenso D, Valentini P, Macchi M, Folino F, Pensabene C, Patria MF, Agostoni C, Castaldi S, Lecce M, Giannì ML, Marchisio P. Caregivers' attitudes toward COVID-19 Vaccination in children and adolescents with a history of SARS-CoV-2 infection. Frontiers in Pediatrics. 2022;10.

628. Byström E, Lindstrand A, Likhite N, Butler R, Emmelin M. Parental attitudes and decision-making regarding MMR vaccination in an anthroposophic community in Sweden–a qualitative study. Vaccine. 2014 Nov 28;32(50):6752-7.
629. Byström E, Lindstrand A, Bergström J, Riesbeck K, Roth A. Confidence in the National Immunization Program among parents in Sweden 2016–A cross-sectional survey. Vaccine. 2020 May 8;38(22):3909-17.
630. Calagna G, Granese R, Giallombardo V, Capra G, Perino A, Schiattarella A, Trucchi C, Caridi G. Attitudes of mothers of pre-adolescent girls on HPV vaccine in Italy. Do we need a turning point?. Minerva Obstetrics and Gynecology. 2022 Oct 4.
631. Campbell H, Edwards A, Letley L, Bedford H, Ramsay M, Yarwood J. Changing attitudes to childhood immunisation in English parents. Vaccine. 2017 May 19;35(22):2979-85.
632. Casiday R, Cresswell T, Wilson D, Panter-Brick C. A survey of UK parental attitudes to the MMR vaccine and trust in medical authority. Vaccine. 2006 Jan 12;24(2):177-84.
633. Caso D, Capasso M, Fabbricatore R, Conner M. Understanding the psychosocial determinants of Italian parents' intentions not to vaccinate their children: an extended theory of planned behaviour model. Psychology & health. 2022 Aug 8;37(9):1111-31.
634. Cassell JA, Leach M, Poltorak MS, Mercer CH, Iversen A, Fairhead JR. Is the cultural context of MMR rejection a key to an effective public health discourse?. Public health. 2006 Sep 1;120(9):783-94.
635. Ceannt R, Vallieres F, Burns H, Murphy J, Hyland P. Covid-19 vaccine hesitancy and resistance amongst parents of children under 18 years of age in Ireland. Vaccine. 2022 Oct 12;40(43):6196-200.
636. Charron J, Gautier A, Jestin C. Influence of information sources on vaccine hesitancy and practices. Medecine et maladies infectieuses. 2020 Nov 1;50(8):727-33.
637. Cintulová LL. The impact of the emotions that frame mothers' decision-making about the vaccination of toddlers. Kontakt. 2019;21:189-96.
638. Craciun C, Baban A. "Who will take the blame?": Understanding the reasons why Romanian mothers decline HPV vaccination for their daughters. Vaccine. 2012 Nov 6;30(48):6789-93.
639. Dannetun E, Tegnell A, Hermansson G, Giesecke J. Parents' reported reasons for avoiding MMR vaccination: a telephone survey. Scandinavian journal of primary health care. 2005 Jan 1;23(3):149-53.
640. Dáňová J, Šálek J, Kocourková A, Čelko AM. Factors associated with parental refusal of routine vaccination in the Czech Republic. Central European journal of public health. 2015 Dec 30;23(4):321-3.
641. Deml MJ, Buhl A, Huber BM, Burton-Jeangros C, Tarr PE. Trust, affect, and choice in parents' vaccination decision-making and health-care provider selection in Switzerland. Sociology of health & illness. 2022 Jan;44(1):41-58.
642. Derdemezis C, Markozannes G, Rontogianni MO, Trigki M, Kanellopoulou A, Papamichail D, Aretouli E, Ntzani E, Tsilidis KK. Parental hesitancy towards the established childhood vaccination programmes in the COVID-19 era: Assessing the drivers of a challenging public health concern. Vaccines. 2022 May 20;10(5):814.

643. Di Giuseppe G, Paduano G, Vaienna S, Maisto G, Pelullo CP, Pavia M. Surveying parents' awareness and adherence to influenza vaccination recommendations in children in Southern Italy. Vaccines. 2022 Aug 11;10(8):1298.
644. Ebi SJ, Deml MJ, Jafflin K, Buhl A, Engel R, Picker J, Häusler J, Wingeier B, Krüerke D, Huber BM, Merten S. Parents' vaccination information seeking, satisfaction with and trust in medical providers in Switzerland: a mixed-methods study. BMJ open. 2022 Feb 1;12(2):e053267.
645. Erb ML, Erlanger TE, Heininger U. Child-parent immunization survey: How well are national immunization recommendations accepted by the target groups?. Vaccine: X. 2019 Apr 11;1:100013.
646. Evans M, Stoddart H, Condon L, Freeman E, Grizzell M, Mullen R. Parents' perspectives on the MMR immunisation: a focus group study. British Journal of General Practice. 2001 Nov 1;51(472):904-10.
647. Facciolà A, Visalli G, Orlando A, Bertuccio MP, Spataro P, Squeri R, Picerno I, Pietro AD. Vaccine hesitancy: An overview on parents' opinions about vaccination and possible reasons of vaccine refusal. Journal of public health research. 2019 Mar 11;8(1):jphr-2019.
648. Fakonti G, Hadjikou A, Tzira E, Kyprianidou M, Giannakou K. Attitudes and perceptions of mothers towards childhood vaccination in Greece: lessons to improve the childhood COVID-19 vaccination acceptance. Frontiers in Pediatrics. 2022;10.
649. Fedele F, Aria M, Esposito V, Micillo M, Cecere G, Spano M, De Marco G. COVID-19 vaccine hesitancy: a survey in a population highly compliant to common vaccinations. Human vaccines & immunotherapeutics. 2021 Oct 3;17(10):3348-54.
650. Fonseca IC, Pereira AI, Barros L. Portuguese parental beliefs and attitudes towards vaccination. Health Psychology and Behavioral Medicine. 2021 Jan 1;9(1):422-35.
651. Gács Z, Koltai J. Understanding Parental Attitudes toward Vaccination: Comparative Assessment of a New Tool and Its Trial on a Representative Sample in Hungary. Vaccines. 2022 Dec;10(12):2006.
652. Galanis P, Vraka I, Siskou O, Konstantakopoulou O, Katsiroumpa A, Moisoglou I, Kaitelidou D. Predictors of real-world parents' acceptance to vaccinate their children against the COVID-19. Acta Medica Lituanica. 2022;29(1):33.
653. Galanis P, Vraka I, Siskou O, Konstantakopoulou O, Katsiroumpa A, Moisoglou I, Kaitelidou D. Predictors of parents' intention to vaccinate their children against the COVID-19 in Greece: a cross-sectional study. medRxiv. 2021 Sep 29:2021-09.
654. Giambi C, Fabiani M, D'Ancona F, Ferrara L, Fiacchini D, Gallo T, Martinelli D, Pascucci MG, Prato R, Filia A, Bella A. Parental vaccine hesitancy in Italy–results from a national survey. Vaccine. 2018 Feb 1;36(6):779-87
655. Gorman DR, Bielecki K, Larson HJ, Willocks LJ, Craig J, Pollock KG. Comparing vaccination hesitancy in Polish migrant parents who accept or refuse nasal flu vaccination for their children. Vaccine. 2020 Mar 17;38(13):2795-9.
656. Grandahl M, Tydén T, Westerling R, Nevéus T, Rosenblad A, Hedin E, Oscarsson M. To consent or decline HPV vaccination: a pilot study at the start of the national school-based vaccination program in Sweden. Journal of school health. 2017 Jan;87(1):62-70.

657. Grandahl M, Oscarsson M, Stenhammar C, Nevéus T, Westerling R, Tydén T. Not the right time: why parents refuse to let their daughters have the human papillomavirus vaccination. Acta paediatrica. 2014 Apr;103(4):436-41.
658. Harmsen, I.A., Doorman, G.G., Mollema, L., Ruiter, R.A., Kok, G. and de Melker, H.E., 2013. Parental information-seeking behaviour in childhood vaccinations. BMC Public Health, 13(1), pp.1-10.
659. Harmsen IA, Mollema L, Ruiter RA, Paulussen TG, de Melker HE, Kok G. Why parents refuse childhood vaccination: a qualitative study using online focus groups. BMC public health. 2013 Dec;13(1):1-8.
660. Harmsen IA, Ruiter RA, Paulussen TG, Mollema L, Kok G, de Melker HE. Factors that influence vaccination decision-making by parents who visit an anthroposophical child welfare center: a focus group study. Advances in preventive medicine. 2012 Jan 1;2012.
661. Herdea V, Ghionaru R, Lungu CN, Leibovitz E, Diaconescu S. Vaccine Coverage in Children Younger Than 1 Year of Age during Periods of High Epidemiological Risk: Are We Preparing for New Outbreaks?. Children. 2022 Sep 1;9(9):1334.
662. Hilton S, Petticrew M, Hunt K. Combined vaccines are like a sudden onslaught to the body's immune system': Parental concerns about vaccine 'overload'and 'immune-vulnerability. Vaccine. 2006 May 15;24(20):4321-7.
663. Hontelez JA, Hahné SJ, Oomen P, de Melker H. Parental attitude towards childhood HBV vaccination in The Netherlands. Vaccine. 2010 Jan 22;28(4):1015-20.
664. Huber A, Gazder J, Dobay O, Mészner Z, Horváth A. Attitudes towards varicella vaccination in parents and paediatric healthcare providers in Hungary. Vaccine. 2020 Jul 14;38(33):5249-55.
665. Iannello P, Colautti L, Magenes S, Antonietti A, Cancer A. Black-and-white thinking and conspiracy beliefs prevent parents from vaccinating their children against COVID-19. Applied Cognitive Psychology. 2022 Sep 21.
666. Jafflin K, Deml MJ, Schwendener CL, Kiener L, Delfino A, Gafner R, Schudel S, Mäusezahl M, Berger C, Huber BM, Merten S. Parental and provider vaccine hesitancy and non-timely childhood vaccination in Switzerland. Vaccine. 2022 May 20;40(23):3193-202.
667. Jama A, Ali M, Lindstrand A, Butler R, Kulane A. Perspectives on the measles, mumps and rubella vaccination among Somali mothers in Stockholm. International Journal of Environmental Research and Public Health. 2018 Nov;15(11):2428.
668. Kornfeld J, Byrne MM, Vanderpool R, Shin S, Kobetz E. HPV knowledge and vaccine acceptability among Hispanic fathers. The journal of primary prevention. 2013 Apr;34(1):59-69.
669. Krakowczyk JB, Bäuerle A, Pape L, Kaup T, Nulle L, Teufel M, Skoda EM. COVID-19 vaccine for children: vaccination willingness of parents and its associated factors—a network analysis. Vaccines. 2022 Jul 20;10(7):1155.
670. Lecce M, Milani GP, Agostoni C, D'Auria E, Banderali G, Biganzoli G, Castellazzi L, Paramithiotti C, Salvatici E, Tommasi P, Zuccotti GV. Caregivers' Intention to Vaccinate Their Children Under 12 Years of Age Against COVID-19: A Cross-Sectional Multi-Center Study in Milan, Italy. Frontiers in Pediatrics. 2022 May 30;10:834363.

671. Lewandowska A, Lewandowski T, Rudzki G, Rudzki S, Laskowska B. Opinions and knowledge of parents regarding preventive vaccinations of children and causes of reluctance toward preventive vaccinations. International journal of environmental research and public health. 2020 Jan;17(10):3694.
672. López N, Salamanca de la Cueva I, Vergés E, Suarez Vicent E, Sánchez A, López AB, Panizo-Santos MB, Garcés-Sánchez M, Montesdeoca A, Rivera AJ, Cotarelo MS. Factors influencing HPV knowledge and vaccine acceptability in parents of adolescent children: results from a survey-based study (KAPPAS study). Human Vaccines & Immunotherapeutics. 2022 Jan 31;18(1):2024065.
673. López N, de la Cueva IS, Taborga E, de Alba AF, Cabeza I, Raba RM, Marès J, Herrera B, Cotarelo M. HPV knowledge and vaccine acceptability: a survey-based study among parents of adolescents (KAPPAS study). Infectious Agents and Cancer. 2022 Dec;17(1):1-1.
674. Mameli C, Faccini M, Mazzali C, Picca M, Colella G, Duca PG, Zuccotti GV. Acceptability of meningococcal serogroup B vaccine among parents and health care workers in Italy: a survey. Human Vaccines & Immunotherapeutics. 2014 Oct 3;10(10):3004-10.
675. Manolescu LS, Zaharia CN, Dumitrescu AI, Prasacu I, Radu MC, Boeru AC, Boidache L, Nita I, Necsulescu A, Medar C, Cristache CM. COVID-19 parental vaccine Hesitancy in Romania: nationwide cross-sectional study. Vaccines. 2022 Mar 23;10(4):493.
676. Mărcău FC, Peptan C, Nedelcuță RM, Băleanu VD, Băleanu AR, Niculescu B. Parental COVID-19 Vaccine Hesitancy for Children in Romania: National Survey. Vaccines. 2022 Apr 1;10(4):547.
677. Martínez-Diz S, Romero MM, Fernández-Prada M, Piqueras MC, Ruano RM, Sierra MF. Demands and expectations of parents who refuse vaccinations and perspective of health professional on the refusal to vaccinate. Anales de Pediatría (English Edition). 2014 Jun 1;80(6):370-8.
678. McHale P, Keenan A, Ghebrehewet S. Reasons for measles cases not being vaccinated with MMR: investigation into parents' and carers' views following a large measles outbreak. Epidemiology & Infection. 2016 Mar;144(4):870-5
679. Miko D, Costache C, Colosi HA, Neculicioiu V, Colosi IA. Qualitative assessment of vaccine hesitancy in Romania. Medicina. 2019 Jun 17;55(6):282.
680. Miliordos K, Giannouchos T, Steletou E, Sanidas G, Karkania A, Vervenioti A, Dimitriou G, Gkentzi D. Parental attitudes towards vaccination against COVID-19 of children 5–11 years old in Greece. Journal of Evaluation in Clinical Practice. 2022 Dec;28(6):943-7.
681. Miraglia del Giudice G, Napoli A, Corea F, Folcarelli L, Angelillo IF. Evaluating COVID-19 vaccine willingness and hesitancy among parents of children aged 5–11 years with chronic conditions in Italy. Vaccines. 2022 Mar 4;10(3):396.
682. Miron VD, Toma AR, Filimon C, Bar G, Craiu M. Optional Vaccines in Children—Knowledge, Attitudes, and Practices in Romanian Parents. Vaccines. 2022 Mar 7;10(3):404.
683. Montalti M, Rallo F, Guaraldi F, Bartoli L, Po G, Stillo M, Perrone P, Squillace L, Dallolio L, Pandolfi P, Resi D. Would parents get their children vaccinated against

SARS-CoV-2? Rate and predictors of vaccine hesitancy according to a survey over 5000 families from Bologna, Italy. Vaccines. 2021 Apr 10;9(4):366.

684. Naoum P, Athanasakis K, Zavras D, Kyriopoulos J, Pavi E. Knowledge, perceptions and attitudes toward HPV vaccination: a survey on parents of girls aged 11–18 years old in Greece. Frontiers in Global Women's Health. 2022;3.

685. Napoli A, Del Giudice GM, Corea F, Folcarelli L, Angelillo IF. Parents' reasons to vaccinate their children aged 5–11 years against COVID-19 in Italy. Frontiers in Medicine. 2022;9.

686. Napolitano F, Miraglia del Giudice G, Angelillo S, Fattore I, Licata F, Pelullo CP, Di Giuseppe G. Hesitancy towards childhood vaccinations among parents of children with underlying chronic medical conditions in Italy. Vaccines. 2022 Aug 4;10(8):1254.

687. Nurmi J, Harman B. Why do parents refuse childhood vaccination? Reasons reported in Finland. Scandinavian Journal of Public Health. 2022 Jun;50(4):490-6.

688. Olszewska M, Smykla B, Gdańska M, Kiełbasa G, Ficinski M, Szymońska I, Starzec K, Kwinta P. The analysis of parental attitude towards active immunoprophylaxis and its influence on the implementation of an immunization schedule among children in Poland. Children's Health Care. 2018 Jul 3;47(3):289-307.

689. Navarro-Illana P, Caballero P, Tuells J, Puig-Barbera J, Diez-Domingo J. Acceptability of human papillomavirus vaccine in mothers from Valencia (Spain). Anales de Pediatría (English Edition). 2015 Nov 1;83(5):318-27

690. Peretti-Watel P, Ward JK, Vergelys C, Bocquier A, Raude J, Verger P. 'I think I made the right decision… I hope I'm not wrong'. Vaccine hesitancy, commitment and trust among parents of young children. Sociology of health & illness. 2019 Jul;41(6):1192-206.

691. Della Polla G, Pelullo CP, Napolitano F, Angelillo IF. HPV vaccine hesitancy among parents in Italy: A cross-sectional study. Human vaccines & immunotherapeutics. 2020 Nov 1;16(11):2744-51.

692. Prospero E, Galmozzi S, Paris V, Felici G, Barbadoro P, D'Alleva A, Zocco G, Ciavattini A. Factors influencing refusing of flu vaccination among pregnant women in Italy: healthcare workers' role. Influenza and Other Respiratory Viruses. 2019 Mar;13(2):201-7.

693. Rees F, Geiger M, Lilleholt L, Zettler I, Betsch C, Böhm R, Wilhelm O. Measuring parents' readiness to vaccinate themselves and their children against COVID-19. Vaccine. 2022 Jun 21;40(28):3825-34.

694. Restivo V, Napoli G, Marsala MG, Bonanno V, Sciuto V, Amodio E, Calamusa G, Vitale F, Firenze A. Factors associated with poor adherence to MMR vaccination in parents who follow vaccination schedule. Human Vaccines & Immunotherapeutics. 2015 Jan 1;11(1):140-5.

695. Price T, McColl E, Visram S. Barriers and facilitators of childhood flu vaccination: the views of parents in North East England. Journal of Public Health. 2022 Nov;30(11):2619-26.

696. Runngren E, Eriksson M, Blomberg K. Parents' reasoning about HPV vaccination in Sweden. Scandinavian Journal of Caring Sciences. 2022 Dec;36(4):1113-22.

697. De Gioia ER, Porqueddu A, Nebiaj O, Bianconi A, Conni A, Montalti M, Pandolfi P, Todeschini R, Fantini MP, Gori D. The Role of Needle Fear in Pediatric Flu Vaccine Hesitancy: A Cross-Sectional Study in Bologna Metropolitan Area. Vaccines. 2022 Aug 25;10(9):1388.
698. Steletou E, Giannouchos T, Karatza A, Sinopidis X, Vervenioti A, Souliotis K, Dimitriou G, Gkentzi D. Parental and Pediatricians' Attitudes towards COVID-19 Vaccination for Children: Results from Nationwide Samples in Greece. Children. 2022 Aug 11;9(8):1211.
699. Di Giuseppe G, Pelullo CP, Volgare AS, Napolitano F, Pavia M. Parents' willingness to vaccinate their children with COVID-19 vaccine: Results of a survey in Italy. Journal of Adolescent Health. 2022 Apr 1;70(4):550-8.
700. Karafillakis E, Peretti-Watel P, Verger P, Chantler T, Larson HJ. "We don't have the same bodies; we don't react the same way": mothers and adolescent girls' perceptions of the risks and benefits of HPV vaccination in France. Human Vaccines & Immunotherapeutics. 2022 Jan 31;18(1):2036555.
701. Smith LE, Weinman J, Amlôt R, Yiend J, Rubin GJ. Parental expectation of side effects following vaccination is self-fulfilling: a prospective cohort study. Annals of Behavioral Medicine. 2019 Mar;53(3):267-82.
702. Rodríguez-Blanco N, Tuells J. Knowledge and attitudes about the flu vaccine among pregnant women in the valencian community (Spain). Medicina. 2019 Aug 11;55(8):467.
703. Romijnders KA, van Seventer SL, Scheltema M, van Osch L, de Vries H, Mollema L. A deliberate choice? Exploring factors related to informed decision-making about childhood vaccination among acceptors, refusers, and partial acceptors. Vaccine. 2019 Sep 3;37(37):5637-44.
704. Schmidtke KA, Kudrna L, Noufaily A, Stallard N, Skrybant M, Russell S, Clarke A. Evaluating the relationship between moral values and vaccine hesitancy in Great Britain during the COVID-19 pandemic: A cross-sectional survey. Social Science & Medicine. 2022 Sep 1;308:115218.
705. Selleri P, Carugati F. Mothers and vaccinations: From personal experiences to shared representations. A challenge for healthcare authorities. Italian Journal of Sociology of Education. 2020;12(3).
706. Seoane MD, Hernández CB, Tango MA, Rodriguez CG, López PS, Martín MP, Pérez MG. Factors related to parents' decision not to vaccinate their children. Vacunas (English Edition). 2020 Jan 1;21(1):4-10.
707. Skirrow H, Barnett S, Bell S, Mounier-Jack S, Kampmann B, Holder B. Women's views and experiences of accessing pertussis vaccination in pregnancy and infant vaccinations during the COVID-19 pandemic: a multi-methods study in the UK. Vaccine. 2022 Aug 12;40(34):4942-54.
708. Skitarelić N, Vidaić M, Skitarelić N. Parents' versus Grandparents' Attitudes about Childhood Vaccination. Children. 2022 Mar 2;9(3):345.
709. Smolarczyk K, Duszewska A, Drozd S, Majewski S. Parents' Knowledge and Attitude towards HPV and HPV Vaccination in Poland. Vaccines. 2022 Feb 2;10(2):228.

710. Stampi S, Ricci R, Ruffilli I, Zanetti F. Compulsory and recommended vaccination in Italy: evaluation of coverage and non-compliance between 1998-2002 in Northern Italy. BMC Public Health. 2005 Dec;5(1):1-8.
711. Stoeckel F, Carter C, Lyons BA, Reifler J. Association of vaccine hesitancy and immunization coverage rates in the European Union. Vaccine. 2021 Jun 29;39(29):3935-9.
712. Sythes L, Bedford H. Motherhood and vaccine refusal in the United Kingdom: A new examination of gender, identity and the journey to contemporary non-vaccination. Child: Care, Health and Development. 2022 Nov;48(6):979-89.
713. Taylor J, Nailer E, Cohen CR, Redman CW, Sherman SM. HPV vaccination and cervical screening: the knowledge and attitudes of mothers of adolescent girls. Psychology & Health. 2022 May 21:1-8.
714. Van Hoecke AL, Sanders JG. An Online Experiment of NHS Information Framing on Mothers' Vaccination Intention of Children against COVID-19. Vaccines. 2022 May 4;10(5):720.
715. Venderbos JR, Eilers R, de Vries H, van Zoonen K. A qualitative study of parental associations and beliefs regarding the HPV vaccination for Dutch boys. BMC Public Health. 2022 Jun 14;22(1):1188.
716. Vrdelja M, Kraigher A, Verčič D, Kropivnik S. The growing vaccine hesitancy: exploring the influence of the internet. European journal of public health. 2018 Oct 1;28(5):934-9.
717. Wagner A, Liberatore F, Schmelzer S, Dratva J. Confident and altruistic– parents' motives to vaccinate their children against COVID-19: a cross-sectional online survey in a Swiss vaccination centre. Swiss Medical Weekly. 2022 Mar 18;152:w30156.
718. Waller J, Forster A, Ryan M, Richards R, Bedford H, Marlow L. Decision-making about HPV vaccination in parents of boys and girls: A population-based survey in England and Wales. Vaccine. 2020 Jan 29;38(5):1040-7.
719. Weiss C, Schröpfer D, Merten S. Parental attitudes towards measles vaccination in the canton of Aargau, Switzerland: a latent class analysis. BMC infectious diseases. 2016 Dec;16(1):1-8.
720. Whelan SO, Moriarty F, Lawlor L, Gorman KM, Beamish J. Vaccine hesitancy and reported non-vaccination in an Irish pediatric outpatient population. European Journal of Pediatrics. 2021 Sep;180(9):2839-47.
721. Wood L, Smith M, Miller CB, O'Carroll RE. The internal consistency and validity of the Vaccination Attitudes Examination Scale: A replication study. Annals of Behavioral Medicine. 2019 Jan;53(1):109-14.
722. Roberts RJ, Sandifer QD, Evans MR, Nolan-Farrell MZ, Davis PM. Reasons for non-uptake of measles, mumps, and rubella catch up immunisation in a measles epidemic and side effects of the vaccine. BMJ. 1995 Jun 24;310(6995):1629-39.
723. Braczkowska B, Kowalska M, Barański K, Gajda M, Kurowski T, Zejda JE. Parental opinions and attitudes about children's vaccination safety in Silesian Voivodeship, Poland. International journal of environmental research and public health. 2018 Apr;15(4):756.

724. Ruggiero KM, Wong J, Sweeney CF, Avola A, Auger A, Macaluso M, Reidy P. Parents' intentions to vaccinate their children against COVID-19. Journal of Pediatric Health Care. 2021 Sep 1;35(5):509-17.
725. Gjini E, Moramarco S, Carestia MC, Cenko F, Ylli A, Mehmeti I, Palombi L, Buonomo E. Parents' and caregivers' role toward childhood vaccination in Albania: assessment of predictors of vaccine hesitancy. Annali di Igiene, Medicina Preventiva e di Comunita. 2023 Jan 1;35(1).
726. Seiler M, Goldman RD, Staubli G, Hoeffe J, Gualco G, Manzano S. Parents' intent to vaccinate against influenza during the COVID-19 pandemic in two regions in Switzerland. Swiss medical weekly. 2021 May 12;151:w20508.
727. Kirkedal AB, Møller JE, Stensballe LG, Zoffmann V. Parents' and Health Professionals' Attitudes to Advancing Primary MMR Vaccine Administration from Fifteen to Six Months of Age—A Qualitative Thematic Analysis Embedded in a Randomized Trial. Vaccines. 2022 Dec 28;11(1):67.
728. Ateş BÖ, Özyavuz G, Cöngöloğlu MA. COVID-19 vaccine hesitancy of adolescents with psychiatric disorders and their parents: data from a child psychiatry outpatient clinic. The Turkish Journal of Pediatrics. 2023 Mar 1;65(2):205-17.
729. Avcı D, Kuş C, Gümüştakim RŞ, Başer DA, Eryilmaz ME. Knowledge, attitudes and behaviors of family physicians about childhood vaccinations that are not in the routine vaccination schedule: a cross-sectional study. Primary Health Care Research & Development. 2023;24:e2.
730. Sherman SM, Lingley-Heath N, Lai J, Sim J, Bedford H. Parental acceptance of and preferences for administration of routine varicella vaccination in the UK: a study to inform policy. Vaccine. 2023 Feb 17;41(8):1438-46.
731. Savarese G, Carpinelli L, De Chiara A, Giordano C, Perillo M, Fornino D, De Caro F, Capunzo M, Moccia G. Anti-SARS-CoV-2 vaccination campaign: risk perception, emotional states, and vaccine hesitancy in a sample of adolescents' vaccinated parents in Southern Italy. Vaccines. 2022 Jun 16;10(6):958.
732. Bağ Ö, Güney SE. Vaccine refusal risk factors among parents of children with autism spectrum disorders. The Turkish journal of pediatrics. 2023;65(2).
733. Bektas İ, Bektas M. The effects of parents' vaccine hesitancy and COVID-19 vaccine literacy on attitudes toward vaccinating their children during the pandemic. Journal of pediatric nursing. 2023 Apr 26.
734. Çelik T, Doğan D. COVID-19 Vaccine Hesitancy of Parents of Children with Type 1 Diabetes in Türkiye: A Mixed-Methods Study. Journal of Pediatric Infection/Cocuk Enfeksiyon Dergisi. 2023 Jun 1;17(2).
735. Duran S, Duran R, Acunaş B, Şahin EM. Changes in parents' attitudes towards childhood vaccines during the course of COVID-19 pandemic. Pediatrics International. 2023 Mar 5:e15520.
736. Šašić M, Bodulić K, Hojsak I, Mašić M, Trivić I, Markić J, Batinić M, Bartulović I, Šurina A, Krajcar N, Tešović G. Parents' attitudes toward childhood COVID-19 immunization in Croatia: a multicenter cross-sectional study. Croatian Medical Journal. 2023 Feb;64(1):52.
737. Şahin A, Aksay AK, Aşcı B, Keleş YE, Üstündağ G, Tüz AE, Taşar S, Maden AA, İnce G, Kanık A, Öncel EK. Attitudes of parents with children aged 12-18 to

COVID-19 vaccines for themselves and their children. Turkish Journal of Pediatrics. 2023 Mar 1;65(2).
738. Štrbac M, Vuković V, Pustahija T, Nikolić N, Rajčević S, Ilić S, Dugandžija T, Patić A, Ristić M, Petrović V. Motives and attitudes of parents toward HPV vaccination: Results from the initial period of HPV vaccine rollout in Serbia. Plos one. 2023 Jul 6;18(7):e0287295.
739. Ourania-Eleni Z, Papadopoulou A. Vaccination: An Essential Protection of Children or a Free Decision of Parents? Cause Behind Parents' Decision to Not Vaccinate Their Children, in Greece. The Educational Review, USA. 2022;6(9):464-72.
740. Esposito S, Rosafio C, Partesotti S, Fiore M, Antodaro F, Bergomi A, Neglia C, Argentiero A, Principi N, Zona S. Knowledge on Parental Hesitancy toward COVID-19 Vaccination of Children 5–11 Years Old. Vaccines. 2023 Mar 3;11(3):587.
741. Ganem F, Folch C, Colom-Cadena A, Bordas A, Alonso L, Soriano-Arandes A, Casabona J, Sentinel School Network Study Group of Catalonia. Determinants of COVID-19 vaccine hesitancy among students and parents in Sentinel Schools Network of Catalonia, Spain. Plos one. 2023 Mar 9;18(3):e0282871.
742. Grechukha YO, Gnyloskurenko GV, Lapii F, Volokha AP. Determinants Of Vaccine Hesitancy Among Parents In Kyiv. Indexed In Pubmed/Medline, Scopus, Embase, Ebsco, Index Copernicus, Polish Ministry Of Education And Science, Polish Medical Bibliography. 2023 Jan 1;76(3):527-33.
743. Gundogdu Z, Sezer OY. Changing Parental Attitudes Towards Rotavirus Vaccine. Cureus. 2023 Feb 23;15(2).
744. Zona S, Partesotti S, Bergomi A, Rosafio C, Antodaro F, Esposito S. Anti-COVID vaccination for adolescents: a survey on determinants of vaccine parental hesitancy. Vaccines. 2021 Nov 10;9(11):1309.
745. Russo L, Croci I, Campagna I, Pandolfi E, Villani A, Reale A, Barbieri MA, Raponi M, Gesualdo F, Tozzi AE. Intention of parents to immunize children against SARS-CoV-2 in Italy. Vaccines. 2021 Dec 11;9(12):1469.
746. Miguel I, Valentim JP, Carugati F, Selleri P. What influences representations on vaccines and children’s vaccination? A psychosocial study on mothers’ representations, values, and decision-making styles.
747. Marron L, Ferenczi A, O'Brien KM, Cotter S, Jessop L, Morrissey Y, Migone C. A national survey of parents’ views on childhood vaccinations in Ireland. Vaccine. 2023 Jun 7;41(25):3740-54.
748. Kiroplis I, Hickey O, Hayes P, O'Donnell P. Nasal flu vaccine: attitudes and perceptions of parents who use social media. Rural and Remote Health. 2023 Jan 10;23(1):8169-.
749. Akman N, Yıldız A. Evaluation of Mothers' Opinions on Routine Childhood Vaccinations. Cocuk Enfeksiyon Dergisi. 2022 Dec 1;16(4):E253-9.(Europe)
750. Smith LE, Sherman SM, Sim J, Amlôt R, Cutts M, Dasch H, Sevdalis N, Rubin GJ. Parents’ intention to vaccinate their child for COVID-19: A mixed-methods study (CoVAccS–wave 3). PLoS One. 2022 Dec 27;17(12):e0279285. (Europe)
751. Bagateli LE, Saeki EY, Fadda M, Agostoni C, Marchisio P, Milani GP. COVID-19 vaccine hesitancy among parents of children and adolescents living in Brazil. Vaccines. 2021 Sep 30;9(10):1115.

752. Benites-Zapata VA, Herrera-Añazco P, Benites-Meza JK, Bonilla-Aguilar K, Urrunaga-Pastor D, Bendezu-Quispe G, Uyen-Cateriano A, Rodriguez-Morales AJ, Hernandez AV. Prevalence of parents' non-intention to vaccinate their children and adolescents against COVID-19: A comparative analysis in Colombia and Peru. Vaccine: X. 2022 Dec 1;12:100198.
753. Brown AL, Sperandio M, Turssi CP, Leite R, Berton VF, Succi RM, Larson H, Napimoga MH. Vaccine confidence and hesitancy in Brazil. Cadernos de saude publica. 2018 Sep 21;34.
754. Burghouts J, Del Nogal B, Uriepero A, Hermans PW, de Waard JH, Verhagen LM. Childhood vaccine acceptance and refusal among Warao Amerindian Caregivers in Venezuela; A qualitative approach. PloS one. 2017 Jan 20;12(1):e0170227.
755. Gentile A, Pacchiotti AC, Giglio N, Nolte MF, Talamona N, Rogers V, Berenstein A, Castellano VE. Vaccine hesitancy in Argentina: validation of WHO scale for parents. Vaccine. 2021 Jul 30;39(33):4611-9.
756. Gonzales A, Choque D, Marcos-Carbajal P, Salvatierra G. Factors associated with diphtheria vaccination completion among children under five years old in Peru 2010–2019: A cross-sectional population-based study. Heliyon. 2022 Nov 1;8(11):e11370.
757. González-Block MÁ, Arroyo-Laguna J, Rodríguez-Zea B, Pelcastre-Villafuerte BE, Gutiérrez-Calderón E, Díaz-Portillo SP, Puentes-Rosas E, Sarti E. The importance of confidence, complacency, and convenience for influenza vaccination among key risk groups in large urban areas of Peru. Human vaccines & immunotherapeutics. 2021 Feb 1;17(2):465-74.
758. Martinez EZ, Zucoloto ML, Ramos VP, Dutra CD, de Jesus GJ, Esteves AV, Abreu IS, Mombelli MA, Reis RA, Campoamor MM, da Silva WR. Brazilian Adults' Attitudes and Practices Regarding the Mandatory COVID-19 Vaccination and Their Hesitancy towards Childhood Vaccination. Vaccines. 2022 Nov 1;10(11):1853.
759. Nehab MF, Camacho KG, Reis AT, de Fátima Junqueira-Marinho M, Abramov DM, de Azevedo ZM, dos Santos Salú M, de Vasconcelos ZF, da Silva Filho OC, de Oliveira Salvador PT, Alves KY. Willingness of Brazilian caregivers in having their children and adolescents vaccinated against Covid-19. Vaccine. 2023 Jan 16;41(3):735-43.
760. Chung-Delgado K, Venero JE, Vu TM. Vaccine hesitancy: characteristics of the refusal of childhood vaccination in a Peruvian population. Cureus. 2021 Mar 25;13(3).
761. Logullo P, Carvalho HB, Saconi R, Massad E. Factors affecting compliance with the measles vaccination schedule in a Brazilian city. Sao Paulo Medical Journal. 2008;126:166-71.
762. Rodrigues ES, Mendes ED, Nucci LB. Parental Justifications for Not Vaccinating Children or Adolescents against Human Papillomavirus (HPV). Vaccines. 2023 Feb 22;11(3):506.
763. Bono SA, Siau CS, Chen WS, Low WY, Faria de Moura Villela E, Pengpid S, Hasan MT, Sessou P, Ditekemena JD, Amodan BO, Hosseinipour MC. Adults' acceptance of COVID-19 vaccine for children in selected lower-and middle-income countries. Vaccines. 2021 Dec 22;10(1):11.

764. Olbrich Neto J, Olbrich SR. Attitudes, hesitancy, concerns, and inconsistencies regarding vaccines reported by parents of preschool children. Revista Paulista de Pediatria. 2023 Mar 13;41:e2022009.
765. Kuan-Mahecha MA, Rahman S, Martínez-Rivera P, Lamb MM, Asturias EJ. Differences in parental vaccine confidence and attitudes by health system in Guatemala and their impact on immunization timeliness. Vaccine. 2023 May 5;41(19):3099-105.
